# Supplementary material for: Photoinduced site-selective alkenylation of alkanes and aldehydes with aryl alkenes
Source: Nat Commun. 2020 Apr 23;11:1956. doi: 10.1038/s41467-020-15878-6 (PMC7181776; doi:10.1038/s41467-020-15878-6)
Supplement: Supplementary file 1 — Supplementary Information [file 41467_2020_15878_MOESM1_ESM.pdf]

Supplementary Information

Photo-Induced Site-Selective Alkenylation of Alkanes and Aldehydes with Aryl Alkenes

Cao *et al.*

Supplementary Methods

General information

Chemicals and anhydrous solvents were purchased from commercial suppliers and used as received. Tetrabutylammonium decatungstate[1] and cobalt catalysts[2] were synthesized according to literature. Uncommercial available substrates were synthesized according to literature. ^{1}H NMR, ^{13}C NMR, ^{19}F NMR, COSY NMR, NOESY NMR, HMQC NMR, HMBC NMR spectra were recorded on a Bruker AV-III400 (400 MHZ) or AMX500 (500 MHz) spectrometer. Chemical shifts were reported in parts per million (ppm) and calibrated using residual undeuterated solvent as an internal reference ($CDCl_3$: 7.26 ppm ^{1}H NMR, 77.0 ppm ^{13}C NMR). Multiplicity was indicated as follows: s (singlet), d (doublet), t (triplet), q (quartet), m (multiplet), dd (doublet of doublet), dt (doublet of triplet), td (triplet of doublet), ddd (doublet of doublet of doublet), dddd (doublet of doublet of doublet of doublet). All high resolution mass spectra (HRMS) were obtained on a Finnigan/MAT 95XL-T spectrometer. Gas Chromatography-Mass Spectrometry (GC-MS) was performed on Aglilent 7820A with FID detection. Hydrogen gas detection was performed on Aglilent GC7890 with TCD detector. Hydrogen gas quantification was performed on Shimadzu GC-2010 Plus. UV-Vis absorption spectra were taken at ambient temperature using Edinburgh FS5 spectrofluorometer. Cyclic voltammetry was performed on Ametek VersaSTAT 3. Flash column chromatography were performed on Merck 60 (0.040-0.063 mm) mesh silica gel and run under positive air pressure. Analytical thin layer chromatography (TLC) was performed with Merck pre-coated TLC plates (silica gel 60F-254, layer thickness 0.25 mm). Visualization was achieved by short wave (254 nm) ultraviolet light or by staining with potassium permanganate ($KMnO_4$) or Phosphomolybdic Acid (PMA), followed by heating.

24W 370 nm LED strip (2.5 meter) was purchased from UnvarySam. More information can be found on: https://www.aliexpress.com/item/UnvarySam-Ultraviolet-LED-Strip-365nm-370nm-375NM-380NM-385NM-5M-12V-SMD3528-600LEDs-UV-Ultraviolet-for/32859140719.html

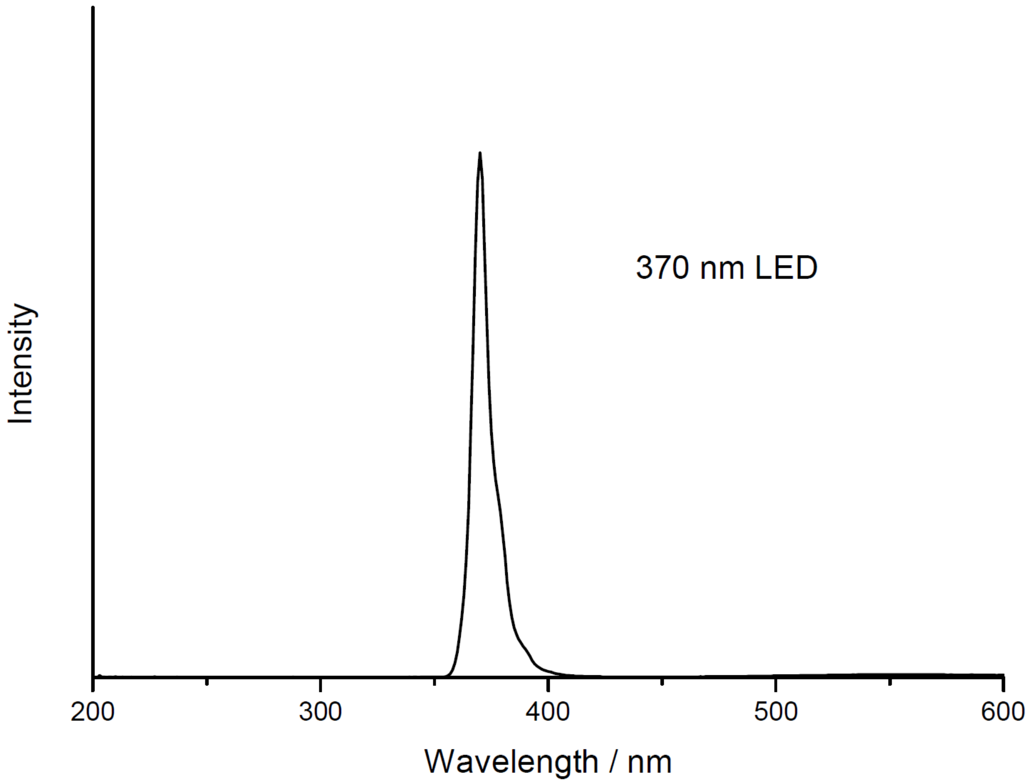

Supplementary Fig. 1 Emission spectrum of 24 W 370 nm LED strips. Maximum emission at around 370 nm.

Experimental procedure

General procedure for dehydrogenative alkenylation of alkanes and aldehydes with alkenes. To a 10 mL oven-dried Schlenk tube equipped with a magnetic stir bar was added the corresponding C-H nucleophile (0.2 mmol, 1.0 equiv), alkene (2.0 mmol, 10 equiv), TBADT (26.6 mg, 0.008 mmol, 4 mol%), Co(dmgH)(dmgH_2)Cl_2 (0.7 mg, 0.002 mmol, 1 mol%), 2,6-lutidine (2.1 mg, 0.02 mmol, 10 mol%) and dry acetonitrile (2 mL). The resulting mixture was cooled to 0 ^{o}C using an ice-water bath, and bubbled with argon balloon for 10 min (if the C-H nucleophile was volatile, it was added after bubbling). After that, the reaction was placed under a 370 nm LED (2.5 meter strips, 24 W), stirred and irradiated under argon atmosphere. The temperature was maintained at 60 °C using a water bath. The reaction mixture was removed from light and quenched by stirring open to air for 5 minutes. The solvent was removed on a rotary evaporator under reduced pressure and the residue was subjected to column chromatography isolation over silica gel or preparative thin layer chromatography to give the corresponding product.

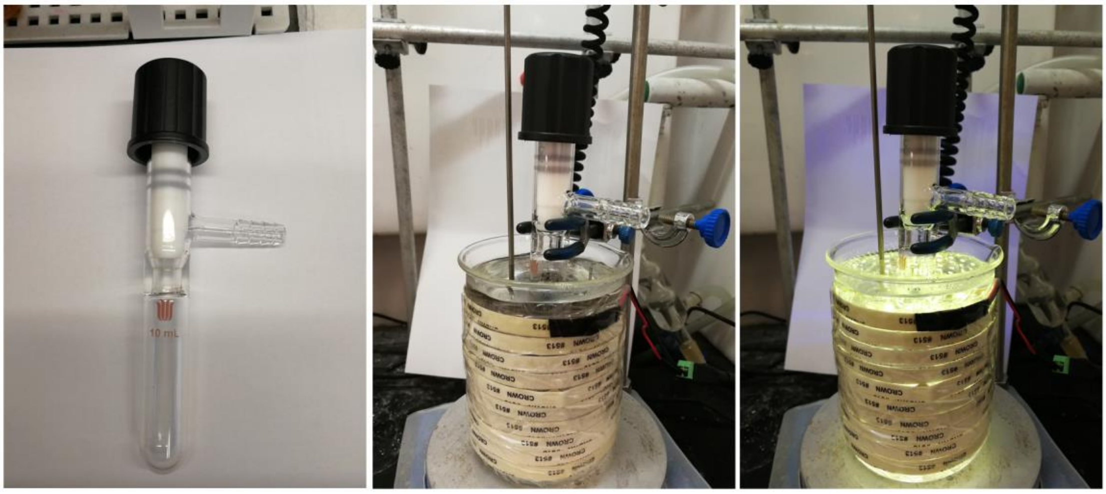

Supplementary Fig. 2 Reaction vessel and reaction set-up. A 10 mL Schlenk tube was used in this study.

Preparation of tetrabutylammonium decatungstate (TBADT):

To a 2 L beaker wrapped in aluminum foil for insulation and equipped with a stir bar were added tetrabutylammonium bromide (4.80 g, 14.9 mmol, 0.49 equiv) and deionized water (1600 mL). In a separate 4 L beaker wrapped in aluminum foil for insulation and equipped with a stir bar were added Na_2WO_4•$2H_2O$ (10 g, 30.3 mmol, 1.0 equiv) and deionized water (1600 mL). Both solutions were rapidly stirred and heated to 90 °C. When both solutions reached 90 °C, concentrated HCl was added to each solution until pH stabilized at 2. At this point, the acidified solutions were combined in the 4 L beaker, and the resultant suspension was stirred at 90 °C for an additional 30 minutes. The reaction mixture was cooled to room temperature, then filtered through a pad of silica gel. The solids were washed with water and left to dry under vacuum. When the silica-supported solids were dry, the receiving flask was exchanged, and the pad was washed with 3 x 200 mL acetonitrile. The filtrate was collected and solvent was removed. The crude residue was thoroughly dried under vacuum, dissolved in minimal hot acetonitrile, then placed in the freezer at –20 °C for 12 hours. The solids were collected on a filter, washed with minimal cold acetonitrile, then dried under vacuum. The filtrate was reconcentrated, dissolved in minimal hot acetonitrile, and crystallized again to afford a second crop of TBADT. Isolated as white solid, 1st crop (6.41 g, 1.934 mmol, 64%), 2nd crop (2.36 g, 0.711 mmol, 87% combined yield). UV-Vis and cyclic voltammetry characterization is consistent

with literature data.[1]

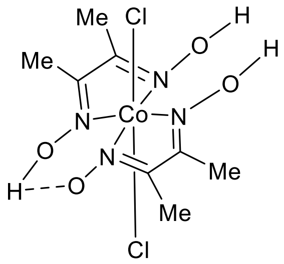

Preparation of Co(dmgH)(dmgH$_2$)Cl$_2$:

In air, to a 100 mL round-bottom flask charged with $CoCl_2$ (1.3 g, 10 mmol, 1.0 equiv) and acetone (50 mL) was added dimethylglyoxime (2.6 g, 22 mmol, 2.2 equiv). A gentle stream of air was passed through the solution via a needle. After 30 minutes, the reaction flask was placed on ice for 20 minutes. The resulting green crystals were collected by filtration over a Büchner funnel. The collected crystals were rinsed with cold acetone (5 mL) and further dried at room temperature under vacuum to afford 3.0 g of a green solid (83% yield). Characterization data is consistent with literature.[2]

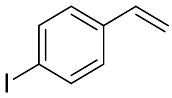

Preparation of 1-iodo-4-vinylbenzene: To a solution of 4-vinylphenylboronic acid (2 mmol, 296 mg) in dry acetonitrile (10 mL), *N*-iodosuccinimide (2.0 mmol, 450 mg) was added and the mixture was stirred at 81 ^{o}C protected from air and light until completion of the reaction was confirmed by TLC (silica gel, hexane : ethyl acetate = 1:1). After the reaction was completed, the reaction mixture was extracted with hexane (3 x 50 mL). The collected hexane extracts were washed with distilled water, saturated aqueous solution of $Na_2S_2O_3$, saturated aqueous solution of $NaHCO_3$ and finally dried over $MgSO_4$. Filtration and evaporation of the solvent in vacuo gave final product as a light yellow solid (287.9 mg, 63 %).

^{1}H NMR (400 MHz, $CDCl_3$) δ 7.67 – 7.63 (m, 2H), 7.16 – 7.12 (m, 2H), 6.64 (dd, J = 17.6, 10.8 Hz, 1H), 5.75 (dd, J = 17.6, 0.8 Hz, 1H), 5.27 (dd, J = 10.8, 0.8 Hz, 1H).

Spectral data is in agreement with the literature.[3]

General procedure for Wittig olefination: A suspension of methyltriphenylphosphonium bromide (2.4 mmol) in 15 mL of THF under inert atmosphere was cooled at 0ºC with an ice bath. Then, *n*-BuLi (2.4 mmol, 0.96 mL, 2.5M in hexanes) was added dropwise. The reaction was stirred for 40 minutes at 0 ºC and then the corresponding aldehyde (2 mmol dissolved in 5 mL of THF) was added dropwise. The reaction was stirred for 12 hours at room temperature. After that, the reaction was quenched with a saturated aqueous solution of ammonium chloride and extracted with Et_2O. The organic phases were collected, washed with brine and dried over magnesium sulfate. The solvent was removed under reduced pressure and the crude product was purified by flash column chromatography (hexanes/ethyl acetate mixtures) over silica gel.

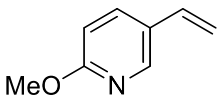

Preparation of 2-methoxy-5-vinylpyridine: Following the general procedure for Wittig olefination using methyltriphenylphosphonium bromide and 6-methoxy-3-pyridinecarboxaldehyde, affording the product as yellow oil (178 mg, 66%).

^{1}H NMR (400 MHz, $CDCl_3$) δ 8.12 (d, J = 2.0 Hz, 1H), 7.69 (dd, J = 8.8, 2.4 Hz, 1H), 6.72 (d, J = 8.8 Hz, 1H), 6.65 (dd, J = 17.6, 11.2 Hz, 1H), 5.64 (d, J = 17.6 Hz, 1H), 5.21 (d, J = 11.2 Hz, 1H), 3.94 (s, 3H).

Spectral data is in agreement with the literature.[4]

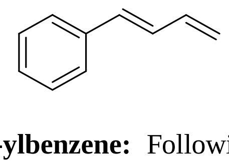

Preparation of (*E*)-buta-1,3-dien-1-ylbenzene: Following the general procedure for Wittig olefination using methyltriphenylphosphonium bromide, *E*-cinnamyl aldehyde to give the product as colorless oil (234 mg, 90% yield).

^{1}H NMR (400 MHz, $CDCl_3$) δ 7.45 – 7.41 (m, 2H), 7.37 – 7.31 (m, 2H), 7.28 – 7.22 (m, 1H), 6.82 (ddt, J = 15.6, 10.8, 0.8 Hz, 1H), 6.59 (d, J = 15.6 Hz, 1H), 6.54 (dt, J = 16.8, 10.8 Hz, 1H), 5.36 (ddd, J = 16.8, 1.6, 0.8 Hz, 1H), 5.20 (ddd, J = 10.8, 1.6, 0.8 Hz, 1H).

Spectral data is in agreement with the literature.[5]

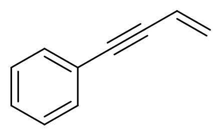

Preparation of but-3-en-1-yn-1-ylbenzene: CuI (55 mg, 0.29 mmol, 0.019 equiv) and $Pd(PPh_3)_4$ (55 mg, 0.048 mmol, 0.0032 equiv) were dissolved in diethylamine (7.5 mL, freshly distilled over KOH) at 10 °C under argon. Phenyl acetylene (1.6 mL, 15 mmol, 1.0 equiv) and vinyl bromide (1 M in THF, 20 mL, 20 mmol, 1.3 equiv) were added dropwise and the resulting suspension was stirred at 23 °C for 15 h. The reaction mixture was then poured into water (50 mL) at 0 °C and extracted with Et_2O (3x40 mL). The combined organic layers were washed with 2 M HCl (2 x 20 mL), dried over anhydrous $MgSO_4$ and concentrated under reduced pressure. The crude mixture was purified by chromatography over silica gel (hexane) to give product as a colorless oil (1.55 g, 12.1 mmol, 83%).

^{1}H NMR (400 MHz, $CDCl_3$) δ 7.50 – 7.42 (m, 2H), 7.38 – 7.27 (m, 3H), 6.03 (dd, J = 17.6, 11.2 Hz, 1H), 5.74 (dd, J = 17.6, 2.0 Hz, 1H), 5.55 (dd, J = 11.2, 2.0 Hz, 1H).

Spectral data is in agreement with the literature.[6]

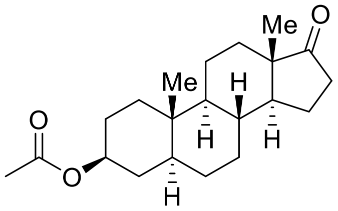

Preparation of (3*S*,5*S*,8*R*,9*S*,10*S*,13*S*,14*S*)-10,13-dimethyl-17-oxohexadecahydro-1*H*-cyclopenta[*a*]phenanthren-3-yl acetate: To an ice cold solution of 3*β*-hydroxy-5*α*-androstan-17-one (2.5 g, 8.6 mmol) in pyridine (15 ml) was added acetic anhydride (2.64 g, 25.8 mmol) followed by stirring for 12 h at rt. The reaction mixture was then poured to a mixture of ice-water (200 mL) and the resulting white precipitate was filtered, washed with water and dried under suction to afford the pure compound (2.78 g, 97%).

^{1}H NMR (400 MHz, $CDCl_3$) δ 4.77 – 4.59 (m, 1H), 2.43 (dd, J = 19.2, 8.4 Hz, 1H), 2.07 (dd, J =

19.2, 9.6 Hz, 1H), 2.02 (s, 3H), 1.97 – 1.88 (m, 1H), 1.86 – 1.13 (m, 16H), 1.09 – 0.90 (m, 2H), 0.85 (s, 3H), 0.85 (s, 3H), 0.76 – 0.65 (m, 1H).

Spectral data is in agreement with the literature.[7]

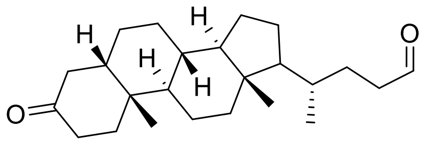

Preparation of (4*S*)-4-((5*S*,8*S*,9*R*,10*R*,13*S*,14*R*)-10,13-dimethyl-3-oxohexadecahydro-1*H*-cyclopenta[*a*]phenanthren-17-yl)pentanal: To a stirred solution of LAH (lithium aluminium hydride) (5.32 mmol, 4 equiv) in dry THF (15 ml), a solution of lithocholic acid (500 mg, 1.33 mmol) in dry THF (15 ml) was added dropwise under N_2. After 24h, saturated Na_2SO_4 solution was added dropwise to the reaction mixture until a white precipitate formed. The solid was filtered off. The filtrate was concentrated, the residue was taken up in ether, washed with H_2O, dried over $MgSO_4$ and evaporated to dryness to obtain the diol as a white amorphous solid (452 mg, 94%). The diol was directly used in the next step without characterization. A solution of the diol (200 mg, 0.55 mmol) in dry DCM (25 ml) was treated with PCC (pyridinium chlorochromate, 1.10 mmol, 2 equiv), and the mixture was refluxed for 18h under N_2. The reaction was then quenched with H_2O and extracted with ether. The ethereal solution was washed with H_2O, $NaHCO_3$ and brine, dried over $MgSO_4$. Purification by column chromatography (hexane/ethyl acetate = 5:1) gave the title compound (154 mg, 78%).

^{1}H NMR (500 MHz, $CDCl_3$) δ 9.78 – 9.75 (m, 1H), 2.74 – 2.64 (m, 1H), 2.52 – 2.41 (m, 1H), 2.40 – 2.27 (m, 2H), 2.20 – 2.12 (m, 1H), 2.08 – 1.97 (m, 3H), 1.92 – 1.76 (m, 4H), 1.66 – 1.56 (m, 2H), 1.53 – 1.06 (m, 13H), 1.02 (s, 3H), 0.99 – 0.82 (m, 4H), 0.68 (s, 3H). ^{13}C NMR (126 MHz, $CDCl_3$) δ 213.37, 203.12, 56.43, 56.00, 44.30, 42.80, 42.36, 40.93, 40.73, 40.04, 37.20, 37.00, 35.53, 35.31, 34.88, 28.21, 27.93, 26.61, 25.76, 24.14, 22.64, 21.18, 18.38, 12.07.

Spectral data is in agreement with the literature.[8]

Supplementary Table 1 Evaluation of the amount of styrene.

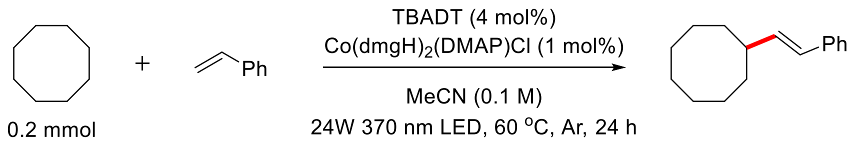

entry	styrene	yield (%)[a]	*E*/*Z*[b]
1	2 equiv	9	> 99:1
2	5 equiv	22	> 99:1
3	8 equiv	36	> 99:1
**4**	**10 equiv**	**43**	**> 99:1**
5	12 equiv	42	> 99:1
6	20 equiv	41	> 99:1
7[c]	20 equiv	40	> 99:1

[a]Yields were determined by crude ^{1}H NMR spectra using 1,3,5-trimethoxybenzene as an internal standard. [b]*E*/*Z* Ratios were determined by GC analysis of the crude mixtures. [c]Reaction time 38 hour.

Supplementary Table 2 Evaluation of reaction concentration.

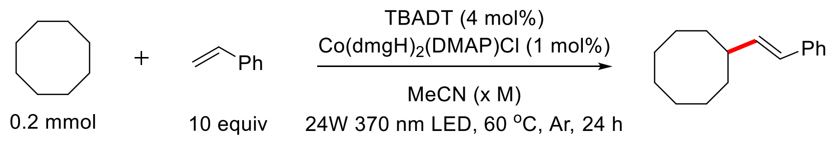

entry	x	yield (%)[a]	*E*/*Z*[b]
1	0.04	38	> 99:1
2	0.05	40	> 99:1
3	0.067	41	> 99:1
**4**	**0.1**	**43**	**> 99:1**
5	0.2	37	> 99:1

[a]Yields were determined by crude ^{1}H NMR spectra using 1,3,5-trimethoxybenzene as an internal standard. [b]*E*/*Z* Ratios were determined by GC analysis of the crude mixtures.

Supplementary Table 3 Evaluation of temperature and time.

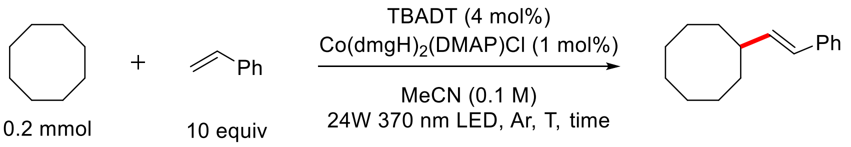

entry	temperature	time	yield (%)[a]	*E*/*Z*[b]
1	30 °C	12 h	21	> 99:1
2	30 °C	21 h	30	> 99:1
3	30 °C	30 h	34	> 99:1
4	30 °C	45 h	32	> 99:1
5	45 °C	20 h	28	> 99:1
6	45 °C	24 h	30	> 99:1
7	45 °C	28 h	38	> 99:1
8	60 °C	12 h	32	> 99:1
9	60 °C	21 h	39	> 99:1
**10**	**60 °C**	**24 h**	**43**	**> 99:1**
11	60 °C	30 h	39	> 99:1
12	60 °C	45 h	34	> 99:1
13	75 °C	20 h	29	> 99:1
14	75 °C	24 h	25	> 99:1

[a]Yields were determined by crude ^{1}H NMR spectra using 1,3,5-trimethoxybenzene as an internal standard. [b]*E*/*Z* Ratios were determined by GC analysis of the crude mixtures.

Supplementary Table 4 Evaluation of solvent.

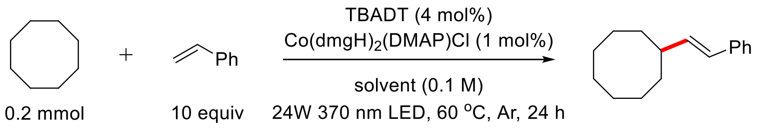

entry	solvent	yield (%)[a]	*E*/*Z*[b]
**1**	**MeCN**	**43**	**> 99:1**
2	MeCN/DCE = 9/1 (v/v)	<10	> 99:1
3	MeCN/$PhCF_3$ = 9/1 (v/v)	31	> 99:1
4	MeCN/H_2O = 9/1 (v/v)	39	> 99:1
5	Acetone	29	> 99:1
6	Acetone/H_2O = 9/1 (v/v)	33	> 99:1
7	$MeNO_2$	<10	> 99:1
8	$PhCF_3$, CF_3CH_2OH or DMSO	0	> 99:1

[a]Yields were determined by crude ^{1}H NMR spectra using 1,3,5-trimethoxybenzene as an internal standard. [b]*E*/*Z* Ratios were determined by GC analysis of the crude mixtures.

Supplementary Table 5 Evaluation of catalyst loading.

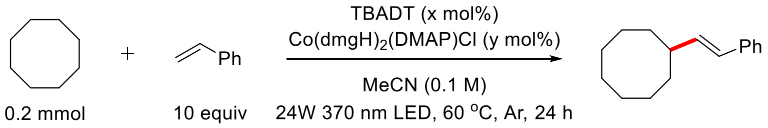

entry	x	y	yield (%)[a]	*E*/*Z*[b]
**1**	**4**	**1**	**43**	**> 99:1**
2	4	2	35	> 99:1
3	4	0.5	25	> 99:1
4	2	1	33	> 99:1
5	6	1	37	> 99:1
6	8	1	40	> 99:1
7	8	2	32	> 99:1

[a]Yields were determined by crude ^{1}H NMR spectra using 1,3,5-trimethoxybenzene as an internal standard. [b]*E*/*Z* Ratios were determined by GC analysis of the crude mixtures.

Supplementary Table 6 Evaluation of light sources.

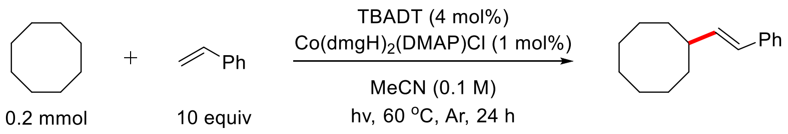

entry	light source	yield (%)[a]	*E*/*Z*[b]
**1**	**24W 370 nm LED**	**43**	**> 99:1**
2	25W UVB lamp	17	> 99:1
3	2 x 40W UVA lamp	43	> 99:1
4	2 x 40W Kessil 370 nm	31	> 99:1
5	2 x 40W Kessil 390 nm	30	> 99:1

[a]Yields were determined by crude ^{1}H NMR spectra using 1,3,5-trimethoxybenzene as an internal standard. [b]*E*/*Z* Ratios were determined by GC analysis of the crude mixtures.

25W UVB lamp (Exo-Terra Reptile UVB 150) was purchased from Exo-Terra. The spectral output of the Exo-Terra Reptile 150 lamp can be found online at: http://www.exo-terra.com/en/products/reptile_uvb150.php

40W UVA lamp (330-410 nm, emission maxima at 366 nm) was purchased from Gongben. More information can be found on: https://www.aliexpress.com/item/E27-40W-220V-Ultraviolet-light-energy-saving-bulb-spiral-quartz-Fluorescent-UV-black-light-Violet-CFL/32833153617.html?spm=a2g0s.9042311.0.0.2af74c4dNt97Fp

Kessil PR160-370 and Kessil PR160-390 lights were purchased from Kessil. More information and spectral output can be found on: http://kessil.com/photoredox/Products.php

Supplementary Table 7 Evaluation of cobalt catalysts.

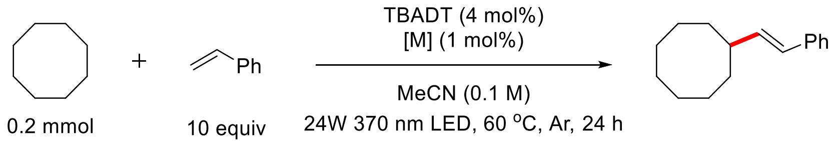

entry	[M]	yield (%)[a]	*E*/*Z*[b]
1	$Co(dmgH)(dmgH_2)Cl_2$	34	> 99:1
2	$Co(dmgH)_2PyCl$	42	> 99:1
**3**	**$Co(dmgH)_2(DMAP)Cl$**	**43**	**> 99:1**
4	$Co(dmgH)_2$(4-COOMePy)Cl	37	> 99:1
5	$Co(dmgH)_2$(4-MeOPy)Cl	39	> 99:1
6	$Co(dmgBF_2)_2$•2MeCN	16	> 99:1
7	$Co(dmgH)_2Py_2PF6$	35	> 99:1
8	$Co(gH)_2(DMAP)Cl$	33	> 99:1
9	$Co(dpgH)_2(DMAP)Cl$	27	> 99:1
10	Co(salen)	0	-
11	Methylcobalamin or Cyanocobalamin	0	-
12	$Pt(cod)Cl_2$, $Pd(TFA)_2$ or $Ni(OTf)_2$	0	-

[a]Yields were determined by crude ^{1}H NMR spectra using 1,3,5-trimethoxybenzene as an internal standard. [b]*E*/*Z* Ratios were determined by GC analysis of the crude mixtures.

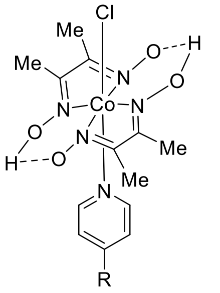

R=H, Co(dmgH)$_2$PyCl
R=COOMe, Co(dmgH)$_2$(4-COOMePy)Cl
R=NMe$_2$, Co(dmgH)$_2$(DMAP)Cl
R=OMe, Co(dmgH)$_2$(4-MeOPy)Cl

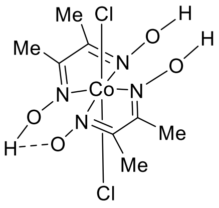

Co(dmgH)(dmgH$_2$)Cl$_2$

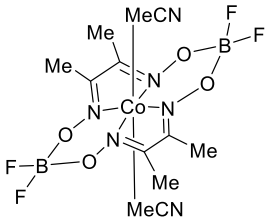

Co(dmgBF$_2$)$_2$·2MeCN

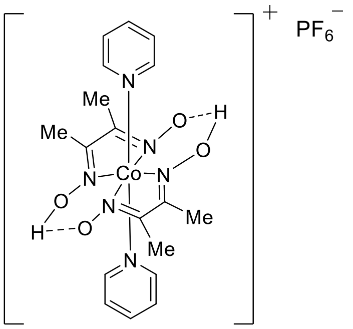

Co(dmgH)$_2$Py$_2$PF$_6$

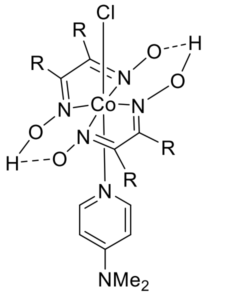

R=H, Co(gH)$_2$(DMAP)Cl
R=Ph, Co(dpgH)$_2$(DMAP)Cl

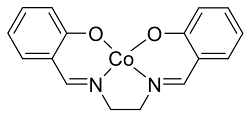

Co(salen)

Co(dmgH)$_2$PyCl[2], Co(dmgH)$_2$(DMAP)Cl[2], Co(dmgH)$_2$(4-COOMePy)Cl[2], Co(dmgH)$_2$(4-MeOPy)Cl[2], Co(dmgBF$_2$)$_2$·(MeCN)$_2$[9], Co(dmgH)$_2$Py$_2$PF$_6$[10], Co(gH)(gH$_2$)Cl$_2$[11], Co(gH)$_2$(DMAP)Cl[11], Co(dpgH)(dpgH$_2$)Cl$_2$[12] and Co(dpgH)$_2$(DMAP)Cl[12] were synthesized according to literature.

Supplementary Table 8 Evaluation of axial ligands for Co(dmgH)(dmgH$_2$)Cl$_2$.[a]

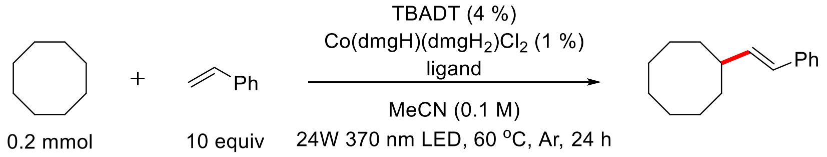

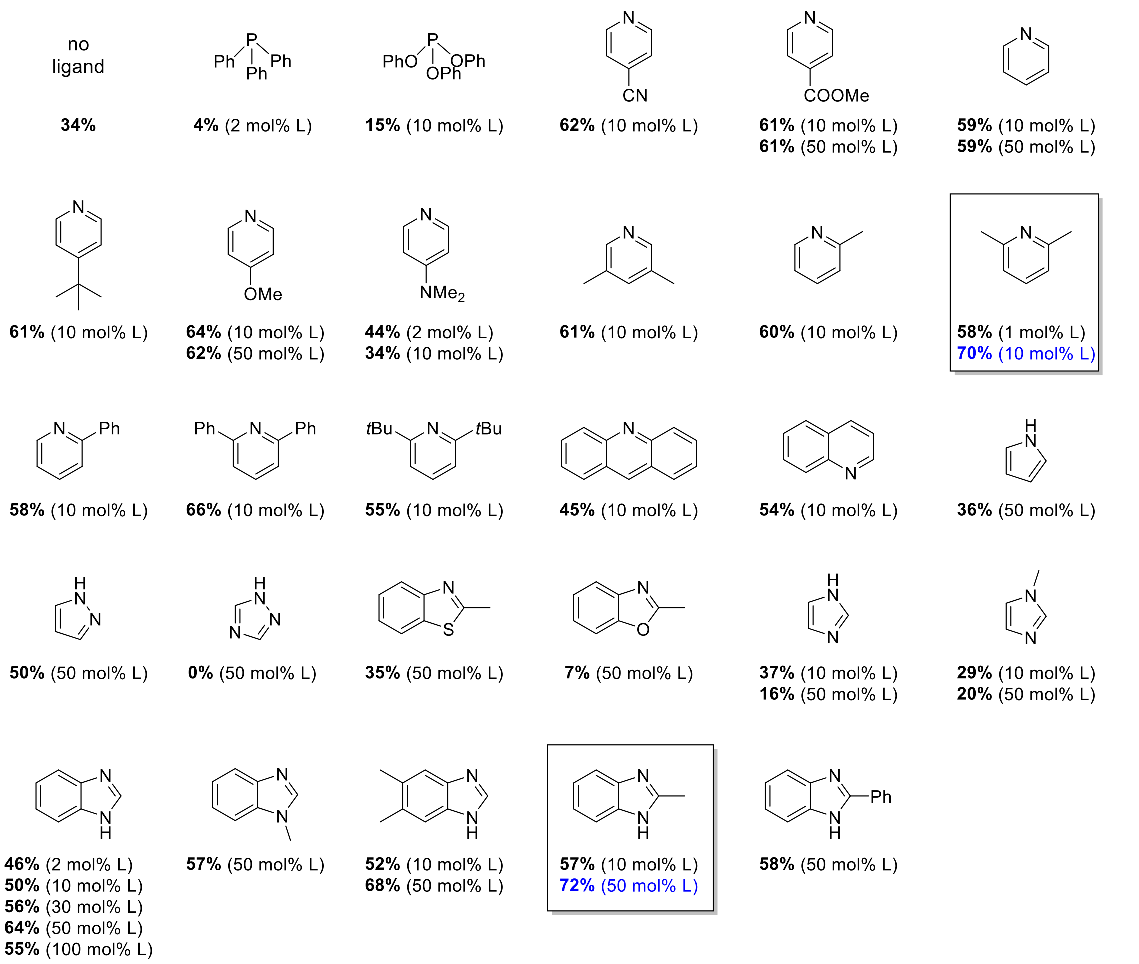

[a]Yields were determined by crude ^{1}H NMR spectra using 1,3,5-trimethoxybenzene as an internal standard. *E*/*Z* Ratios were determined by GC analysis and were >99:1 in all cases.

Supplementary Table 9 Control experiments on the role of pyridine ligand.

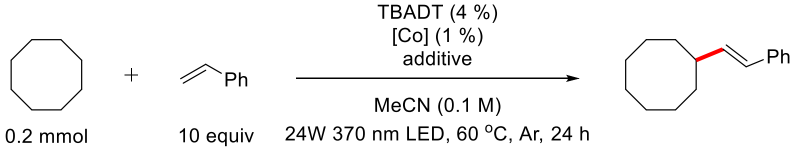

entry	[Co]	additive	yield (%)[a]	*E*/*Z*[b]
1	Co(dmgH)(dmgH$_2$)Cl$_2$	-	34	> 99:1
2	Co(dmgH)(dmgH$_2$)Cl$_2$	10 mol% 4-methoxypyridine	64	> 99:1
3	Co(dmgH)(dmgH$_2$)Cl$_2$	10 mol% $NaHCO_3$	34	> 99:1
4	Co(dmgH)(dmgH$_2$)Cl$_2$	50 mol% $NaHCO_3$	36	> 99:1
5	Co(dmgH)$_2$(4-MeOPy)Cl	-	39	> 99:1
6	Co(dmgH)$_2$(4-MeOPy)Cl	10 mol% 4-methoxypyridine	63	> 99:1
7	Co(dmgH)$_2$(4-MeOPy)Cl	10 mol% $NaHCO_3$	40	> 99:1
8	Co(dmgH)$_2$(4-MeOPy)Cl	50 mol% $NaHCO_3$	43	> 99:1
9	Co(dmgH)$_2$PyCl	-	42	> 99:1
10	Co(dmgH)$_2$PyCl	10 mol% $NaHCO_3$	43	> 99:1
11	Co(dmgH)$_2$(DMAP)Cl	-	43	> 99:1
12	Co(dmgH)$_2$(DMAP)Cl	10 mol% $NaHCO_3$	43	> 99:1

[a]Yields were determined by crude ^{1}H NMR spectra using 1,3,5-trimethoxybenzene as an internal standard. [b]*E*/*Z* Ratios were determined by GC analysis of the crude mixtures.

Supplementary Table 10 Control experiments on reaction parameters.

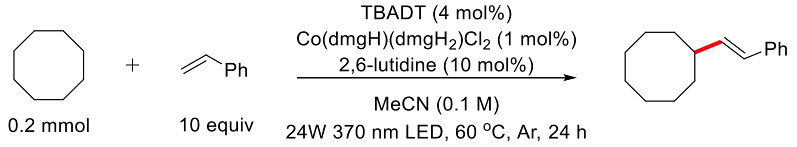

entry	variation from standard condition	yield (%)[a]	*E*/*Z*[b]
1	none	70%	> 99:1
2	no TBADT	0	-
3	no $Co(dmgH)(dmgH_2)Cl_2$	0	-
4	no 2,6-lutidine	34	> 99:1
5	no light	0	-
6	under air atmosphere	32	> 99:1
7	$CoCl_2$ instead of $Co(dmgH)(dmgH_2)Cl_2$	0	-
8	argon degassing instead of freeze-pump-thaw	70%	> 99:1

[a]Yields were determined by crude ^1H NMR spectra using 1,3,5-trimethoxybenzene as an internal standard. [b]*E*/*Z* Ratios were determined by GC analysis of the crude mixtures.

Supplementary Table 11 Attempted alkenylation using different types of alkenes.

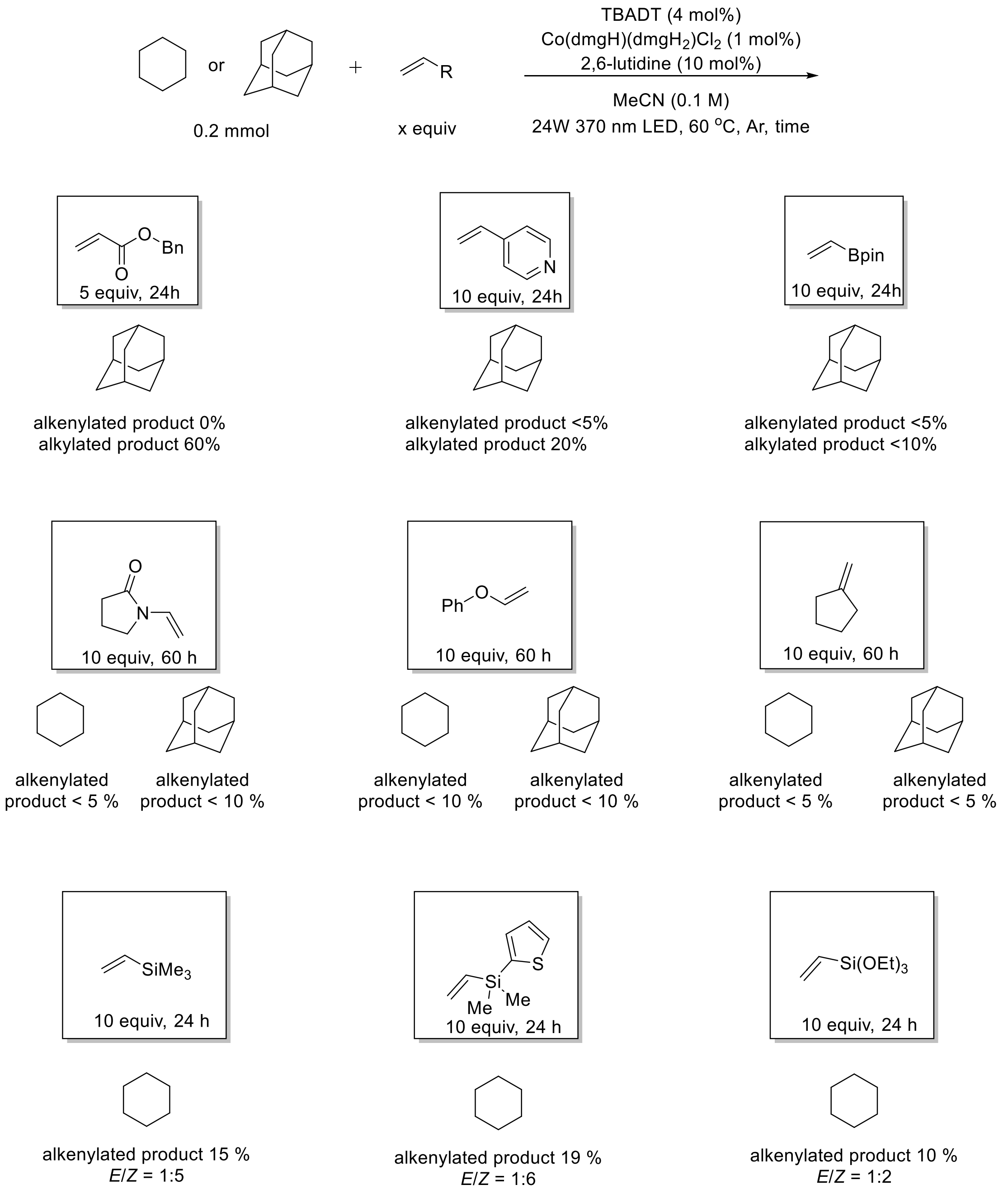

[a]Yields were determined by crude ^{1}H NMR spectra using 1,3,5-trimethoxybenzene as an internal standard. [b]*E*/*Z* Ratios were determined by GC analysis of the crude mixtures.

Supplementary Discussion

Elucidation on whether styrene serves as a hydrogen acceptor

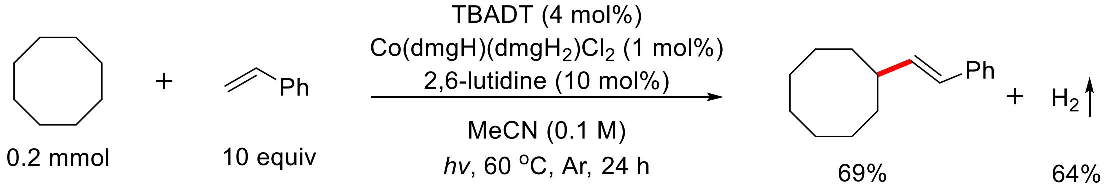

Supplementary Fig. 3 Detection and quantitative analysis of generated hydrogen gas. Dehydrogenative alkenylation of cyclooctane with styrene in a 10 mL microwave tube equipped with a penetrable septum. The upper atmosphere was analyzed by GC after 24 h of 370 nm LED irradiation.

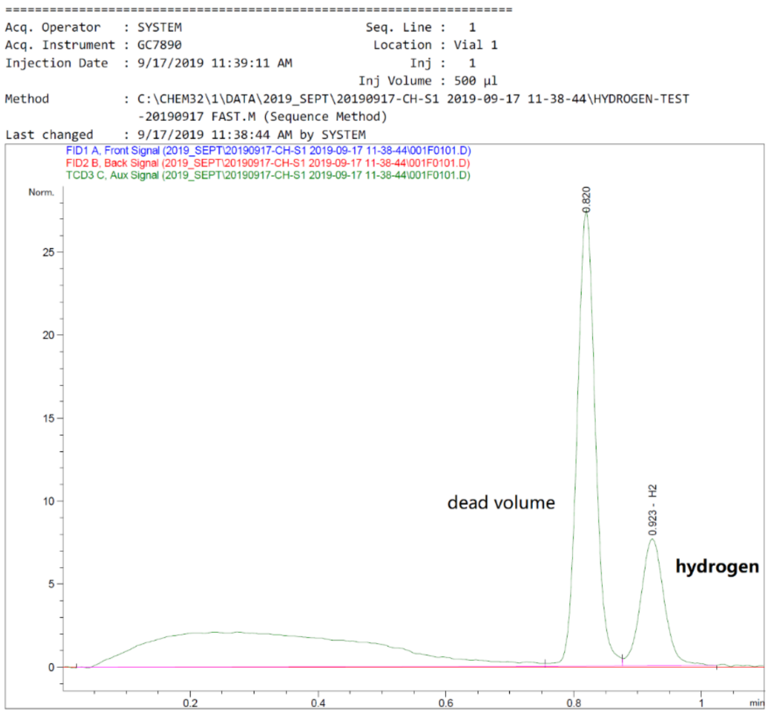

Supplementary Fig. 4 Detection of hydrogen gas by Aglilent GC7890. Retention time of pure H_2 gas is 0.923 min.

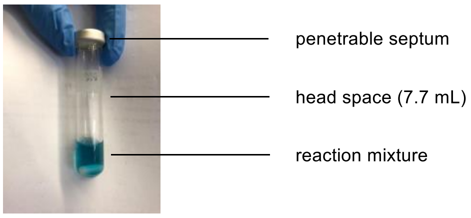

Ret. Time	Area	Height	Conc.	Unit	Vol. of Gas in 7.7 mL headspace (mL)	No. of μmol	Yield
1.712	887082.6	3636126.8	39.77201	%	3.062444	127.6011	64%

Supplementary Fig. 5 Quantitative analysis of generated hydrogen gas by Shimadzu GC-2010 Plus. The gas phase in the headspace (headspace = 7.7 mL) of the reaction vessel was analyzed by gas chromatography. A gas-tight syringe was used to take a sample (0.10 mL) from the vessel. Before each sampling, the gas in the syringe was replaced with the gas in the reaction vessel; 0.10 mL of gas in the headspace of the vessel was once taken and discharged. Then, 0.10 mL of gas in the vessel was newly taken and injected into a gas chromatograph.

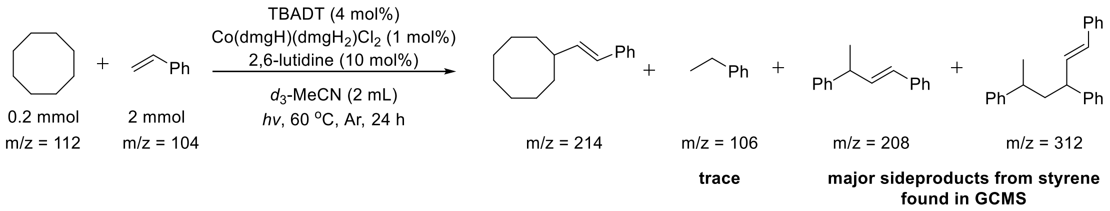

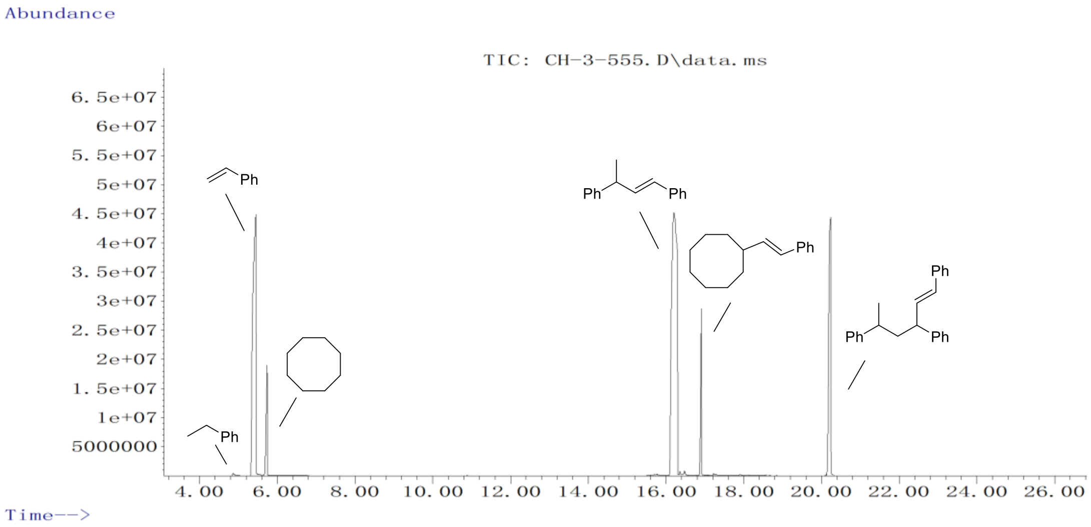

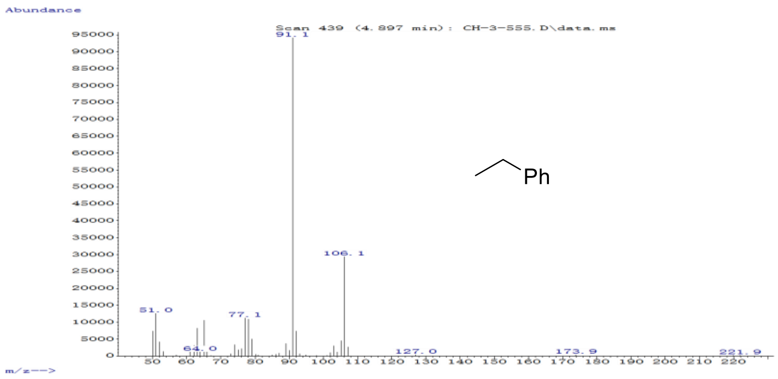
Abundance
Scan 439 (4.897 min): CH-3-555.D\data.ms
91.1
106.1
51.0
77.1
64.0
127.0
173.9
221.9
Ph
m/z-->

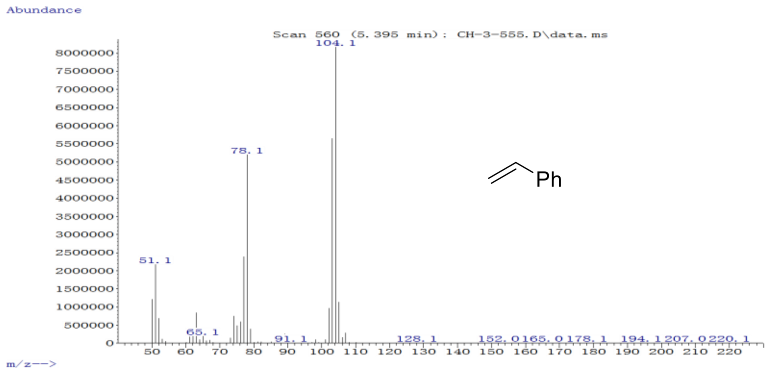
Abundance
Scan 560 (5.395 min): CH-3-555.D\data.ms
104.1
78.1
51.1
65.1
91.1
128.1
Ph
m/z-->

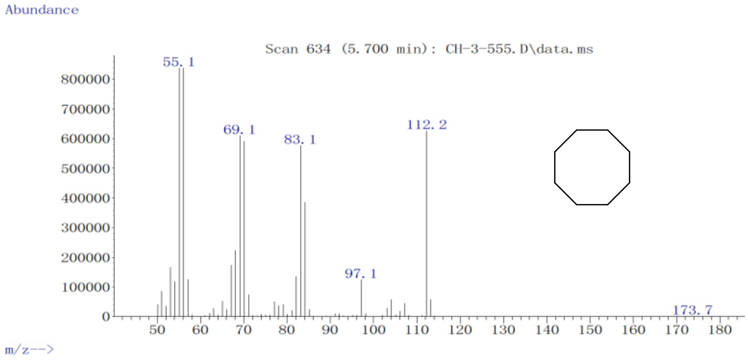
Abundance
Scan 634 (5.700 min): CH-3-555.D\data.ms
55.1
69.1
83.1
112.2
97.1
173.7
m/z-->

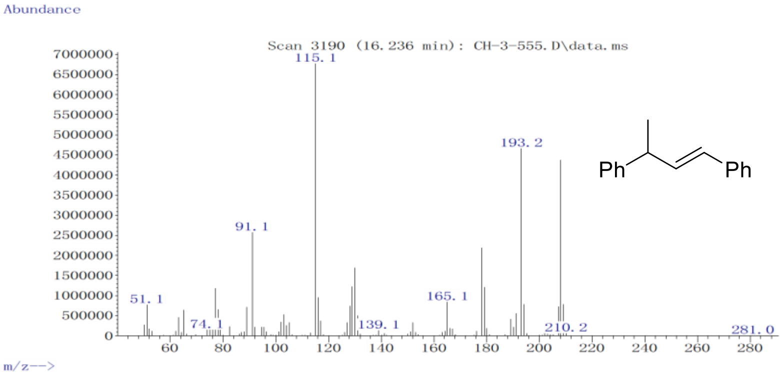
Abundance
Scan 3190 (16.236 min): CH-3-555.D\data.ms
115.1
193.2
91.1
51.1
74.1
139.1
165.1
210.2
281.0
Ph
Ph
m/z-->

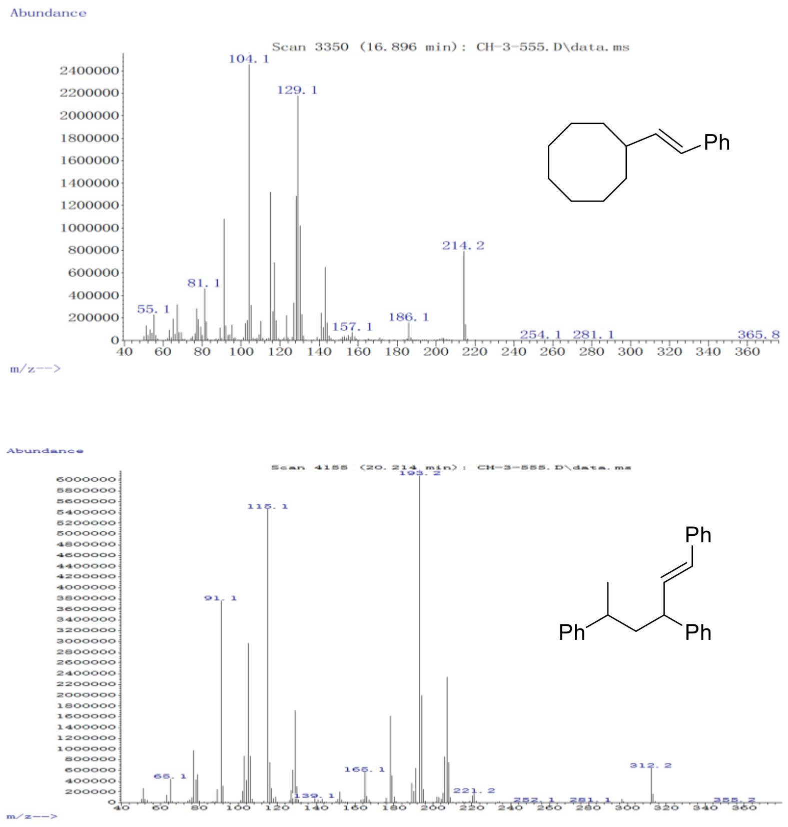

Supplementary Fig. 6 GCMS spectra of the crude reaction mixture from dehydrogenative alkenylation of cyclooctane with styrene following general procedure.

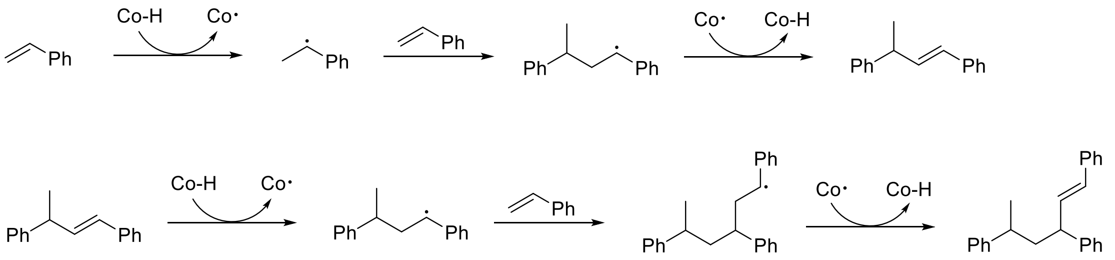

Supplementary Fig. 7 Cobalt hydride catalyzed oligomerization of styrene.

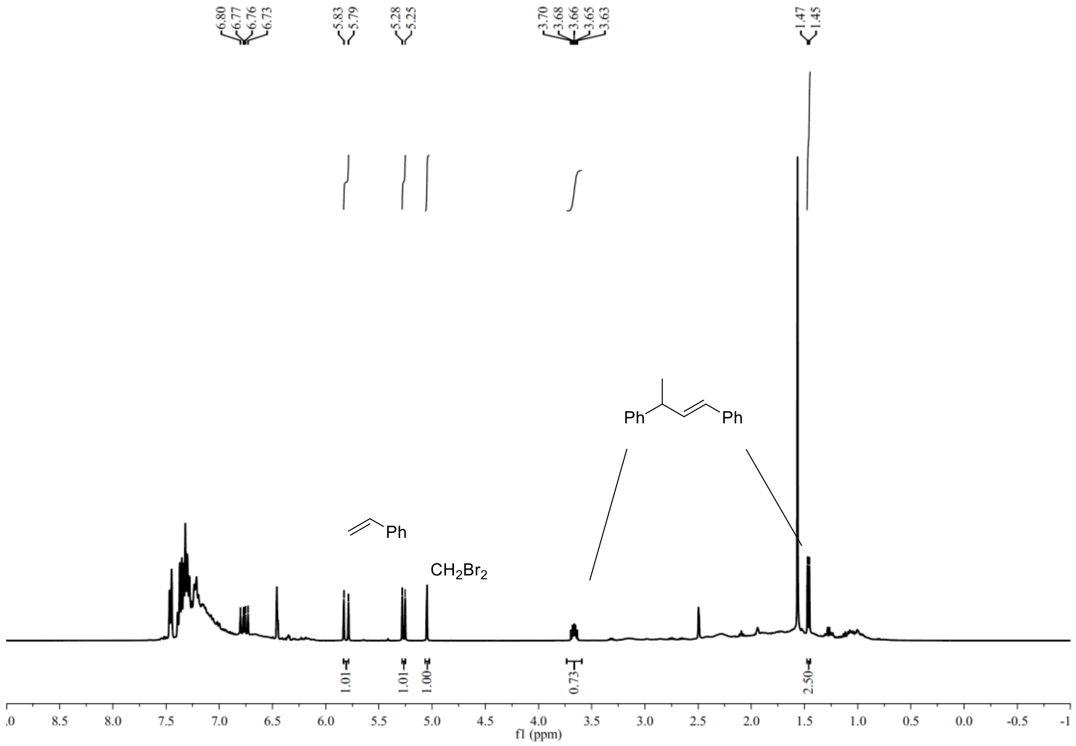

Supplementary Fig. 8 Crude ^{1}H NMR spectrum of dehydrogenative alkenylation of cyclooctane with styrene. Following general procedure in deuterated acetonitrile. 20 μL dibromomethane was added after 24 hour irradiation and reaction mixture was directly subjected to ^{1}H NMR analysis.

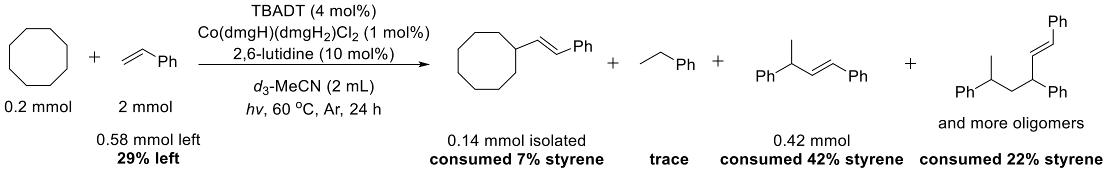

Supplementary Fig. 9 Quantitative analysis on the consumption of styrene based on GCMS and ^{1}H NMR spectra.

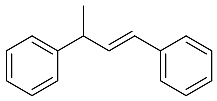

(*E*)-but-1-ene-1,3-diyldibenzene

Following the general procedure for dehydrogenative alkenylation (t = 24 h) with cyclooctane (22.4 mg, 0.2 mmol) and styrene (208.3 mg, 10 equiv), the major product from styrene oligomerization was obtained as a colorless liquid (eluent: hexane, R_f = 0.5). ^{1}H NMR (400 MHz, $CDCl_3$) δ 7.36 – 7.22 (m, 8H), 7.22 – 7.13 (m, 2H), 6.46 – 6.31 (m, 2H), 3.68 – 3.55 (m, 1H), 1.45 (d, J = 7.2 Hz, 3H). ^{13}C NMR (101 MHz, $CDCl_3$) δ 145.58, 137.53, 135.18, 128.50, 128.45, 127.27, 127.01, 126.18, 126.12, 42.53, 21.19. HRMS EI $[M]^+$ Calculated for $C_{16}H_{16}$ 208.1247, found 208.1245.

In conclusion, hydrogen gas was produced in 64% yield by GC analysis of the crude product mixture, which is very close to the yield of alkenylated product (Supplementary Figs. 3-5). Based on GCMS and ^{1}H NMR results (Supplementary Figs. 6-9), it is not likely that styrene or styrene derivative serves as hydrogen acceptor since hydrogenated byproducts from olefins were not detected. Cobalt hydride catalyzed oligomerization of styrene was found to be the competing side reaction and consumes most of the styrene. It is also the reason that excess amount of styrene is required to achieve high yields in the dehydrogenative alkenylation reactions.

Mechanistic investigations

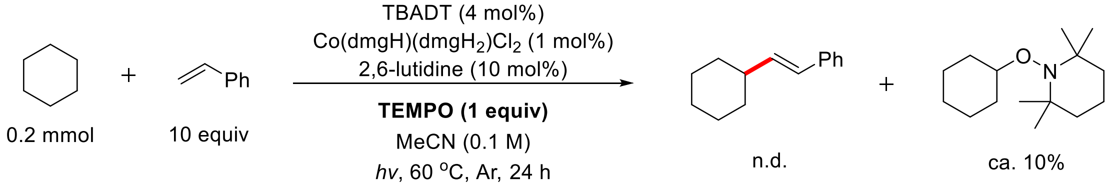

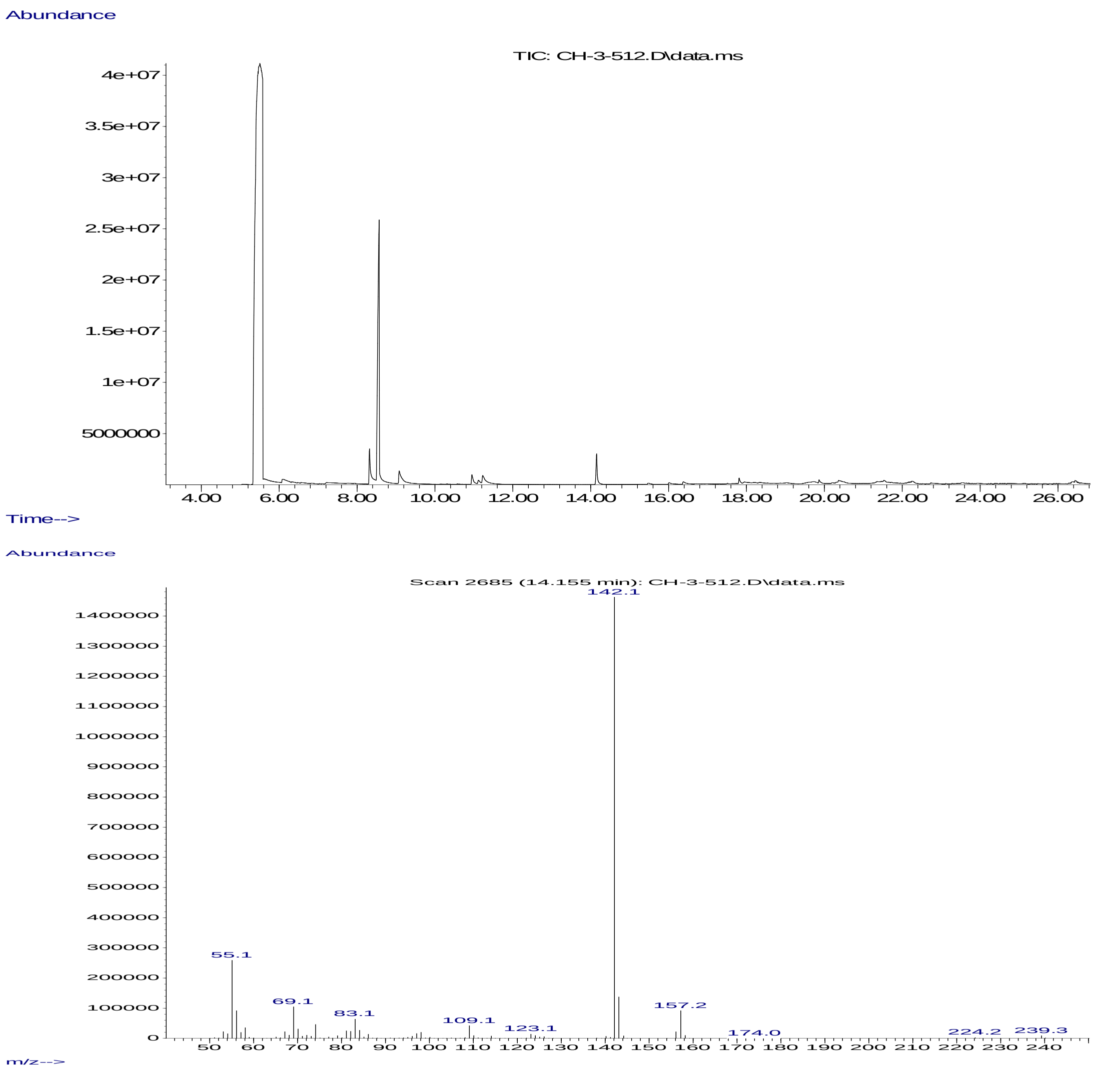

Supplementary Fig. 10 Radical trapping experiments and GCMS spectra of the crude reaction mixture. Calculated molecular weight of TEMPO-trapped cyclohexyl radical $C_{15}H_{29}NO$: 239.2, detected: 239.3 (retention time at 14.155 min).

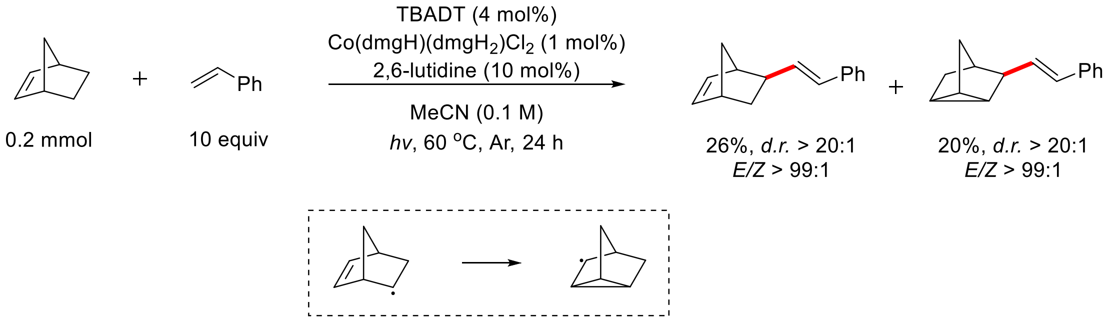

Supplementary Fig. 11 Dehydrogenative alkenylation of norbornene with styrene following the general procedure.

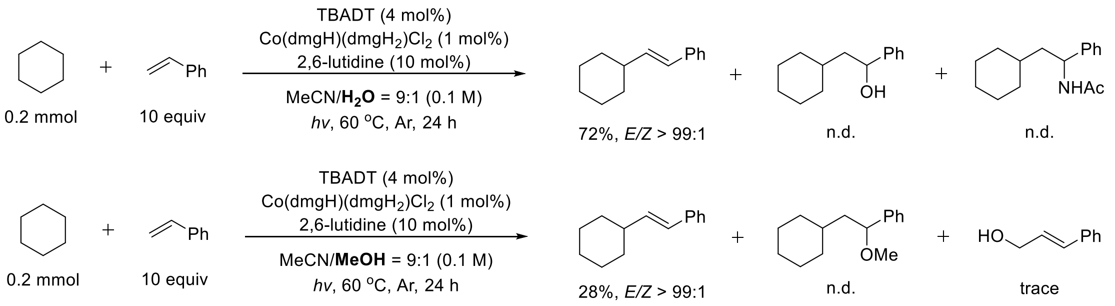

Supplementary Fig. 12 Benzylic cation abstraction experiments. To investigate the possibility of presence of a benzylic cation intermediate, water or MeOH was added to dehydrogenative alkenylation reaction of cyclohexane. However, no alcohol, amide or ether product was observed.

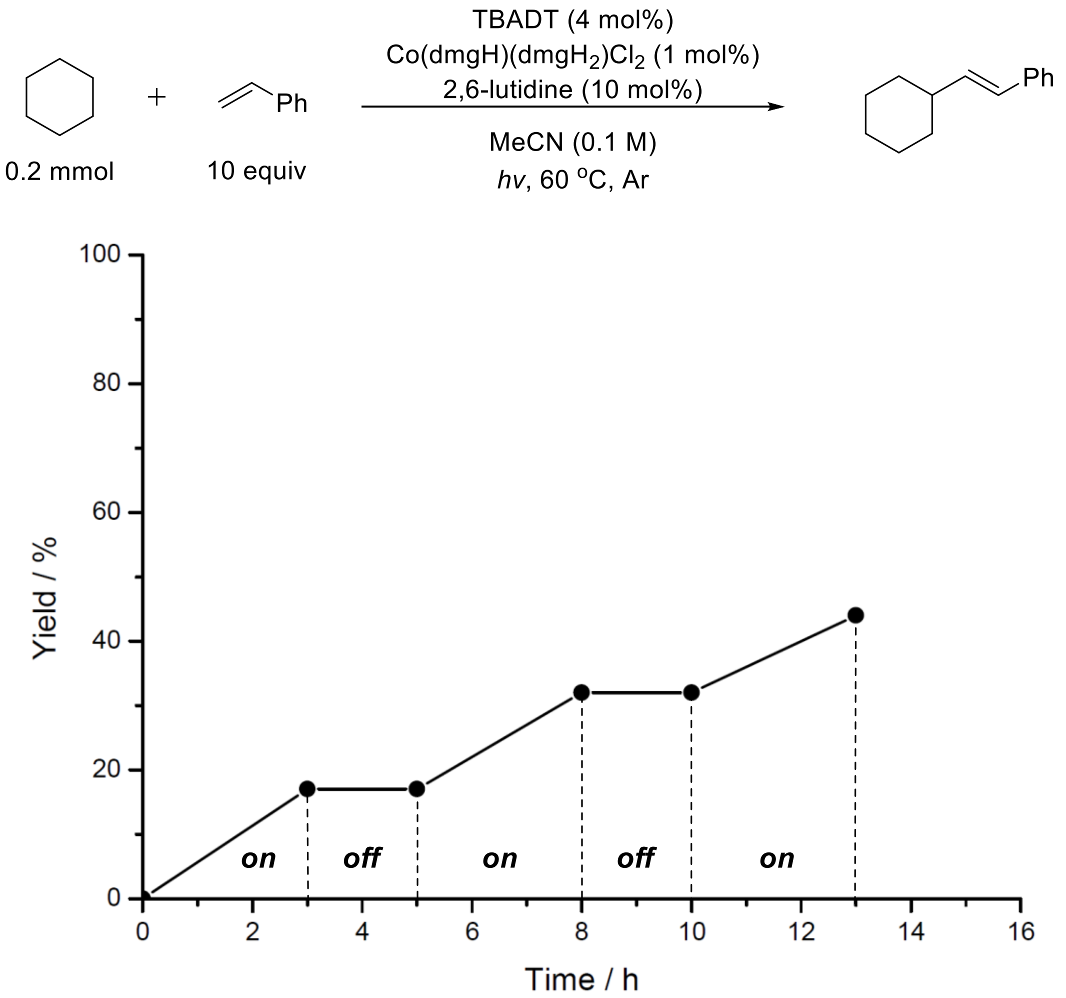

Supplementary Fig. 13 Light on-off experiments over time. To examine the impact of light, we conducted experiments under alternating periods of irradiation and darkness. These resulted in a

total interruption of the reaction progress in the absence of light and recuperation of reactivity on further illumination. The results demonstrated that light was a necessary component of the reaction. Even though we could not definitively rule out a radical-chain process, the data shown that any chain-propagation process must be short-lived.

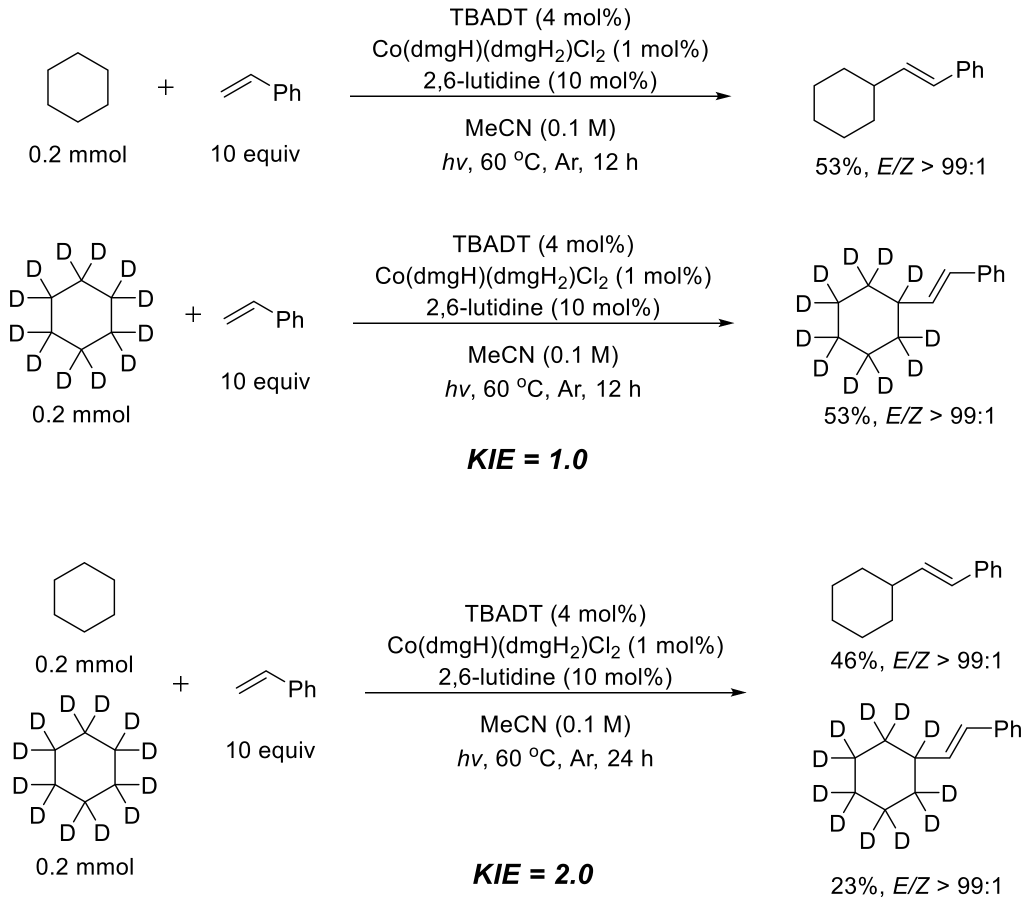

Supplementary Fig. 14 Measurement of KIE from two parallel reactions (top) and from intermolecular competition reaction (bottom).

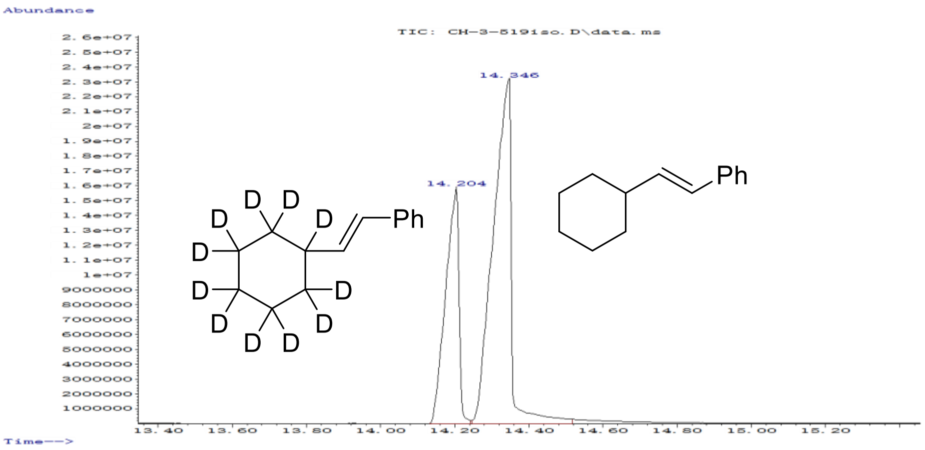

Signal : TIC: CH-3-519iso.D\data.ms

peak #	R.T. min	first scan	max scan	last scan	PK TY	peak height	corr. area	corr. % max.	% of total
1	14.204	2680	2697	2706	M	15926440	396945295	49.68%	33.192%
2	14.346	2707	2732	2773	M	24558659	798953595	100.00%	66.808%

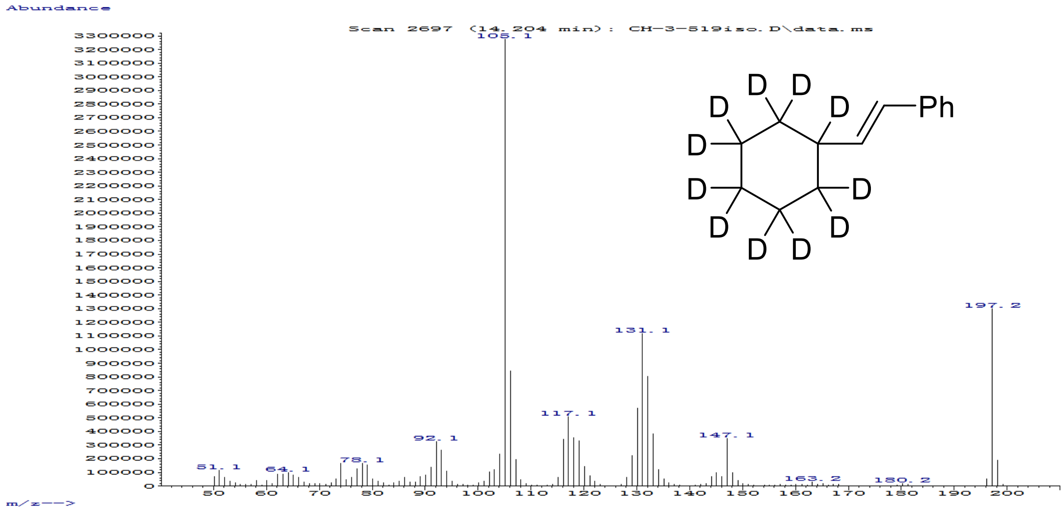

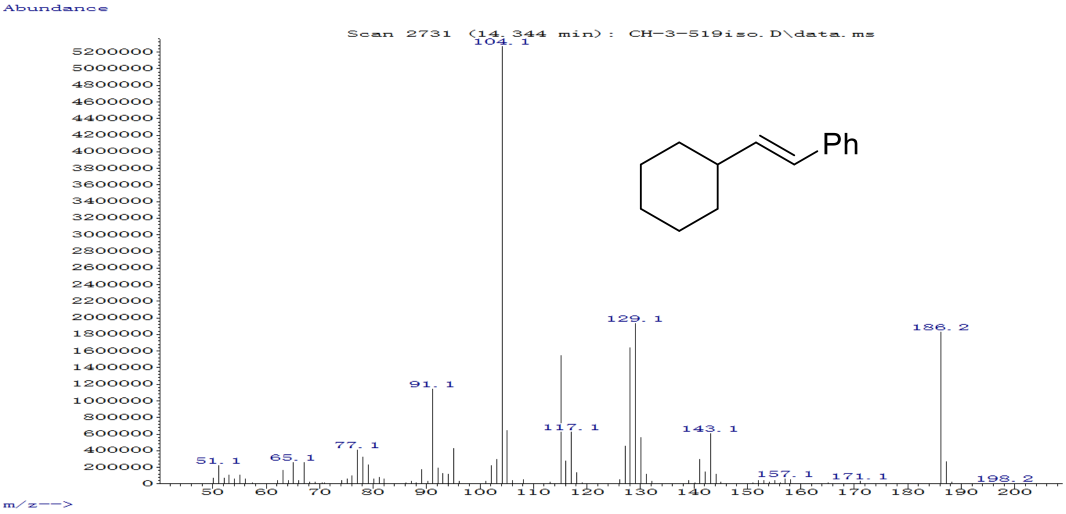

Supplementary Fig. 15 GCMS spectra of isolated products from intermolecular competition experiment.

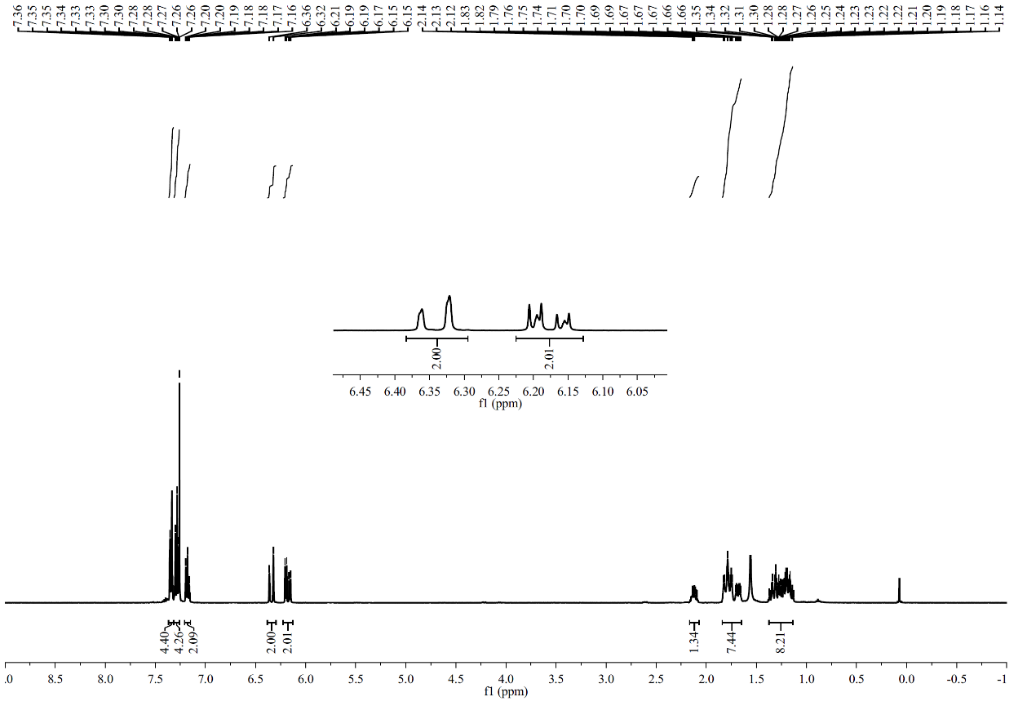

Supplementary Fig. 16 ^{1}H NMR spectrum of isolated products from intermolecular competition experiment.

We measured the KIE from two parallel reactions under standard conditions for 12 h using cyclohexane and d_{12}-cyclohexane as the substrate separately (Supplementary Fig. 14, top). We managed to obtain non-deuterated and deuterated products both in 53% yield. The KIE value was calculated to be 1.0. Next, we measured the KIE from intermolecular competition experiment by adding cyclohexane/d_{12}-cyclohexane (1:1) into the same reaction mixture (Supplementary Fig. 14, bottom), the KIE value was calculated to be 2.0 based on GCMS and NMR analysis (Supplementary Figs. 15-16). These results indicated that C-H cleavage is not the rate-determining step, and different C-H cleavage rates between cyclohexane and d_{12}-cyclohexane determined the product distribution in intermolecular competition experiment.

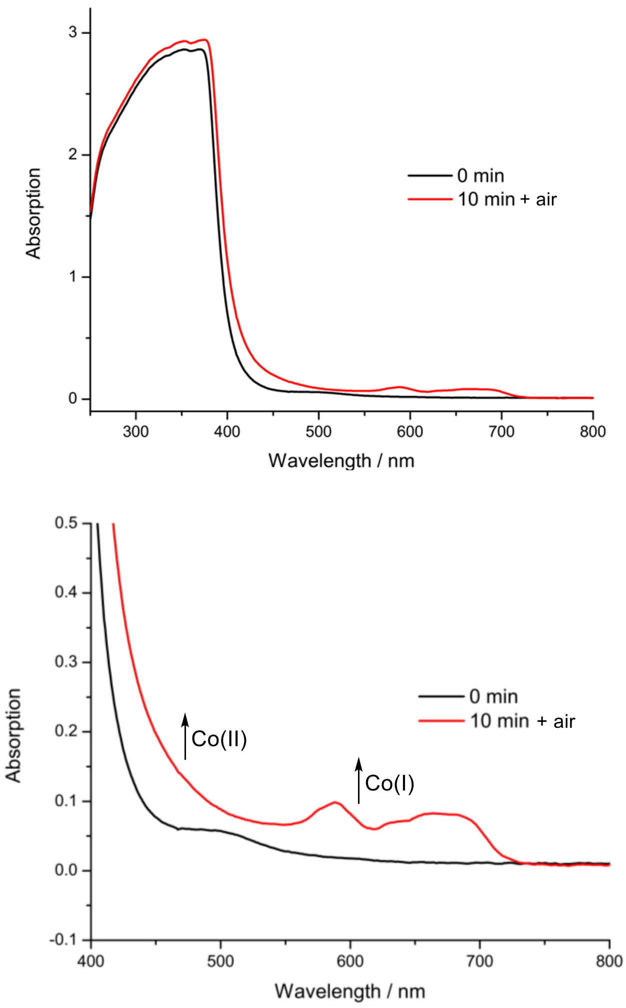

Supplementary Fig. 17 UV-Vis spectra of the standard reaction mixture before irradiation (black like) and after 10 min irradiation and exposure to air for 5 min (red line).

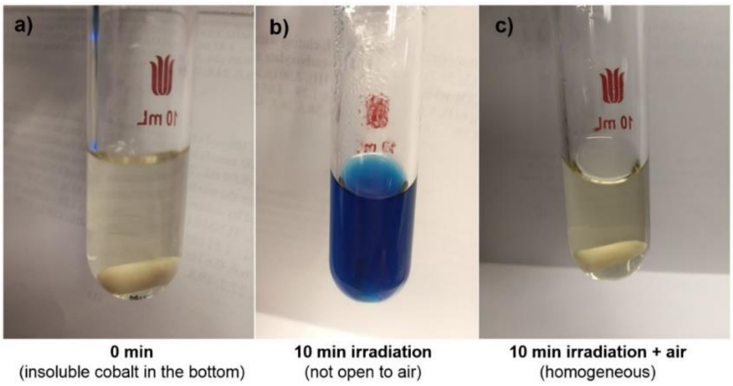

Supplementary Fig. 18 Images of reaction mixture. a) Before irradiation (light yellow). b) After 10 min irradiation without exposure to air (deep blue). c) After 10 min irradiation with exposure to air (light yellow).

A UV-Vis monitoring study of the reaction mixture revealed that two absorption bands at 440-500 nm and 550-700 nm appeared after 10 min light irradiation and exposure to air (Supplementary Fig. 17), which was in agreement with the formation of Co^{II} and Co^{I} intermediates, respectively.[13,14] It is well known that derivatives from $[W_{10}O_{32}]^{4-}$ photocatalyst, such as $H^+[W_{10}O_{32}]^{5-}$, $[W_{10}O_{32}]^{5-}$ and $[W_{10}O_{32}]^{6-}$, are deep blue with strong absorption bands in the 600-800 nm region.[15] However, these absorption bands would disappear quickly after exposure to air (Supplementary Fig. 18). Quick disappearance of these bands in air was also observed by the Sorenson group.[16] Based on our finding and literature reports, the absorption bands at 440-500 nm and 550-700 nm were attributed to Co^{II} and Co^{I} intermediates respectively.

Supplementary Table 12 Dehydrogenative alkenylation of adamantane with different cobalt catalysts.

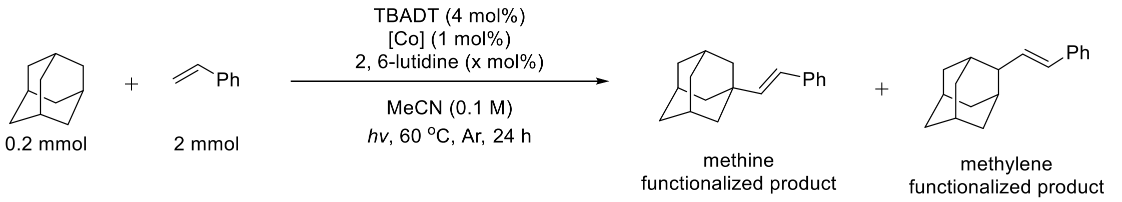

entry	[Co]	x	combined yield (%)[a]	methine/methylene product[b]	Methine/methylene C-H selectivity[c]
1	$Co(dmgH)(dmgH_2)Cl_2$	0	48	1 : 1.47	2.0 : 1
2	$Co(dmgH)(dmgH_2)Cl_2$	1	68	1 : 1.42	2.1 : 1
3	$Co(dmgH)(dmgH_2)Cl_2$	10	82	1 : 1.29	2.3 : 1
4	$Co(dpgH)(dpgH_2)Cl_2$	10	69%	1 : 0.85	3.5 : 1
5	$Co(gH)(gH_2)Cl_2$	10	< 10%	1 : 0.67	4.5 : 1

[a]Isolated yields. *E*/*Z* Ratios were determined by GC analysis of the crude mixtures and were >99:1 in all cases. [b]Product ratios were determined from ^1H NMR of isolated product and GCMS data of crude reaction mixture. [c]C-H selectivity was determined after correction by the number of hydrogen atoms.

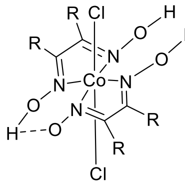

R=H, $Co(gH)(gH)_2Cl_2$
R=Me, $Co(dmgH)(dmgH_2)Cl_2$
R=Ph, $Co(dpgH)(dpgH)_2Cl_2$

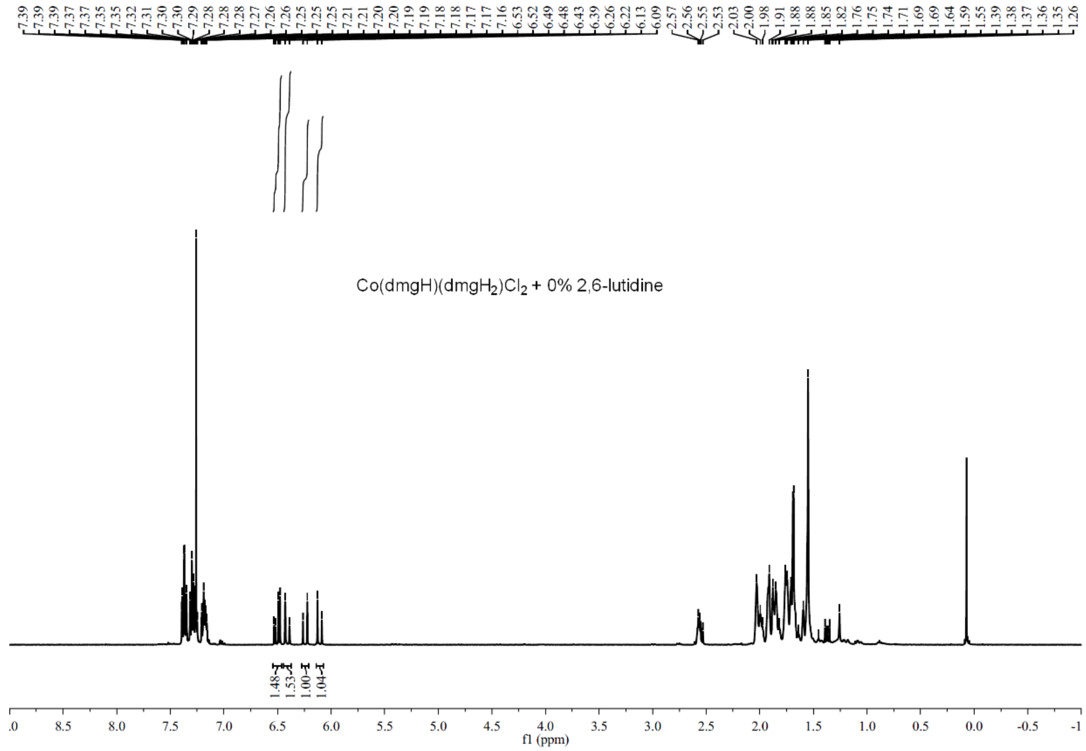

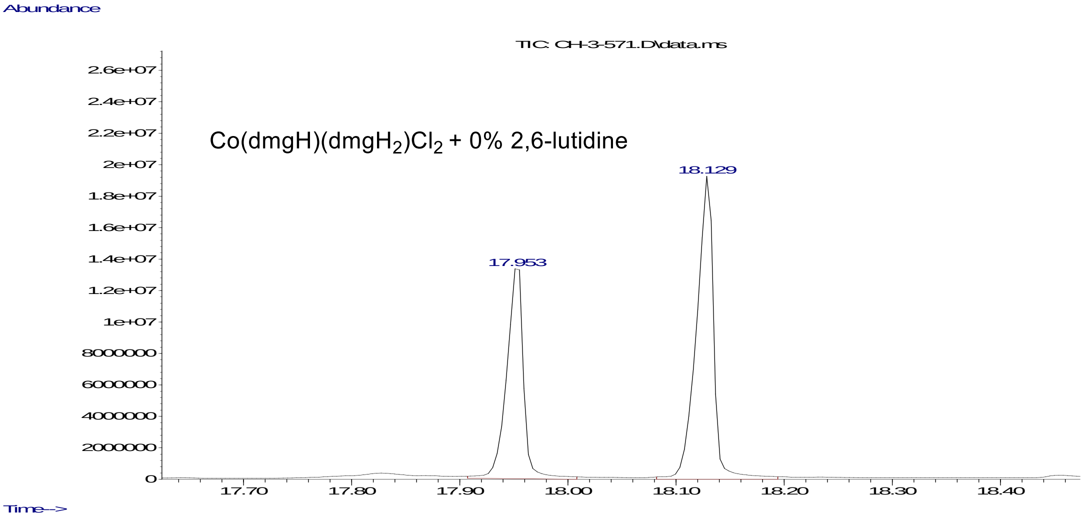

Signal : TIC: CH-3-571.D\data.ms

peak #	R.T. min	first scan	max scan	last scan	PK TY	peak height	corr. area	corr. % max.	% of total
1	17.953	3595	3606	3620	M	13762337	146845215	68.25%	40.565%
2	18.129	3638	3649	3665	M	19292074	215151211	100.00%	59.435%

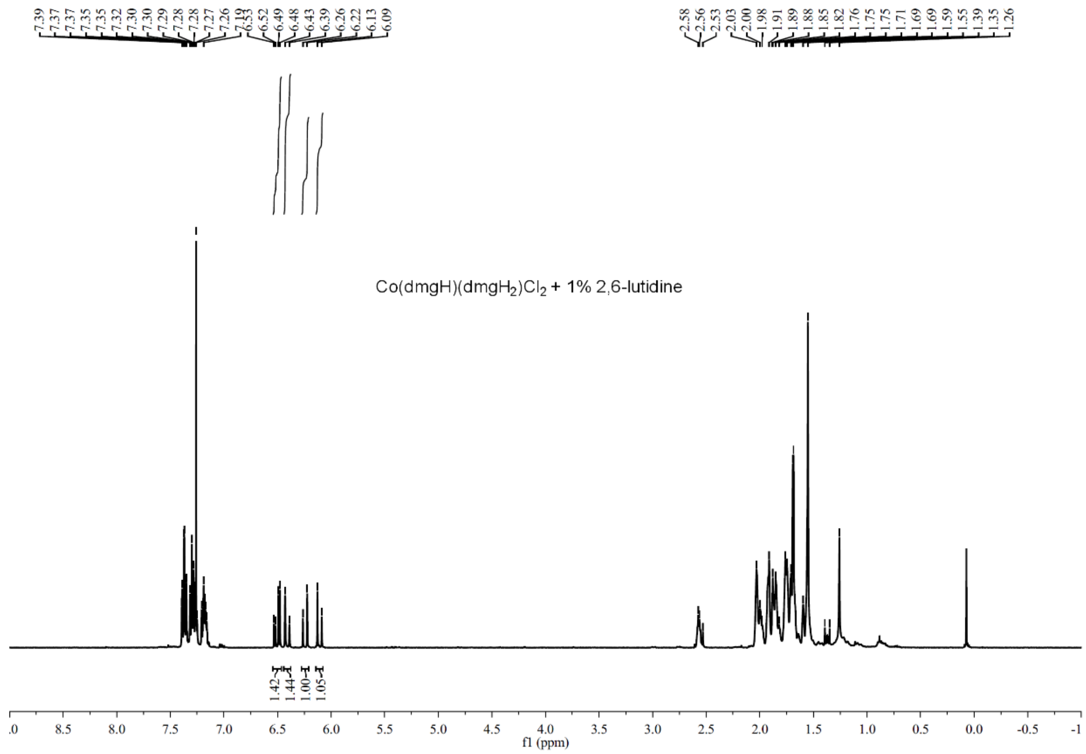

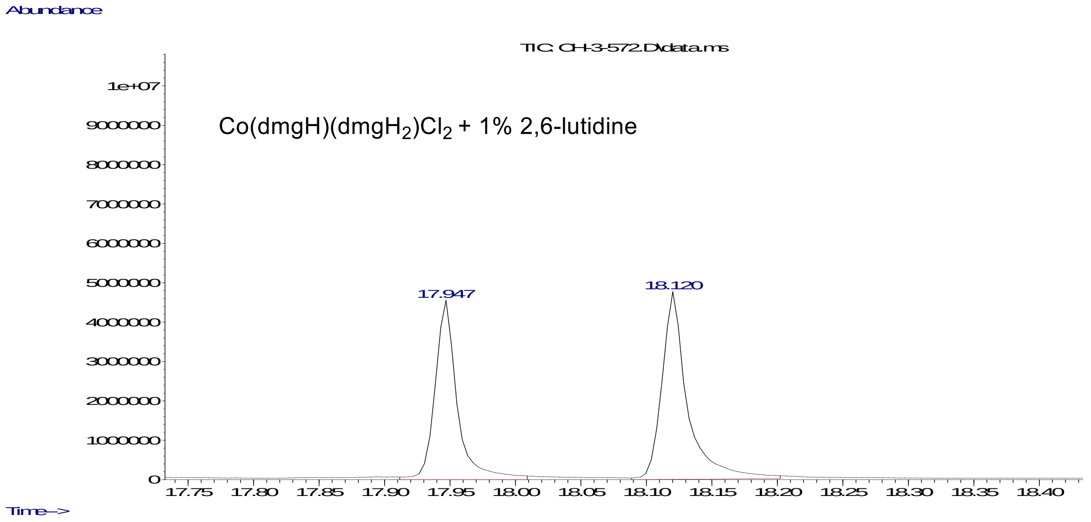

Signal : TIC: CH-3-572.D\data.ms

peak #	R.T. min	first scan	max scan	last scan	PK TY	peak height	corr. area	corr. % max.	% of total
1	17.947	3596	3605	3620	M	4571331	54042325	83.45%	45.490%
2	18.120	3639	3647	3667	M	4768269	64758271	100.00%	54.510%

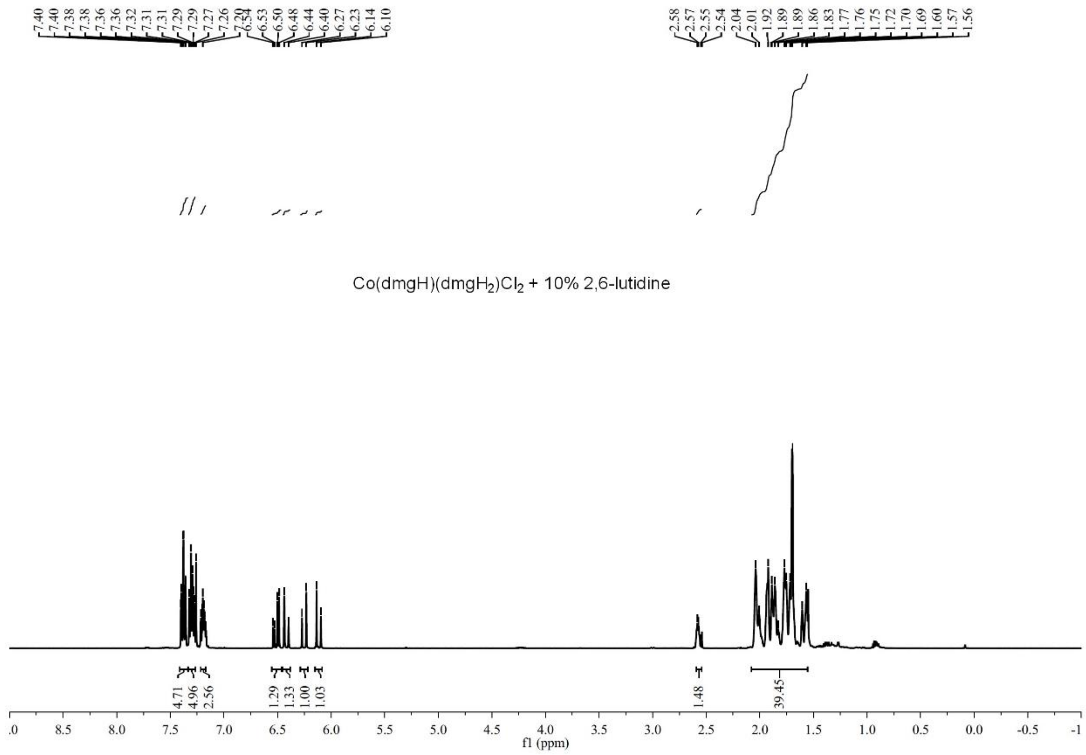

Co(dmgH)(dmgH$_2$)Cl$_2$ + 10% 2,6-lutidine

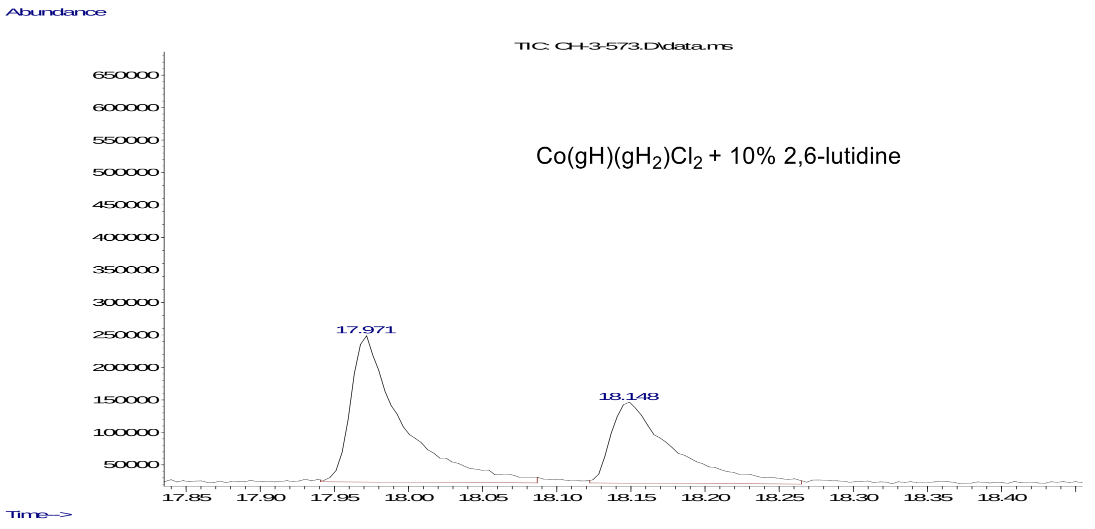

Signal : TIC: CH-3-573.D\data.ms

peak #	R.T. min	first scan	max scan	last scan	PK TY	peak height	corr. area	corr. % max.	% of total
1	17.971	3603	3611	3639	M2	226130	5442417	100.00%	59.998%
2	18.148	3648	3654	3682	M3	125003	3628506	66.67%	40.002%

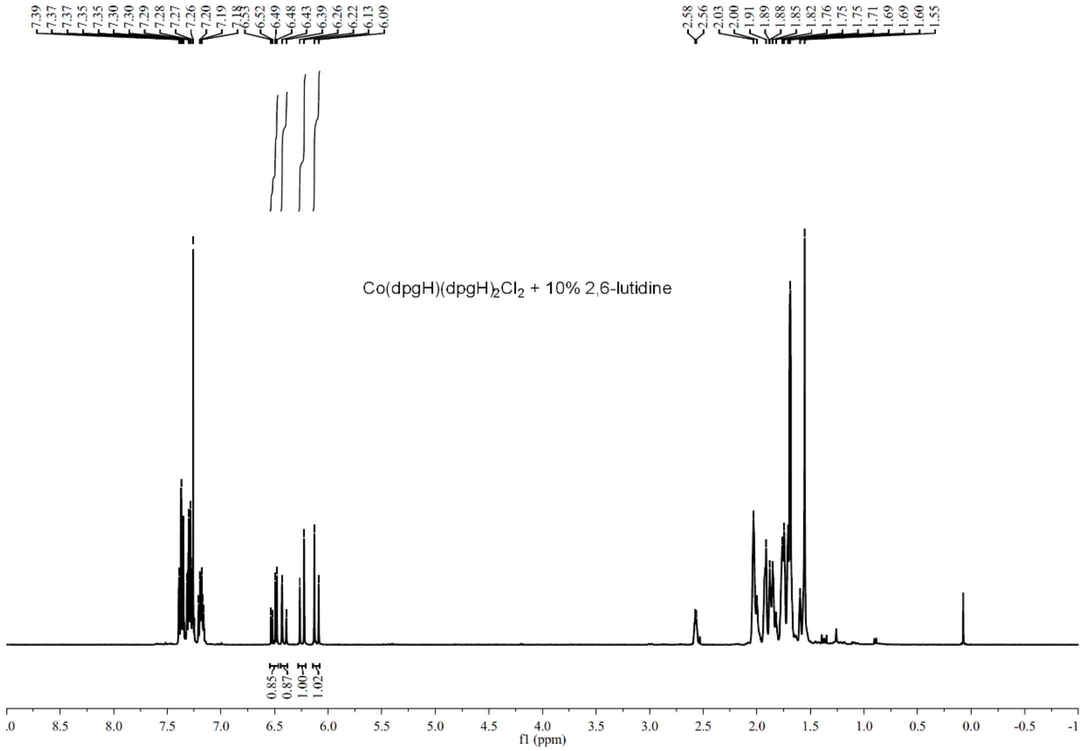

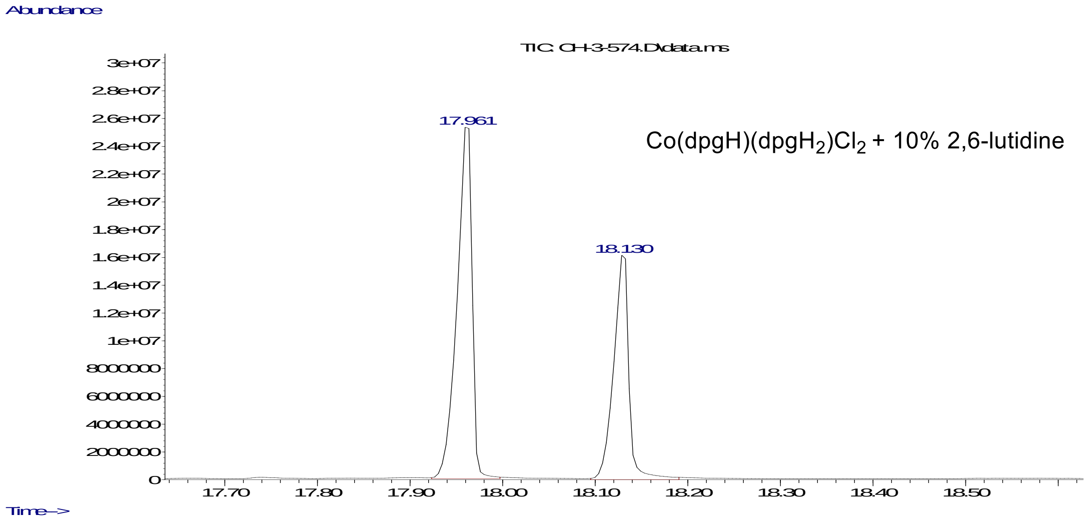

Signal : TIC: CH-3-574.D\data.ms

peak #	R.T. min	first scan	max scan	last scan	PK TY	peak height	corr. area	corr. % max.	% of total
1	17.961	3599	3608	3617	M	26022759	287776851	100.00%	60.684%
2	18.130	3641	3649	3664	M	16556770	186447831	64.79%	39.316%

Supplementary Fig. 19 ^{1}H NMR spectra of isolated products and GCMS spectra of crude reaction mixture to study the effect of cobalt catalyst on reaction selectivity.

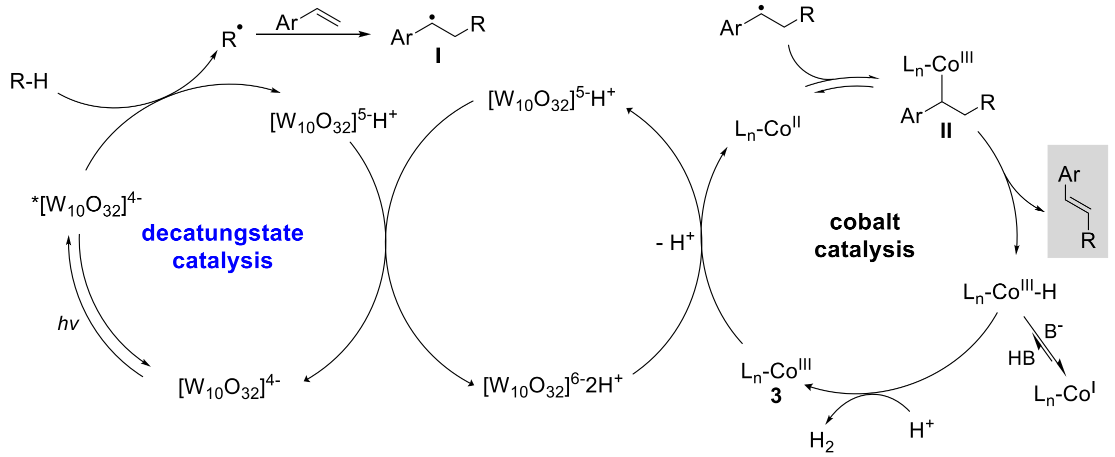

Supplementary Fig. 20 Alternative reaction mechanism where Co catalyst is reduced by $[W_{10}O_{32}]^{6-}2H^{+}$.

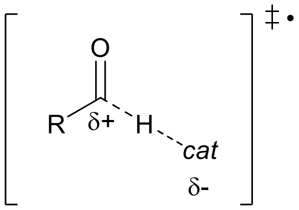

Supplementary Fig. 21 Polarity-matched transition state for the abstraction of formyl C-H by excited decatungstate anion.

Calculation of bond dissociation energy

Theoretical calculations are performed using the Gaussian 16 Rev. A.03 software suite.[17] Bond dissociation enthalpies (BDE) was calculated by the formation enthalpy difference between the sum of two individual fragments formed after the homolysis of the involved bond and the corresponding species before the bond dissociation.[18] The geometries optimization and enthalpy calculations in this study were all performed at the (U)M062X/6-31G(d) level of theory, taking into account the solvent effect of acetonitrile using SMD solvent model. All frequency calculations gave no imaginary frequencies.

Supplementary Table 13 Calculated C-H bond strengths of *trans*-1,4-dimethylcyclohexane, 1-methylcyclopentanol and hexamethyldisilane in kcal/mol.

Bond	BDEs
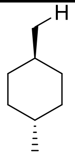	102.8
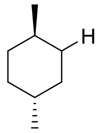	99.8
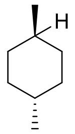	96.9
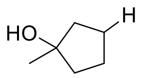	96.6
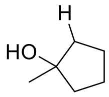	97.5
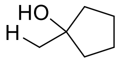	102.9
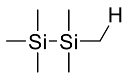	101.3

Supplementary Note 1

Analytical data of isolated compounds

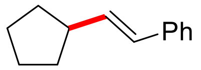

(*E*)-(2-cyclopentylvinyl)benzene, 12

Following the general procedure for dehydrogenative alkenylation (t = 24 h) with cyclopentane (14.0 mg, 0.2 mmol) and styrene (208.3 mg, 10 equiv), obtained in 54% yield as a colorless liquid (18.7 mg, eluent: hexane, R_f = 0.7). ^{1}H NMR (400 MHz, $CDCl_3$) δ 7.37 – 7.32 (m, 2H), 7.31 – 7.26 (m, 2H), 7.22 – 7.14 (m, 1H), 6.37 (d, J = 16.0 Hz, 1H), 6.21 (dd, J = 16.0, 7.8 Hz, 1H), 2.68 – 2.53 (m, 1H), 1.93 – 1.81 (m, 2H), 1.78 – 1.55 (m, 4H), 1.47 – 1.34 (m, 2H). ^{13}C NMR (101 MHz, $CDCl_3$) δ 137.96, 135.71, 128.44, 127.84, 126.69, 125.91, 43.79, 33.21, 25.23. HRMS EI $[M]^+$ Calculated for $C_{13}H_{16}$ 172.1252, found 172.1252.

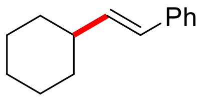

(*E*)-(2-cyclohexylvinyl)benzene, 13

Following the general procedure for dehydrogenative alkenylation (t = 24 h) with cyclohexane (16.8 mg, 0.2 mmol) and styrene (208.3 mg, 10 equiv), obtained in 78% yield as a colorless liquid (29.0 mg, eluent: hexane, R_f = 0.7). ^{1}H NMR (400 MHz, $CDCl_3$) δ 7.38 – 7.33 (m, 2H), 7.32 – 7.26 (m, 2H), 7.23 – 7.14 (m, 1H), 6.35 (d, J = 16.0 Hz, 1H), 6.19 (dd, J = 16.0, 6.8 Hz, 1H), 2.21 – 2.07 (m, 1H), 1.86 – 1.73 (m, 4H), 1.74 – 1.65 (m, 1H), 1.38 – 1.16 (m, 5H). ^{13}C NMR (101 MHz, $CDCl_3$) δ 138.07, 136.84, 128.43, 127.22, 126.70, 125.93, 41.14, 32.96, 26.18, 26.04. HRMS EI $[M]^+$ Calculated for $C_{14}H_{18}$ 186.1409, found 186.1408.

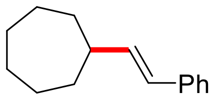

(*E*)-styrylcycloheptane, 14

Following the general procedure for dehydrogenative alkenylation (t = 24 h) with cycloheptane (19.6 mg, 0.2 mmol) and styrene (208.3 mg, 10 equiv), obtained in 62% yield as a colorless liquid (24.9 mg, eluent: hexane, R_f = 0.7). ^{1}H NMR (500 MHz, $CDCl_3$) δ 7.36 – 7.32 (m, 2H), 7.31 – 7.26 (m, 2H), 7.20 – 7.15 (m, 1H), 6.32 (d, J = 16.0 Hz, 1H), 6.22 (dd, J = 16.0, 7.5 Hz, 1H), 2.39 – 2.28 (m, 1H), 1.87 – 1.79 (m, 2H), 1.75 – 1.67 (m, 2H), 1.67 – 1.60 (m, 2H), 1.57 – 1.41 (m, 6H). ^{13}C NMR (126 MHz, $CDCl_3$) δ 138.13, 137.69, 128.43, 126.64, 125.92, 43.22, 34.72, 28.40, 26.27. HRMS EI $[M]^+$ Calculated for $C_{15}H_{20}$ 200.1560, found 200.1562.

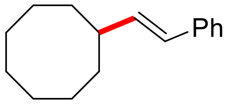

(*E*)-styrylcyclooctane, 11

Following the general procedure for dehydrogenative alkenylation (t = 24 h) with cyclooctane (22.4 mg, 0.2 mmol) and styrene (208.3 mg, 10 equiv), obtained in 69% yield as a colorless liquid (29.7 mg, eluent: hexane, R_f = 0.7). ^{1}H NMR (400 MHz, $CDCl_3$) δ 7.36 – 7.32 (m, 2H), 7.31 – 7.26 (m, 2H), 7.20 – 7.16 (m, 1H), 6.32 (d, J = 16.0 Hz, 1H), 6.21 (dd, J = 16.0, 7.2 Hz, 1H), 2.45-2.33 (m, 1H), 1.78 – 1.49 (m, 14H). ^{13}C NMR (101 MHz, $CDCl_3$) δ 138.14, 137.82, 128.43, 126.82, 126.63, 125.92, 41.31, 31.86, 27.43, 26.00, 25.08. HRMS EI $[M]^+$ Calculated for $C_{16}H_{22}$ 214.1716, found 214.1716.

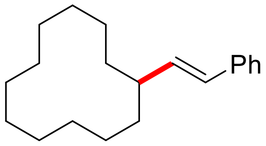

(*E*)-styrylcyclododecane, 15

Following the general procedure for dehydrogenative alkenylation (t = 24 h) with cyclododecane (33.7 mg, 0.2 mmol) and styrene (208.3 mg, 10 equiv), obtained in 61% yield as a colorless oil (33.2 mg, eluent: hexane, R_f = 0.7). ^{1}H NMR (500 MHz, $CDCl_3$) δ 7.38 – 7.33 (m, 2H), 7.31 – 7.27 (m, 2H), 7.20 – 7.16 (m, 1H), 6.34 (d, J = 16.0 Hz, 1H), 6.11 (dd, J = 16.0, 8.0 Hz, 1H), 2.39 – 2.29 (m, 1H), 1.60 – 1.29 (m, 22H). ^{13}C NMR (126 MHz, $CDCl_3$) δ 138.04, 136.60, 128.43, 128.20, 126.67,

125.92, 37.60, 29.99, 23.83, 23.76, 23.42, 22.32. HRMS EI $[M]^+$ Calculated for $C_{20}H_{30}$ 270.2342, found 270.2345.

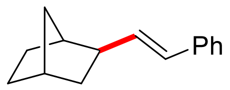

2-((*E*)-styryl)bicyclo[2.2.1]heptane, 16

Following the general procedure for dehydrogenative alkenylation (t = 24 h) with norbornane (19.2 mg, 0.2 mmol) and styrene (208.3 mg, 10 equiv), obtained in 70% yield as a colorless liquid (27.7 mg, eluent: hexane, R_f = 0.6). ^1H NMR (400 MHz, $CDCl_3$) δ 7.36 – 7.31 (m, 2H), 7.31 – 7.26 (m, 2H), 7.21 – 7.15 (m, 1H), 6.30 (d, J = 16.0 Hz, 1H), 6.13 (dd, J = 16.0, 8.0 Hz, 1H), 2.32 – 2.22 (m, 2H), 2.17 – 2.12 (m, 1H), 1.57 – 1.37 (m, 5H), 1.30 – 1.16 (m, 3H). ^{13}C NMR (126 MHz, $CDCl_3$) δ 137.99, 136.48, 128.43, 127.23, 126.67, 125.91, 45.41, 42.69, 37.90, 36.64, 35.82, 29.76, 29.02. HRMS EI $[M]^+$ Calculated for $C_{15}H_{18}$ 198.1403, found 198.1405. Spectral data is in agreement with *exo* structure in previous reports.[19,20]

5.0 mmol scale procedure:

To a 250 mL oven-dried Schlenk bottle equipped with a magnetic stir bar was added norbornane (480.9 mg, 5.0 mmol), styrene (5.208 g, 10 equiv), TBADT (664.0 mg, 0.2 mmol, 4 mol%), Co(dmgH)($dmgH_2$)Cl_2 (18.1 mg, 0.05 mmol, 1 mol%), 2,6-lutidine (53.6 mg, 0.5 mmol, 10 mol%) and dry acetonitrile (50 mL). The resulting mixture was sealed and then subjected to freeze-pump-thaw for three times. After that, the reaction was placed under a 370 nm LED (2.5 meter strips, 24 W), stirred and irradiated for 24 h. The temperature was maintained at 60 °C using a water bath. The reaction mixture was removed from light and quenched by stirring open to air for 5 minutes. The solvent was removed on a rotary evaporator under reduced pressure and the residue was subjected to column chromatography isolation on silica gel to give the corresponding product as a colorless liquid (704 mg, 71% yield, eluent: hexane, R_f = 0.6).

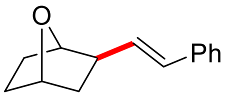

2-((*E*)-styryl)-7-oxabicyclo[2.2.1]heptane, 17

Following the general procedure for dehydrogenative alkenylation (t = 24 h) with 7-oxabicyclo[2.2.1]heptane (19.6 mg, 0.2 mmol) and styrene (208.3 mg, 10 equiv), obtained in 64% yield as a colorless liquid (25.5 mg, eluent: hexane : ethyl acetate = 50/1, R_f = 0.1). ^{1}H NMR (400 MHz, $CDCl_3$) δ 7.37 – 7.32 (m, 2H), 7.31 – 7.26 (m, 2H), 7.21 – 7.19 (m, 1H), 6.34 (d, J = 16.0 Hz, 1H), 6.17 (dd, J = 16.0, 9.2 Hz, 1H), 4.70 – 4.61 (m, 1H), 4.41 – 4.34 (m, 1H), 2.61 – 2.47 (m, 1H), 1.88 – 1.80 (m, 2H), 1.77 – 1.72 (m, 2H), 1.61 – 1.57 (m, 2H). ^{13}C NMR (126 MHz, $CDCl_3$) δ 137.48, 134.37, 128.47, 128.41, 126.97, 126.04, 81.45, 76.59, 47.15, 38.90, 29.82, 29.69. HRMS EI $[M]^+$ Calculated for $C_{14}H_{16}O$ 200.1196, found 200.1196.

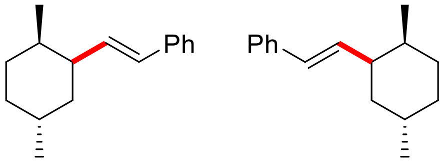

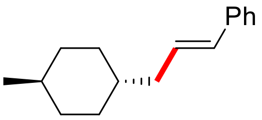

major minor

((*E*)-2-((2*R*,5*R*)-2,5-dimethylcyclohexyl)vinyl)benzene and **((*E*)-2-((2*S*,5*S*)-2,5-dimethylcyclohexyl)vinyl)benzene (18, major)**

((*E*)-3-((1*r*,4*r*)-4-methylcyclohexyl)prop-1-en-1-yl)benzene (18, minor)

Following the general procedure for dehydrogenative alkenylation (t = 24 h) with *trans*-1,4-dimethylcyclohexane (22.4 mg, 0.2 mmol) and styrene (208.3 mg, 10 equiv), obtained in 67% yield as a colorless liquid (28.6 mg, containing all isomers, major : minor = 12.2 : 1, diastereoselectivity of major product = 3 : 1, eluent: hexane, R_f = 0.7). Attempt to identify the two diastereomers in major product by 2D NMR is not successful. ^{1}H NMR (400 MHz, $CDCl_3$) δ 7.39 – 7.32 (m, 2H), 7.31 – 7.26 (m, 2H), 7.21 – 7.16 (m, 1H), δ 6.34 (d, J = 16.0 Hz, 1H, major 1), 6.33 (d, J = 16.4 Hz, 1H, major 2), 6.31 (d, J = 16.0 Hz, 1H, minor), 6.22 (dt, J = 16.0, 7.2 Hz, 1H, minor), 6.20 (dd, J = 16.4, 8.4 Hz, 1H, major 2), 6.04 (dd, J = 16.0, 8.8 Hz, 1H, major 1), 2.10 (td, J = 7.2, 1.2 Hz, 2H, minor), 1.82 – 0.84 (m, 15H, major 1), 1.82 – 0.84 (m, 15H, major 2), 1.82 – 0.84 (m, 14H, minor).

^{13}C NMR (126 MHz, $CDCl_3$) δ 138.03, 135.96, 128.91, 128.43, 126.67, 125.92, 49.21, 42.28, 36.76, 35.18, 32.35, 29.69, 22.60, 20.66. HRMS EI $[M]^+$ Calculated for $C_{16}H_{22}$ 214.1716, found 214.1715.

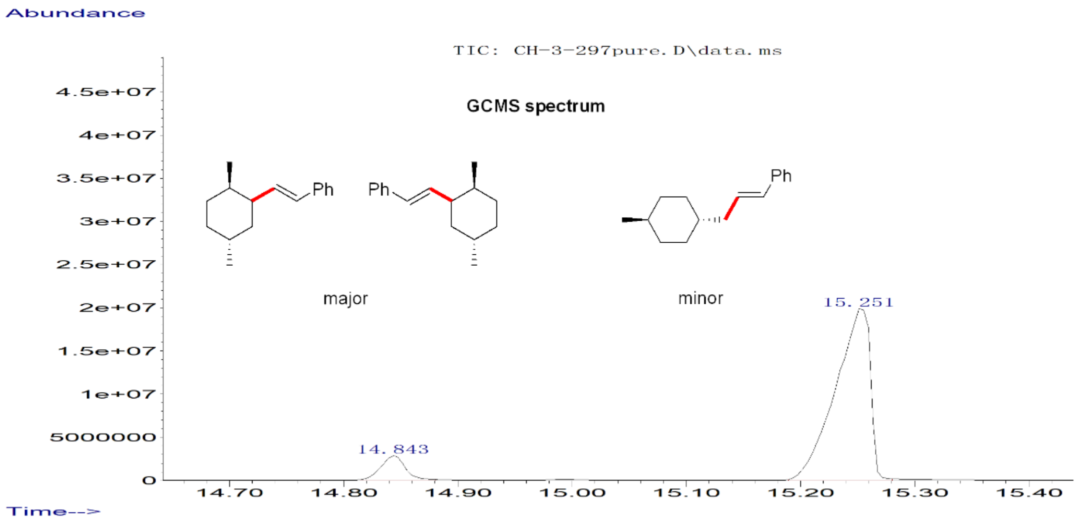

Signal : TIC: CH-3-297pure.D\data.ms

peak #	R.T. min	first scan	max scan	last scan	PK TY	peak height	corr. area	corr. % max.	% of total
1	14.843	2845	2852	2861	rM	2866674	4355647	10.31%	9.343%
2	15.251	2936	2951	2958	rM	19893580	42261805	100.00%	90.657%

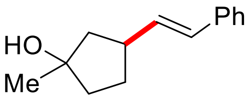

1-methyl-3-((*E*)-styryl)cyclopentan-1-ol, 19

Following the general procedure for dehydrogenative alkenylation (t = 24 h) with 1-methylcyclopentan-1-ol (20.0 mg, 0.2 mmol) and styrene (208.3 mg, 10 equiv), obtained in 66% yield as a colorless liquid (26.7 mg, *d. r.* = 1.6 : 1, eluent: hexane/ethyl acetate = 10:1, R_f = 0.15). ^{1}H NMR (400 MHz, $CDCl_3$) δ 7.36 – 7.33 (m, 2H), 7.31 – 7.27 (m, 2H), 7.21 – 7.17 (m, 1H), 6.39 (d, J = 16.0, 1H, major), 6.36 (d, J = 16.0, 1H, minor), 6.27 (dd, J = 16.0, 7.6 Hz, 1H, minor), 6.18 (dd, J = 16.0, 7.6 Hz, 1H, major), 3.12 – 2.99 (m, 1H, major), 2.83 – 2.70 (m, 1H, minor), 2.08 – 1.66 (m, 6H), 1.41 (s, 3H, major), 1.39 (s, 3H, minor). ^{13}C NMR (126 MHz, $CDCl_3$) δ 137.66, 137.62, 135.42, 134.78, 128.44, 128.23, 128.07, 126.82, 125.95, 125.92, 80.00, 79.76, 48.65, 48.26, 42.64, 42.01, 41.78, 40.89, 32.03, 31.64, 29.10, 28.78. HRMS EI $[M]^+$ Calculated for $C_{14}H_{18}O$

202.1352, found 202.1353.

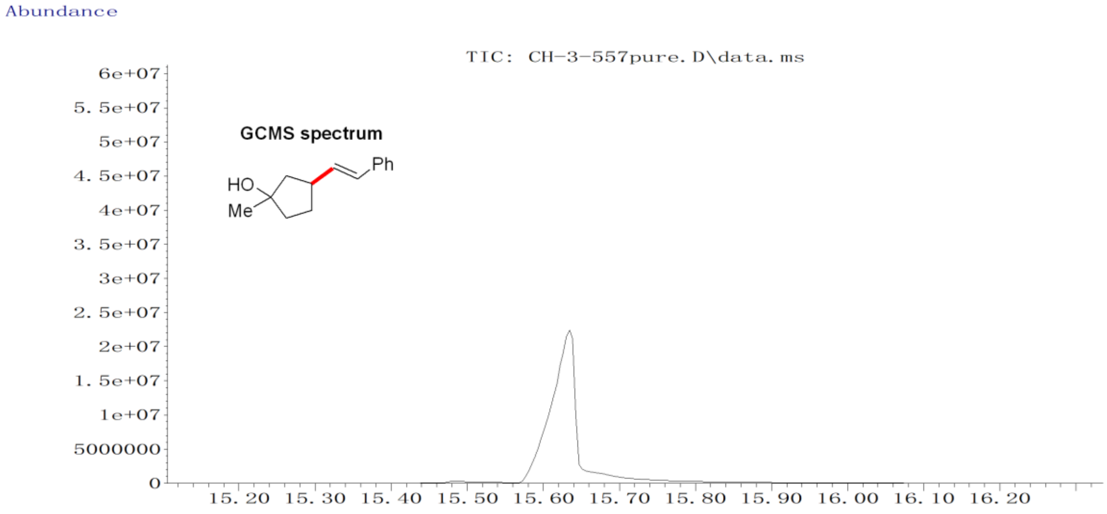

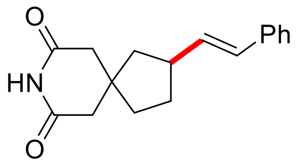

(*E*)-2-styryl-8-azaspiro[4.5]decane-7,9-dione, 20

Following the general procedure for dehydrogenative alkenylation (t = 24 h) with 8-azaspiro[4.5]decane-7,9-dione (33.4 mg, 0.2 mmol) and styrene (208.3 mg, 10 equiv), obtained in 49% yield as a colorless oil (26.2 mg, eluent: hexane/ethyl acetate = 2:1, R_f = 0.3). ^{1}H NMR (400 MHz, $CDCl_3$) δ 7.79 (s, 1H), 7.35 – 7.27 (m, 4H), 7.23 – 7.19 (m, 1H), 6.38 (d, J = 16.0 Hz, 1H), 6.12 (dd, J = 16.0, 7.6 Hz, 1H), 2.93 – 2.78 (m, 1H), 2.60 (s, 2H), 2.59 (s, 2H), 2.04 – 1.99 (m, 1H), 1.89 (dd, J = 13.2, 7.6 Hz, 1H), 1.75 – 1.63 (m, 3H), 1.47 (dd, J = 13.2, 10.4 Hz, 1H). ^{13}C NMR (126 MHz, $CDCl_3$) δ 171.76, 137.20, 133.06, 129.30, 128.55, 127.21, 126.03, 44.94, 44.80, 44.41, 42.47, 40.56, 37.44, 32.02. HRMS APCI $[M+H]^+$ Calculated for $C_{17}H_{20}NO_2$ 270.1489, found 270.1486.

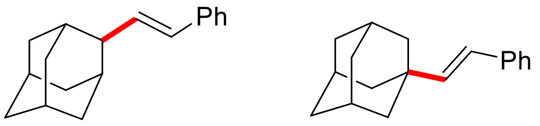

major minor

2-((*E*)-styryl)adamantane (21, major)

1-((*E*)-styryl)adamantane (21, minor)

Following the general procedure for dehydrogenative alkenylation (t = 24 h) with adamantane (27.2 mg, 0.2 mmol) and styrene (208.3 mg, 10 equiv), obtained in 82% yield as a colorless oil (38.9 mg, containing two regioisomers, major : minor = 1.3 : 1, eluent: hexane, $R_f = 0.6$). ^1H NMR (400 MHz, $CDCl_3$) δ 7.41 – 7.34 (m, 2H), 7.33 – 7.27 (m, 2H), 7.22 – 7.16 (m, 1H), 6.51 (dd, J = 16.4, 6.4 Hz, 1H, major), 6.42 (d, J = 16.4 Hz, 1H, major), 6.25 (d, J = 16.4 Hz, 1H, minor), 6.12 (d, J = 16.4 Hz, 1H, minor), 2.60 – 2.54 (m, 1H), 2.08 – 1.55 (m, 14H, major), 2.08 – 1.55 (m, 15H, minor). ^{13}C NMR (126 MHz, $CDCl_3$) δ 142.08, 138.20, 135.07, 128.87, 128.46, 128.43, 128.25, 126.72, 126.67, 125.96, 125.92, 124.49, 47.36, 42.43, 42.23, 38.73, 38.04, 37.26, 36.89, 35.15, 33.07, 32.20, 28.77, 28.48, 28.07, 27.83. HRMS EI $[M]^+$ Calculated for $C_{18}H_{22}$ 238.1716, found 238.1716.

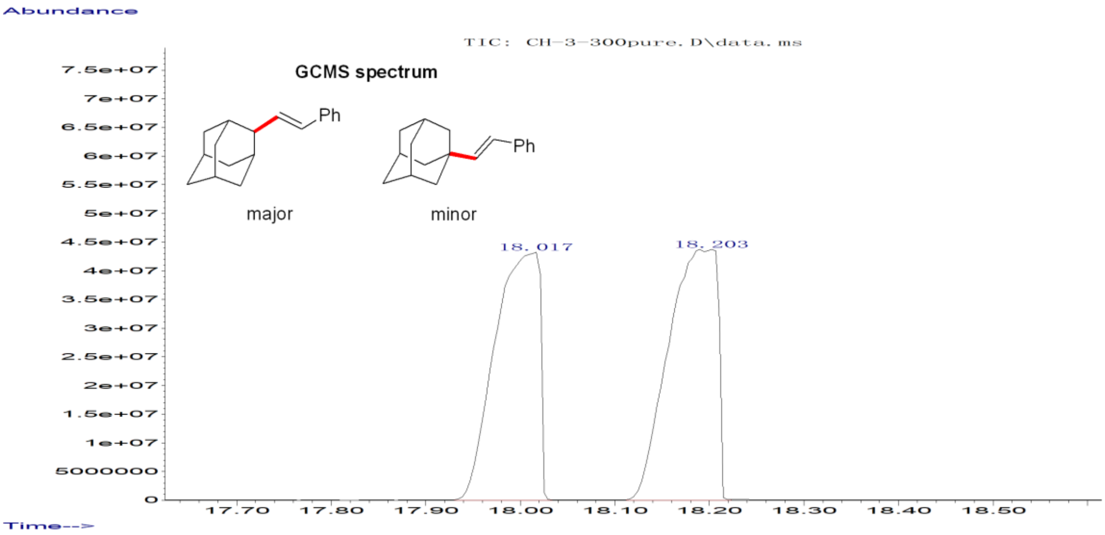

Signal : TIC: CH-3-300pure.D\data.ms

peak #	R.T. min	first scan	max scan	last scan	PK TY	peak height	corr. area	corr. % max.	% of total
1	18.017	3601	3622	3626	rM 4	43202416	142603911	89.97%	47.360%
2	18.203	3645	3667	3672	rM 3	43719728	158505060	100.00%	52.640%

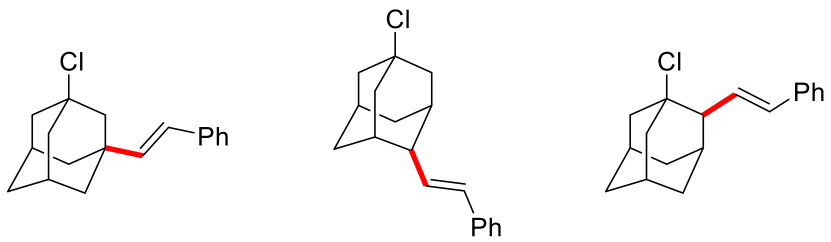

major minor 1 minor 2

1-chloro-3-((*E*)-styryl)adamantane (22, major)

1-chloro-4-((*E*)-styryl)adamantane (22, minor 1)

1-chloro-2-((*E*)-styryl)adamantane (22, minor 2)

Following the general procedure for dehydrogenative alkenylation (t = 24 h) with 1-chloroadamantane (34.1 mg, 0.2 mmol) and styrene (208.3 mg, 10 equiv), obtained in 77% yield as a colorless oil (42.0 mg, containing three regioisomers, 60% selective for major product, eluent: pentane, R_f = 0.5). ^{1}H NMR (400 MHz, $CDCl_3$) δ 7.40 – 7.27 (m, 4H), 7.24 – 7.16 (m, 1H), 6.47 – 6.34 (m, 2H, minor 1 + minor 2), 6.29 (d, J = 16.4 Hz, 1H, major), 6.09 (d, J = 16.4 Hz, 1H, major), 2.62 – 1.44 (m, 14H). ^{13}C NMR (126 MHz, $CDCl_3$) δ 139.19, 137.61, 137.59, 133.02, 132.78, 130.05, 129.90, 128.54, 128.52, 128.36, 128.27, 127.12, 127.08, 126.06, 126.02, 125.98, 125.69, 68.59, 68.41, 51.84, 48.47, 48.01, 46.95, 45.60, 45.25, 42.45, 40.49, 39.46, 36.54, 36.51, 36.17, 34.81, 31.50, 31.17, 30.25. HRMS EI $[M]^+$ Calculated for $C_{18}H_{21}Cl$ 272.1326, found 272.1328.

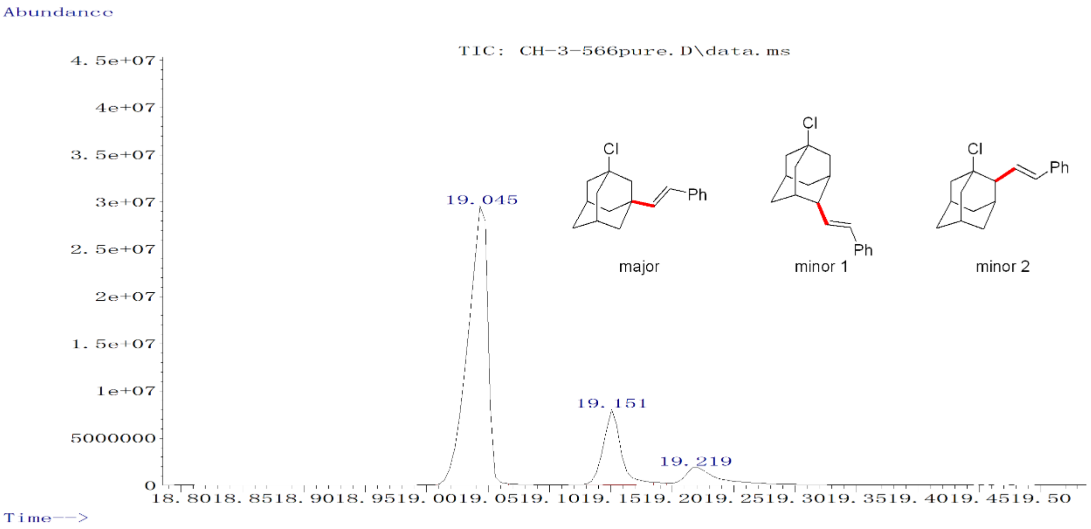

Signal : TIC: CH-3-566pure.D\data.ms

peak #	R.T. min	first scan	max scan	last scan	PK TY	peak height	corr. area	corr. % max.	% of total
1	19.045	3862	3871	3877	M	29857841	334476360	100.00%	74.455%
2	19.151	3892	3897	3905	M	7968572	79218462	23.68%	17.634%
3	19.219	3908	3914	3927	M	1924521	35540380	10.63%	7.911%

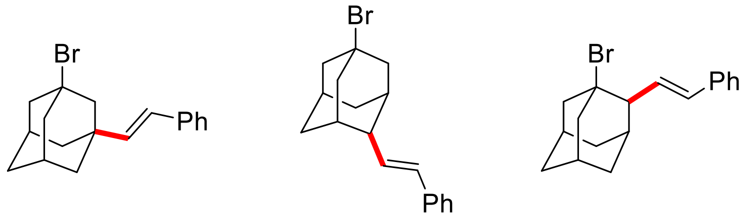

major　　minor 1　　minor 2

1-bromo-3-((*E*)-styryl)adamantane (23, major)

1-bromo-4-((*E*)-styryl)adamantane (23, minor 1)

1-bromo-2-((*E*)-styryl)adamantane (23, minor 2)

Following the general procedure for dehydrogenative alkenylation (t = 24 h) with 1-bromoadamantane (43.0 mg, 0.2 mmol) and styrene (208.3 mg, 10 equiv), obtained in 66% yield as a colorless oil (42.1 mg, containing three regioisomers, 67% selective for major product, eluent: pentane, R_f = 0.5). ^{1}H NMR (400 MHz, $CDCl_3$) δ 7.41 – 7.27 (m, 4H), 7.25 – 7.15 (m, 1H), 6.48 – 6.32 (m, 2H, minor 1 + minor 2), 6.29 (d, J = 16.4 Hz, 1H, major), 6.07 (d, J = 16.4 Hz, 1H, major), 2.68 – 1.42 (m, 14H). ^{13}C NMR (126 MHz, $CDCl_3$) δ 139.10, 137.55, 132.95, 130.05, 129.97, 128.54, 128.52, 128.36, 128.26, 127.15, 127.10, 126.06, 126.03, 125.97, 125.72, 66.19, 65.59, 53.38, 50.02, 49.59, 48.79, 48.47, 45.21, 43.97, 40.40, 40.15, 37.32, 37.06, 36.49, 34.77, 32.28, 32.03, 30.22. HRMS EI $[M]^+$ Calculated for $C_{18}H_{21}Br$ 316.0821, found 316.0824.

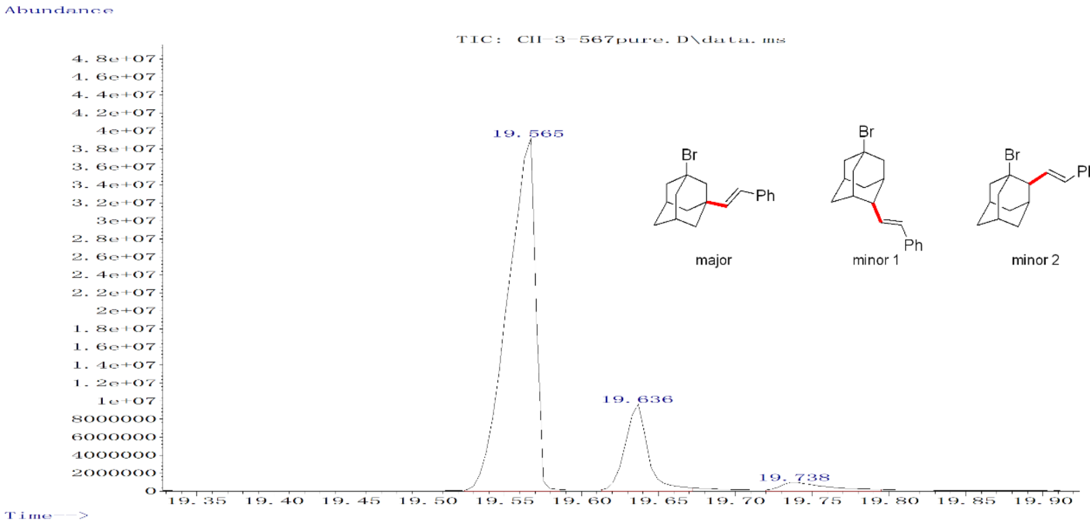

Signal : TIC: CH-3-567pure.D\data.ms

peak #	R.T. min	first scan	max scan	last scan	PK TY	peak height	corr. area	corr. % max.	% of total
1	19.565	3988	3998	4003	M	41121347	494251797	100.00%	80.168%
2	19.636	4009	4015	4023	M	9803636	100275117	20.29%	16.265%
3	19.738	4035	4040	4053	M	1014695	21991080	4.45%	3.567%

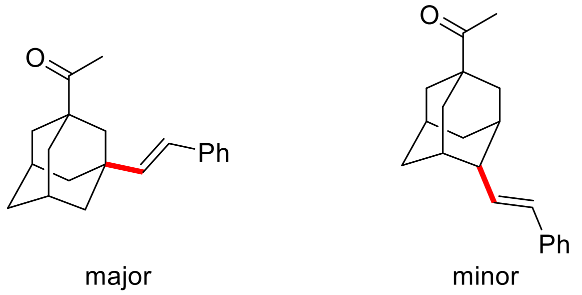

1-(3-((*E*)-styryl)adamantan-1-yl)ethan-1-one (24, major)

1-(4-((*E*)-styryl)adamantan-1-yl)ethan-1-one (24, minor)

Following the general procedure for dehydrogenative alkenylation (t = 24 h) with 1-(adamantan-1-yl)ethan-1-one (35.7 mg, 0.2 mmol) and styrene (208.3 mg, 10 equiv), obtained in 47% yield as a colorless oil (26.5 mg, containing two regioisomers, major : minor = 2.5 : 1, eluent: hexane/ethyl acetate = 20:1, R_f = 0.2). ^{1}H NMR (400 MHz, $CDCl_3$) δ 7.39 – 7.33 (m, 2H), 7.32 – 7.27 (m, 2H), 7.22 – 7.18 (m, 1H), 6.43 (d, J = 16.0 Hz, 1H, minor), 6.38 (dd, J = 16.0, 5.2 Hz, 1H, minor), 6.29 (d, J = 16.4 Hz, 1H, major), 6.12 (d, J = 16.4 Hz, 1H, major), 2.58 – 2.54 (m, 1H, minor), 2.24 – 2.17 (m, 2H, major), 2.13 (s, 3H, major), 2.09 (s, 3H, minor), 2.08 – 2.01 (m, 3H, minor), 1.86 – 1.58 (m, 12H, major), 1.86 – 1.58 (m, 10H, minor). ^{13}C NMR (126 MHz, $CDCl_3$) δ 213.57, 140.54, 137.79, 133.64, 129.56, 128.49, 126.99, 126.94, 126.01, 125.98, 125.34, 47.14, 46.37, 46.07, 42.65, 41.28, 38.66, 37.67, 37.54, 35.77, 35.40, 32.87, 32.74, 28.26, 27.50, 24.46, 24.28. HRMS EI $[M]^+$ Calculated for $C_{20}H_{24}O$ 280.1822, found 280.1823.

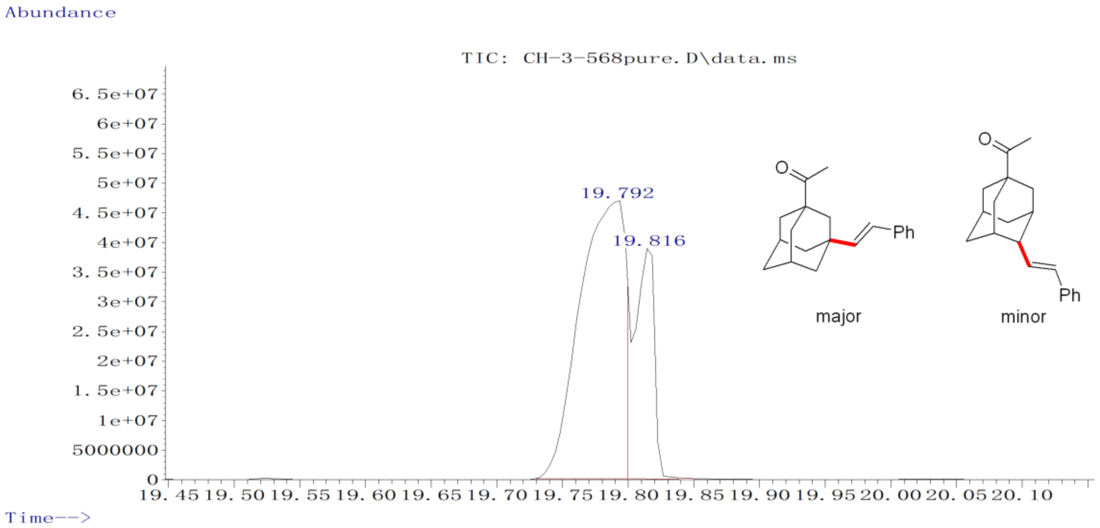

Signal : TIC: CH-3-568pure.D\data.ms

peak #	R.T. min	first scan	max scan	last scan	PK TY	peak height	corr. area	corr. % max.	% of total
1	19.792	4037	4053	4054	M2	47560754	1119049957	100.00%	72.151%
2	19.816	4054	4058	4067	M	39216975	431942749	38.60%	27.849%

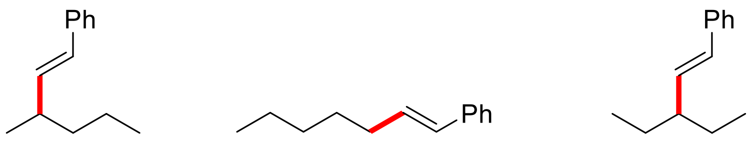

major minor 1 minor 2

(*E*)-(3-methylhex-1-en-1-yl)benzene (25, major)

(*E*)-hept-1-en-1-ylbenzene (25, minor 1)

(*E*)-(3-ethylpent-1-en-1-yl)benzene (25, minor 2)

Following the general procedure for dehydrogenative alkenylation (t = 24 h) with pentane (14.4 mg, 0.2 mmol) and styrene (208.3 mg, 10 equiv), obtained in 39% yield as a colorless liquid (13.7 mg, containing three regioisomers, major : minor 1 : minor 2 = 4.2 : 1.1 : 1, eluent: hexane, R_f = 0.7). ^{1}H NMR (400 MHz, $CDCl_3$) δ 7.38 – 7.32 (m, 2H), 7.32 – 7.26 (m, 2H), 7.22 – 7.15 (m, 1H), 6.37 (d, J = 16.0 Hz, 1H, minor 2), 6.33 (d, J = 16.0 Hz, 1H, major), 6.33 (d, J = 16.0 Hz, 1H, minor 1), 6.23 (dt, J = 16.0, 6.8 Hz, 1H, minor 1), 6.09 (dd, J = 16.0, 8.0 Hz, 1H, major), 5.95 (dd, J = 16.0, 8.8 Hz, 1H, minor 2), 2.36 – 2.25 (m, 1H, major), 2.24 – 2.17 (m, 2H, minor 1), 1.97 – 1.91 (m, 1H, minor 2), 1.53 – 1.44 (m, 2H, minor 1), 1.53 – 1.44 (m, 4H, minor 2), 1.38 – 1.31 (m, 4H, major), 1.38 – 1.31 (m, 4H, minor 1), 1.07 (d, J = 6.8 Hz, 3H, major), 0.93 – 0.87 (m, 3H, major), 0.93 – 0.87 (m, 3H, minor 1), 0.93 – 0.87 (m, 6H, minor 2). ^{13}C NMR (126 MHz, $CDCl_3$) δ 137.98, 137.06, 135.29, 131.26, 129.80, 129.67, 128.44, 127.90, 126.71, 125.94, 125.89, 46.83, 39.34, 36.98, 33.00, 31.43, 29.05, 27.78, 22.55, 20.63, 20.47, 14.16, 14.05, 11.83. HRMS EI $[M]^+$ Calculated for $C_{13}H_{18}$ 174.1403, found 174.1402. NMR data of major[21], minor 1[22] and minor 2[23] product is in agreement with literature.

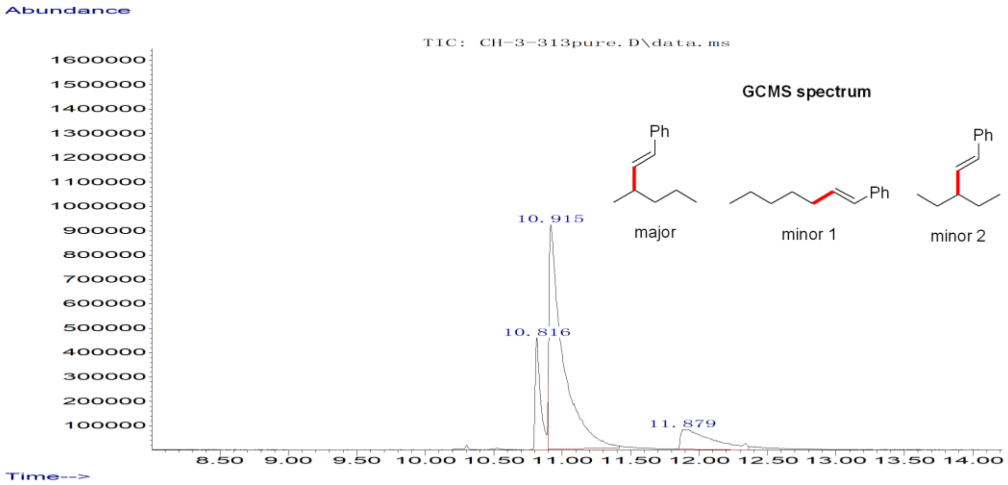

Signal : TIC: CH-3-313pure.D\data.ms

peak #	R.T. min	first scan	max scan	last scan	PK TY	peak height	corr. area	corr. % max.	% of total
1	10.816	1868	1875	1895	rM	461249	1346992	20.96%	14.864%
2	10.915	1895	1899	2021	rM 3	921519	6426745	100.00%	70.920%
3	11.879	2122	2133	2250	rM 7	80903	1288286	20.05%	14.216%

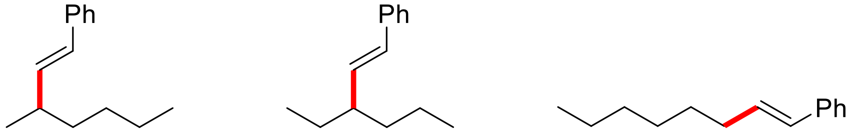

major minor 1 minor 2

(*E*)-(3-methylhept-1-en-1-yl)benzene (26, major)

(*E*)-(3-ethylhex-1-en-1-yl)benzene (26, minor 1)

(*E*)-oct-1-en-1-ylbenzene (26, minor 2)

Following the general procedure for dehydrogenative alkenylation (t = 24 h) with hexane (15.8 mg, 0.2 mmol) and styrene (208.3 mg, 10 equiv), obtained in 42% yield as a colorless liquid (21.4 mg, containing three regioisomers, major : minor 1 : minor 2 = 3.7 : 1.7 : 1, eluent: hexane, R_f = 0.7). ^{1}H NMR (400 MHz, $CDCl_3$) δ 7.38 – 7.32 (m, 2H), 7.32 – 7.26 (m, 2H), 7.21 – 7.16 (m, 1H), 6.37 (d, J = 16.0 Hz, 1H, minor 2), 6.33 (d, J = 16.0 Hz, 1H, major), 6.33 (d, J = 16.0 Hz, 1H, minor 1), 6.23 (dt, J = 16.0, 6.8 Hz, 1H, minor 2), 6.10 (dd, J = 16.0, 8.0 Hz, 1H, major), 5.95 (dd, J = 16.0, 8.8 Hz, 1H, minor 1), 2.33 – 2.24 (m, 1H, major), 2.24 – 2.18 (m, 2H, minor 2), 2.09 – 1.99 (m, 1H, minor 1), 1.41 – 1.21 (m, 6H, major), 1.41 – 1.21 (m, 4H, minor 1), 1.41 – 1.21 (m, 8H, minor 2), 1.07 (d, J = 6.8 Hz, 3H, major), 0.91 – 0.85 (m, 3H, major), 0.91 – 0.85 (m, 6H, minor 1), 0.91 – 0.85 (m, 3H, minor 2). ^{13}C NMR (126 MHz, $CDCl_3$) δ 137.99, 137.11, 135.56, 131.26, 129.66,

129.58, 128.44, 127.89, 126.71, 126.69, 125.94, 125.89, 44.87, 37.37, 37.25, 36.82, 33.04, 31.92, 31.75, 29.69, 29.35, 28.90, 28.18, 22.83, 22.62, 20.67, 20.46, 14.20, 14.09, 11.83. HRMS EI $[M]^+$ Calculated for $C_{14}H_{20}$ 188.1560, found 188.1561. NMR data of major[24], minor 2[25] and all three isomers[26] is in agreement with literature.

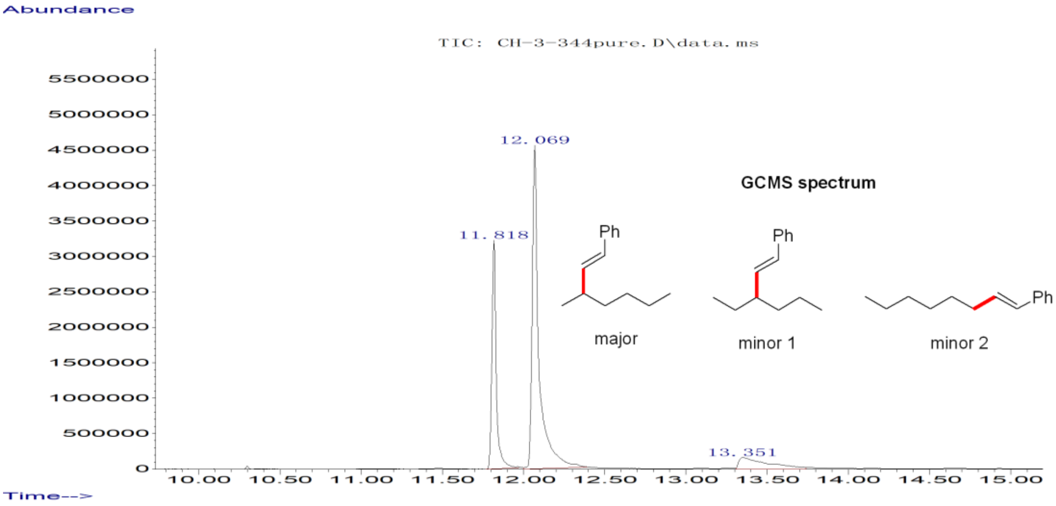

peak #	R.T. min	first scan	max scan	last scan	PK TY	peak height	corr. area	corr. % max.	% of total
1	11.818	2106	2118	2155	rM	3225716	6026356	45.01%	28.115%
2	12.069	2162	2179	2258	rM	4562600	13389882	100.00%	62.468%
3	13.351	2480	2490	2584	rM 5	161558	2018464	15.07%	9.417%

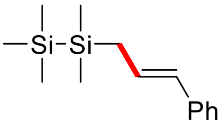

1-cinnamyl-1,1,2,2,2-pentamethyldisilane, 27

Following the general procedure for dehydrogenative alkenylation (t = 24 h) with 1,1,1,2,2,2-hexamethyldisilane (29.3 mg, 0.2 mmol) and styrene (208.3 mg, 10 equiv), obtained in 50% yield as a colorless liquid (24.7 mg, eluent: hexane, $R_f = 0.6$). ^{1}H NMR (400 MHz, $CDCl_3$) δ 7.35 – 7.27 (m, 4H), 7.18 – 7.12 (m, 1H), 6.26 – 6.21 (m, 2H), 1.77 – 1.72 (m, 2H), 0.08 (s, 6H), 0.08 (s, 9H). ^{13}C NMR (126 MHz, $CDCl_3$) δ 128.49, 128.43, 128.15, 128.05, 126.16, 125.47, 22.02, -2.08, -4.44. HRMS EI $[M]^+$ Calculated for $C_{14}H_{24}Si_2$ 248.1411, found 248.1411.

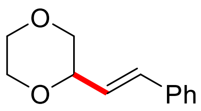

(*E*)-2-styryl-1,4-dioxane, 28

Following the general procedure for dehydrogenative alkenylation (t = 24 h) with 1,4-dioxane (17.6 mg, 0.2 mmol) and styrene (208.3 mg, 10 equiv), obtained in 46% yield as a colorless oil (17.5 mg, eluent: hexane/ethyl acetate = 20:1, R_f = 0.2). ^{1}H NMR (400 MHz, $CDCl_3$) δ 7.39 – 7.30 (m, 4H), 7.28 – 7.25 (m, 1H), 6.69 (d, J = 16.0 Hz, 1H), 6.09 (dd, J = 16.0, 6.4 Hz, 1H), 4.35 – 4.14 (m, 1H), 3.91 – 3.59 (m, 5H), 3.47 – 3.39 (m, 1H). ^{13}C NMR (126 MHz, $CDCl_3$) δ 136.38, 132.66, 128.55, 127.90, 126.49, 125.10, 76.06, 70.93, 66.59, 66.28. HRMS EI $[M]^+$ Calculated for $C_{12}H_{14}O_2$ 190.0988, found 190.0990.

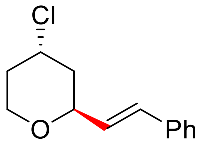

(2*S*,4*S*)-4-chloro-2-((*E*)-styryl)tetrahydro-2*H*-pyran, 29

Following the general procedure for dehydrogenative alkenylation (t = 24 h) with 4-chlorotetrahydro-2*H*-pyran (24.1 mg, 0.2 mmol) and styrene (208.3 mg, 10 equiv), obtained in 59% yield as a colorless oil (26.4 mg, eluent: hexane : ethyl acetate = 50/1, R_f = 0.1). ^{1}H NMR (400 MHz, $CDCl_3$) δ 7.36 – 7.29 (m, 2H), 7.29 – 7.23 (m, 2H), 7.21 – 7.16 (m, 1H), 6.59 (dd, J = 16.0, 1.2 Hz, 1H), 6.12 (dd, J = 16.0, 6.0 Hz, 1H), 4.57 (p, J = 3.2, 3.2, 3.2, 3.2 Hz, 1H), 4.52 – 4.44 (m, 1H), 4.02 (td, J = 12.0, 2.0 Hz, 1H), 3.89 (dd, J = 12.0, 4.8 Hz, 1H), 2.13 – 2.04 (m, 1H), 2.03 – 1.96 (m, 1H), 1.94 – 1.87 (m, 1H), 1.82 – 1.77 (m, 1H). ^{13}C NMR (126 MHz, $CDCl_3$) δ 136.65, 130.81, 129.26, 128.52, 127.67, 126.43, 71.93, 62.25, 56.02, 39.60, 33.48. HRMS EI $[M]^+$ Calculated for $C_{13}H_{15}ClO$ 222.0806, found 222.0805.

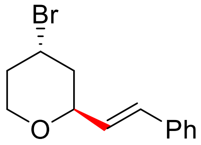

(2*S*,4*S*)-4-bromo-2-((*E*)-styryl)tetrahydro-2*H*-pyran, 30

Following the general procedure for dehydrogenative alkenylation (t = 24 h) with 4-bromotetrahydro-2*H*-pyran (33.0 mg, 0.2 mmol) and styrene (208.3 mg, 10 equiv), obtained in 67% yield as a colorless oil (35.8 mg, eluent: hexane : ethyl acetate = 50/1, R_f = 0.1). ^{1}H NMR (400 MHz, $CDCl_3$) δ 7.41 – 7.34 (m, 2H), 7.34 – 7.28 (m, 2H), 7.26 – 7.22 (m, 1H), 6.64 (dd, J = 16.0, 1.2 Hz, 1H), 6.18 (dd, J = 16.0, 6.0 Hz, 1H), 4.76 (p, J = 3.2, 3.2, 3.2, 3.2 Hz, 1H), 4.49 – 4.60 (m, 1H), 4.07 (td, J = 11.6, 2.0 Hz, 1H), 3.96 (dd, J = 11.6, 4.8 Hz, 1H), 2.21 – 2.11 (m, 2H), 2.03 – 1.91 (m, 2H). ^{13}C NMR (126 MHz, $CDCl_3$) δ 136.64, 130.91, 129.12, 128.53, 127.69, 126.45, 72.60, 62.93, 49.68, 40.08, 34.02. HRMS EI $[M]^+$ Calculated for $C_{13}H_{15}BrO$ 266.0301, found 266.0302.

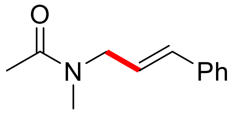

***N*-cinnamyl-*N*-methylacetamide, 31**

Following the general procedure for dehydrogenative alkenylation (t = 24 h) with *N*,*N*-dimethylacetamide (17.4 mg, 0.2 mmol) and styrene (208.3 mg, 10 equiv), obtained in 65% yield as a colorless oil (24.5 mg, eluent: hexane : ethyl acetate = 1/1, R_f = 0.2). ^{1}H NMR (400 MHz, $CDCl_3$) δ 7.39 – 7.22 (m, 5H), 6.49 (d, J = 16.0 Hz, 1H, rotamer 1), 6.46 (d, J = 16.0 Hz, 1H, rotamer 2), 6.14 (dt, J = 16.0, 6.4 Hz, 1H, rotamer 1), 6.12 (dt, J = 16.0, 5.6 Hz, 1H, rotamer 2), 4.14 (dd, J = 6.4, 1.2 Hz, 2H, rotamer 1), 4.05 (dd, J = 5.6, 1.6 Hz, 2H, rotamer 2), 2.98 (s, 3H, rotamer 1), 2.97 (s, 3H, rotamer 2), 2.14 (s, 3H, rotamer 1), 2.12 (s, 3H, rotamer 2). ^{13}C NMR (126 MHz, $CDCl_3$) δ 170.78, 170.45, 136.53, 136.06, 132.75, 131.70, 128.64, 128.51, 127.91, 127.62, 126.34, 126.33, 124.53, 123.63, 52.57, 49.24, 35.39, 33.44, 21.78, 21.26. HRMS EI $[M]^+$ Calculated for $C_{12}H_{15}NO$ 189.1148, found 189.1149.

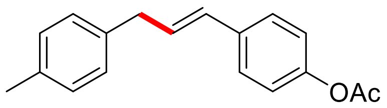

(*E*)-4-(3-(*p*-tolyl)prop-1-en-1-yl)phenyl acetate, 32

Following the general procedure for dehydrogenative alkenylation (t = 24 h) with *p*-xylene (21.2 mg, 0.2 mmol) and 4-vinylphenyl acetate (324.4 mg, 10 equiv), obtained in 38% yield as a yellow oil (20.2 mg, eluent: hexane : ethyl acetate = 20/1, R_f = 0.35). ^{1}H NMR (400 MHz, $CDCl_3$) δ 7.38 – 7.33 (m, 2H), 7.13 (s, 4H), 7.04 – 6.99 (m, 2H), 6.43 (d, J = 15.6 Hz, 1H), 6.30 (dt, J = 15.6, 6.8 Hz, 1H), 3.51 (dd, J = 6.8, 0.8 Hz, 2H), 2.34 (s, 3H), 2.29 (s, 3H). ^{13}C NMR (126 MHz, $CDCl_3$) δ 169.48, 149.62, 136.91, 135.70, 135.37, 129.85, 129.81, 129.17, 128.51, 126.99, 121.54, 38.85, 21.10, 20.99. HRMS EI $[M]^+$ Calculated for $C_{18}H_{18}O_2$ 266.1301, found 266.1300.

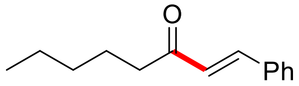

(*E*)-1-phenyloct-1-en-3-one, 33

Following the general procedure for dehydrogenative alkenylation (t = 12 h) with hexanal (20.0 mg, 0.2 mmol) and styrene (208.3 mg, 10 equiv), obtained in 68% yield as a colorless oil (27.6 mg, eluent: hexane : ethyl acetate = 50/1, R_f = 0.1). ^{1}H NMR (400 MHz, $CDCl_3$) δ 7.56 (d, J = 16.4 Hz, 1H), 7.59 – 7.53 (m, 2H), 7.43 – 7.36 (m, 3H), 6.75 (d, J = 16.4 Hz, 1H), 2.70 – 2.63 (m, 2H), 1.72 – 1.66 (m, 2H), 1.38 – 1.32 (m, 4H), 0.92 (t, J = 6.8 Hz, 3H). ^{13}C NMR (126 MHz, $CDCl_3$) δ 200.70, 142.29, 134.61, 130.35, 128.92, 128.22, 126.28, 40.93, 31.50, 24.07, 22.49, 13.93. HRMS EI $[M]^+$ Calculated for $C_{14}H_{18}O$ 202.1352, found 202.1350.

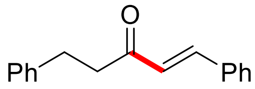

(*E*)-1,5-diphenylpent-1-en-3-one, 34

Following the general procedure for dehydrogenative alkenylation (t = 12 h) with 3-phenylpropanal (26.8 mg, 0.2 mmol) and styrene (208.3 mg, 10 equiv), obtained in 84% yield as a colorless oil (39.8

mg, eluent: hexane : ethyl acetate = 50/1, R_f = 0.1). ^{1}H NMR (400 MHz, $CDCl_3$) δ 7.61 – 7.48 (m, 3H), 7.43 – 7.37 (m, 3H), 7.33 – 7.21 (m, 5H), 6.75 (d, J = 16.4 Hz, 1H), 3.02 (s, 4H). ^{13}C NMR (126 MHz, $CDCl_3$) δ 199.28, 142.67, 141.18, 134.44, 130.45, 128.92, 128.49, 128.36, 128.23, 126.13, 126.10, 42.42, 30.12. HRMS EI $[M]^+$ Calculated for $C_{17}H_{16}O$ 236.1196, found 236.1192.

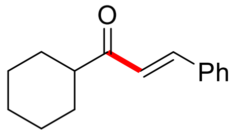

(*E*)-1-cyclohexyl-3-phenylprop-2-en-1-one, 35

Following the general procedure for dehydrogenative alkenylation (t = 12 h) with cyclohexanecarbaldehyde (22.4 mg, 0.2 mmol) and styrene (208.3 mg, 10 equiv), obtained in 52% yield as a colorless oil (22.2 mg, eluent: hexane : ethyl acetate = 100/1, R_f = 0.1). ^{1}H NMR (400 MHz, $CDCl_3$) δ 7.66 – 7.49 (m, 3H), 7.44 – 7.34 (m, 3H), 6.82 (d, J = 16.0 Hz, 1H), 2.66 (tt, J = 11.2, 3.2 Hz, 1H), 1.91 – 1.72 (m, 4H), 1.47 – 1.26 (m, 6H). ^{13}C NMR (126 MHz, $CDCl_3$) δ 203.16, 142.21, 134.76, 130.27, 128.88, 128.25, 124.73, 49.41, 28.71, 25.90, 25.76. HRMS EI $[M]^+$ Calculated for $C_{15}H_{18}O$ 214.1352, found 214.1352.

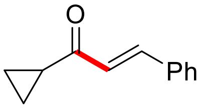

(*E*)-1-cyclopropyl-3-phenylprop-2-en-1-one, 36

Following the general procedure for dehydrogenative alkenylation (t = 12 h) with cyclopropanecarbaldehyde (14.0 mg, 0.2 mmol) and styrene (208.3 mg, 10 equiv), obtained in 43% yield as a colorless oil (14.9 mg, eluent: hexane : ethyl acetate = 40/1, R_f = 0.1). ^{1}H NMR (400 MHz, $CDCl_3$) δ 7.62 (d, J = 16.0 Hz, 1H), 7.59 – 7.54 (m, 2H), 7.44 – 7.36 (m, 3H), 6.88 (d, J = 16.0 Hz, 1H), 2.26 (tt, J = 8.0, 4.4 Hz, 1H), 1.20 – 1.14 (m, 2H), 1.01 – 0.95 (m, 2H). ^{13}C NMR (126 MHz, $CDCl_3$) δ 200.05, 141.99, 134.72, 130.30, 128.91, 128.26, 126.45, 19.63, 11.30. HRMS APCI $[M+H]^+$ Calculated for $C_{12}H_{13}O$ 173.0961, found 173.0955.

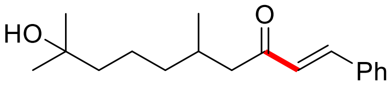

(*E*)-9-hydroxy-5,9-dimethyl-1-phenyldec-1-en-3-one, 37

Following the general procedure for dehydrogenative alkenylation (t = 12 h) with 7-hydroxy-3,7-dimethyloctanal (34.5 mg, 0.2 mmol) and styrene (208.3 mg, 10 equiv), obtained in 72% yield as a colorless oil (39.7 mg, eluent: hexane : ethyl acetate = 10/1, R_f = 0.1). ^1H NMR (400 MHz, $CDCl_3$) δ 7.60 – 7.47 (m, 3H), 7.43 – 7.35 (m, 3H), 6.75 (d, J = 16.4 Hz, 1H), 2.65 (dd, J = 15.2, 6.0 Hz, 1H), 2.48 (dd, J = 15.2, 8.0 Hz, 1H), 1.48 – 1.34 (m, 7H), 1.22 (s, 6H), 0.96 (d, J = 6.8 Hz, 3H). ^{13}C NMR (126 MHz, $CDCl_3$) δ 200.39, 142.40, 134.57, 130.40, 128.92, 128.26, 126.60, 70.97, 48.41, 43.93, 37.48, 29.81, 29.35, 29.19, 21.70, 19.93. HRMS EI $[M]^+$ Calculated for $C_{18}H_{26}O_2$ 274.1933, found 274.1933.

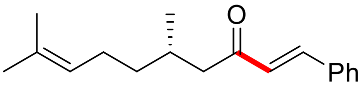

(*S*,*E*)-5,9-dimethyl-1-phenyldeca-1,8-dien-3-one, 38

Following the general procedure for dehydrogenative alkenylation (t = 12 h) with (*S*)-3,7-dimethyloct-6-enal (30.9 mg, 0.2 mmol) and styrene (208.3 mg, 10 equiv), obtained in 68% yield as a colorless oil (35.0 mg, eluent: hexane : ethyl acetate = 120/1, R_f = 0.1). ^1H NMR (400 MHz, $CDCl_3$) δ 7.60 – 7.49 (m, 3H), 7.43 – 7.35 (m, 3H), 6.75 (d, J = 16.0 Hz, 1H), 5.11 (dddt, J = 8.4, 5.6, 2.8, 1.2 Hz, 1H), 2.66 (dd, J = 15.2, 5.6 Hz, 1H), 2.46 (dd, J = 15.2, 8.4 Hz, 1H), 2.17 – 1.94 (m, 3H), 1.69 (s, 3H), 1.61 (s, 3H), 1.45 – 1.22 (m, 2H), 0.96 (d, J = 6.8 Hz, 3H). ^{13}C NMR (126 MHz, $CDCl_3$) δ 200.42, 142.33, 134.62, 131.50, 130.37, 128.92, 128.25, 126.60, 124.36, 48.40, 37.13, 29.60, 25.70, 25.52, 19.84, 17.66. HRMS EI $[M]^+$ Calculated for $C_{18}H_{24}O$ 256.1822, found 256.1819.

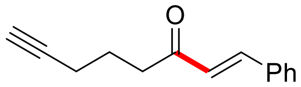

(*E*)-1-phenyloct-1-en-7-yn-3-one, 39

Following the general procedure for dehydrogenative alkenylation (t = 12 h) with hex-5-ynal (19.2 mg, 0.2 mmol) and styrene (208.3 mg, 10 equiv), obtained in 46% yield as a colorless oil (18.2 mg, eluent: hexane : ethyl acetate = 50/1, R_f = 0.15). ^{1}H NMR (400 MHz, $CDCl_3$) δ 7.64 – 7.51 (m, 3H), 7.43 – 7.36 (m, 3H), 6.75 (d, J = 16.4 Hz, 1H), 2.84 (t, J = 7.2 Hz, 2H), 2.31 (td, J = 6.8, 2.8 Hz, 2H), 1.99 (t, J = 2.8 Hz, 1H), 1.96 – 1.87 (m, 2H). ^{13}C NMR (126 MHz, $CDCl_3$) δ 199.60, 142.61, 134.48, 130.47, 128.94, 128.26, 126.21, 83.71, 69.06, 39.15, 22.75, 17.89. HRMS EI $[M]^+$ Calculated for $C_{14}H_{14}O$ 198.1039, found 198.1044.

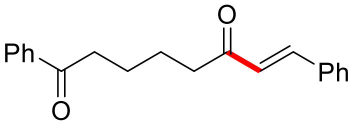

(*E*)-1,8-diphenyloct-7-ene-1,6-dione, 40

Following the general procedure for dehydrogenative alkenylation (t = 20 h) with 6-oxo-6-phenylhexanal (38.0 mg, 0.2 mmol) and styrene (208.3 mg, 10 equiv), obtained in 71% yield as a light yellow oil (41.5 mg, eluent: hexane : ethyl acetate = 10/1, R_f = 0.2). ^{1}H NMR (400 MHz, $CDCl_3$) δ 7.99 – 7.93 (m, 2H), 7.61 – 7.35 (m, 9H), 6.75 (d, J = 16.4 Hz, 1H), 3.03 (t, J = 6.8 Hz, 2H), 2.74 (t, J = 6.8 Hz, 2H), 1.86 – 1.75 (m, 4H). ^{13}C NMR (126 MHz, $CDCl_3$) δ 200.07, 200.00, 142.52, 136.92, 134.48, 132.95, 130.40, 128.90, 128.55, 128.24, 127.99, 126.12, 40.62, 38.31, 23.87, 23.84. HRMS APCI $[M+H]^+$ Calculated for $C_{20}H_{21}O_2$ 293.1536, found 293.1537.

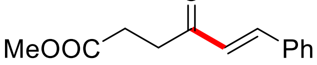

methyl (*E*)-4-oxo-6-phenylhex-5-enoate, 41

Following the general procedure for dehydrogenative alkenylation (t = 12 h) with methyl 4-oxobutanoate (23.2 mg, 0.2 mmol) and styrene (208.3 mg, 10 equiv), obtained in 49% yield as a

colorless oil (21.5 mg, eluent: hexane : ethyl acetate = 10/1, R_f = 0.1). ^{1}H NMR (400 MHz, $CDCl_3$) δ 7.60 (d, J = 16.4 Hz, 1H), 7.57 – 7.52 (m, 2H), 7.43 – 7.37 (m, 3H), 6.76 (d, J = 16.4 Hz, 1H), 3.70 (s, 3H), 3.03 (t, J = 6.8 Hz, 2H), 2.71 (t, J = 6.8 Hz, 2H). ^{13}C NMR (126 MHz, $CDCl_3$) δ 197.95, 173.33, 142.94, 134.37, 130.54, 128.94, 128.29, 125.80, 51.81, 35.23, 27.91. HRMS EI $[M]^+$ Calculated for $C_{13}H_{14}O_3$ 218.0937, found 218.0935.

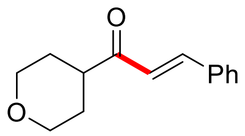

(*E*)-3-phenyl-1-(tetrahydro-2*H*-pyran-4-yl)prop-2-en-1-one, 42

Following the general procedure for dehydrogenative alkenylation (t = 12 h) with tetrahydro-2*H*-pyran-4-carbaldehyde (22.8 mg, 0.2 mmol) and styrene (208.3 mg, 10 equiv), obtained in 47% yield as a colorless oil (20.3 mg, eluent: hexane : ethyl acetate = 10/1, R_f = 0.2). ^{1}H NMR (500 MHz, $CDCl_3$) δ 7.64 (d, J = 16.0 Hz, 1H), 7.60 - 7.53 (m, 2H), 7.43 – 7.37 (m, 3H), 6.84 (d, J = 16.0 Hz, 1H), 4.09 – 4.01 (m, 2H), 3.55 – 3.47 (m, 2H), 2.92 – 2.83 (m, 1H), 1.85 – 1.78 (m, 4H). ^{13}C NMR (126 MHz, $CDCl_3$) δ 200.85, 143.02, 134.48, 130.55, 128.95, 128.33, 126.18, 67.29, 46.26, 28.34. HRMS EI $[M]^+$ Calculated for $C_{14}H_{16}O_2$ 216.1145, found 216.1144.

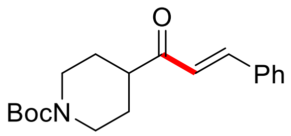

***tert*-butyl 4-cinnamoylpiperidine-1-carboxylate, 43**

Following the general procedure for dehydrogenative alkenylation (t = 12 h) with *tert*-butyl 4-formylpiperidine-1-carboxylate (42.7 mg, 0.2 mmol) and styrene (208.3 mg, 10 equiv), obtained in 44% yield as a colorless oil (28.0 mg, eluent: hexane : ethyl acetate = 10/1, R_f = 0.2). ^{1}H NMR (500 MHz, $CDCl_3$) δ 7.63 (d, J = 16.0 Hz, 1H), 7.60 - 7.52 (m, 2H), 7.44 – 7.37 (m, 3H), 6.83 (d, J = 16.0 Hz, 1H), 4.40 – 3.80 (m, 4H), 2.82 – 2.76 (m, 1H), 1.91 – 1.81 (m, 2H), 1.68 – 1.62 (m, 2H), 1.47 (s, 9H). ^{13}C NMR (126 MHz, $CDCl_3$) δ 201.14, 154.71, 143.06, 134.46, 130.58, 128.95, 128.35,

126.19, 79.60, 47.23, 43.22, 28.44, 27.71. HRMS ESI $[M+H]^+$ Calculated for $C_{19}H_{26}NO_3$ 316.1913, found 316.1912.

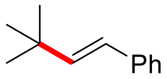

(*E*)-(3,3-dimethylbut-1-en-1-yl)benzene, 44

Following the general procedure for dehydrogenative alkenylation (t = 24 h) with pivalaldehyde (17.2 mg, 0.2 mmol) and styrene (208.3 mg, 10 equiv), obtained in 70% yield as a colorless liquid (22.4 mg, eluent: hexane, R_f = 0.7). ^1H NMR (400 MHz, $CDCl_3$) δ 7.40 – 7.33 (m, 2H), 7.33 – 7.26 (m, 2H), 7.23 – 7.16 (m, 1H), 6.36 – 6.22 (m, 2H), 1.13 (s, 9H). ^{13}C NMR (101 MHz, $CDCl_3$) δ 141.85, 138.06, 128.45, 126.72, 126.01, 124.57, 33.33, 29.59. HRMS EI $[M]^+$ Calculated for $C_{12}H_{16}$ 160.1252, found 160.1252.

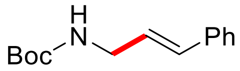

***tert*-butyl cinnamylcarbamate, 45**

Following the general procedure for dehydrogenative alkenylation (t = 24 h) with *tert*-butyl (2-oxoethyl)carbamate (31.8 mg, 0.2 mmol) and styrene (208.3 mg, 10 equiv), obtained in 79% yield as a light yellow oil (35.2 mg, eluent: hexane : ethyl acetate = 20/1, R_f = 0.1). ^1H NMR (400 MHz, $CDCl_3$) δ 7.36 – 7.29 (m, 4H), 7.25 – 7.21 (m, 1H), 6.51 (d, J = 16.0 Hz, 1H), 6.19 (dt, J = 16.0, 6.0 Hz, 1H), 4.66 (br. s, 1H), 3.91 (t, J = 4.8 Hz, 2H), 1.47 (s, 9H). ^{13}C NMR (126 MHz, $CDCl_3$) δ 155.77, 136.68, 131.46, 128.55, 127.58, 126.34, 79.72, 42.76, 28.41. The ^1H and ^{13}C NMR spectra are in agreement with those reported in the literature.[27]

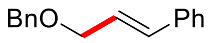

(*E*)-(3-(benzyloxy)prop-1-en-1-yl)benzene, 46

Following the general procedure for dehydrogenative alkenylation (t = 24 h) with 2-

(benzyloxy)acetaldehyde (30.0 mg, 0.2 mmol) and styrene (208.3 mg, 10 equiv), obtained in 62% yield as a colorless oil (28.0 mg, eluent: hexane, R_f = 0.4). ^{1}H NMR (400 MHz, $CDCl_3$) δ 7.43 – 7.22 (m, 10H), 6.64 (d, J = 16.0 Hz, 1H), 6.34 (dt, J = 16.0, 6.0 Hz, 1H), 4.59 (s, 2H), 4.21 (dd, J = 6.0, 1.6 Hz, 2H). ^{13}C NMR (101 MHz, $CDCl_3$) δ 138.28, 136.73, 132.51, 128.54, 128.41, 127.79, 127.66, 127.63, 126.48, 126.09, 72.17, 70.75. HRMS APCI $[M+H]^+$ $C_{16}H_{17}O$ 225.1279, found 225.1273.

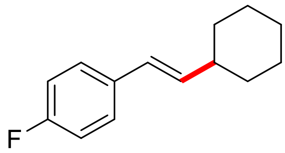

(*E*)-1-(2-cyclohexylvinyl)-4-fluorobenzene, 47

Following the general procedure for dehydrogenative alkenylation (t = 24 h) with cyclohexane (16.8 mg, 0.2 mmol) and 1-fluoro-4-vinylbenzene (244.3 mg, 10 equiv), obtained in 76% yield as a colorless liquid (31.2 mg, eluent: hexane, R_f = 0.6). ^{1}H NMR (400 MHz, $CDCl_3$) δ 7.33 – 7.27 (m, 2H), 7.02 – 6.92 (m, 2H), 6.30 (d, J = 16.0 Hz, 1H), 6.08 (dd, J = 16.0, 7.2 Hz, 1H), 2.11 (tdt, J = 10.4, 7.2, 3.6 Hz, 1H), 1.85 – 1.63 (m, 5H), 1.33 – 1.16 (m, 5H). ^{13}C NMR (126 MHz, $CDCl_3$) δ 161.84 (d, J = 245.8 Hz), 136.59 (d, J = 1.8 Hz), 134.19 (d, J = 3.5 Hz), 127.30 (d, J = 7.9 Hz), 126.06, 115.25 (d, J = 21.4 Hz), 41.09, 32.94, 26.14, 26.02. ^{19}F NMR (377 MHz, $CDCl_3$) δ -116.06 (s, 1F). HRMS EI $[M]^+$ Calculated for $C_{14}H_{17}F$ 204.1309, found 204.1310.

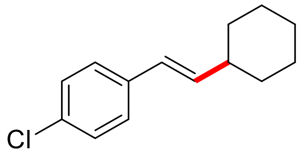

(*E*)-1-chloro-4-(2-cyclohexylvinyl)benzene, 48

Following the general procedure for dehydrogenative alkenylation (t = 24 h) with cyclohexane (16.8 mg, 0.2 mmol) and 1-chloro-4-vinylbenzene (277.2 mg, 10 equiv), obtained in 79% yield as a colorless liquid (35.0 mg, eluent: hexane, R_f = 0.6). ^{1}H NMR (400 MHz, $CDCl_3$) δ 7.34 – 7.23 (m, 4H), 6.29 (dd, J = 16.0, 0.8 Hz, 1H), 6.15 (dd, J = 16.0, 6.8 Hz, 1H), 2.17 – 2.07 (m, 1H), 1.83 –

1.65 (m, 5H), 1.33 – 1.18 (m, 5H). ^{13}C NMR (126 MHz, $CDCl_3$) δ 137.55, 136.56, 132.20, 128.54, 127.13, 126.08, 41.11, 32.85, 26.12, 25.99. HRMS EI $[M]^+$ Calculated for $C_{14}H_{17}Cl$ 220.1013, found 220.1015.

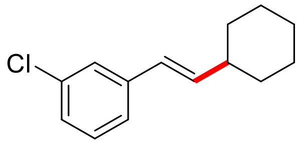

(*E*)-1-chloro-3-(2-cyclohexylvinyl)benzene, 49

Following the general procedure for dehydrogenative alkenylation (t = 24 h) with cyclohexane (16.8 mg, 0.2 mmol) and 1-chloro-3-vinylbenzene (277.2 mg, 10 equiv), obtained in 72% yield as a colorless liquid (31.6 mg, eluent: hexane, R_f = 0.6). ^{1}H NMR (400 MHz, $CDCl_3$) δ 7.35 – 7.31 (m, 1H), 7.21 – 7.13 (m, 3H), 6.28 (d, J = 16.0 Hz, 1H), 6.18 (dd, J = 16.0, 6.8 Hz, 1H), 2.17 – 2.07 (m, 1H), 1.83 – 1.64 (m, 5H), 1.33 – 1.16 (m, 5H). ^{13}C NMR (126 MHz, $CDCl_3$) δ 139.99, 138.41, 134.38, 129.63, 126.62, 126.04, 125.82, 124.20, 41.11, 32.81, 26.11, 25.97. HRMS EI $[M]^+$ Calculated $C_{14}H_{17}Cl$ 220.1013, found 220.1016.

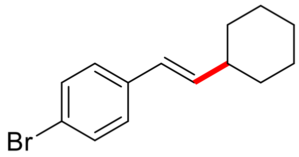

(*E*)-1-bromo-4-(2-cyclohexylvinyl)benzene, 50

Following the general procedure for dehydrogenative alkenylation (t = 24 h) with cyclohexane (16.8 mg, 0.2 mmol) and 1-bromo-4-vinylbenzene (366.1 mg, 10 equiv), obtained in 70% yield as a white solid (37.3 mg, eluent: hexane, R_f = 0.7). ^{1}H NMR (400 MHz, $CDCl_3$) δ 7.43 – 7.36 (m, 2H), 7.23 – 7.17 (m, 2H), 6.27 (d, J = 16.0 Hz, 1H), 6.16 (dd, J = 16.0, 6.8 Hz, 1H), 2.17 – 2.04 (m, 1H), 1.84 – 1.64 (m, 5H), 1.38 – 1.11 (m, 5H). ^{13}C NMR (126 MHz, $CDCl_3$) δ 137.68, 137.01, 131.47, 127.48, 126.13, 120.28, 41.12, 32.82, 26.11, 25.98. HRMS EI $[M]^+$ Calculated for $C_{14}H_{17}Br$ 264.0508, found 264.0507.

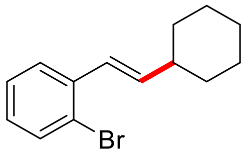

(*E*)-1-bromo-2-(2-cyclohexylvinyl)benzene, 51

Following the general procedure for dehydrogenative alkenylation (t = 24 h) with cyclohexane (16.8 mg, 0.2 mmol) and 1-bromo-2-vinylbenzene (366.1 mg, 10 equiv), obtained in 85% yield as a colorless liquid (45.1 mg, eluent: hexane, R_f = 0.6). ^{1}H NMR (400 MHz, $CDCl_3$) δ 7.53 – 7.47 (m, 2H), 7.25 – 7.20 (m, 1H), 7.07 – 7.01 (m, 1H), 6.67 (d, J = 16.0 Hz, 1H), 6.12 (dd, J = 16.0, 6.8 Hz, 1H), 2.25 – 2.13 (m, 1H), 1.88 – 1.64 (m, 5H), 1.38 – 1.18 (m, 5H). ^{13}C NMR (126 MHz, $CDCl_3$) δ 139.83, 137.80, 132.79, 128.03, 127.33, 126.72, 126.28, 123.34, 41.23, 32.82, 26.13, 25.97. HRMS EI $[M]^+$ Calculated $C_{14}H_{17}Br$ 264.0508, found 264.0511.

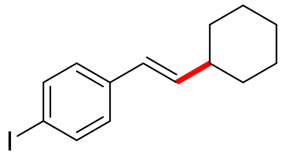

(*E*)-1-(2-cyclohexylvinyl)-4-iodobenzene, 52

Following the general procedure for dehydrogenative alkenylation (t = 24 h) with cyclohexane (16.8 mg, 0.2 mmol) and 1-iodo-4-vinylbenzene (460.1 mg, 10 equiv), obtained in 68% yield as a light yellow oil (42.3 mg, eluent: hexane, R_f = 0.7). ^{1}H NMR (400 MHz, $CDCl_3$) δ 7.64 – 7.55 (m, 2H), 7.13 – 7.03 (m, 2H), 6.26 (d, J = 16.0 Hz, 1H), 6.17 (dd, J = 16.0, 6.4 Hz, 1H), 2.18 – 2.05 (m, 1H), 1.85 – 1.63 (m, 5H), 1.39 – 1.10 (m, 5H). ^{13}C NMR (126 MHz, $CDCl_3$) δ 137.82, 137.60, 137.43, 127.78, 126.22, 91.59, 41.12, 32.80, 26.11, 25.98. HRMS EI $[M]^+$ Calculated for $C_{14}H_{17}I$ 312.0369, found 312.0371.

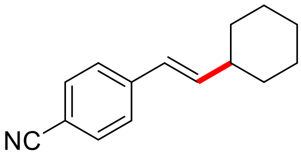

(*E*)-4-(2-cyclohexylvinyl)benzonitrile, 53

Following the general procedure for dehydrogenative alkenylation (t = 24 h) with cyclohexane (16.8 mg, 0.2 mmol) and 4-vinylbenzonitrile (258.3 mg, 10 equiv), obtained in 55% yield as a colorless liquid (23.4 mg, eluent: hexane/ethyl acetate = 20:1, R_f = 0.4). ^{1}H NMR (400 MHz, $CDCl_3$) δ 7.59 – 7.52 (m, 2H), 7.44 – 7.36 (m, 2H), 6.41 – 6.24 (m, 2H), 2.23 – 2.10 (m, 1H), 1.85 – 1.64 (m, 5H), 1.39 – 1.11 (m, 5H). ^{13}C NMR (126 MHz, $CDCl_3$) δ 142.61, 140.98, 132.27, 126.38, 125.98, 119.16, 109.83, 41.22, 32.60, 26.01, 25.87. HRMS EI $[M]^+$ Calculated for $C_{15}H_{17}N$ 211.1356, found 211.1354.

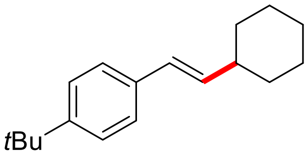

(*E*)-1-(*tert*-butyl)-4-(2-cyclohexylvinyl)benzene, 54

Following the general procedure for dehydrogenative alkenylation (t = 24 h) with cyclohexane (16.8 mg, 0.2 mmol) and 1-(*tert*-butyl)-4-vinylbenzene (320.5 mg, 10 equiv), obtained in 82% yield as a colorless liquid (39.9 mg, eluent: hexane, R_f = 0.8). ^{1}H NMR (400 MHz, $CDCl_3$) δ 7.34 – 7.26 (m, 4H), 6.32 (dd, J = 16.0, 0.8 Hz, 1H), 6.14 (dd, J = 16.0, 6.8 Hz, 1H), 2.18 – 2.05 (m, 1H), 1.84 – 1.64 (m, 5H), 1.37 – 1.11 (m, 5H), 1.31 (s, 9H). ^{13}C NMR (126 MHz, $CDCl_3$) δ 149.73, 136.14, 135.28, 126.87, 125.59, 125.35, 41.15, 34.45, 33.01, 31.31, 26.18, 26.05. HRMS EI $[M]^+$ Calculated for $C_{18}H_{26}$ 242.2029, found 242.2030.

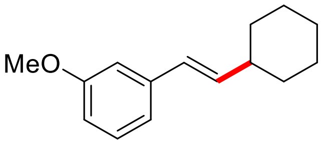

(*E*)-1-(2-cyclohexylvinyl)-3-methoxybenzene, 55

Following the general procedure for dehydrogenative alkenylation (t = 24 h) with cyclohexane (16.8 mg, 0.2 mmol) and 1-methoxy-3-vinylbenzene (268.4 mg, 10 equiv), obtained in 48% yield as a yellow liquid (20.6 mg, eluent: hexane, R_f = 0.3). ^{1}H NMR (400 MHz, $CDCl_3$) δ 7.24 – 7.17 (m, 1H), 6.98 – 6.92 (m, 1H), 6.91 – 6.87 (m, 1H), 6.78 – 6.71 (m, 1H), 6.32 (d, J = 16.0 Hz, 1H), 6.18 (dd, J = 16.0, 6.8 Hz, 1H), 3.81 (s, 3H), 2.20 – 2.05 (m, 1H), 1.88 – 1.62 (m, 5H), 1.32 – 1.20 (m, 5H). ^{13}C NMR (126 MHz, $CDCl_3$) δ 159.78, 139.56, 137.19, 129.39, 127.12, 118.64, 112.41, 111.23, 55.17, 41.12, 32.92, 26.16, 26.03. HRMS EI $[M]^+$ Calculated for $C_{15}H_{20}O$ 216.1509, found 216.1512.

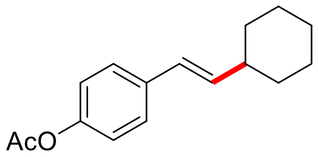

(*E*)-4-(2-cyclohexylvinyl)phenyl acetate, 56

Following the general procedure for dehydrogenative alkenylation (t = 24 h) with cyclohexane (16.8 mg, 0.2 mmol) and 4-vinylphenyl acetate (324.4 mg, 10 equiv), obtained in 89% yield as a white solid (43.5 mg, eluent: hexane/ethyl acetate = 20:1, R_f = 0.4). ^{1}H NMR (400 MHz, $CDCl_3$) δ 7.38 – 7.30 (m, 2H), 7.04 – 6.98 (m, 2H), 6.32 (d, J = 16.0 Hz, 1H), 6.13 (dd, J = 16.0, 6.8 Hz, 1H), 2.29 (s, 3H), 2.19 – 2.05 (m, 1H), 1.84 – 1.64 (m, 5H), 1.39 – 1.11 (m, 5H). ^{13}C NMR (126 MHz, $CDCl_3$) δ 169.50, 149.38, 137.12, 135.90, 126.78, 126.26, 121.47, 41.09, 32.90, 26.13, 26.00, 21.10. HRMS EI $[M]^+$ Calculated for $C_{16}H_{20}O_2$ 244.1458, found 244.1459.

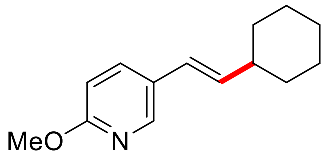

(*E*)-5-(2-cyclohexylvinyl)-2-methoxypyridine, 57

Following the general procedure for dehydrogenative alkenylation (t = 24 h) with cyclohexane (16.8 mg, 0.2 mmol) and 2-methoxy-5-vinylpyridine (270.3 mg, 10 equiv), obtained in 59% yield as a colorless liquid (25.6 mg, eluent: hexane/ethyl acetate = 20:1, R_f = 0.4). ^{1}H NMR (400 MHz, $CDCl_3$)

δ 8.05 (d, J = 2.4 Hz, 1H), 7.63 (dd, J = 8.8, 2.4 Hz, 1H), 6.68 (d, J = 8.8 Hz, 1H), 6.26 (d, J = 16.0 Hz, 1H), 6.05 (dd, J = 16.0, 6.8 Hz, 1H), 3.92 (s, 3H), 2.17 – 2.04 (m, 1H), 1.84 – 1.63 (m, 5H), 1.38 – 1.09 (m, 5H). ^{13}C NMR (126 MHz, $CDCl_3$) δ 163.06, 144.74, 136.34, 135.33, 127.21, 123.23, 110.68, 53.48, 41.14, 32.92, 26.12, 26.01. HRMS EI $[M]^+$ Calculated for $C_{14}H_{19}NO$ 217.1461, found 217.1462.

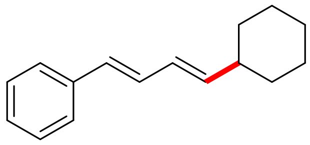

((1*E*,3*E*)-4-cyclohexylbuta-1,3-dien-1-yl)benzene, 58

Following the general procedure for dehydrogenative alkenylation (t = 24 h) with cyclohexane (16.8 mg, 0.2 mmol) and (*E*)-buta-1,3-dien-1-ylbenzene (260.4 mg, 10 equiv), obtained in 63% yield as a colorless liquid (26.6 mg, eluent: hexane, R_f = 0.7). ^{1}H NMR (400 MHz, $CDCl_3$) δ 7.38 – 7.27 (m, 4H), 7.21 – 7.16 (m, 1H), 6.74 (dd, J = 15.6, 10.4 Hz, 1H), 6.45 (d, J = 15.6 Hz, 1H), 6.23 – 6.13 (dddd, J = 15.2, 10.4, 1.2, 0.8 Hz, 1H), 5.79 (dd, J = 15.2, 6.8 Hz, 1H), 2.12 – 2.02 (m, 1H), 1.79 – 1.64 (m, 5H), 1.30 – 1.14 (m, 5H). ^{13}C NMR (126 MHz, $CDCl_3$) δ 141.73, 137.73, 130.03, 129.77, 128.52, 127.93, 127.00, 126.09, 40.92, 32.84, 26.15, 26.00. HRMS EI $[M]^+$ Calculated for $C_{16}H_{20}$ 212.1560, found 212.1560.

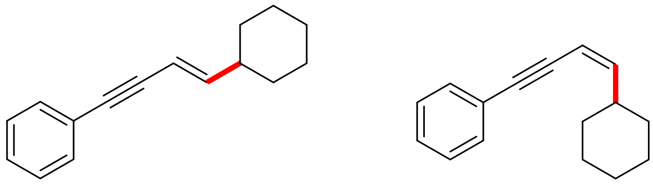

(*E*)-(4-cyclohexylbut-3-en-1-yn-1-yl)benzene (59, major)

(*Z*)-(4-cyclohexylbut-3-en-1-yn-1-yl)benzene (59, minor)

Following the general procedure for dehydrogenative alkenylation (t = 24 h) with cyclohexane (16.8 mg, 0.2 mmol) and but-3-en-1-yn-1-ylbenzene (256.3 mg, 10 equiv), obtained in 66% overall yield as a colorless liquid (27.9 mg in total, 10.3 mg of or *Z* isomer,17.6 mg of *E* isomer, *E*/*Z* = 1.7:1,

eluent: pentane, R_f = 0.65 for *Z* isomer, R_f = 0.6 for *E* isomer).

^{1}H NMR (400 MHz, $CDCl_3$, *Z* isomer) δ 7.47 – 7.40 (m, 2H), 7.35 – 7.27 (m, 3H), 5.82 (dd, J = 10.8, 9.2 Hz, 1H), 5.58 (dd, J = 10.8, 0.8 Hz, 1H), 2.76 – 2.61 (m, 1H), 1.81 – 1.63 (m, 5H), 1.36 – 1.15 (m, 5H). ^{13}C NMR (126 MHz, $CDCl_3$, *Z* isomer) δ 149.78, 131.38, 128.27, 127.92, 123.77, 106.99, 93.06, 86.55, 39.49, 32.35, 25.99, 25.73.

^{1}H NMR (400 MHz, $CDCl_3$, *E* isomer) δ 7.44 – 7.38 (m, 2H), 7.33 – 7.26 (m, 3H), 6.21 (dd, J = 16.0, 7.2 Hz, 1H), 5.66 (dd, J = 16.0, 1.6 Hz, 1H), 2.15 – 2.04 (m, 1H), 1.80 – 1.63 (m, 5H), 1.32 – 1.12 (m, 5H). ^{13}C NMR (126 MHz, $CDCl_3$, *E* isomer) δ 150.54, 131.39, 128.23, 127.81, 123.66, 107.19, 88.54, 88.09, 41.38, 32.28, 26.01, 25.82.

HRMS EI $[M]^+$ Calculated for $C_{16}H_{18}$ 210.1403, found 210.1404. (*Z* isomer).

HRMS EI $[M]^+$ Calculated for $C_{16}H_{18}$ 210.1403, found 210.1406. (*E* isomer).

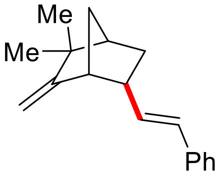

major

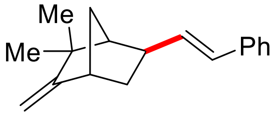

minor

(1*R*,4*R*,5*R*)-2,2-dimethyl-3-methylene-5-((*E*)-styryl)bicyclo[2.2.1]heptane (60, major)

(1*S*,4*S*,6*R*)-2,2-dimethyl-3-methylene-6-((*E*)-styryl)bicyclo[2.2.1]heptane (60, minor)

Following the general procedure for dehydrogenative alkenylation (t = 24 h) with (±)-camphene (27.2 mg, 0.2 mmol) and styrene (208.3 mg, 10 equiv), obtained in 57% yield as a colorless oil (27.3 mg, containing two regioisomers, major : minor = 1.4 : 1, d.r. > 20:1 for both isomers, eluent: hexane, R_f = 0.6). ^{1}H NMR (400 MHz, $CDCl_3$) δ 7.36 – 7.27 (m, 4H), 7.22 – 7.17 (m, 1H), 6.35 (d, J = 15.6 Hz, 1H), 6.20 (dd, J = 15.6, 7.6 Hz, 1H, minor), 6.15 (dd, J = 15.6, 8.4 Hz, 1H, major), 4.84 – 4.75 (m, 1H), 4.61 – 4.54 (m, 1H), 2.87 – 2.77 (m, 1H, minor), 2.76 – 2.71 (m, 1H, minor), 2.63 – 2.57 (m, 1H, major), 2.42 – 2.34 (m, 1H, major), 2.07 – 2.03 (m, 1H, major), 2.00 – 1.94 (m, 1H, major), 1.88 – 1.85 (m, 1H, minor), 1.72 – 1.65 (m, 2H, major), 1.64 – 1.55 (m, 3H, minor), 1.51 – 1.46 (m, 1H, major), 1.51 – 1.46 (m, 1H, minor), 1.16 (s, 3H, minor), 1.10 (s, 3H, major), 1.08 (s, 3H, minor), 1.07 (s, 3H, major). ^{13}C NMR (126 MHz, $CDCl_3$) δ 165.46, 165.19, 137.87, 137.79, 136.49, 135.24,

128.47, 128.26, 128.01, 127.61, 126.83, 126.78, 125.97, 125.92, 99.99, 99.81, 54.41, 53.32, 48.52, 47.24, 47.04, 44.62, 42.23, 38.47, 36.83, 34.85, 34.53, 32.29, 29.51, 29.38, 25.68, 25.55. HRMS EI $[M]^+$ Calculated for $C_{18}H_{22}$ 238.1716, found 238.1719.

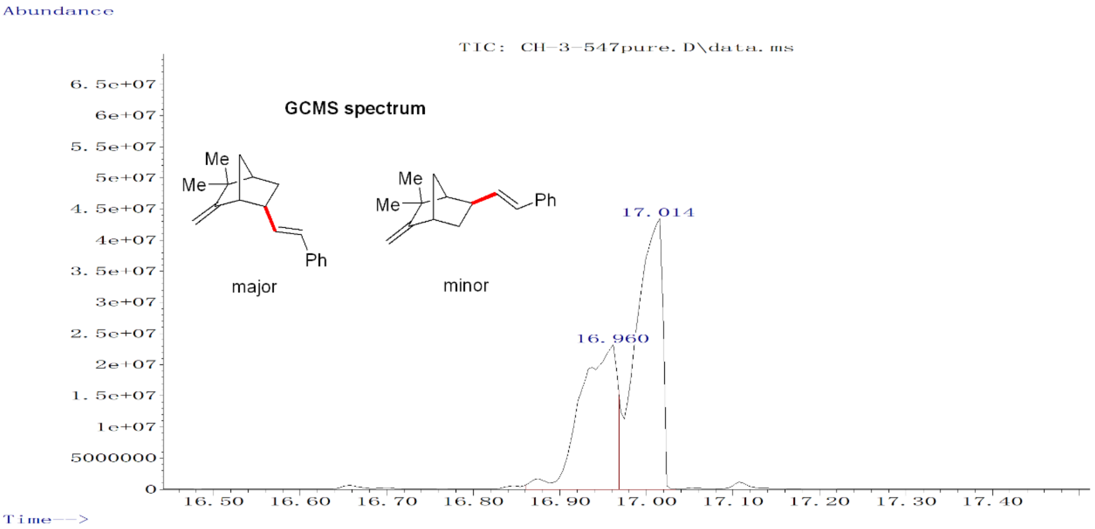

peak #	R.T. min	first scan	max scan	last scan	PK TY	peak height	corr. area	corr. % max.	% of total
1	16.960	3341	3366	3367	M2	23560288	671072223	73.20%	42.262%
2	17.014	3368	3379	3383	M	45285484	916810006	100.00%	57.738%

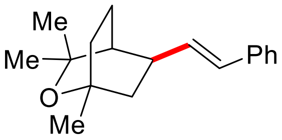

(1*R*,4*S*,5*R*)-1,3,3-trimethyl-5-((*E*)-styryl)-2-oxabicyclo[2.2.2]octane, 61

Following the general procedure for dehydrogenative alkenylation (t = 24 h) with eucalyptol (30.9 mg, 0.2 mmol) and styrene (208.3 mg, 10 equiv), obtained in 51% yield as a colorless oil (26.0 mg, eluent: hexane/ether = 100:1, R_f = 0.1). Two isomers (ratio 8.2 to 1) were detected in crude reaction mixture by GCMS, but only one isomer was obtained after column chromatography. ^{1}H NMR (400 MHz, $CDCl_3$) δ 7.39 – 7.35 (m 2H), 7.33 – 7.28 (m, 2H), 7.23 – 7.19 (m, 1H), 6.42 (d, J = 16.0 Hz, 1H), 6.31 (dd, J = 16.0, 6.8 Hz, 1H), 3.12 – 3.00 (m, 1H), 1.95 (ddd, J = 14.0, 10.8, 3.6 Hz, 1H), 1.90 – 1.75 (m, 2H), 1.73 – 1.65 (m, 1H), 1.50 – 1.40 (m, 3H), 1.33 (s, 3H), 1.31 (s, 3H), 1.10 (s, 3H). ^{13}C NMR (126 MHz, $CDCl_3$) δ 137.68, 135.28, 129.16, 128.54, 126.99, 125.99, 74.05, 70.40,

39.24, 37.84, 34.60, 31.68, 28.86, 28.66, 27.42, 16.76. HRMS EI $[M]^+$ Calculated for $C_{18}H_{24}O$ 256.1822, found 256.1823.

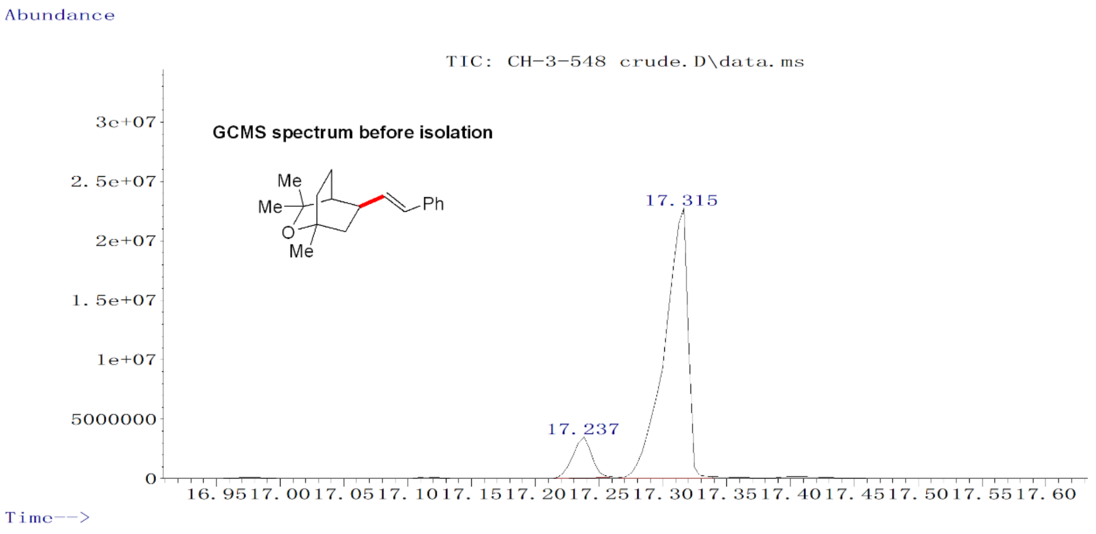

peak #	R.T. min	first scan	max scan	last scan	PK TY	peak height	corr. area	corr. % max.	% of total
1	17.237	3428	3433	3438	M	3497106	35480604	12.13%	10.817%
2	17.315	3440	3452	3457	M	23816511	292523102	100.00%	89.183%

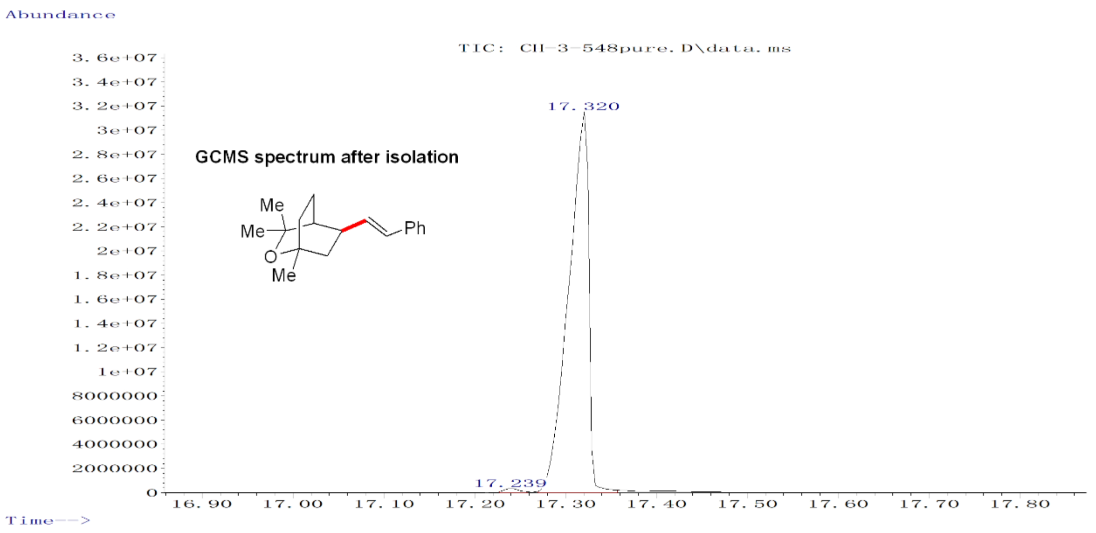

peak #	R.T. min	first scan	max scan	last scan	PK TY	peak height	corr. area	corr. % max.	% of total
1	17.239	3430	3433	3438	M	338328	3621927	0.74%	0.739%
2	17.320	3441	3453	3462	M	31686463	486282182	100.00%	99.261%

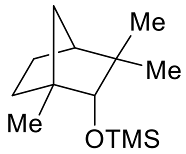

Trimethyl(((1*S*,2*R*,4*S*)-1,3,3-trimethylbicyclo[2.2.1]heptan-2-yl)oxy)silane

To a solution of (+)-Fenchol [(1*R*)-*endo*-(+)-Fenchyl alcohol, CAS: 2217-02-9] (2.0 mmol) in 10 mL dry dichloromethane, hexamethyldisilazane (HMDS, 1.2 mmol) was added followed by a catalytic amount of ammonium thiocyanate (0.1 mmol) at 0 °C. The reaction mixture was stirred and monitored by thin-layer chromatography (TLC). After completion of the reaction, water (6 mL) was added, and the reaction mixture was extracted with dichloromethane (3 x 10 mL). Evaporation of the solvent gave product that was purified by column chromatography on neutral alumina (eluent: hexane, colorless liquid, 443.8 mg, 98%). ^{1}H NMR (400 MHz, $CDCl_3$) δ 3.20 (d, J = 1.6 Hz, 1H), 1.79 – 1.69 (m, 1H), 1.68 – 1.58 (m, 2H), 1.45 – 1.29 (m, 2H), 1.07 (dd, J = 10.0, 1.6 Hz, 1H), 0.99 (s, 3H), 0.94 (s, 3H), 0.91 – 0.84 (m, 1H), 0.77 (s, 3H), 0.10 – 0.04 (m, 9H). ^{13}C NMR (126 MHz, $CDCl_3$) δ 85.35, 49.40, 48.38, 40.90, 39.26, 30.47, 26.24, 25.43, 21.05, 19.89, 0.42. HRMS EI $[M]^+$ Calculated for $C_{13}H_{26}OSi$ 226.1747, found 226.1744.

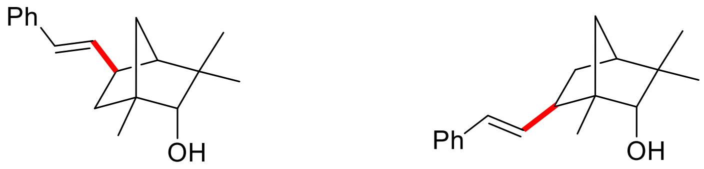

major minor 1

(1*S*,2*R*,4*R*,5*S*)-1,3,3-trimethyl-5-((*E*)-styryl)bicyclo[2.2.1]heptan-2-ol (62, major)

(1*R*,2*S*,4*S*,6*R*)-1,3,3-trimethyl-6-((*E*)-styryl)bicyclo[2.2.1]heptan-2-ol (62, minor 1)

Following the general procedure for dehydrogenative alkenylation (t = 24 h) with trimethyl(((1*S*,2*R*,4*S*)-1,3,3-trimethylbicyclo[2.2.1]heptan-2-yl)oxy)silane (45.3 mg, 0.2 mmol) and styrene (208.3 mg, 10 equiv), the product, along with (*E*)-but-1-ene-1,3-diyldibenzene, were isolated from column chromatography (eluent: hexane/Et_3N = 100:1, R_f = 0.6-0.7). The crude mixture was dissolved in 10 mL THF and then treated with tetra-*n*-butylammonium fluoride (0.2 mmol) overnight. Finally, column chromatography gave the title compounds along with other minor isomers in 47% yield (containing all isomers, 74% selectivity for major, 13% selectivity for minor

1, <5% selectivity for other isomers, 24.2 mg, eluent: hexane/ethyl acetate = 10:1, R_f = 0.2). ^{1}H NMR (400 MHz, $CDCl_3$, major) δ 7.37 – 7.27 (m, 4H), 7.21 – 7.16 (m, 1H), 6.33 (d, J = 16.0 Hz, 1H), 6.20 (dd, J = 16.0, 7.2 Hz, 1H), 3.30 (d, J = 1.6 Hz, 1H), 2.80 – 2.71 (m, 1H), 2.02 (ddd, J = 13.2, 8.8, 1.6 Hz, 1H), 1.68 – 1.65 (m, 1H), 1.43 – 1.40 (m, 2H), 1.28 – 1.25 (m, 1H), 1.13 (s, 3H), 1.04 (s, 3H), 0.97 (s, 3H). ^{13}C NMR (126 MHz, $CDCl_3$) δ 137.88, 136.66, 134.58, 129.02, 128.46, 127.92, 126.77, 125.98, 125.91, 85.95, 85.66, 85.37, 84.50, 53.99, 52.48, 49.38, 47.38, 40.14, 39.88, 39.02, 38.54, 37.85, 37.60, 34.70, 33.23, 30.68, 20.02, 19.36, 17.52. HRMS EI $[M]^+$ Calculated for $C_{18}H_{24}O$ 256.1822, found 256.1820.

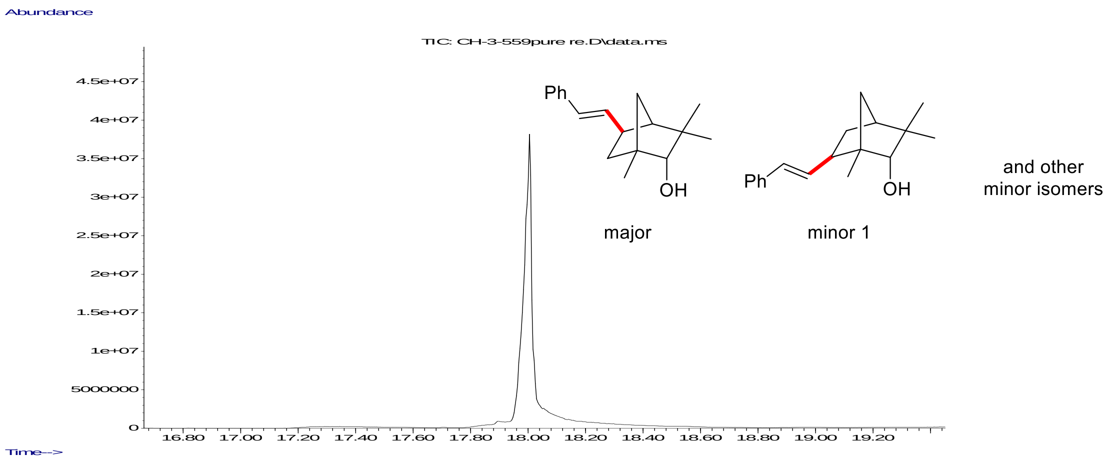

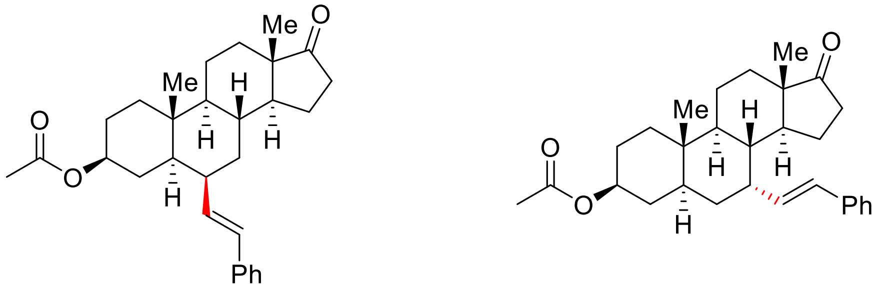

major minor

(3*S*,5*R*,6*S*,8*R*,9*S*,10*R*,13*S*,14*S*)-10,13-dimethyl-17-oxo-6-((*E*)-styryl)hexadecahydro-1*H*-cyclopenta[*a*]phenanthren-3-yl acetate (63, major)

(3*S*,5*S*,7*S*,8*R*,9*S*,10*S*,13*S*,14*S*)-10,13-dimethyl-17-oxo-7-((*E*)-styryl)hexadecahydro-1*H*-cyclopenta[*a*]phenanthren-3-yl acetate (63, minor)

Following the general procedure for dehydrogenative alkenylation (t = 24 h) with *trans*-androsterone acetate (66.5 mg, 0.2 mmol) and styrene (208.3 mg, 10 equiv), obtained in 64% yield as a colorless oil (55.6 mg, containing two isomers, major : minor = 2.5:1, eluent: hexane : ethyl acetate = 10/1, R_f = 0.2). ^{1}H NMR (400 MHz, $CDCl_3$) δ 7.36 – 7.27 (m, 4H), 7.22 – 7.19 (m, 1H), 6.35 (d, J = 16.0 Hz, 1H, minor), 6.34 (d, J = 15.6 Hz, 1H, major), 6.14 (dd, J = 16.0, 8.8 Hz, 1H, minor), 5.91 (dd, J = 15.6, 9.2 Hz, 1H, major), 4.74 – 4.62 (m, 1H), 2.44 (dd, J = 19.6, 8.4 Hz, 1H, major), 2.32 (dd, J = 19.6, 8.0 Hz, 1H, minor), 2.15 – 2.03 (m, 2H), 2.02 (s, 3H, minor), 1.98 (s, 3H, major), 1.94 – 1.89 (m, 1H), 1.84 – 1.25 (m, 14H), 1.15 – 1.04 (m, 2H), 0.93 (s, 3H, major), 0.89 (s, 3H, minor), 0.89 (s, 3H, minor), 0.88 (s, 3H, major), 0.84 – 0.76 (m, 1H). ^{13}C NMR (126 MHz, $CDCl_3$, major) δ 220.96, 170.49, 137.33, 134.51, 129.99, 128.51, 127.05, 125.98, 73.47, 54.02, 51.23, 48.75, 47.72, 42.03, 38.33, 36.79, 35.82, 35.71, 34.27, 31.48, 31.29, 27.29, 21.70, 21.37, 20.45, 13.80, 13.06. ^{13}C NMR (126 MHz, $CDCl_3$, minor) δ 221.39, 170.63, 137.74, 136.96, 128.60, 128.56, 126.94, 125.81, 73.26, 53.39, 51.81, 48.48, 47.20, 43.47, 40.37, 37.36, 36.86, 36.07, 35.19, 33.60, 31.53, 27.46, 25.44, 21.40, 20.74, 14.13, 12.34. HRMS APCI $[M+H]^+$ Calculated for $C_{29}H_{39}O_3$ 435.2894, found 435.2895.

Regiochemistry assignments were made by NMR analysis and comparison with standard samples. Standard samples were synthesized according to the following procedure reported by the Alexanian group.[28]

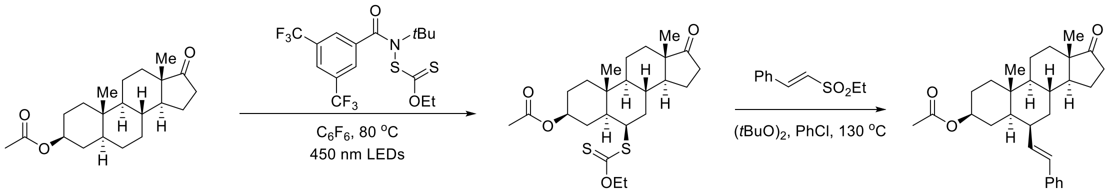

A 1 dram vial with a stir bar was charged with xanthylamide (3 equiv) and *trans*-androsterone acetate (1 equiv) in the dark (overhead laboratory lights turned off), fitted with a PTFE lined screw cap, and taken into the glovebox. The xanthylamide was dissolved in C_6F_6 (1 M), and the resulting solution was sealed with Teflon tape and removed from the glovebox. The vial was placed directly on a stir plate maintained at 80 °C and irradiated with a Kessil Blue KSH150B 34W LED Grow Light from the side (2 cm away) with the apparatus covered by aluminum foil until completion (24 h). The reaction was then concentrated in vacuo. The crude residue was purified by flash column

chromatography to afford the C6-xanthylated product (26% yield).

To a solution of the xanthylated product and styryl ethyl sulfone (3 equiv) dissolved in PhCl (0.05 M) and stirring at 130 °C, *tert*-butyl peroxide (1 equiv) was added. Four more *tert*-butyl peroxide (0.5 equiv) additions were added in the following 12 hours. After the last addition, the reaction was left stirring overnight. The resulting dark brown mixture was concentrated, and the product was isolated by flash column chromatography to yield the alkenylated product (45% yield).

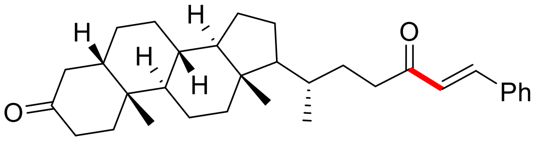

(5*S*,8*S*,9*R*,10*R*,13*S*,14*R*)-10,13-dimethyl-17-((*S*,*E*)-5-oxo-7-phenylhept-6-en-2-yl)hexadecahydro-3*H*-cyclopenta[*a*]phenanthren-3-one, 64

Following the general procedure for dehydrogenative alkenylation (t = 24 h) with (4*S*)-4-((5*S*,8*S*,9*R*,10*R*,13*S*,14*R*)-10,13-dimethyl-3-oxohexadecahydro-1*H*-cyclopenta[*a*]phenanthren-17-yl)pentanal (71.7 mg, 0.2 mmol) and styrene (208.3 mg, 10 equiv), obtained in 75% yield as a colorless oil (68.8 mg, eluent: hexane : ethyl acetate = 10/1, R_f = 0.15). ^{1}H NMR (400 MHz, $CDCl_3$) δ 7.65 – 7.43 (m, 3H), 7.45 – 7.33 (m, 3H), 6.74 (d, J = 16.4 Hz, 1H), 2.74 – 2.64 (m, 1H), 2.63 – 2.53 (m, 1H), 2.40 – 2.27 (m, 2H), 2.19 – 2.12 (m, 1H), 2.05 – 2.00 (m, 3H), 1.91 – 1.81 (m, 4H), 1.64 – 1.56 (m, 2H), 1.44 – 1.11 (m, 14H), 1.02 (s, 3H), 0.97 (d, J = 6.4 Hz, 3H), 0.70 (s, 3H). ^{13}C NMR (126 MHz, $CDCl_3$) δ 213.43, 200.99, 142.25, 134.60, 130.37, 128.93, 128.23, 126.23, 56.45, 56.08, 44.32, 42.80, 42.36, 40.74, 40.07, 37.94, 37.21, 37.01, 35.53, 35.48, 34.88, 30.37, 28.21, 26.61, 25.76, 24.17, 22.64, 21.20, 18.55, 12.09. HRMS APCI $[M+H]^+$ Calculated for $C_{32}H_{45}O_2$ 461.3414, found 461.3408.

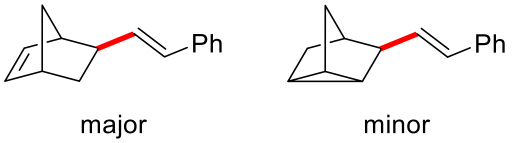

5-((*E*)-styryl)bicyclo[2.2.1]hept-2-ene (major)

3-((*E*)-styryl)tricyclo[2.2.1.02,6]heptane (minor)

Following the general procedure for dehydrogenative alkenylation (t = 24 h) with norbornene (18.8 mg, 0.2 mmol) and styrene (208.3 mg, 10 equiv), obtained in 46% yield as a colorless liquid (18.2 mg, containing two isomers, major : minor = 1.3 : 1, eluent: hexane, R_f = 0.55). ^{1}H NMR (400 MHz, $CDCl_3$) δ 7.37 – 7.26 (m, 4H), 7.22 – 7.16 (m, 1H), 6.44 (d, J = 15.6 Hz, 1H, minor), 6.41 (dd, J = 15.6, 5.6 Hz, 1H, major), 6.27 – 6.09 (m, 3H, major), 6.27 – 6.09 (m, 1H, minor), 2.91 (s, 1H, major), 2.70 (s, 1H, major), 2.38 – 2.18 (m, 1H), 1.83 (s, 1H, minor), 1.53 – 1.00 (m, 4H, major), 1.53 – 1.00 (m, 6H, minor). ^{13}C NMR (126 MHz, $CDCl_3$) δ 137.91, 137.90, 137.20, 136.57, 136.39, 136.01, 131.51, 129.75, 128.64, 128.48, 128.44, 126.77, 125.95, 125.91, 48.50, 47.67, 45.60, 42.26, 42.03, 34.78, 34.22, 33.12, 29.37, 14.63, 11.42, 9.68. HRMS EI $[M]^+$ Calculated for $C_{15}H_{16}$ 196.1247, found 196.1246. Spectral data is in agreement with the literature.[29]

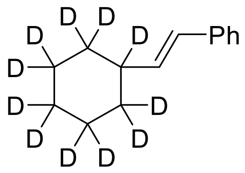

(*E*)-(2-(cyclohexyl-*d*$_{11}$)vinyl)benzene

Following the general procedure for dehydrogenative alkenylation (t = 12 h) with cyclohexane-d_{12} (19.2 mg, 0.2 mmol) and styrene (208.3 mg, 10 equiv), obtained in 53% yield as a colorless liquid (20.9 mg, eluent: hexane, R_f = 0.7). ^{1}H NMR (400 MHz, $CDCl_3$) δ 7.40 – 7.27 (m, 4H), 7.21 – 7.15 (m, 1H), 6.34 (d, J = 16.0 Hz, 1H), 6.17 (d, J = 16.0 Hz, 1H). ^{13}C NMR (126 MHz, $CDCl_3$) δ 138.07, 136.88, 128.43, 127.18, 126.69, 125.91. HRMS EI $[M]^+$ Calculated for $C_{14}H_7D_{11}$ 197.2093, found 197.2094.

Supplementary Note 2

^{1}H, ^{13}C, ^{19}F, COSY, NOESY, HMQC and HMBC spectra

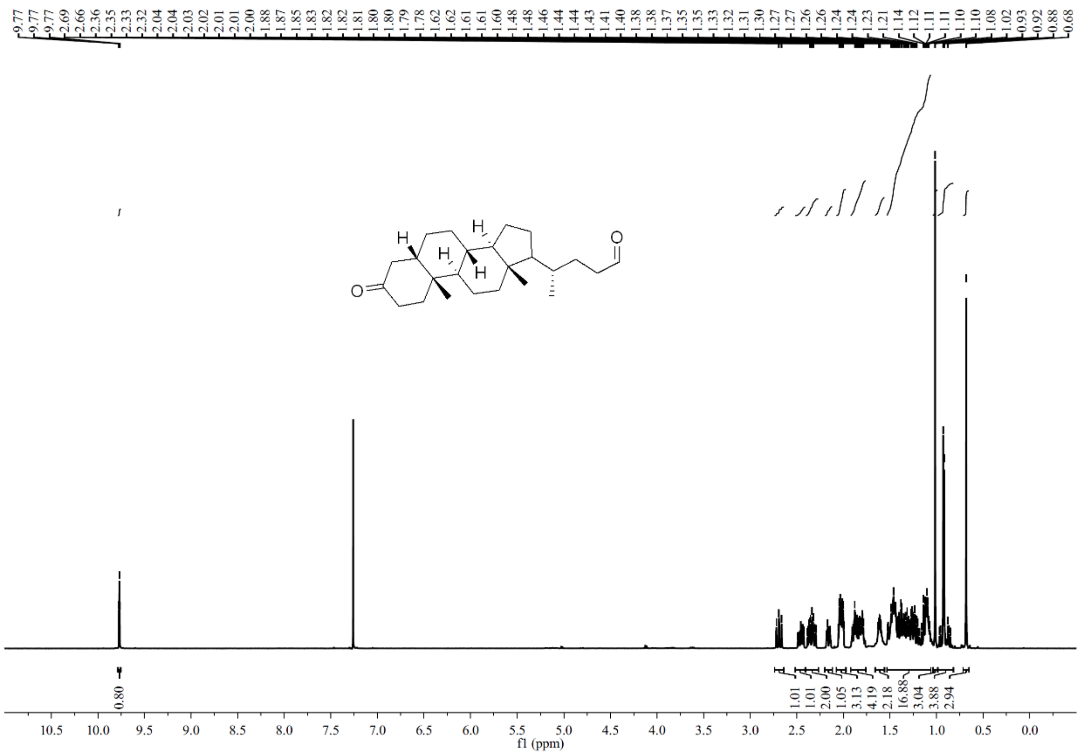

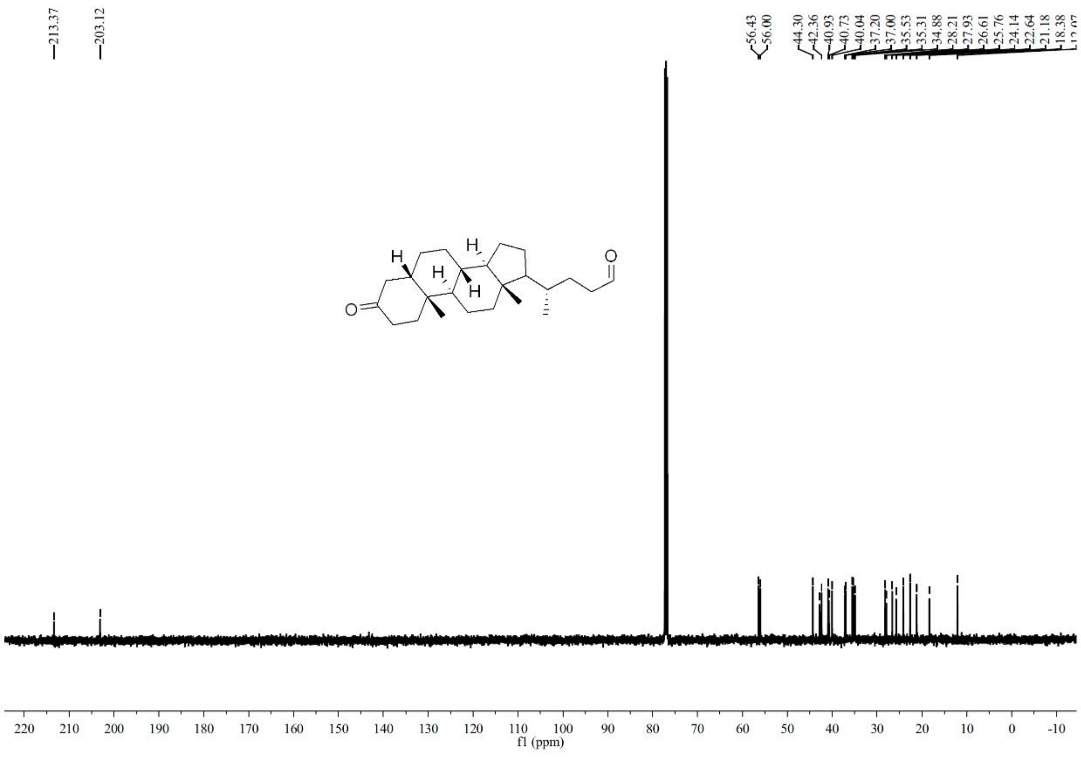

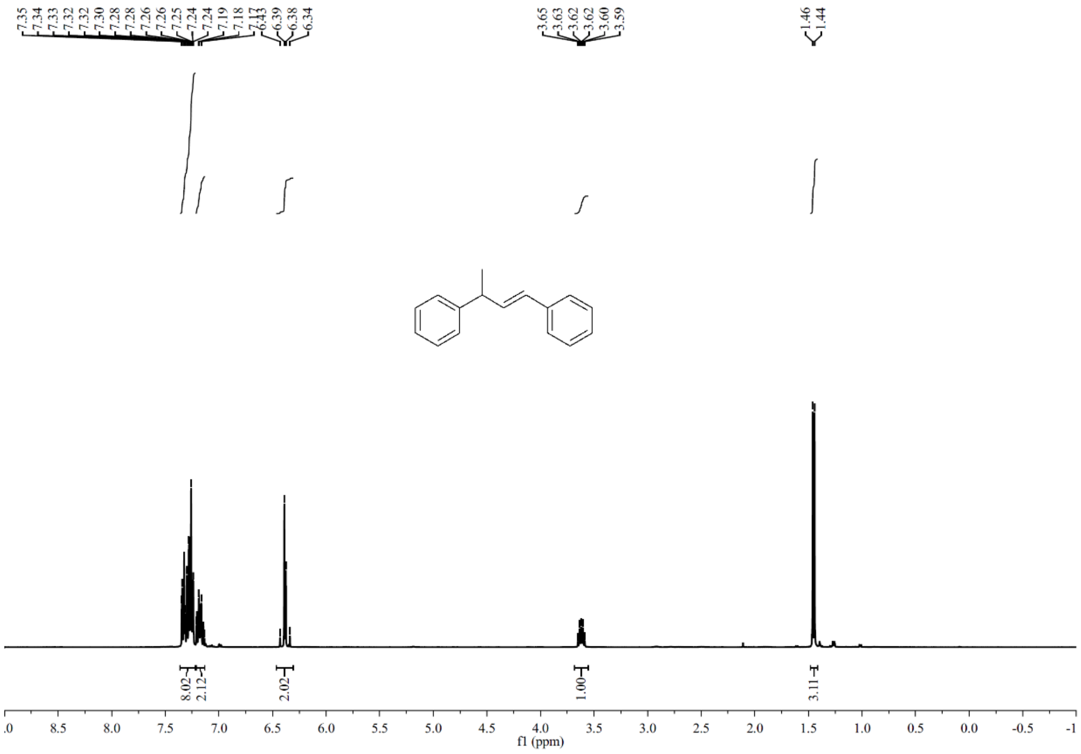

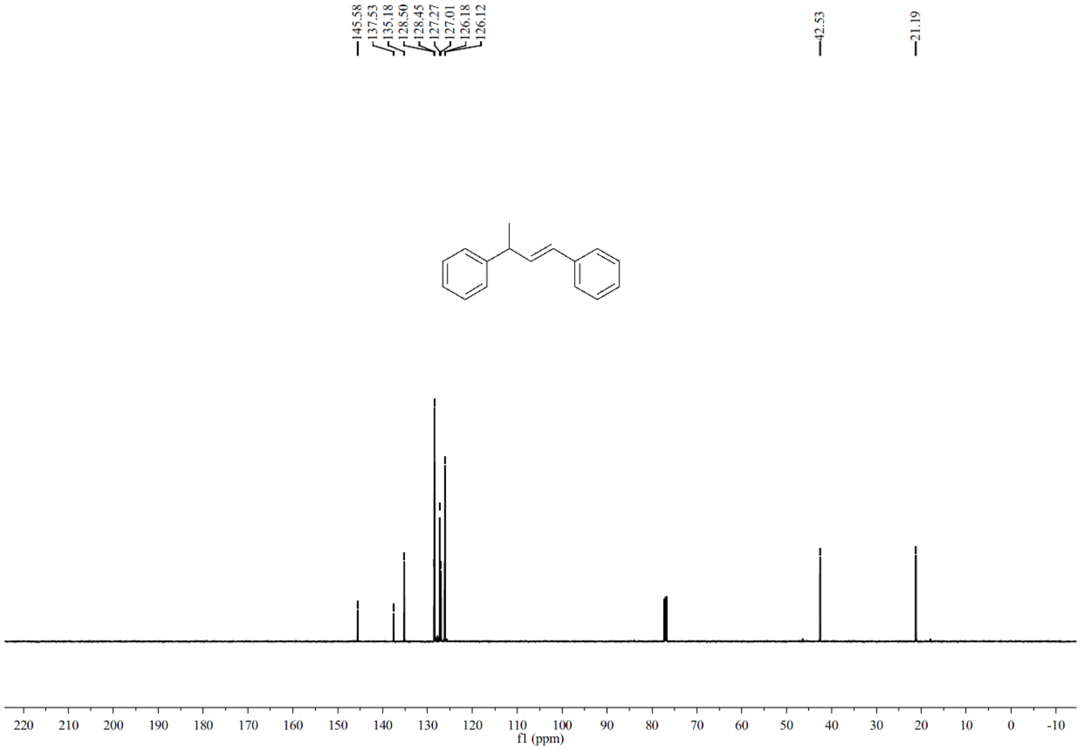

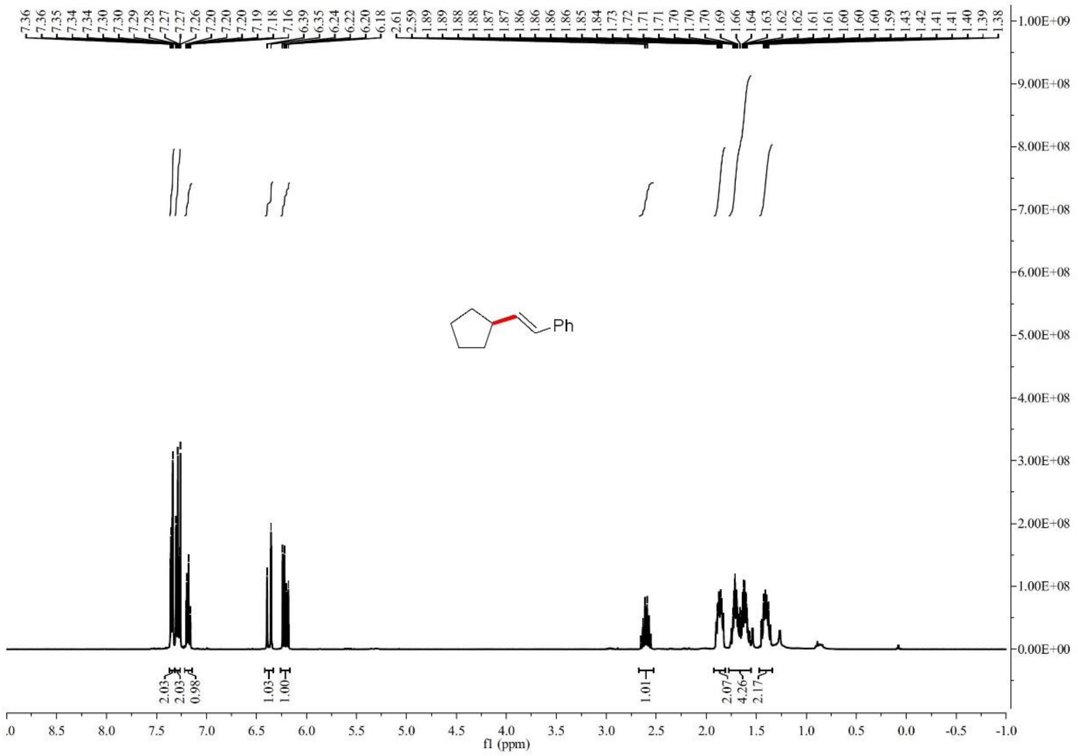

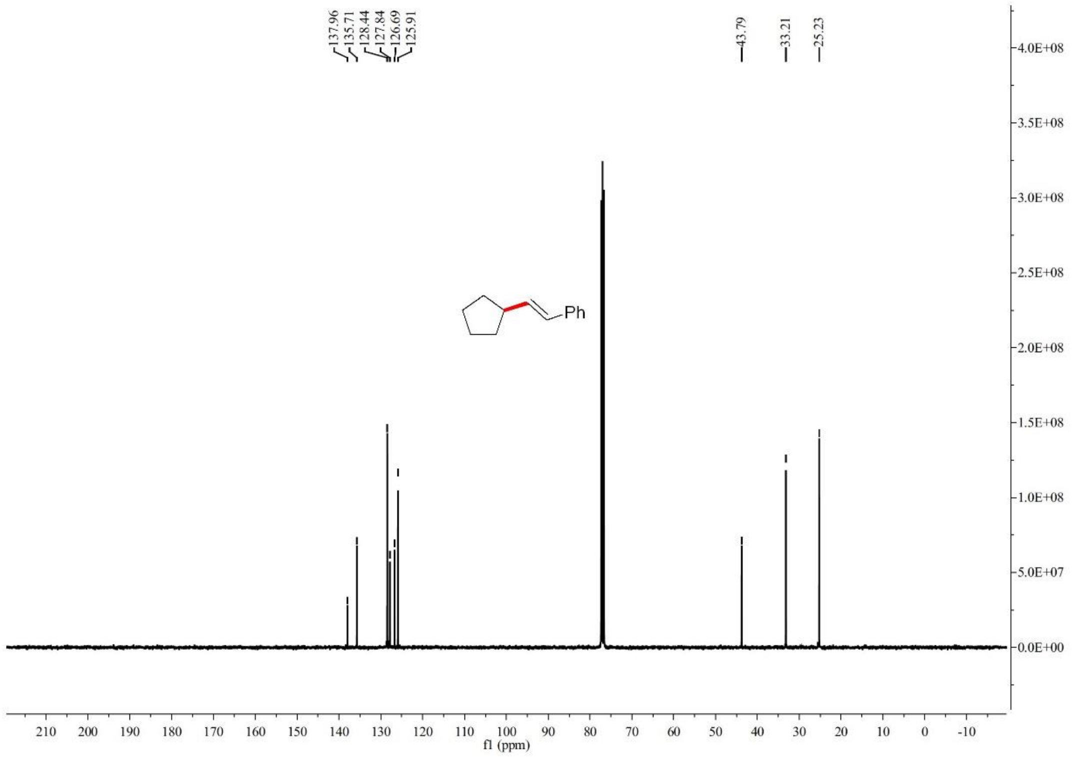

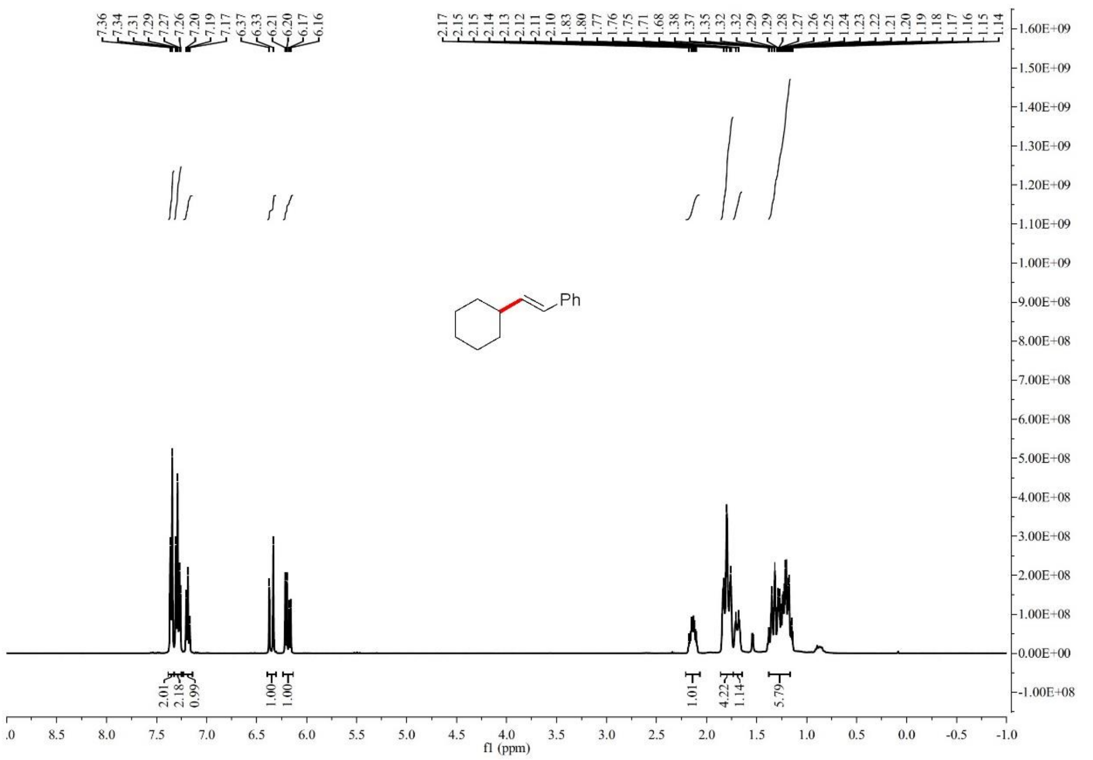
7.36
7.34
7.31
7.29
7.27
7.26
7.20
7.19
7.17
6.37
6.33
6.21
6.20
6.17
6.16
2.17
2.15
2.15
2.14
2.13
2.12
2.11
2.10
1.83
1.80
1.77
1.76
1.75
1.71
1.68
1.38
1.37
1.35
1.32
1.32
1.29
1.29
1.28
1.27
1.26
1.25
1.24
1.23
1.22
1.21
1.20
1.19
1.18
1.17
1.16
1.15
1.14
Ph
2.01
2.18
0.99
1.00
1.00
1.01
4.22
1.14
5.79
fl (ppm)

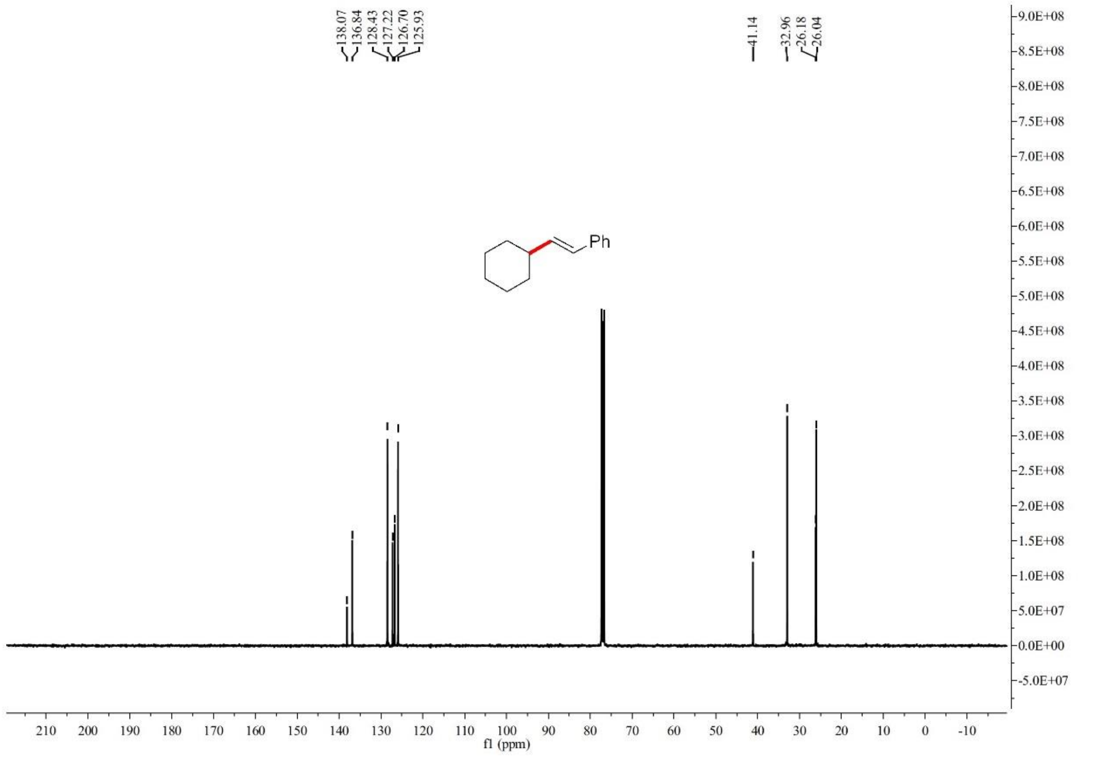
138.07
136.84
128.43
127.22
126.70
125.93
41.14
32.96
26.18
26.04
Ph
fl (ppm)

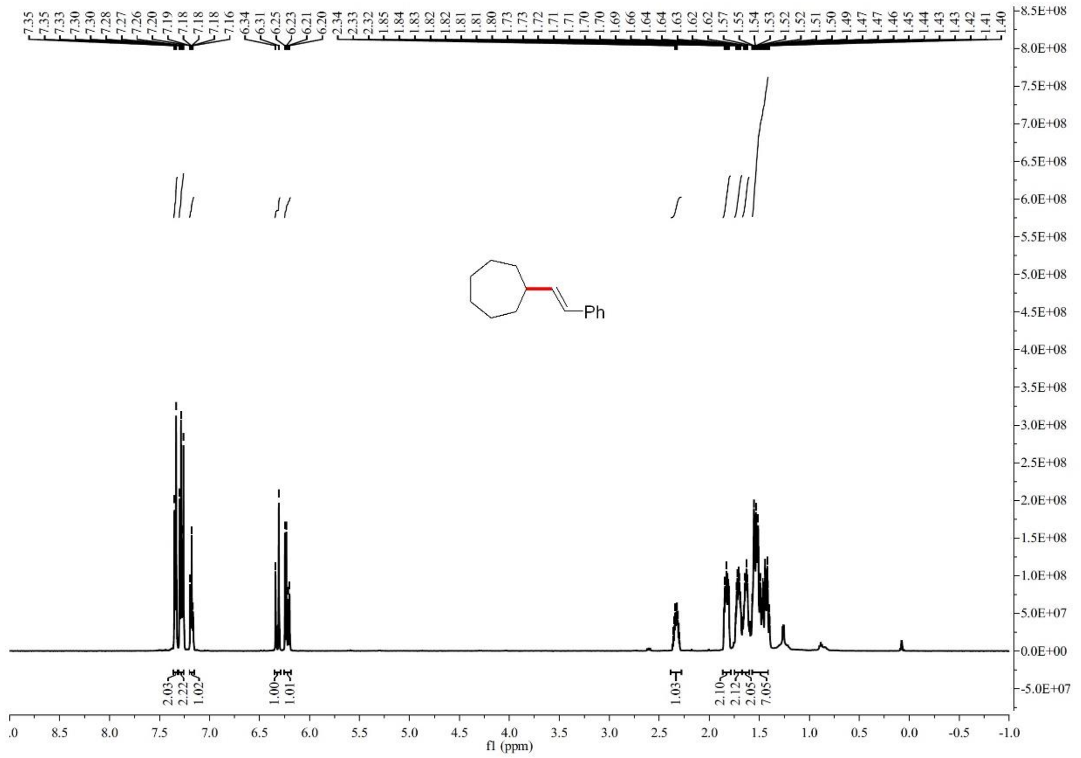
Ph
f1 (ppm)

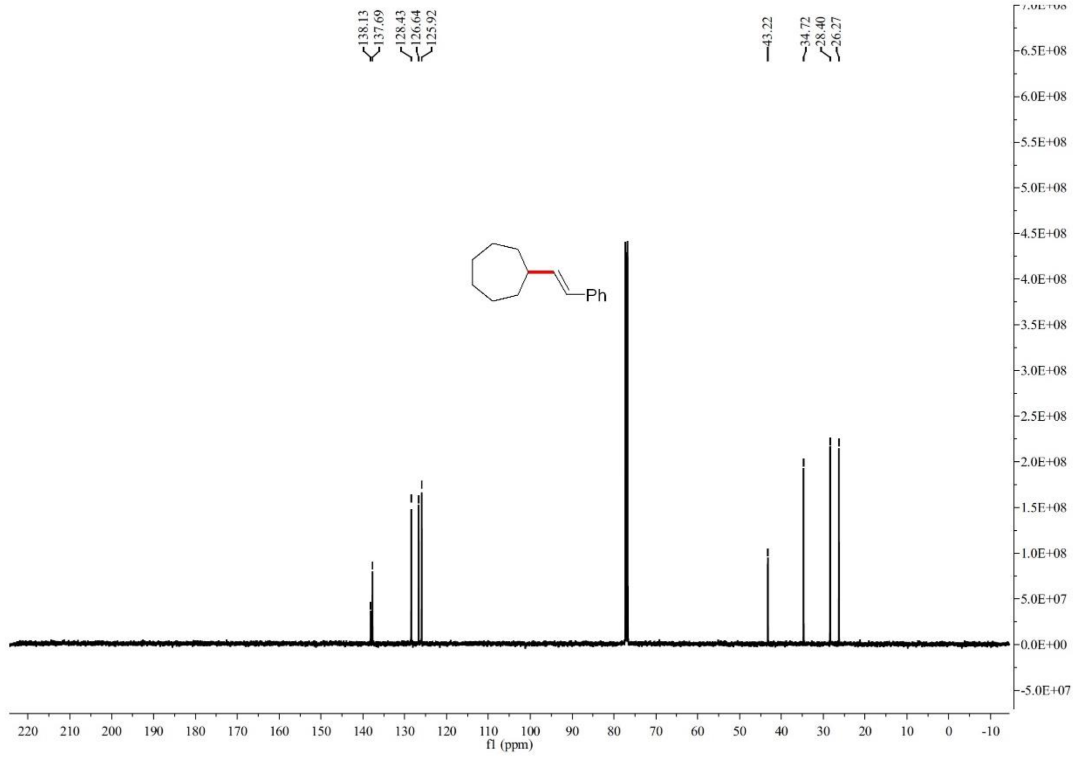
Ph
f1 (ppm)

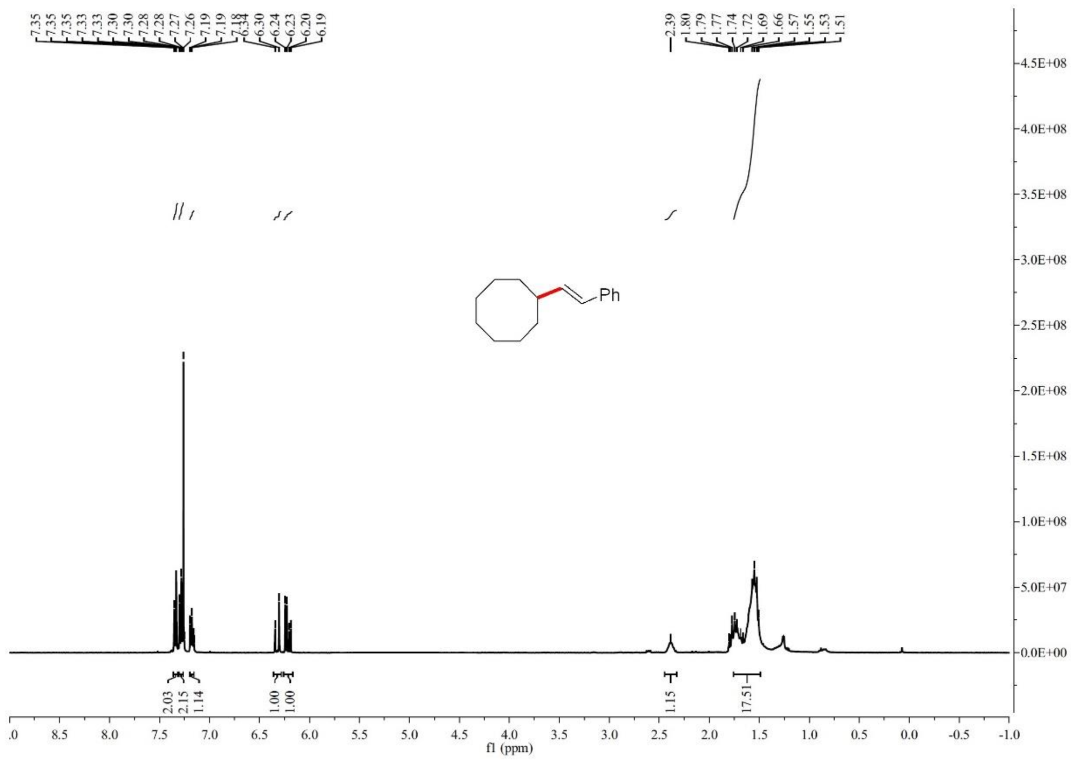

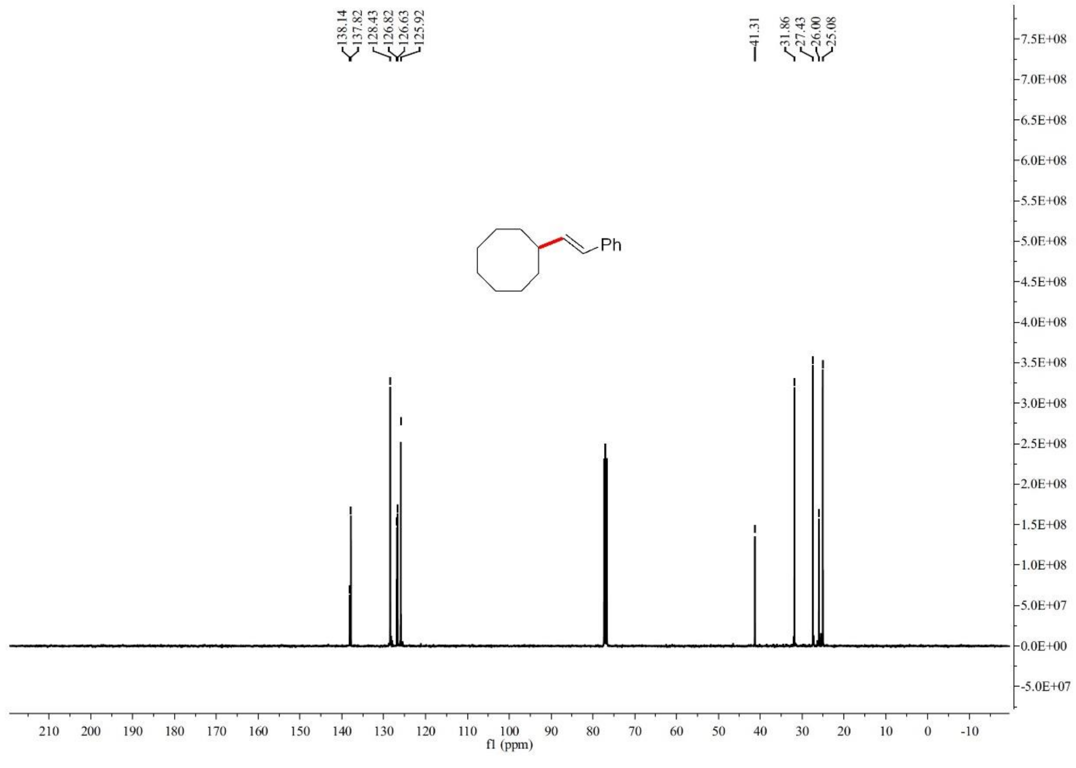

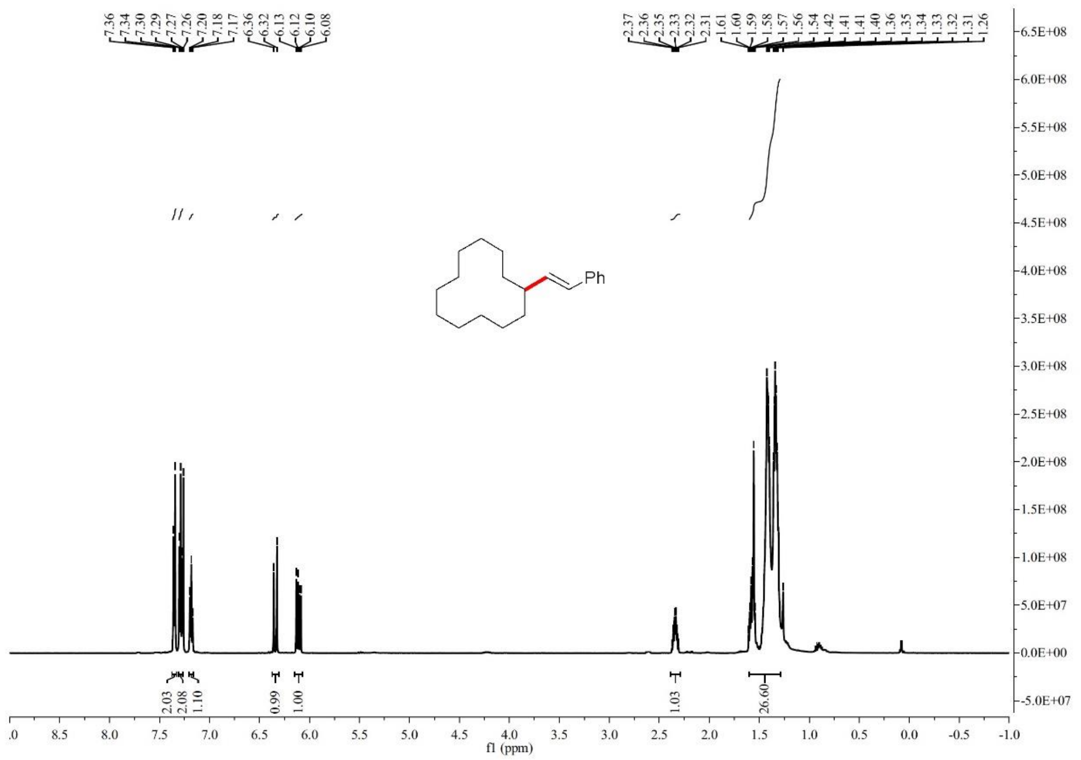
7.36
7.34
7.30
7.29
7.27
7.26
7.20
7.18
7.17
6.36
6.32
6.13
6.12
6.10
6.08
2.37
2.36
2.35
2.33
2.32
2.31
1.61
1.60
1.59
1.58
1.57
1.56
1.54
1.42
1.41
1.41
1.40
1.36
1.35
1.34
1.33
1.32
1.31
1.26
Ph
2.03
2.08
1.10
0.99
1.00
1.03
26.60
fl (ppm)

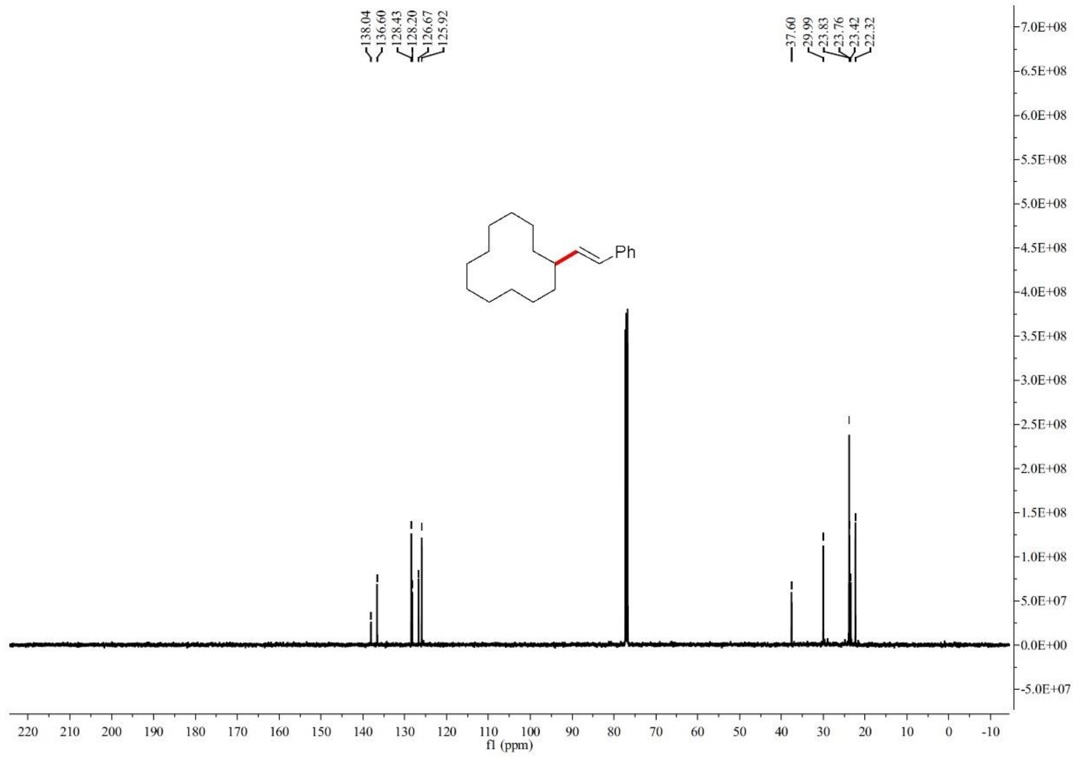
138.04
136.60
128.43
128.20
126.67
125.92
37.60
29.99
23.83
23.76
23.42
22.32
Ph
fl (ppm)

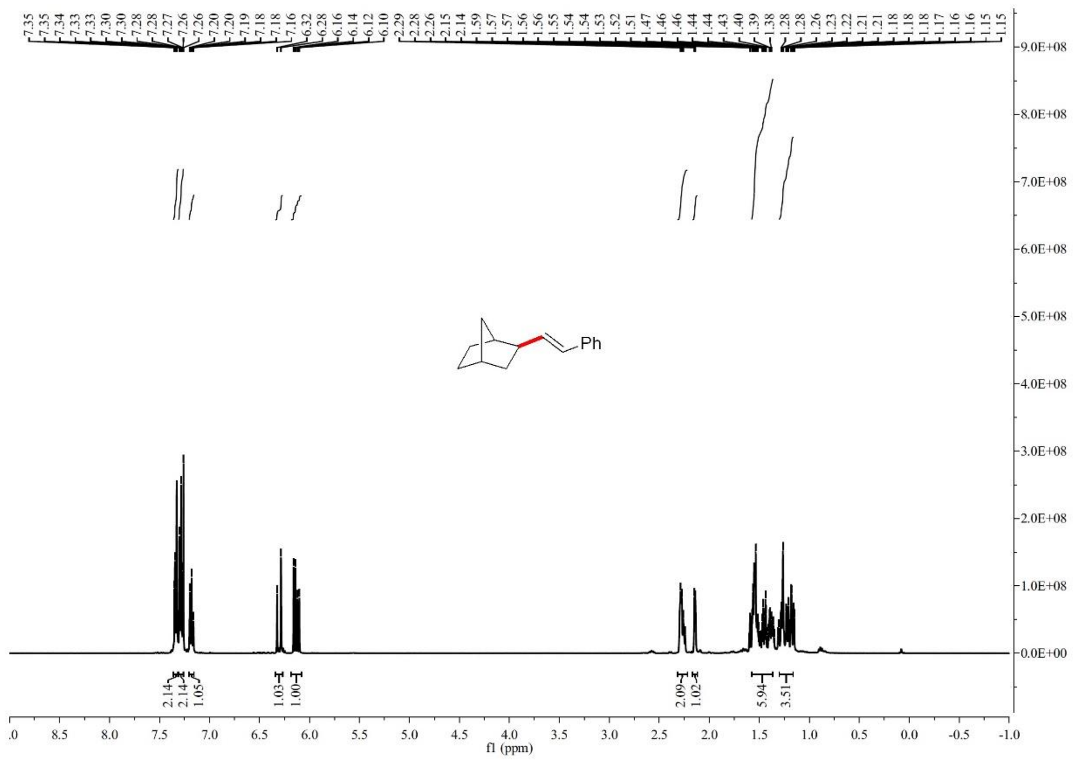

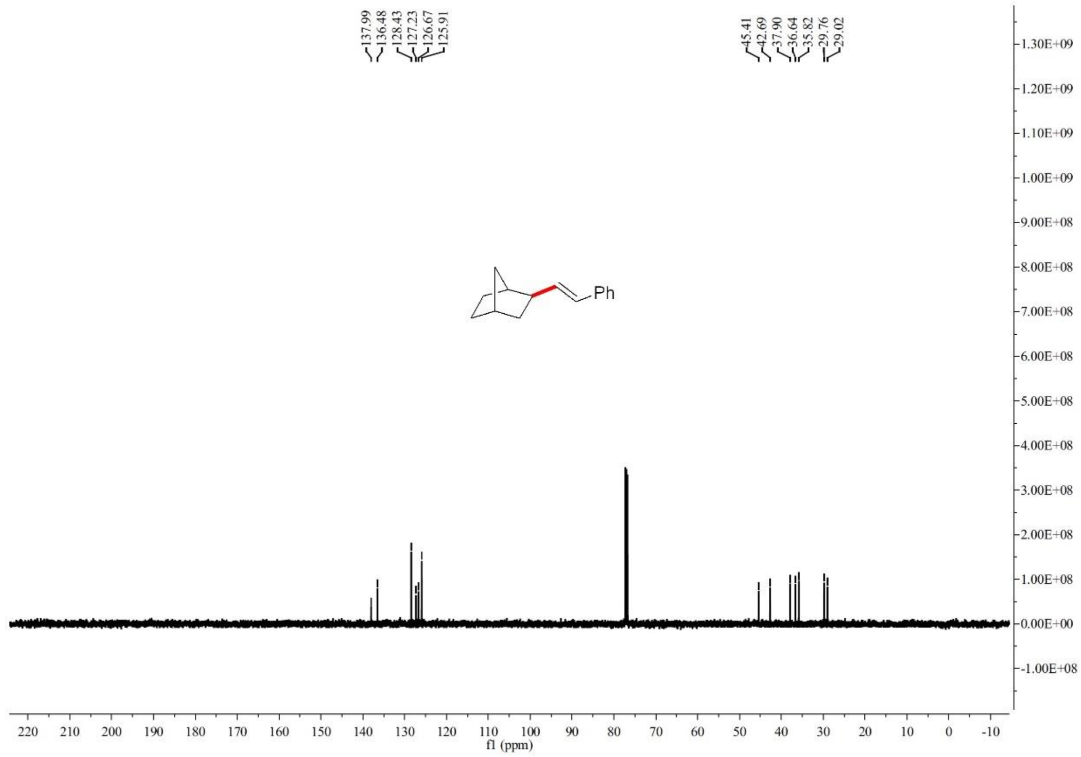

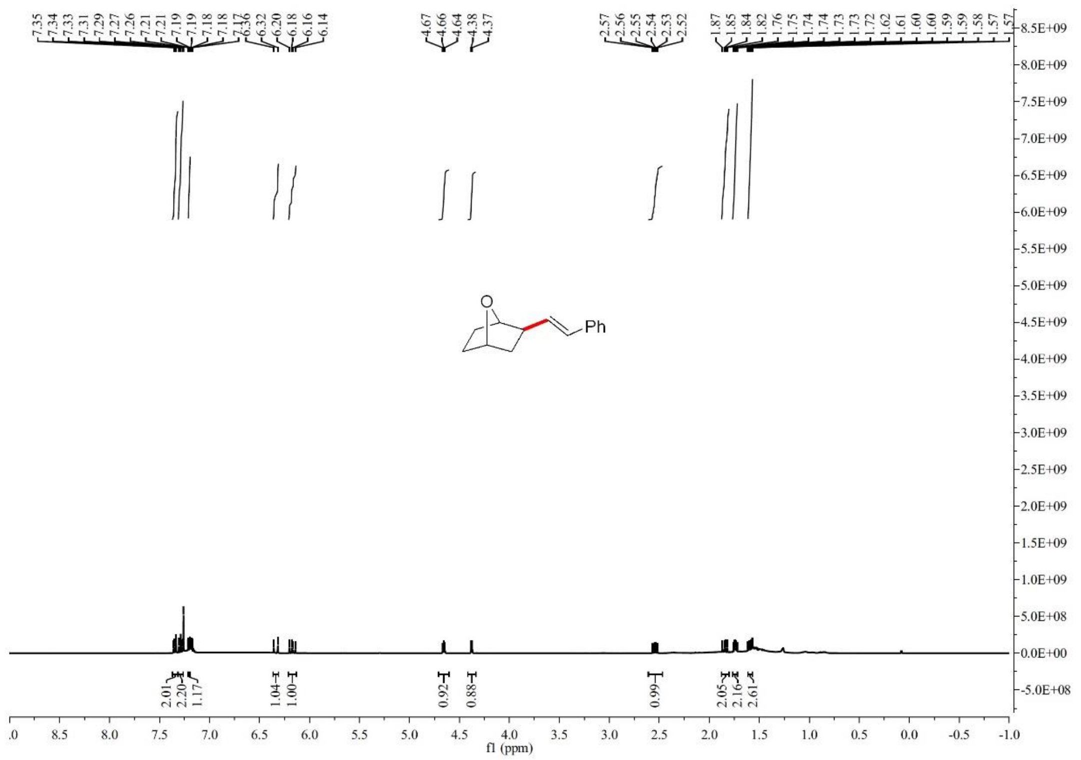

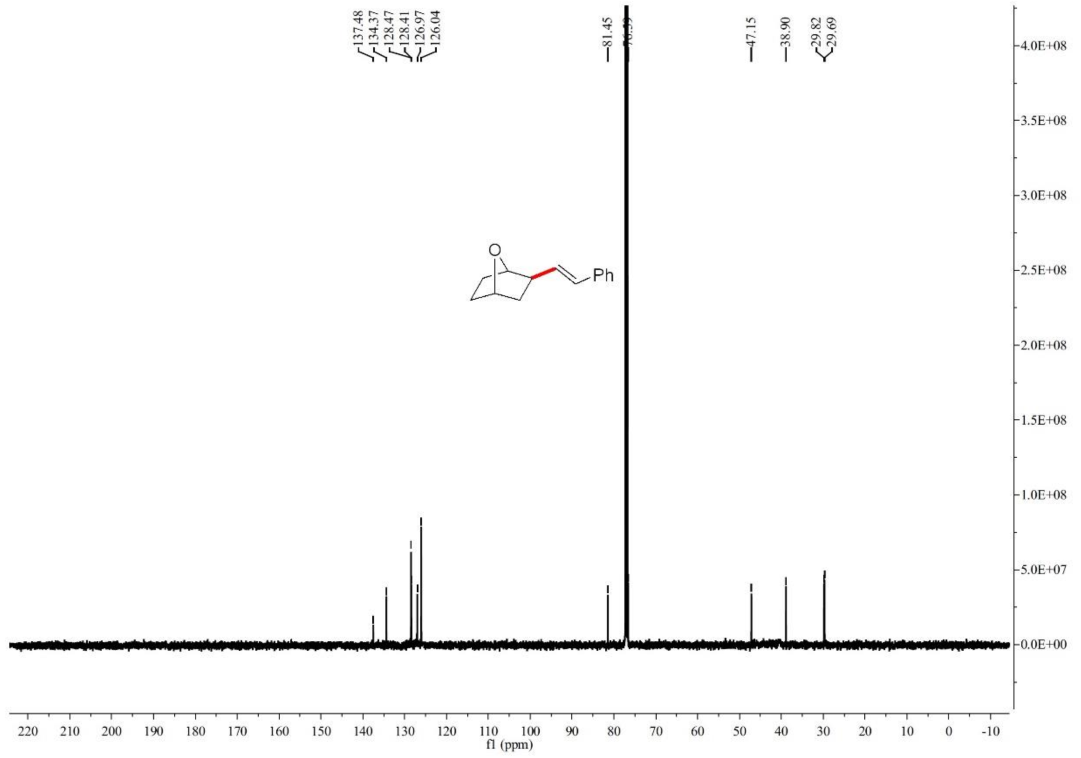

key COSY correlations

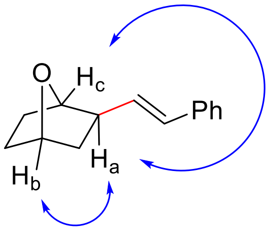

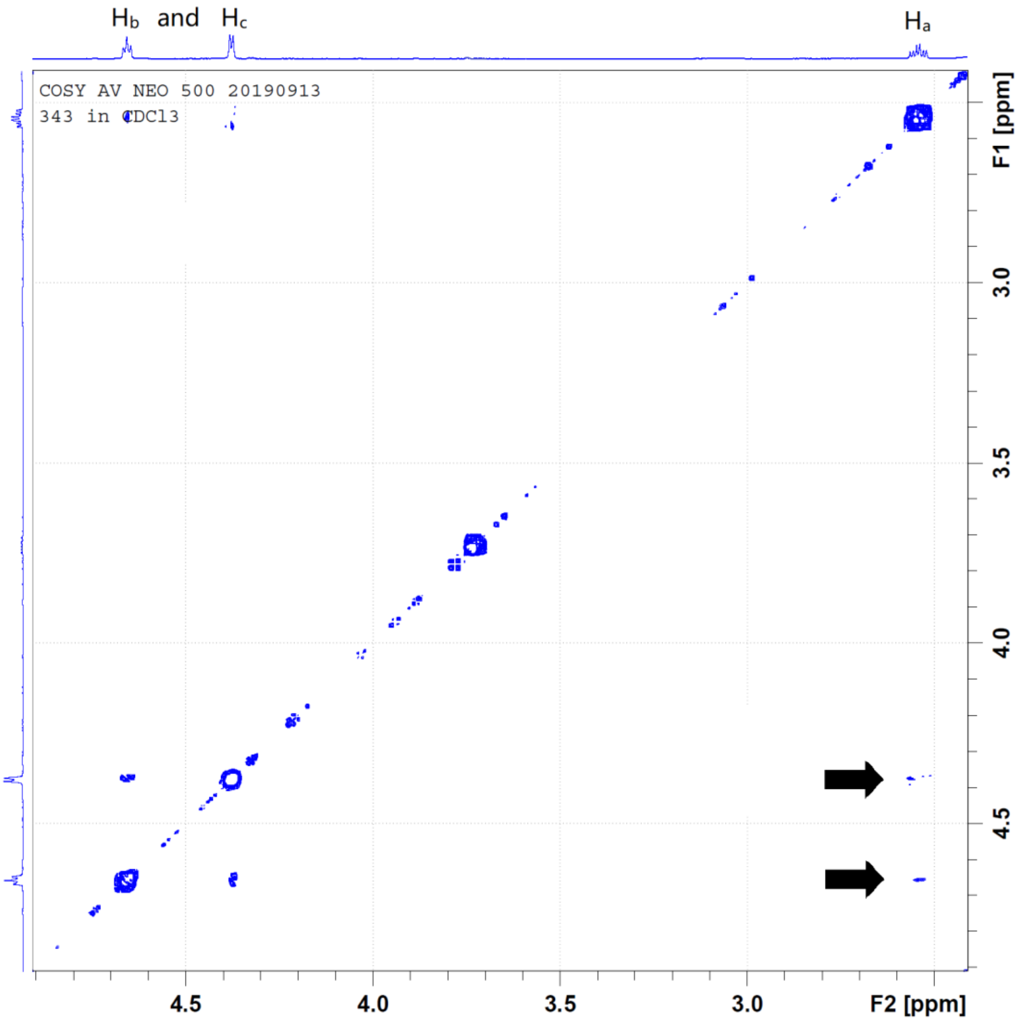

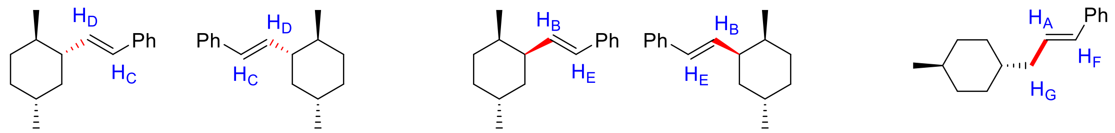

major 1 or major 2 major 2 or major 1 minor

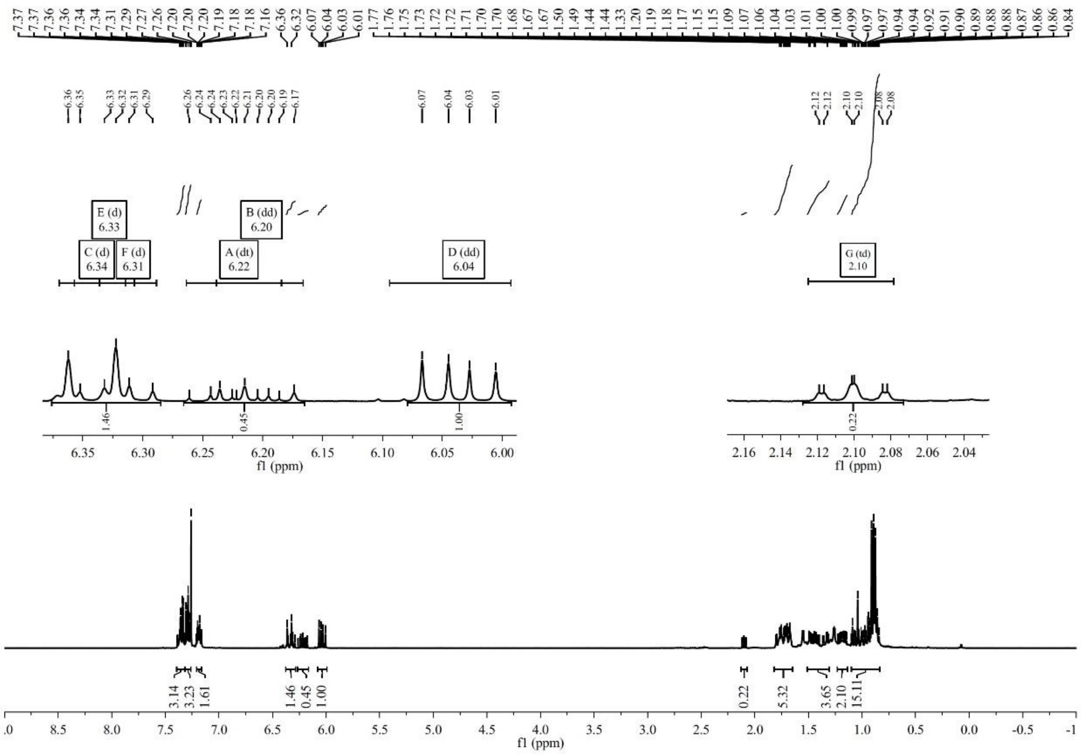

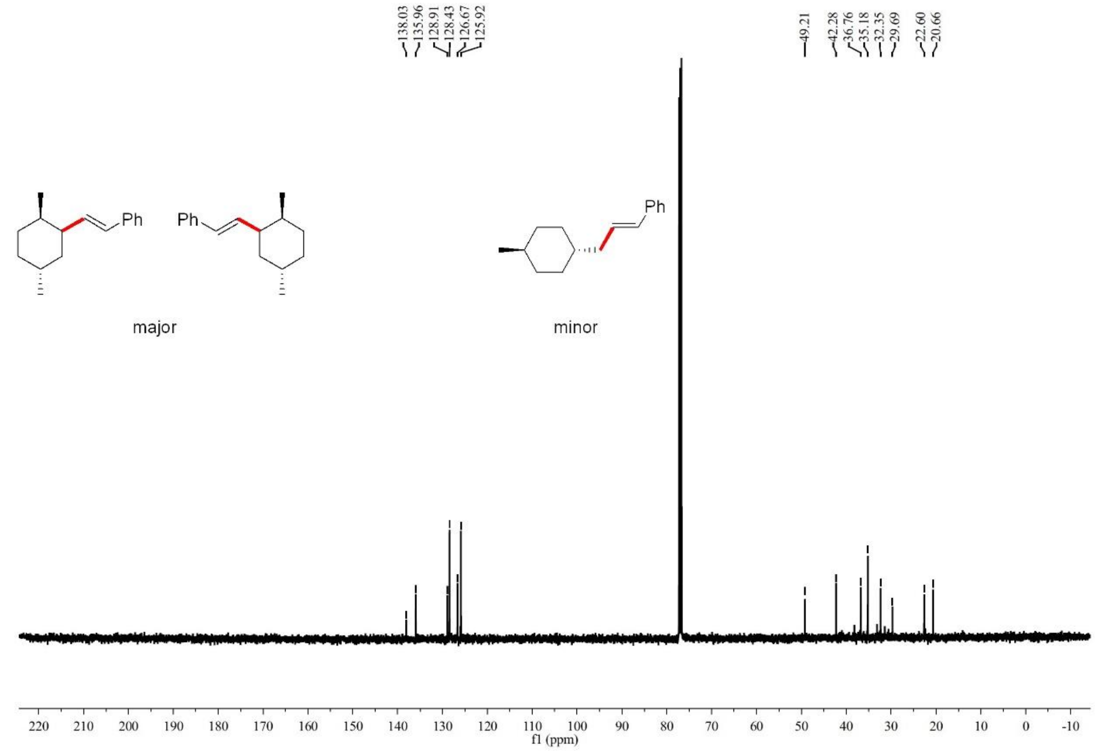

Ph
Ph
major
Ph
minor

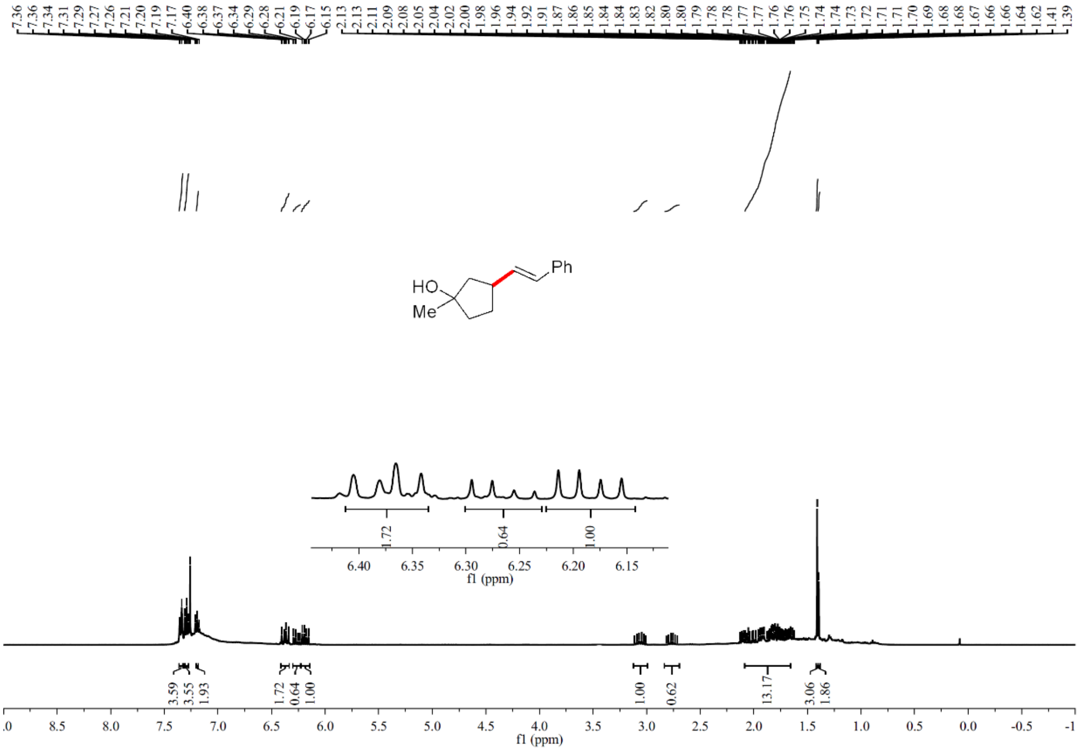

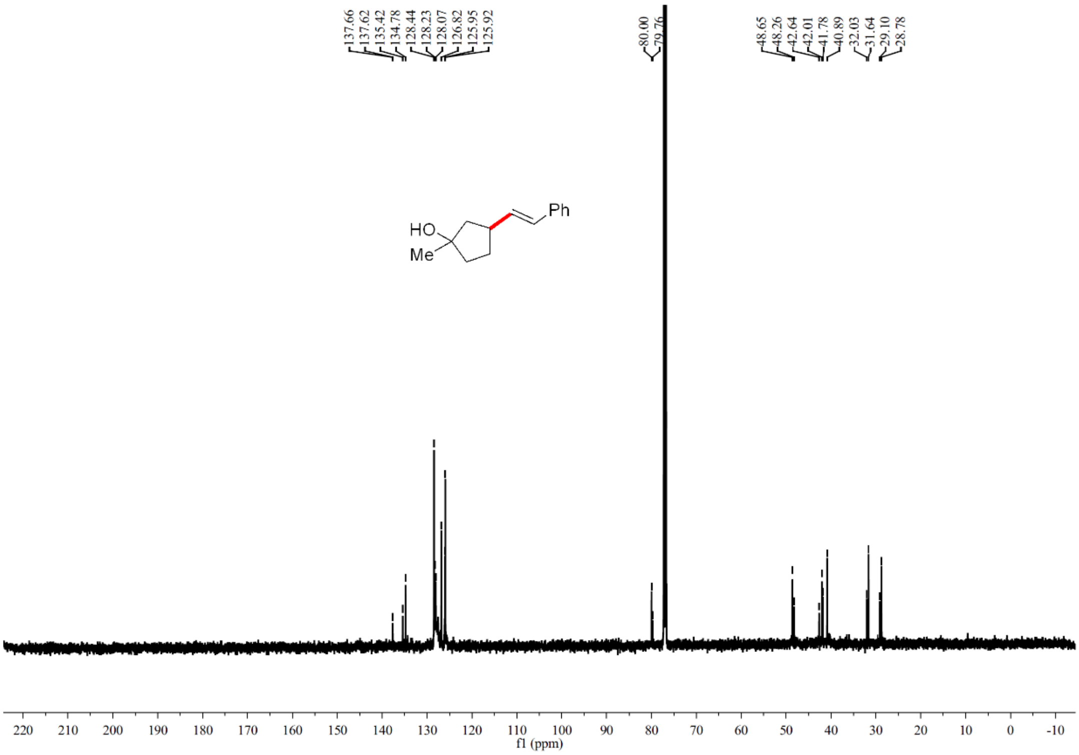

key NOESY correlations

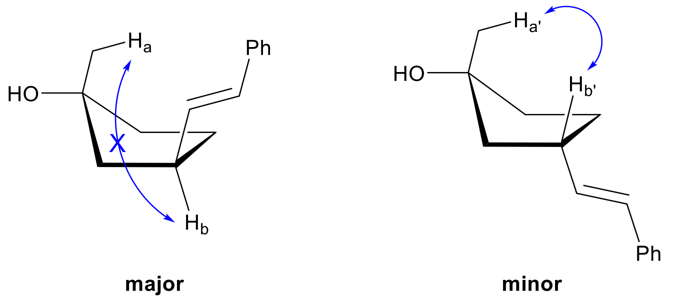

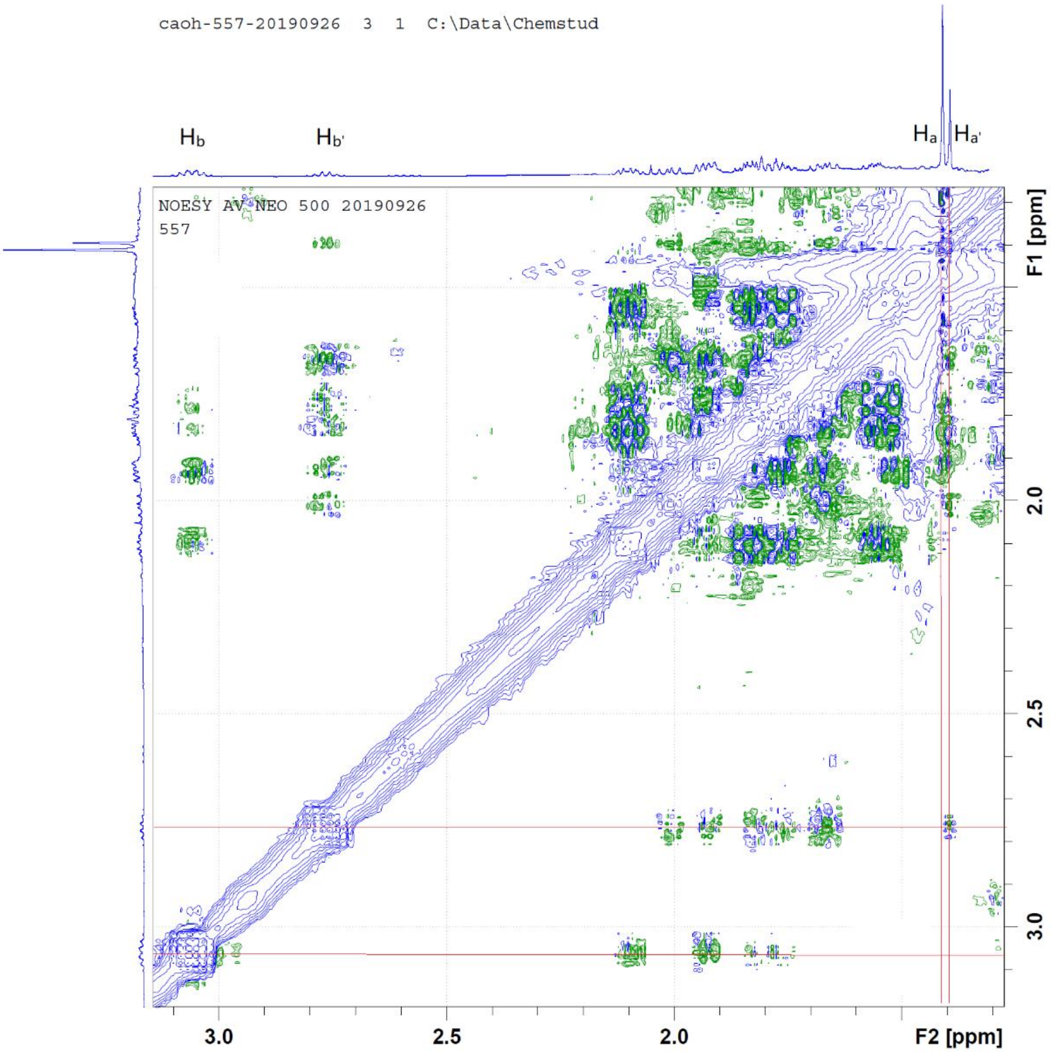

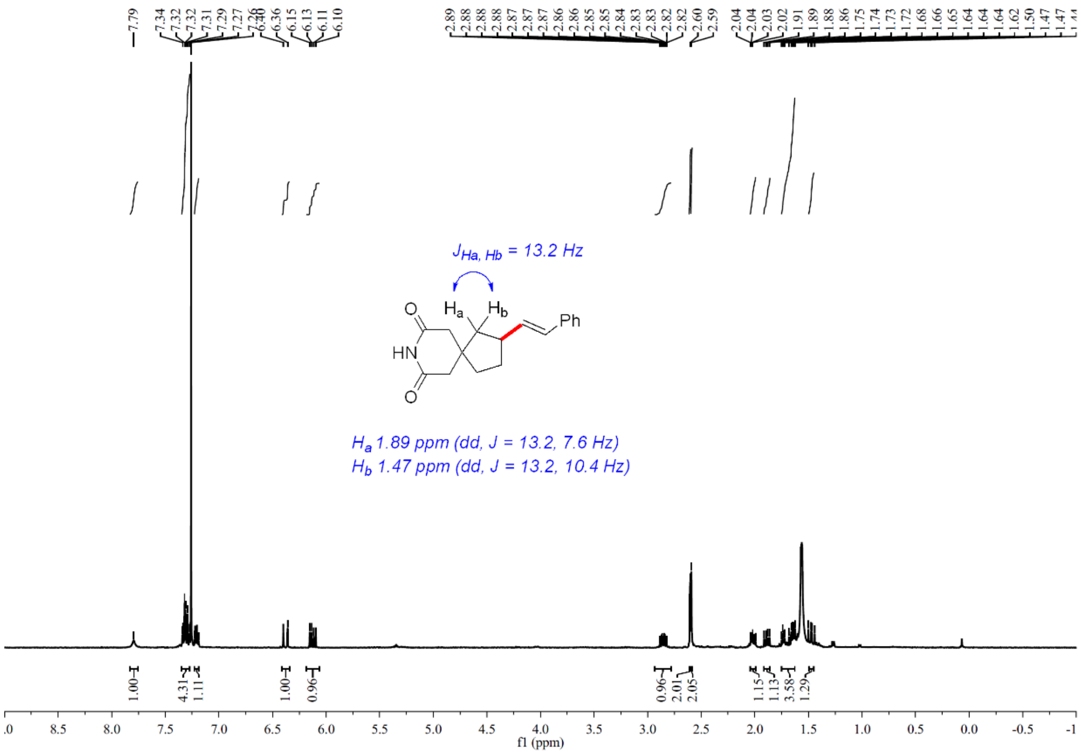
$J_{Ha, Hb}$ = 13.2 Hz
H_a 1.89 ppm (dd, J = 13.2, 7.6 Hz)
H_b 1.47 ppm (dd, J = 13.2, 10.4 Hz)
f1 (ppm)

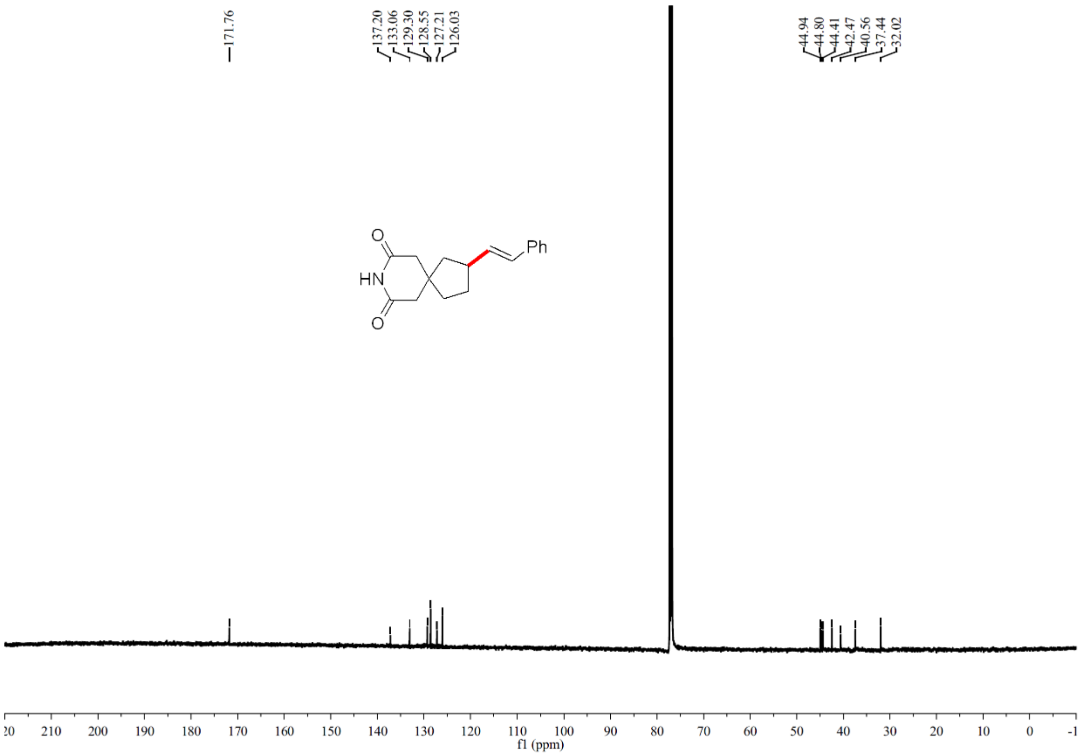
f1 (ppm)

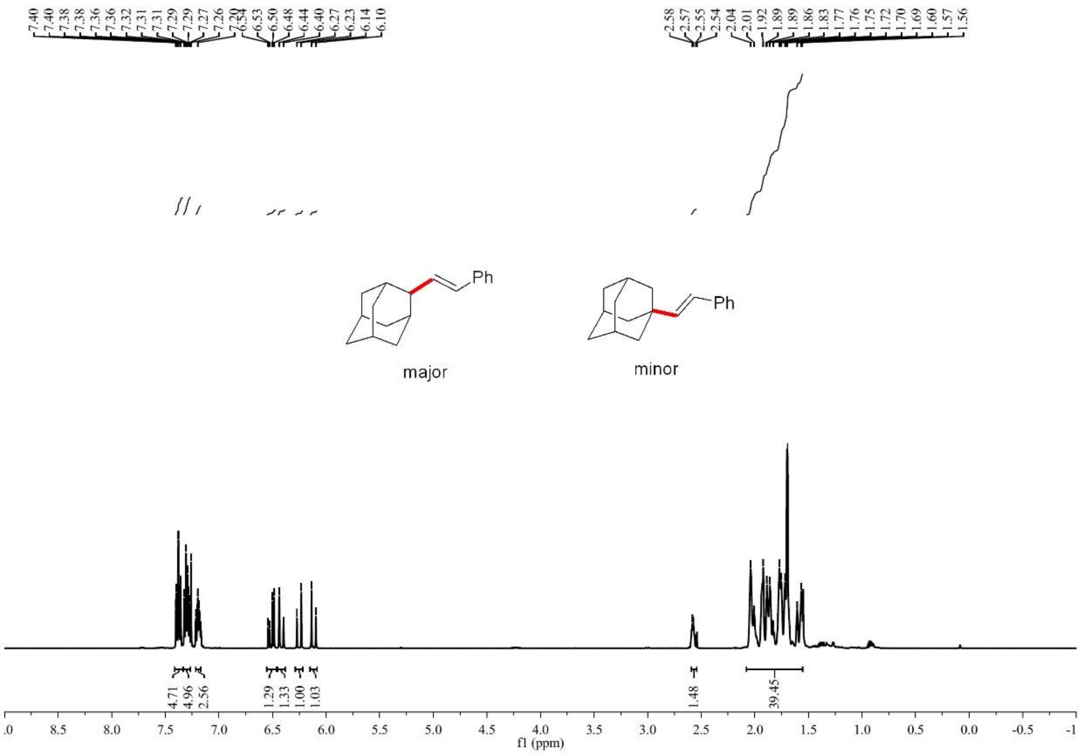

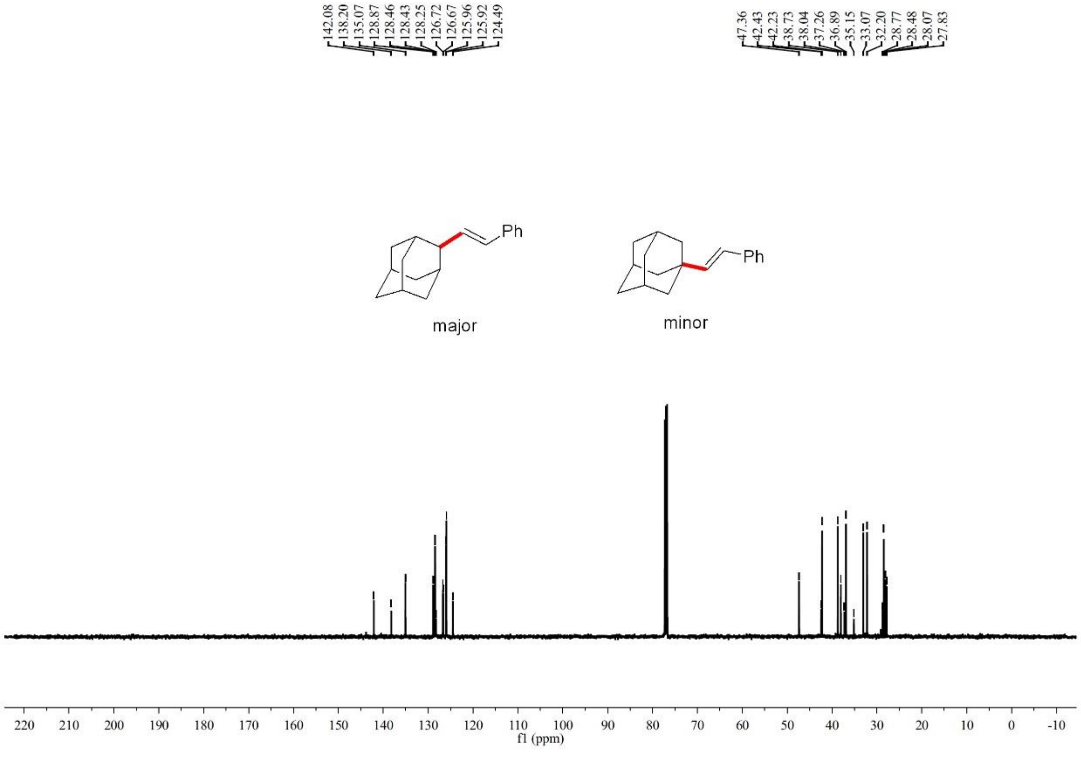

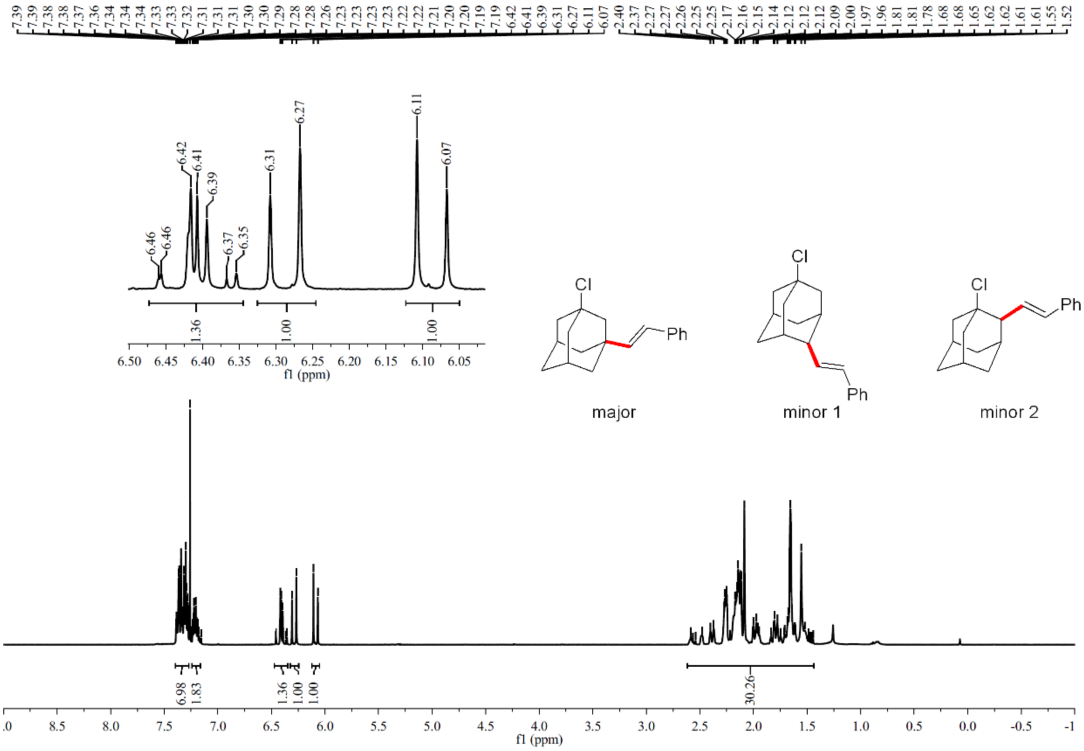
7.39 7.39 7.38 7.38 7.37 7.36 7.34 7.34 7.34 7.33 7.33 7.32 7.31 7.31 7.31 7.30 7.29 7.28 7.28 7.26 7.23 7.23 7.23 7.23 7.22 7.22 7.21 7.20 7.20 7.19 7.19 6.42 6.41 6.39 6.31 6.27 6.11 6.07 2.40 2.37 2.27 2.27 2.26 2.25 2.25 2.17 2.16 2.15 2.14 2.12 2.12 2.12 2.09 2.00 1.97 1.96 1.81 1.81 1.78 1.68 1.65 1.62 1.62 1.61 1.61 1.55 1.52
6.46 6.46 6.42 6.41 6.39 6.37 6.35 6.31 6.27 6.11 6.07
1.36 1.00 1.00
6.50 6.45 6.40 6.35 6.30 6.25 6.20 6.15 6.10 6.05
f1 (ppm)
Cl
Ph
major
minor 1
minor 2
6.98 1.83 1.36 1.00 1.00 30.26
.0 8.5 8.0 7.5 7.0 6.5 6.0 5.5 5.0 4.5 4.0 3.5 3.0 2.5 2.0 1.5 1.0 0.5 0.0 -0.5 -1
f1 (ppm)

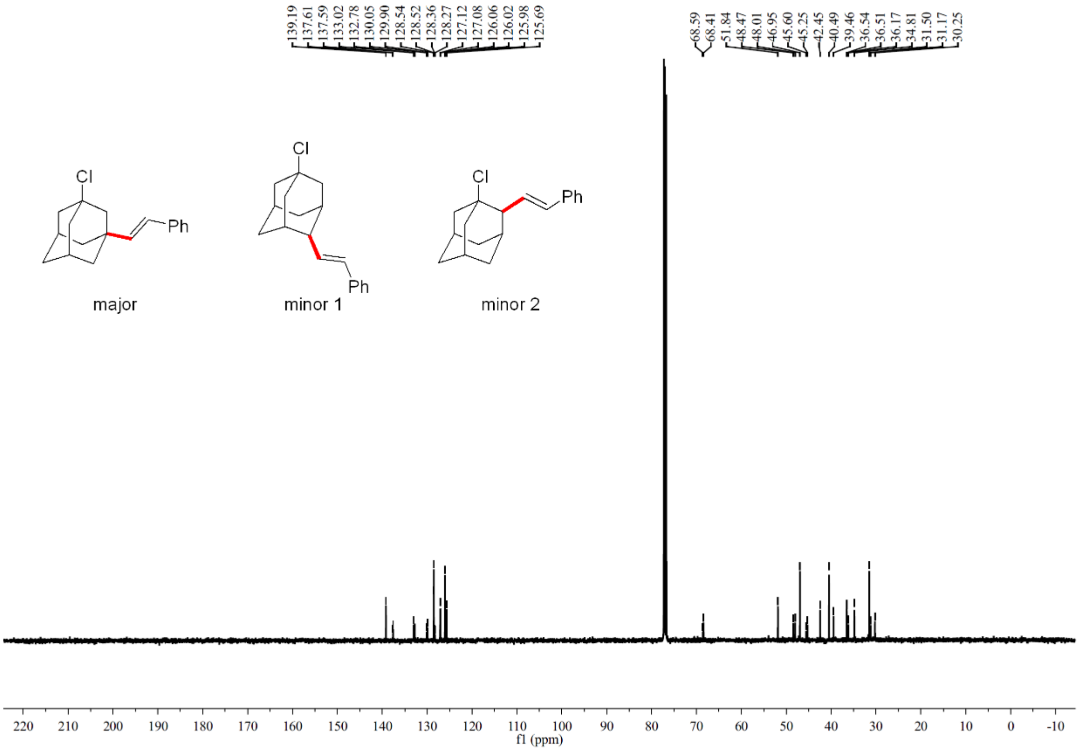
139.19 137.61 137.59 133.02 132.78 130.05 129.90 128.54 128.52 128.36 128.27 127.12 127.08 126.06 126.02 125.98 125.69
68.59 68.41 51.84 48.47 48.01 46.95 45.60 45.25 42.45 40.49 39.46 36.54 36.51 36.17 34.81 31.50 31.17 30.25
Cl
Ph
major
minor 1
minor 2
220 210 200 190 180 170 160 150 140 130 120 110 100 90 80 70 60 50 40 30 20 10 0 -10
f1 (ppm)

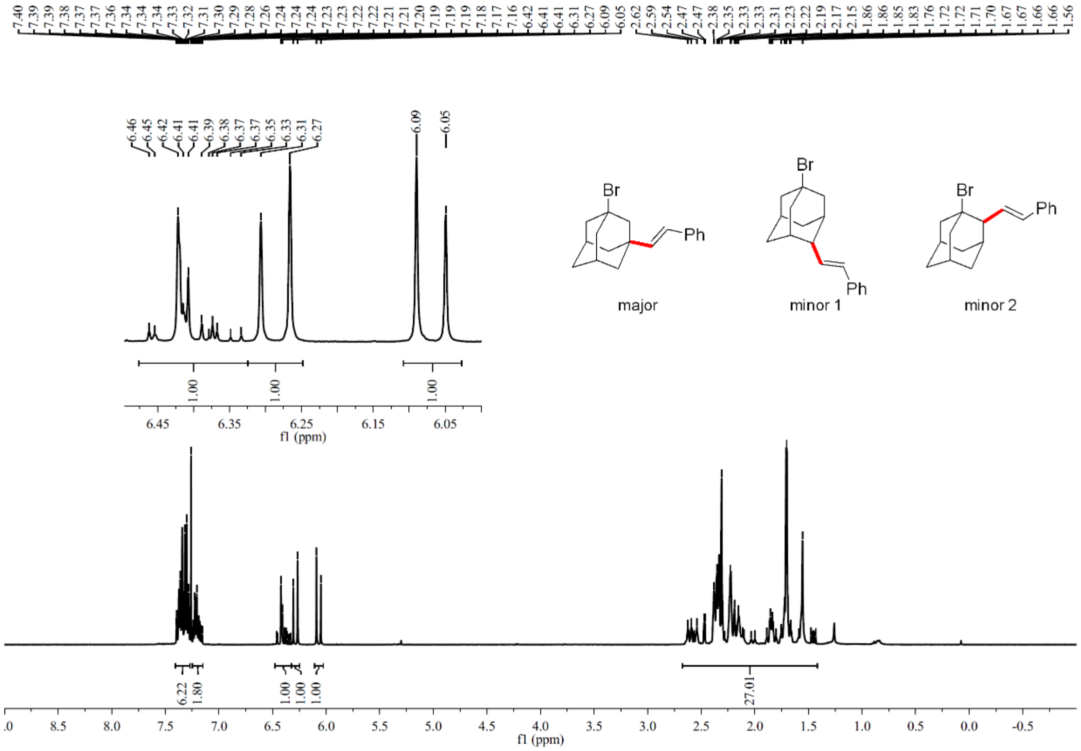
7.40 7.39 7.39 7.38 7.37 7.37 7.36 7.34 7.34 7.34 7.33 7.32 7.31 7.30 7.29 7.28 7.26 7.24 7.24 7.24 7.23 7.23 7.22 7.22 7.21 7.21 7.20 7.19 7.19 7.19 7.18 7.17 7.16 6.42 6.41 6.41 6.31 6.27 6.09 6.05 2.62 2.59 2.54 2.47 2.47 2.38 2.35 2.33 2.33 2.31 2.23 2.22 2.19 2.17 2.15 1.86 1.86 1.85 1.83 1.76 1.72 1.72 1.71 1.70 1.67 1.67 1.66 1.66 1.56
6.46 6.45 6.42 6.41 6.41 6.39 6.38 6.37 6.37 6.35 6.33 6.31 6.27
6.09
6.05
Br
Ph
major
minor 1
minor 2
1.00
1.00
1.00
f1 (ppm)
6.22
1.80
27.01

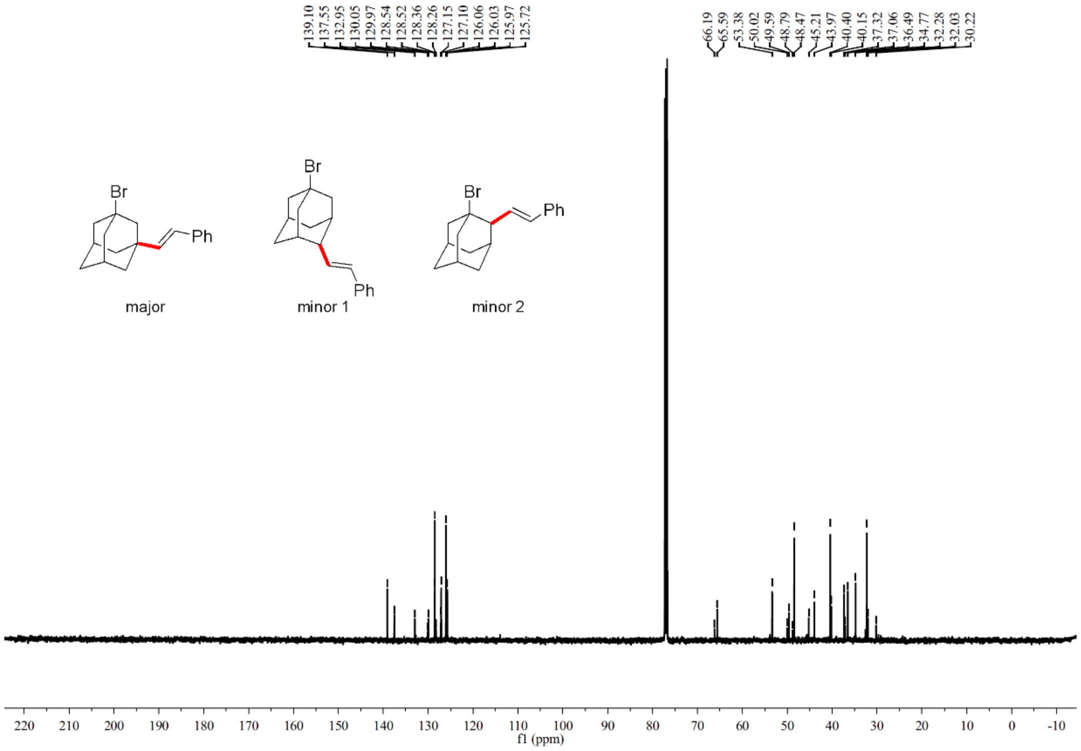
139.10 137.55 132.95 130.05 129.97 128.54 128.52 128.36 128.26 127.15 127.10 126.06 126.03 125.97 125.72
66.19 65.59 53.38 50.02 49.59 48.79 48.47 45.21 43.97 40.40 40.15 37.32 37.06 36.49 34.77 32.28 32.03 30.22
Br
Ph
major
minor 1
minor 2
f1 (ppm)

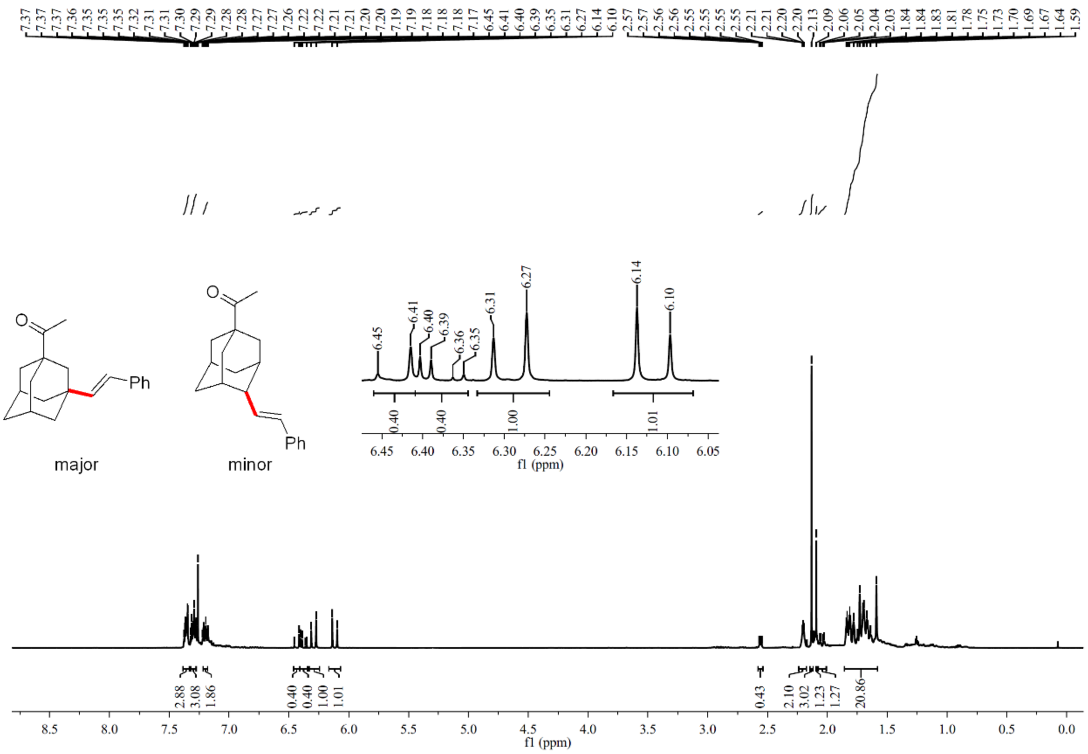

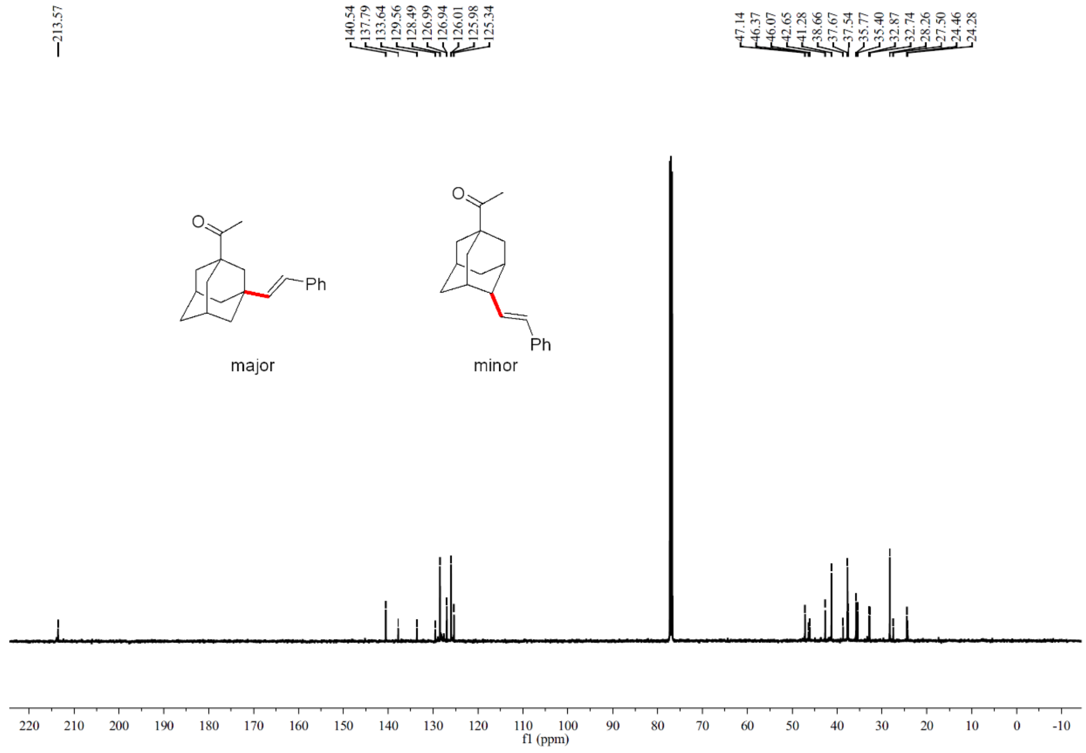

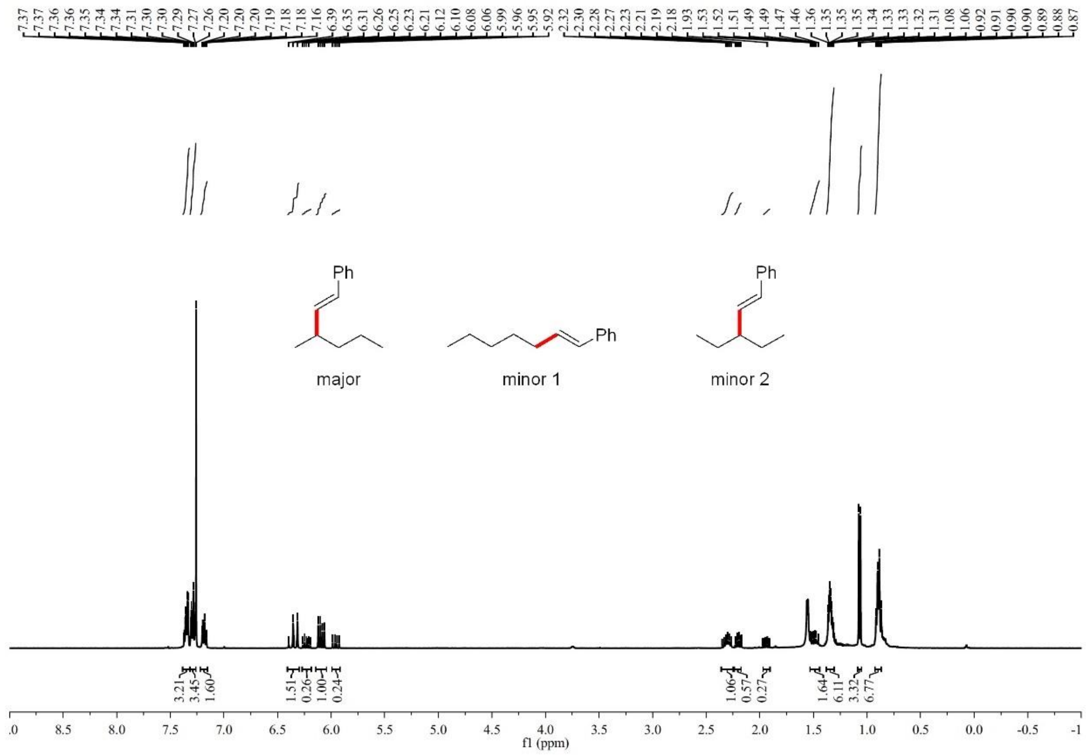
Ph
major
Ph
minor 1
Ph
minor 2
f1 (ppm)

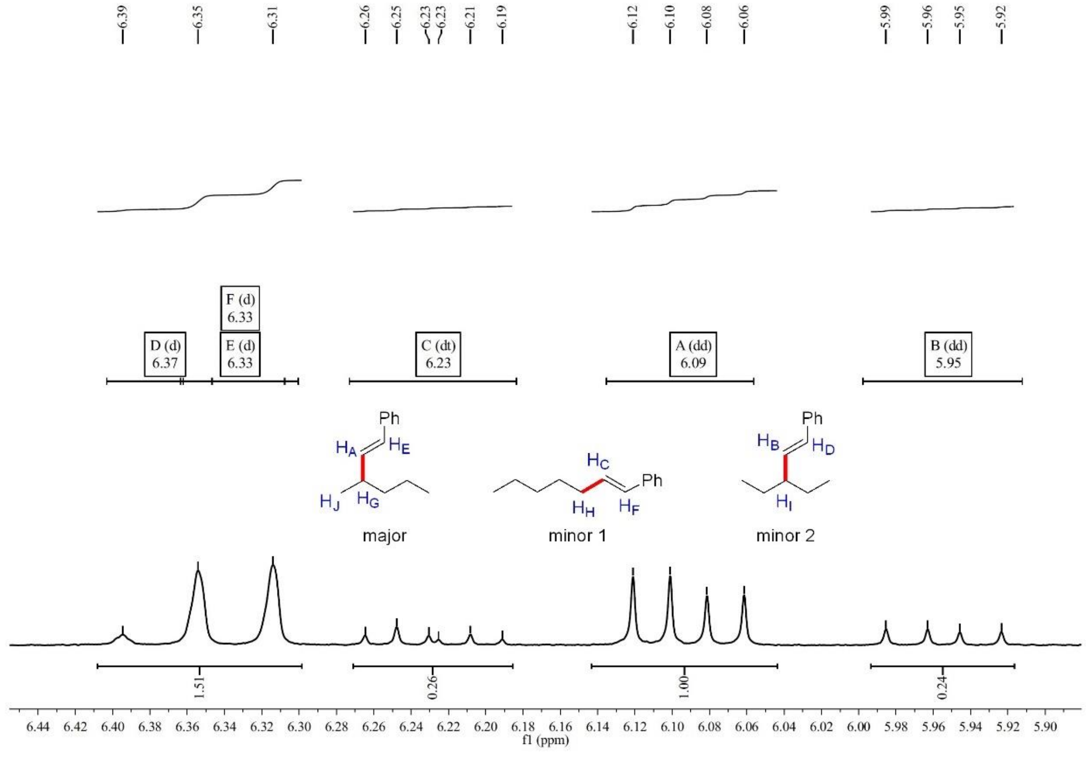
F (d)
6.33
D (d)
6.37
E (d)
6.33
C (dt)
6.23
A (dd)
6.09
B (dd)
5.95
major
minor 1
minor 2
f1 (ppm)

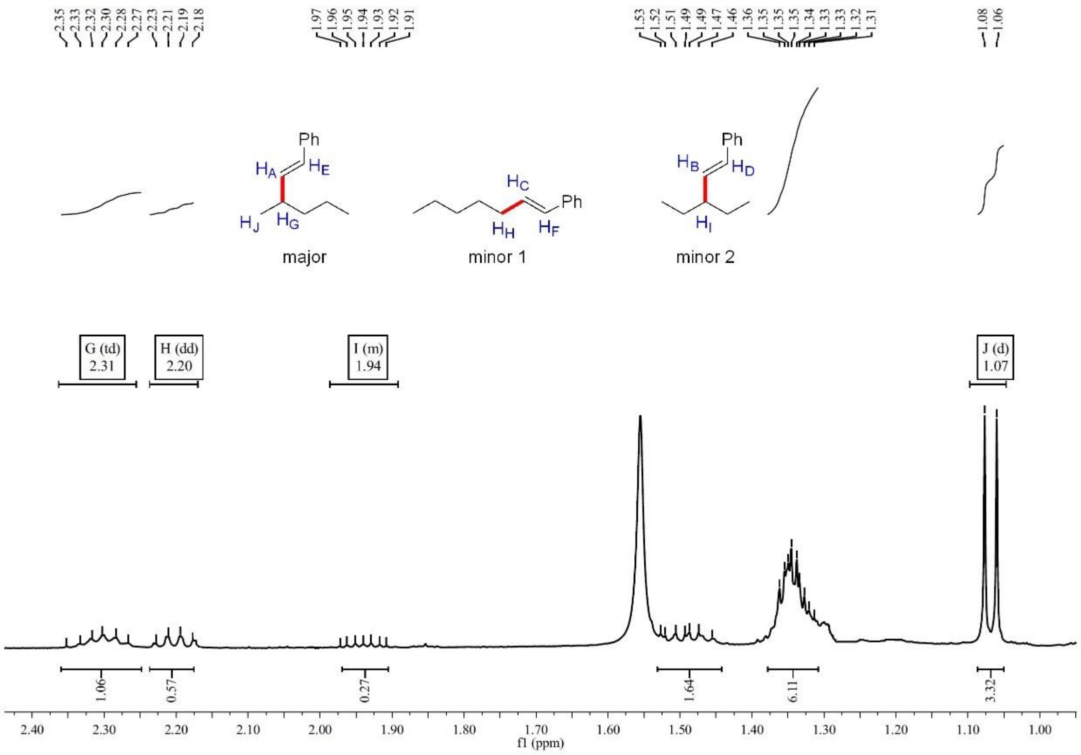
Ph
major
minor 1
minor 2
G (td) 2.31
H (dd) 2.20
I (m) 1.94
J (d) 1.07
f1 (ppm)

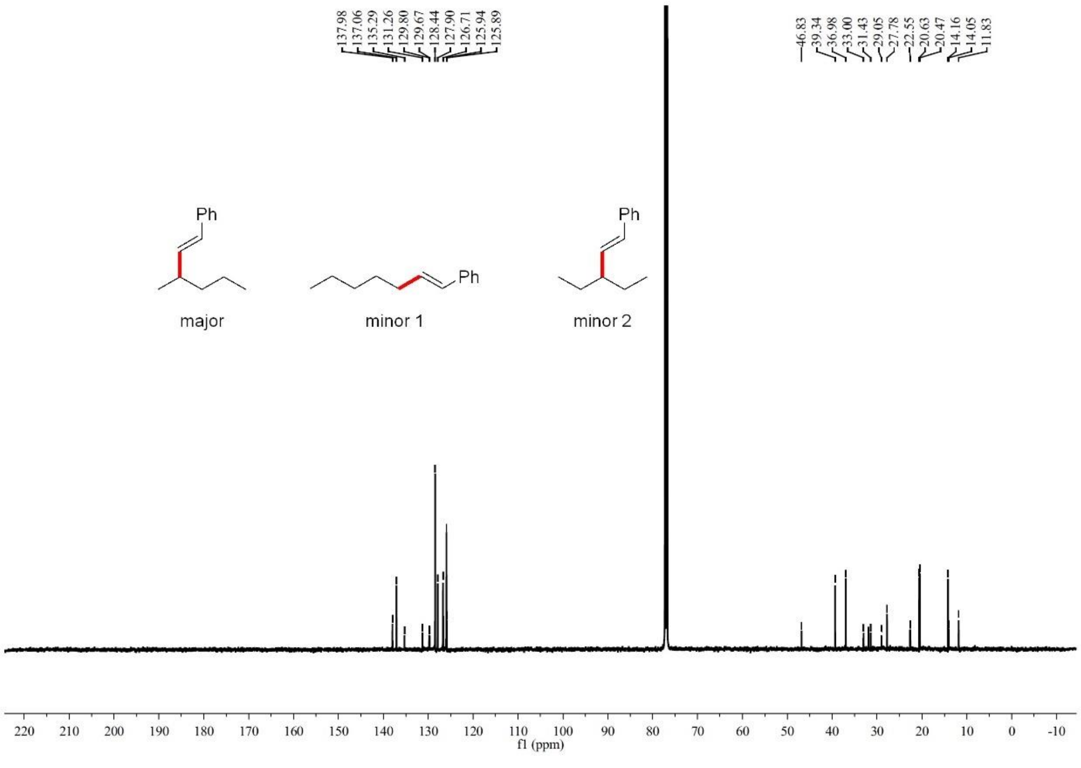
Ph
major
minor 1
minor 2
f1 (ppm)

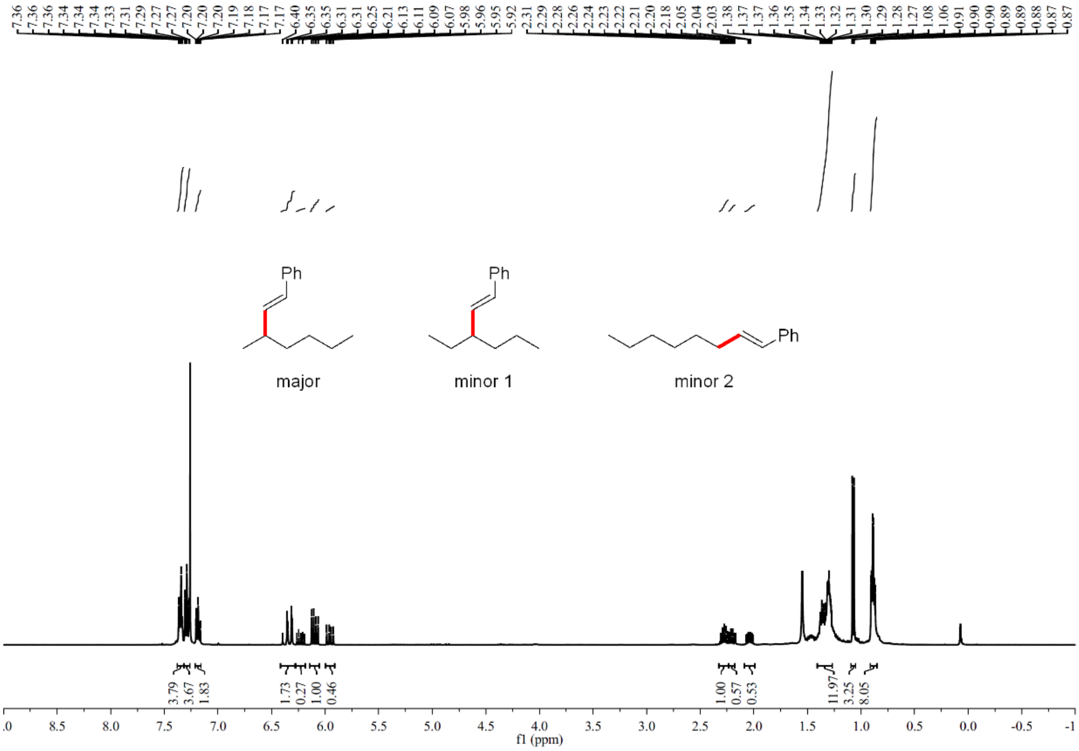

Ph
Ph
Ph
major
minor 1
minor 2
3.79
3.67
1.83
1.73
0.27
1.00
0.46
1.00
0.57
0.53
11.97
3.25
8.05
f1 (ppm)

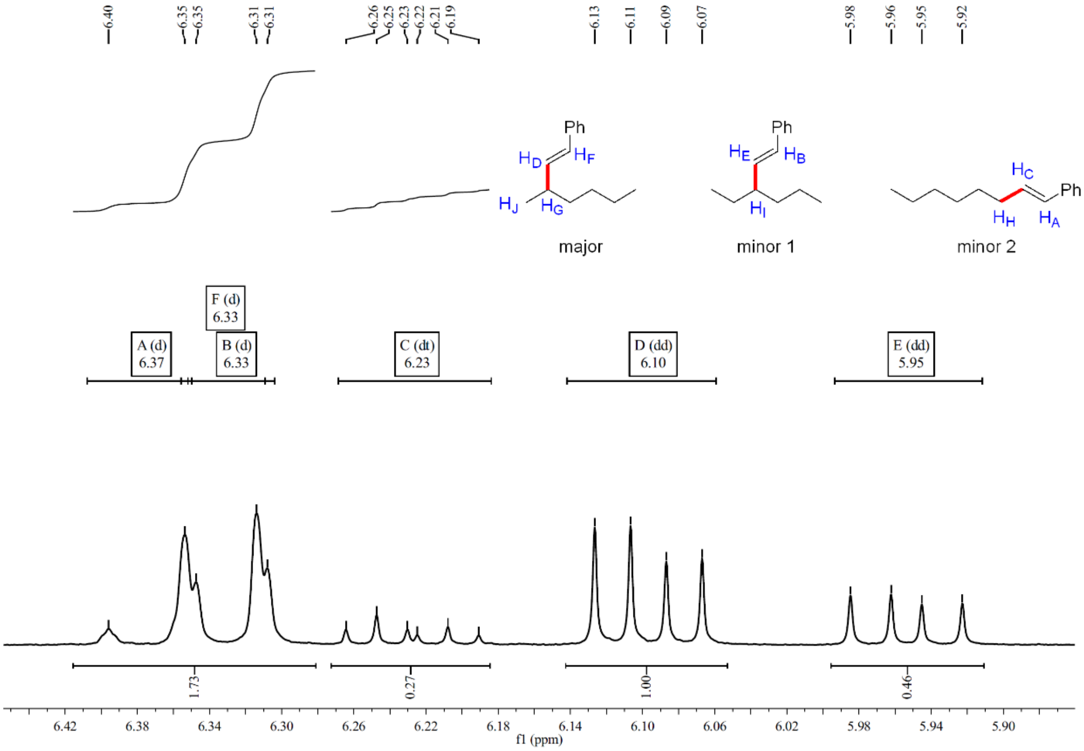

Ph
Ph
Ph
major
minor 1
minor 2
F (d)
6.33
A (d)
6.37
B (d)
6.33
C (dt)
6.23
D (dd)
6.10
E (dd)
5.95
1.73
0.27
1.00
0.46
f1 (ppm)

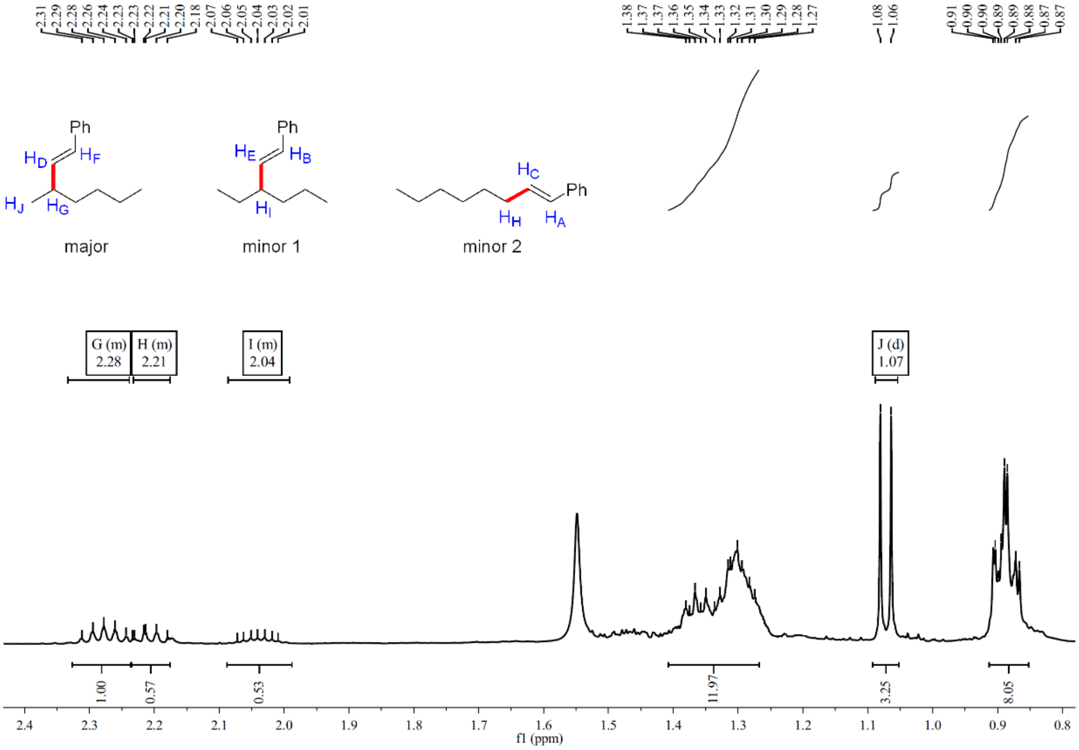
2.31 2.29 2.28 2.26 2.24 2.23 2.23 2.22 2.21 2.20 2.18 2.07 2.06 2.05 2.04 2.03 2.02 2.01
1.38 1.37 1.37 1.36 1.35 1.34 1.33 1.32 1.31 1.30 1.29 1.28 1.27
1.08 1.06
0.91 0.90 0.90 0.89 0.89 0.88 0.87 0.87
Ph
HD HF
HJ HG
major
Ph
HE HB
HI
minor 1
HC
Ph
HH HA
minor 2
G (m) 2.28
H (m) 2.21
I (m) 2.04
J (d) 1.07
1.00
0.57
0.53
11.97
3.25
8.05
f1 (ppm)

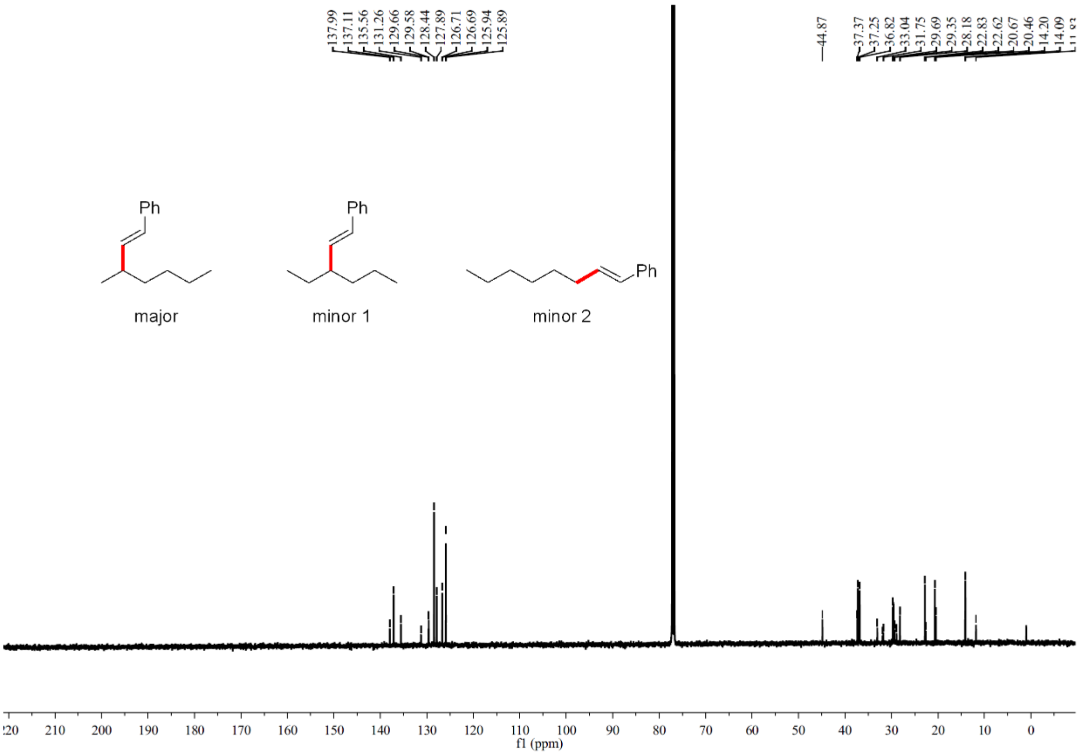
137.99 137.11 135.56 131.26 129.66 129.58 128.44 127.89 126.71 126.69 125.94 125.89
44.87
37.37 37.25 36.82 33.04 31.75 29.69 29.35 28.18 22.83 22.62 20.67 20.46 14.20 14.09
Ph
major
Ph
minor 1
Ph
minor 2
f1 (ppm)

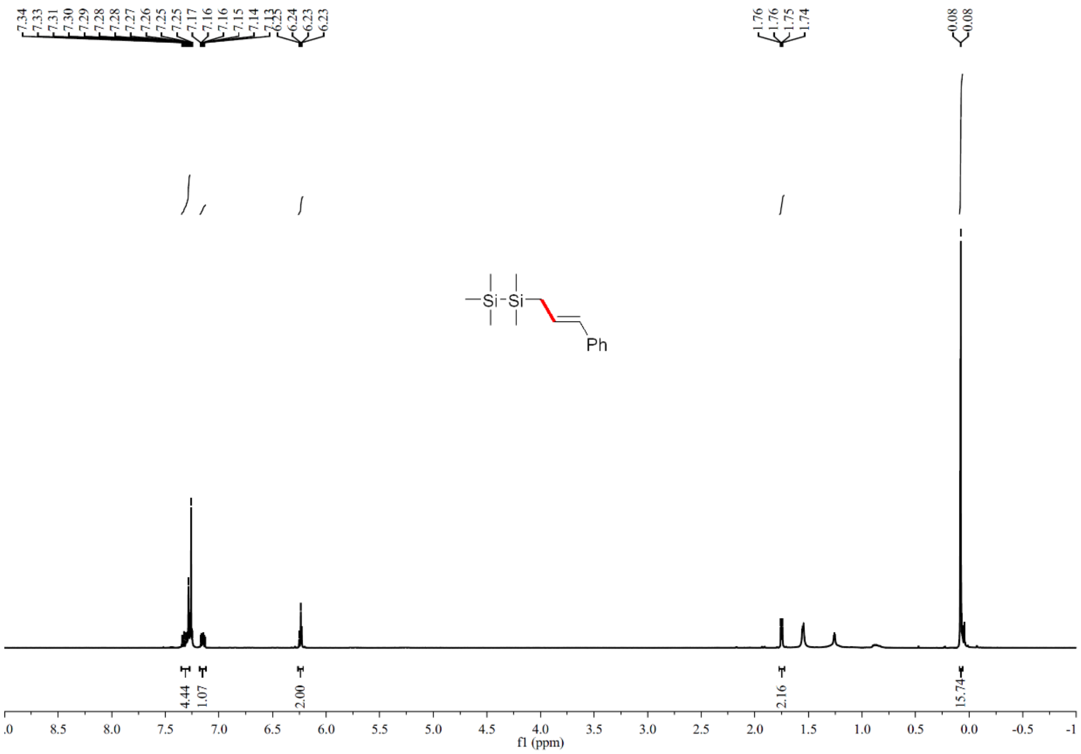

Si
Si
Ph
f1 (ppm)

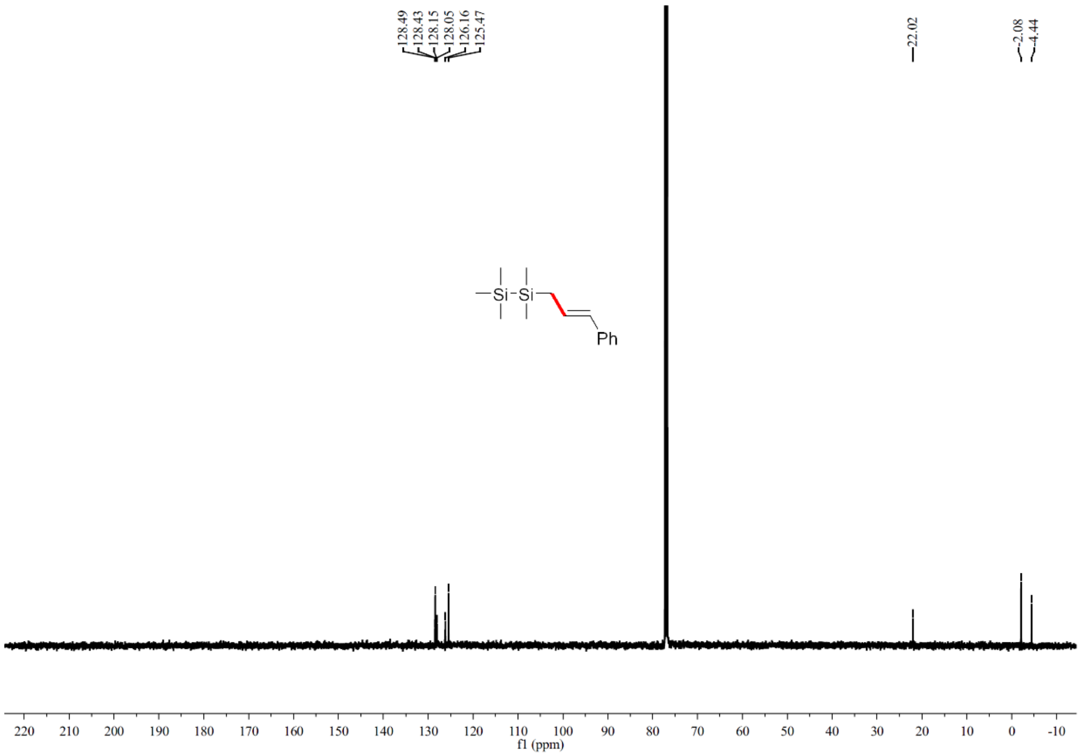

Si
Si
Ph
f1 (ppm)

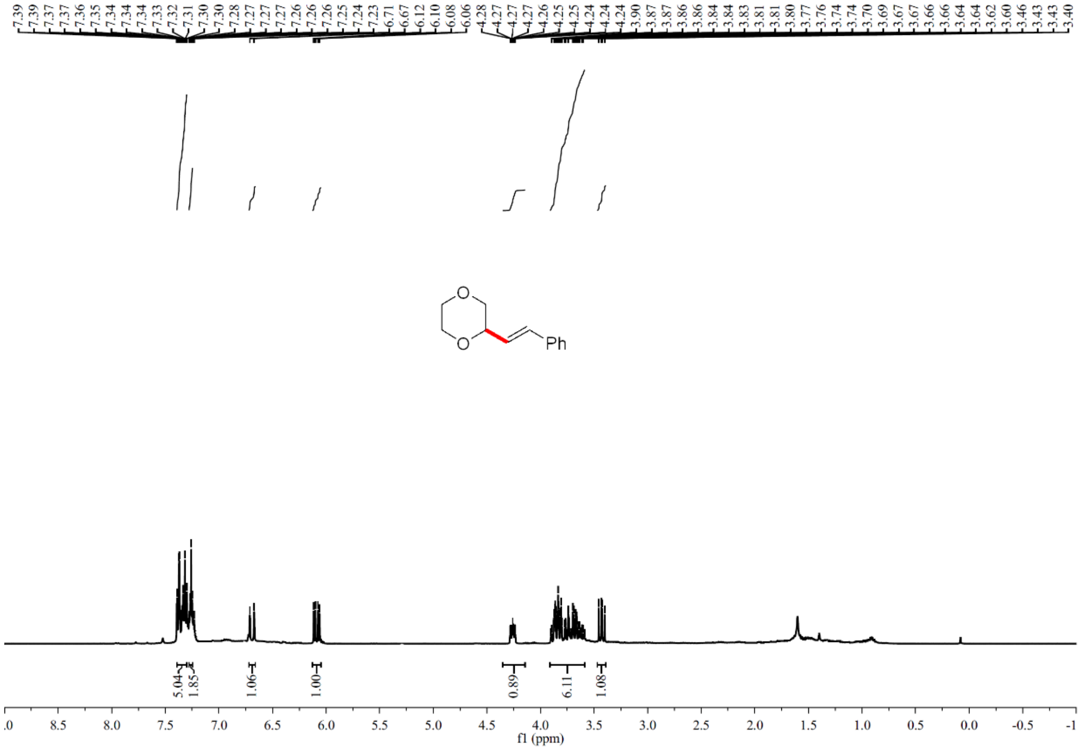

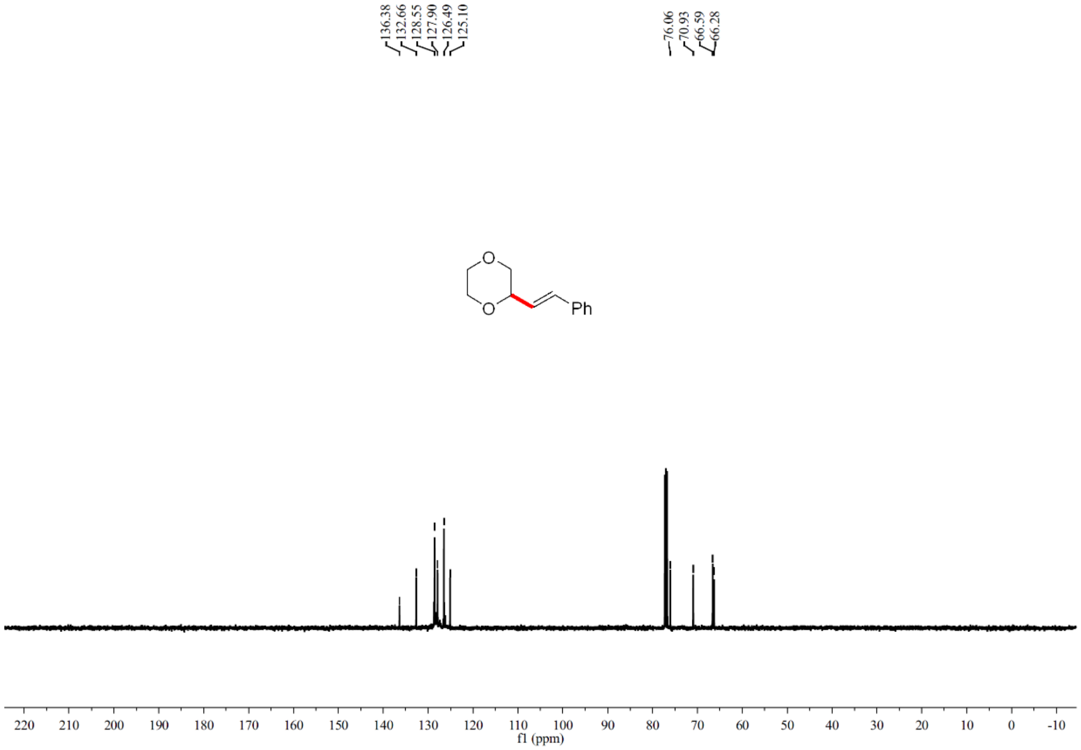

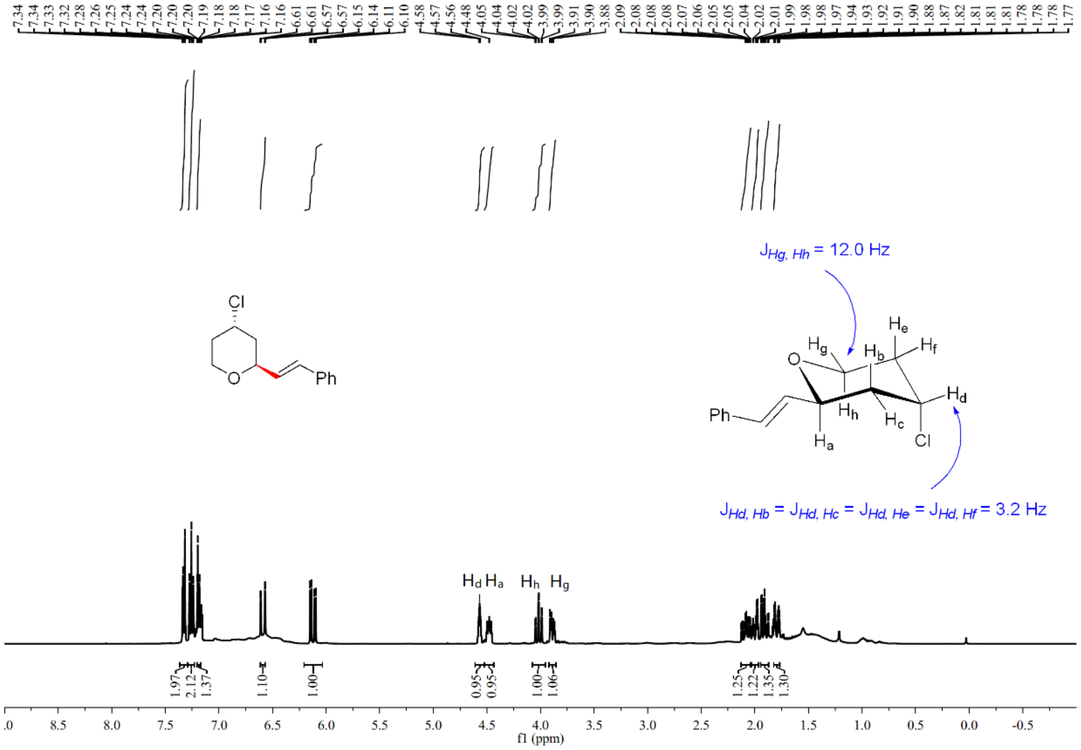

7.34
7.34
7.33
7.32
7.28
7.26
7.25
7.24
7.24
7.20
7.20
7.20
7.19
7.18
7.18
7.17
7.16
7.16
6.61
6.61
6.57
6.57
6.15
6.14
6.11
6.10
4.58
4.57
4.56
4.48
4.05
4.04
4.02
4.02
3.99
3.99
3.91
3.90
3.88
2.09
2.08
2.08
2.08
2.07
2.06
2.05
2.05
2.04
2.02
2.01
1.99
1.98
1.98
1.97
1.94
1.93
1.92
1.91
1.90
1.88
1.87
1.82
1.81
1.81
1.81
1.78
1.78
1.77
Cl
O
Ph
$J_{Hg, Hh}$ = 12.0 Hz
He
Hf
Hg
Hb
O
Hd
Ph
Hh
Hc
Ha
Cl
$J_{Hd, Hb}$ = $J_{Hd, Hc}$ = $J_{Hd, He}$ = $J_{Hd, Hf}$ = 3.2 Hz
Hd Ha
Hh
Hg
1.97
2.12
1.37
1.10
1.00
0.95
0.95
1.00
1.06
1.25
1.22
1.35
1.30
.0
8.5
8.0
7.5
7.0
6.5
6.0
5.5
5.0
4.5
4.0
3.5
3.0
2.5
2.0
1.5
1.0
0.5
0.0
-0.5
f1 (ppm)

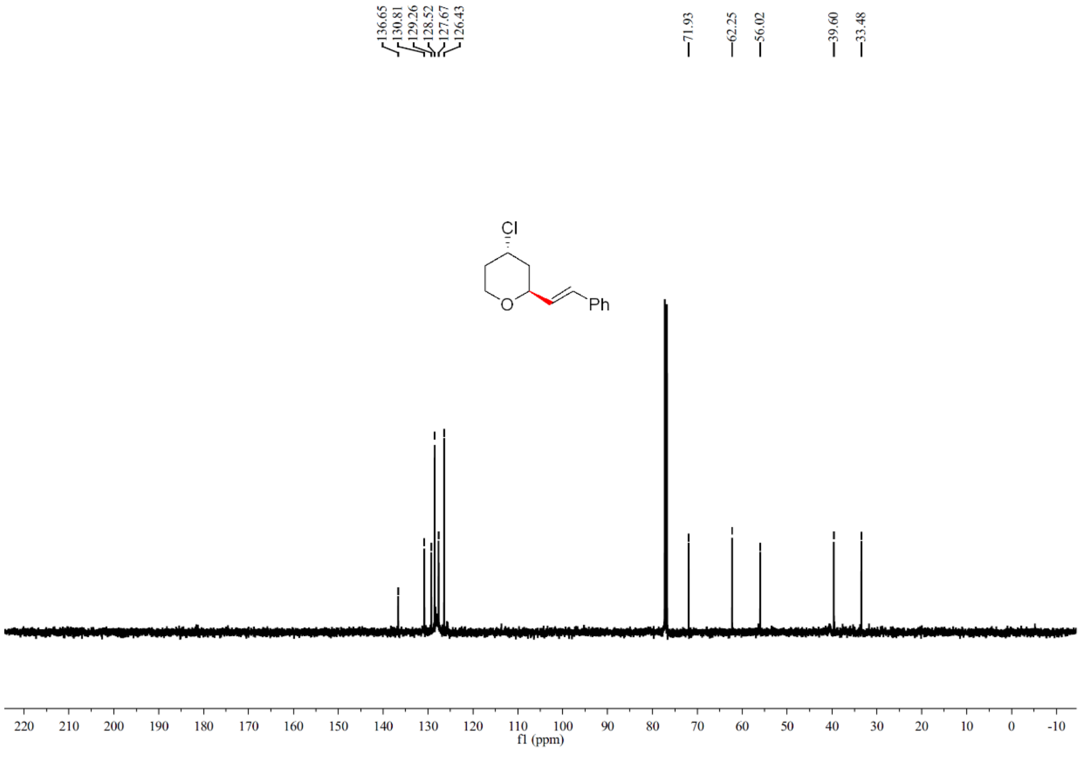

136.65
130.81
129.26
128.52
127.67
126.43
71.93
62.25
56.02
39.60
33.48
Cl
O
Ph
220
210
200
190
180
170
160
150
140
130
120
110
100
90
80
70
60
50
40
30
20
10
0
-10
f1 (ppm)

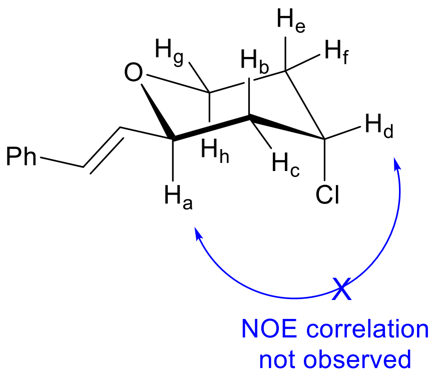

O
Ph
Ha
Hb
Hc
Hd
He
Hf
Hg
Hh
Cl
NOE correlation
not observed

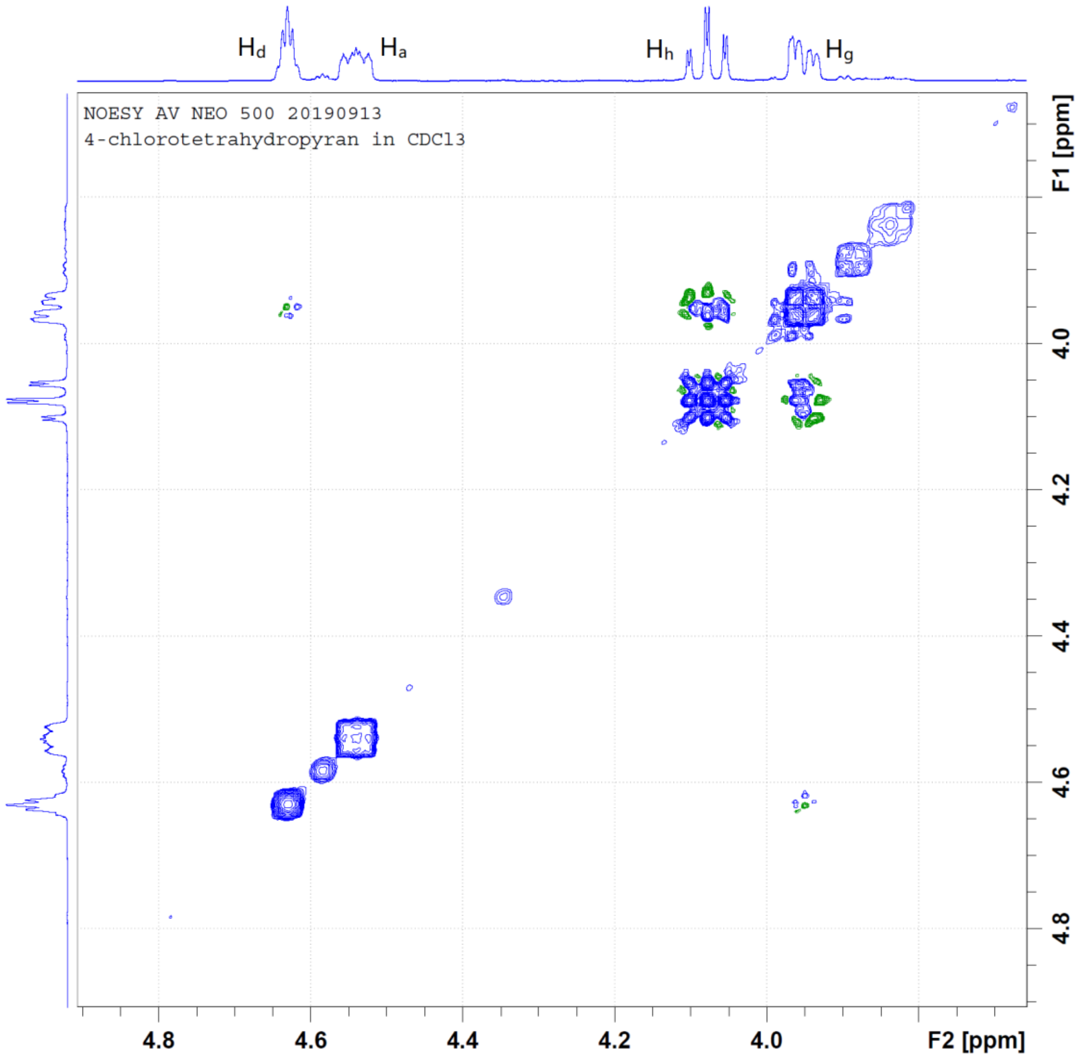

Hd
Ha
Hh
Hg
NOESY AV NEO 500 20190913
4-chlorotetrahydropyran in CDCl3
F1 [ppm]
F2 [ppm]
4.8
4.6
4.4
4.2
4.0

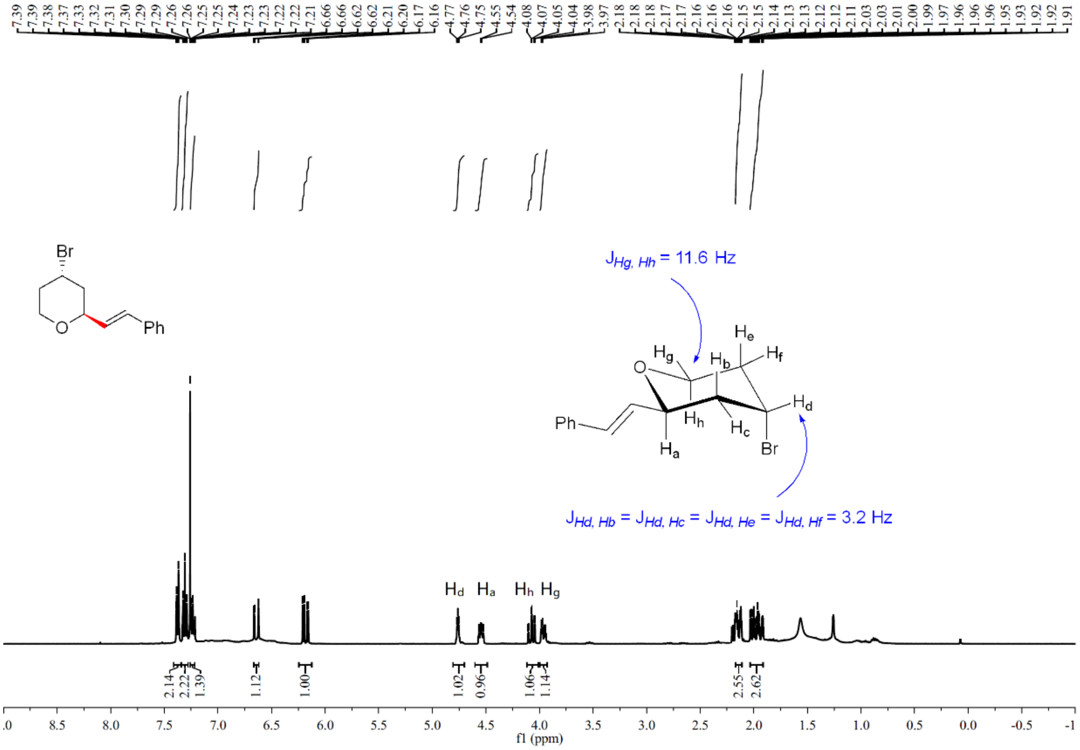

Br
Ph
O
$J_{Hg, Hh}$ = 11.6 Hz
$J_{Hd, Hb}$ = $J_{Hd, Hc}$ = $J_{Hd, He}$ = $J_{Hd, Hf}$ = 3.2 Hz
f1 (ppm)

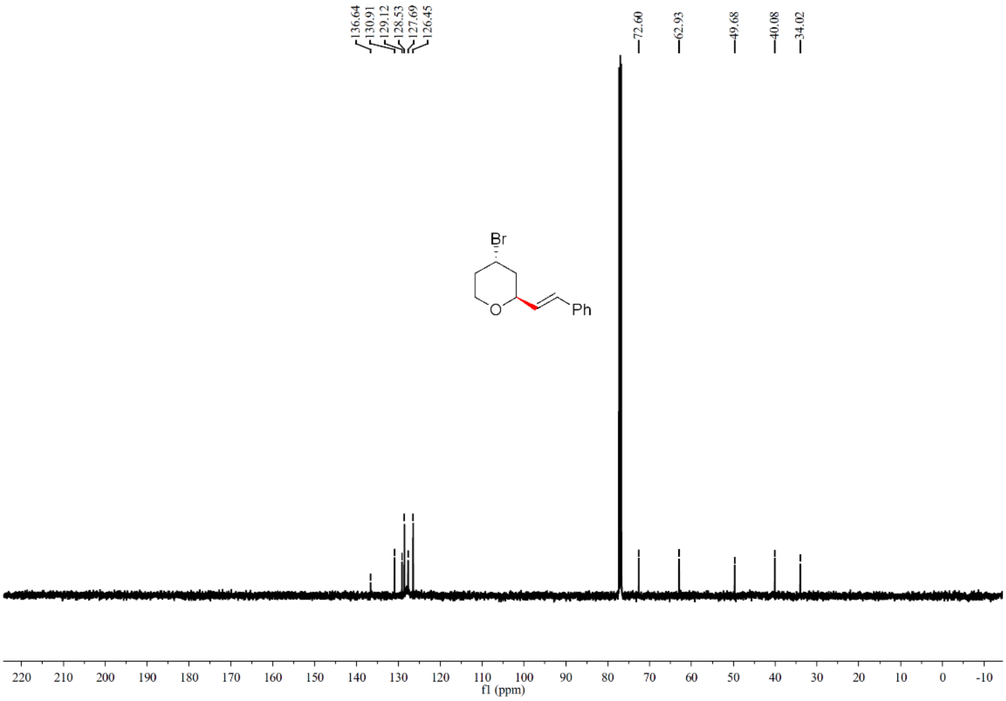

136.64
130.91
129.12
128.53
127.69
126.45
72.60
62.93
49.68
40.08
34.02
Br
O
Ph
f1 (ppm)

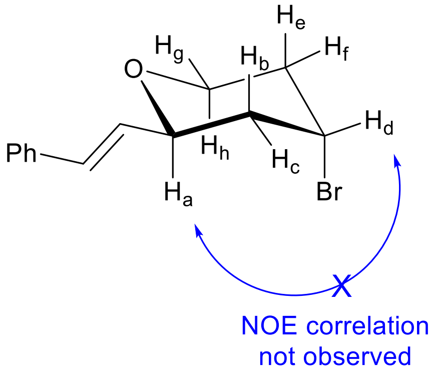
He
Hf
Hg
Hb
O
Hd
Ph
Hh
Hc
Ha
Br
NOE correlation
not observed

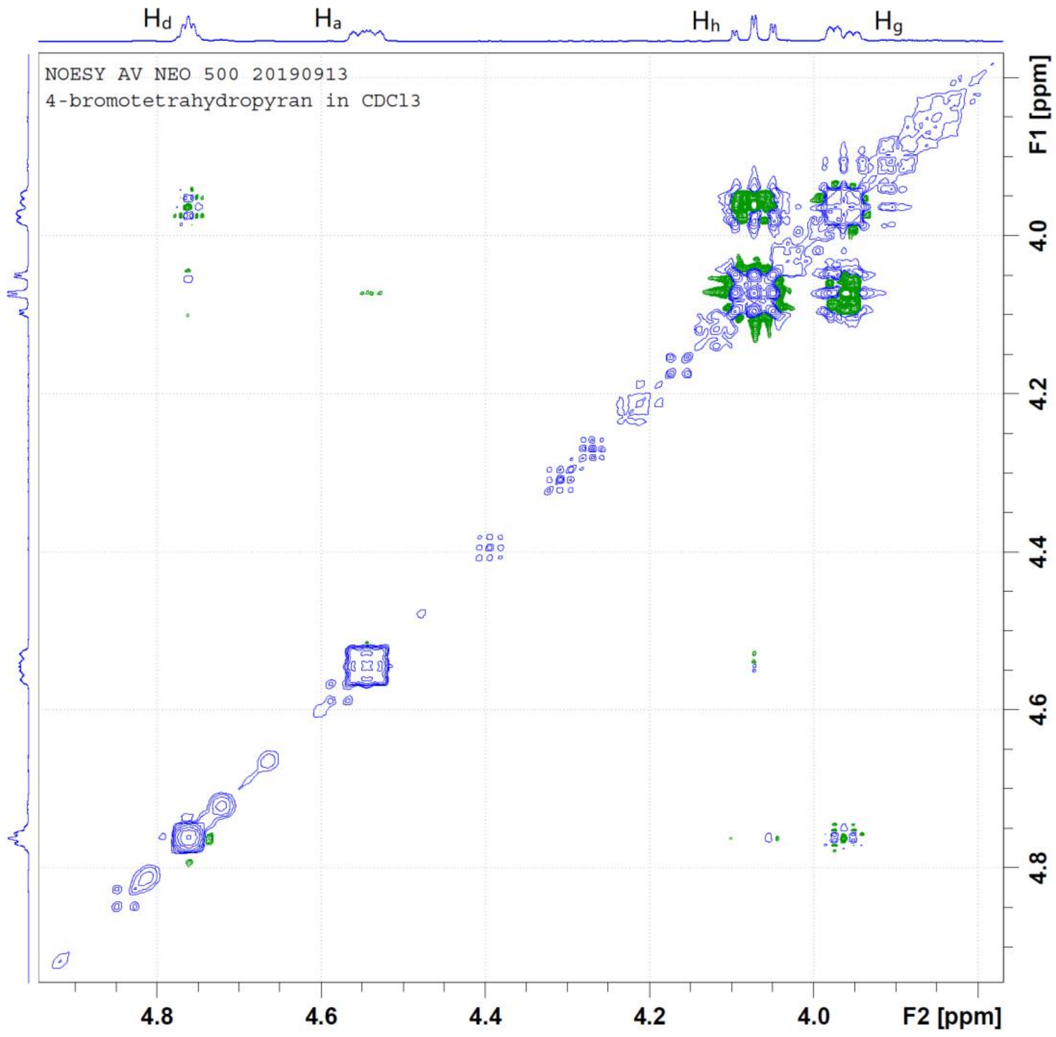
Hd
Ha
Hh
Hg
NOESY AV NEO 500 20190913
4-bromotetrahydropyran in CDCl3
F1 [ppm]
4.0
4.2
4.4
4.6
4.8
4.8
4.6
4.4
4.2
4.0
F2 [ppm]

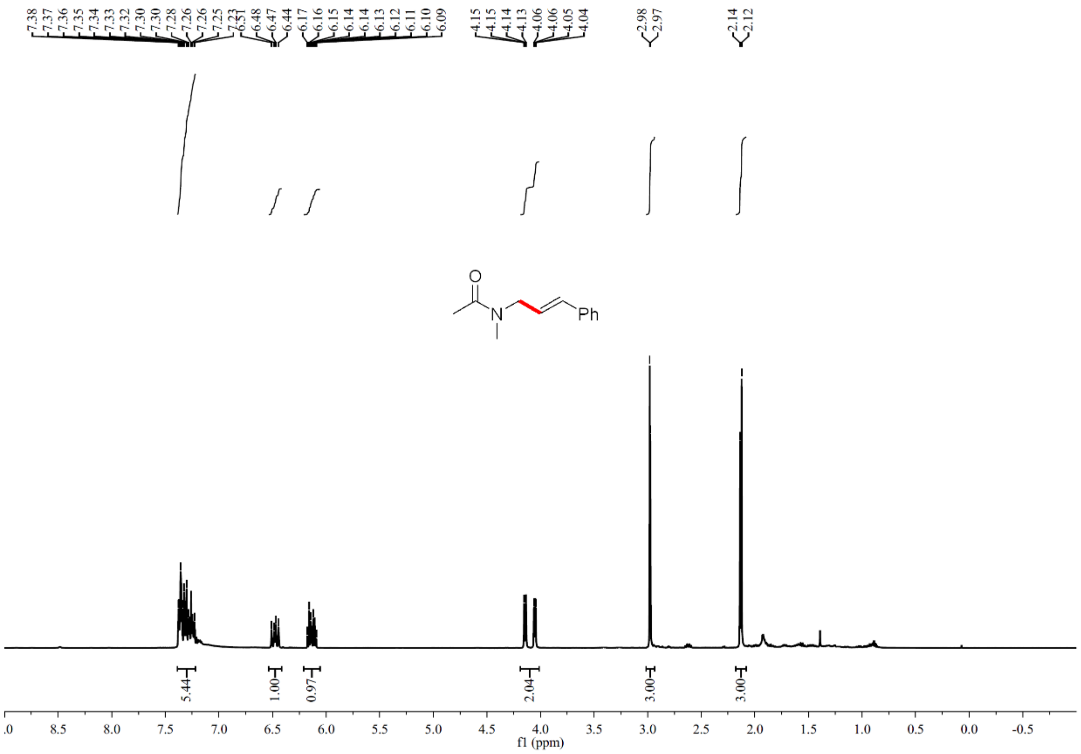

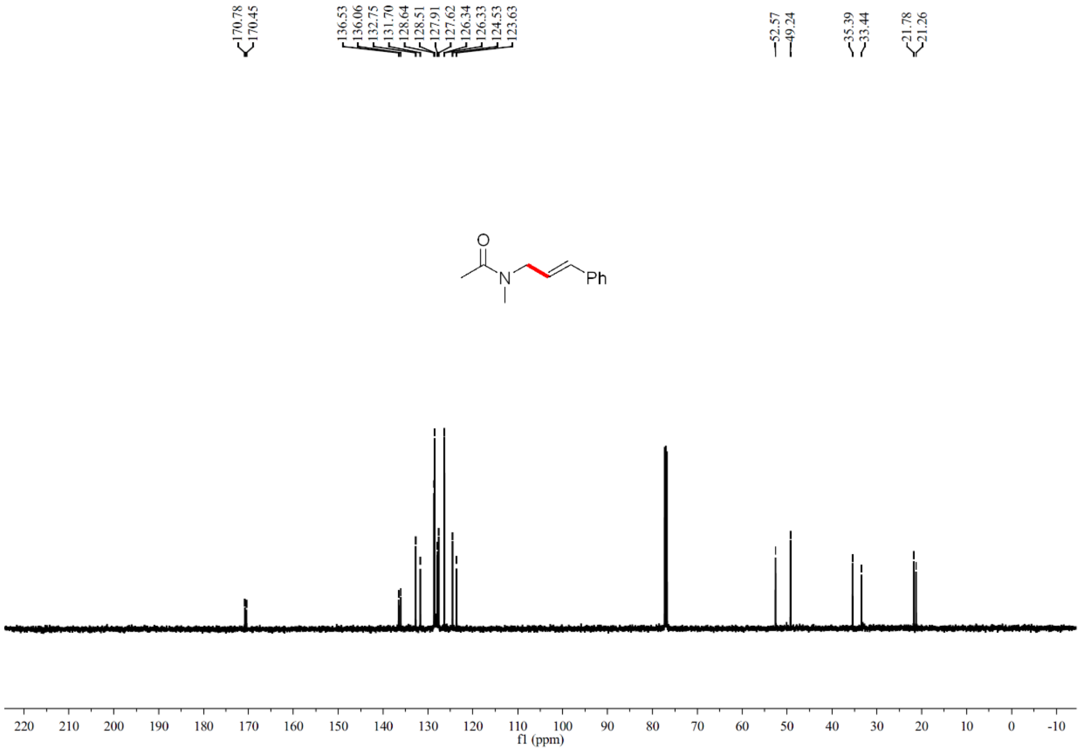

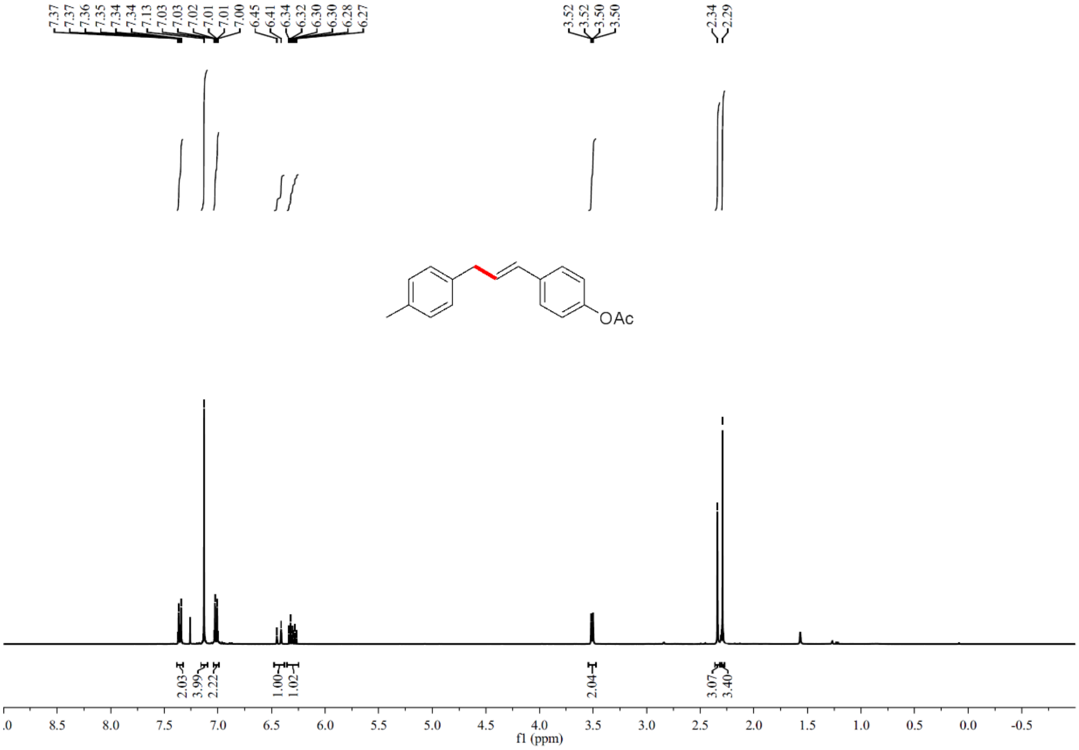
7.37
7.37
7.36
7.35
7.34
7.34
7.13
7.03
7.03
7.02
7.01
7.01
7.00
6.45
6.41
6.34
6.32
6.30
6.30
6.28
6.27
3.52
3.52
3.50
3.50
2.34
2.29
OAc
2.03
3.99
2.22
1.00
1.02
2.04
3.07
3.40
f1 (ppm)

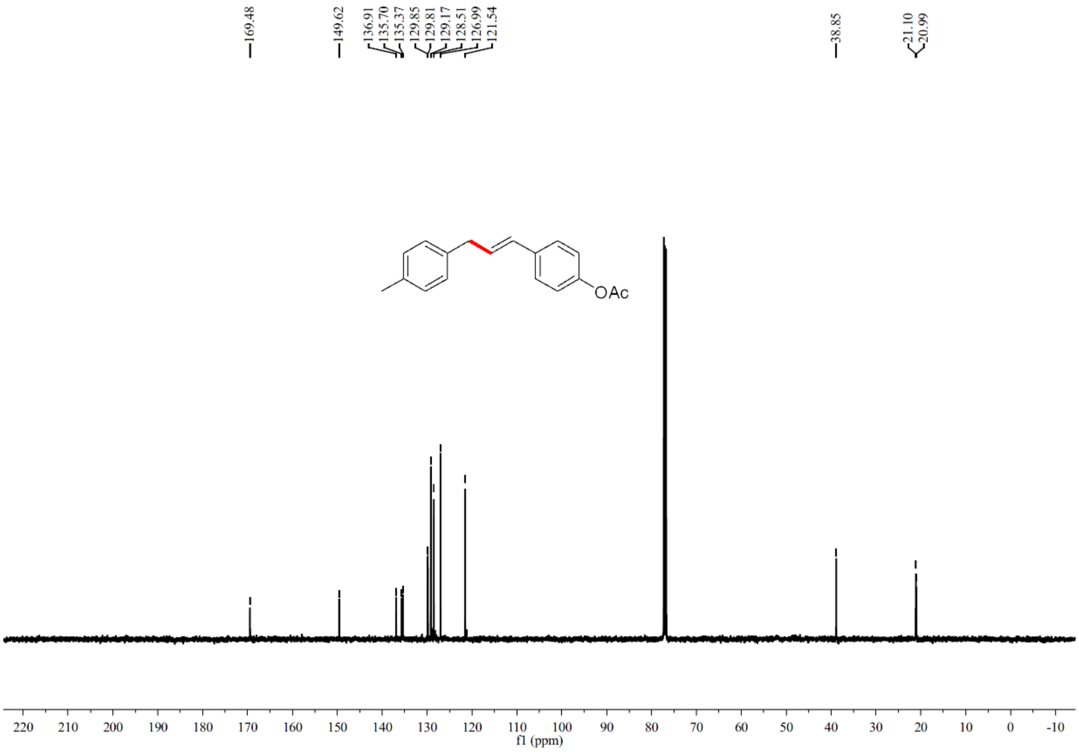
169.48
149.62
136.91
135.70
135.37
129.85
129.81
129.17
128.51
126.99
121.54
38.85
21.10
20.99
OAc
f1 (ppm)

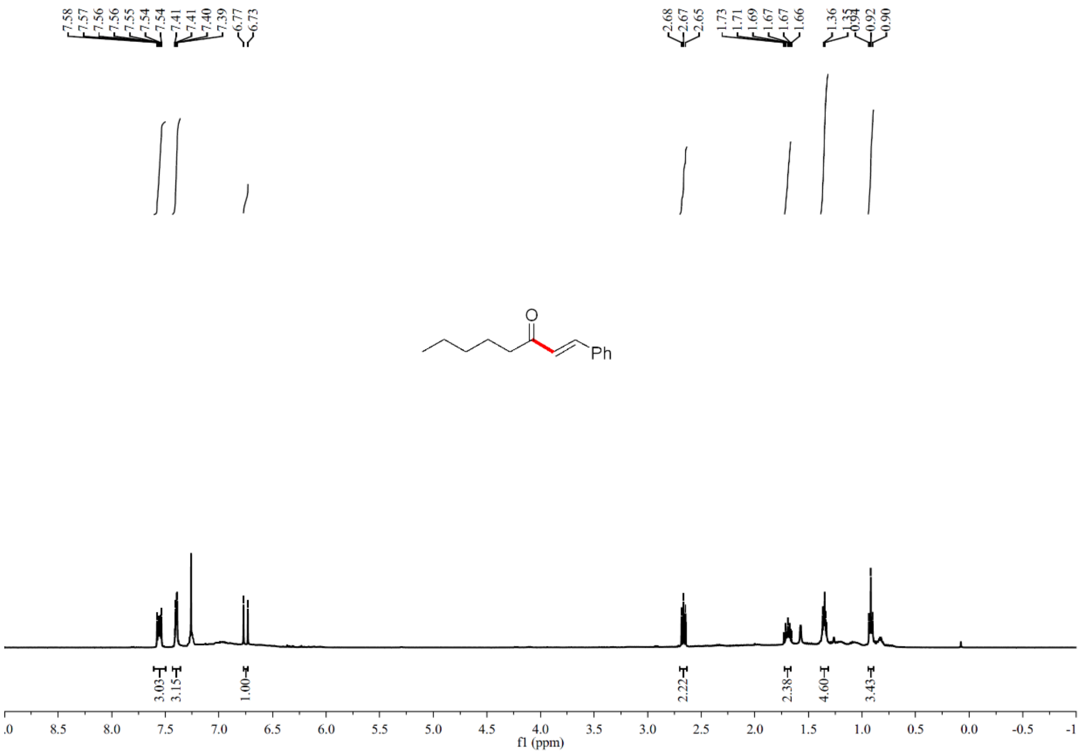
7.58
7.57
7.56
7.55
7.54
7.54
7.41
7.41
7.40
7.39
6.77
6.73
2.68
2.67
2.65
1.73
1.71
1.69
1.67
1.67
1.66
1.36
0.94
0.92
0.90
O
Ph
3.03
3.15
1.00
2.22
2.38
4.60
3.43
f1 (ppm)

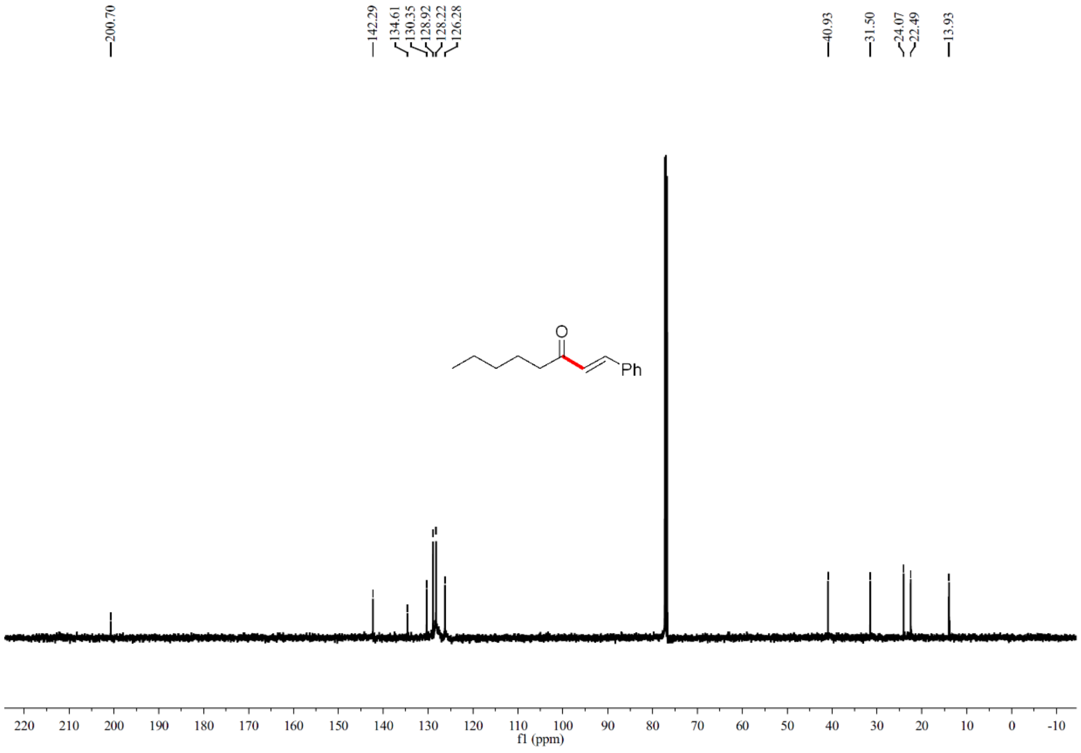
200.70
142.29
134.61
130.35
128.92
128.22
126.28
40.93
31.50
24.07
22.49
13.93
O
Ph
f1 (ppm)

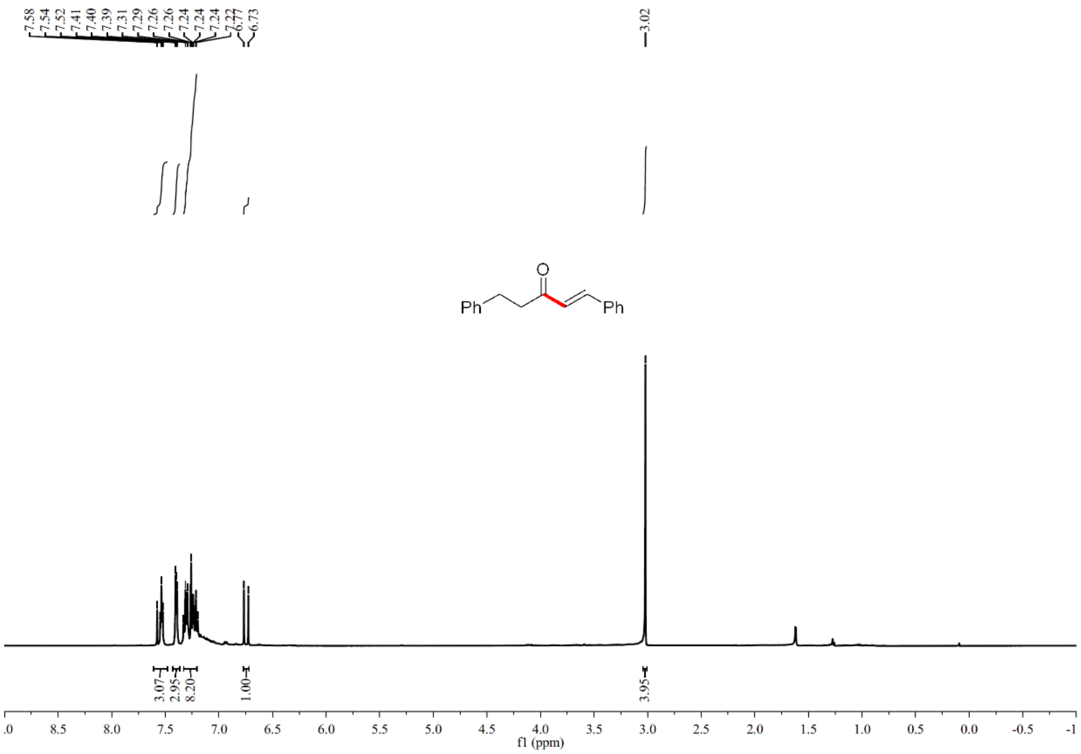
7.58
7.54
7.52
7.41
7.40
7.39
7.31
7.29
7.26
7.26
7.24
7.24
7.24
7.22
6.77
6.73
3.02
Ph
O
Ph
3.07
2.95
8.20
1.00
3.95
f1 (ppm)

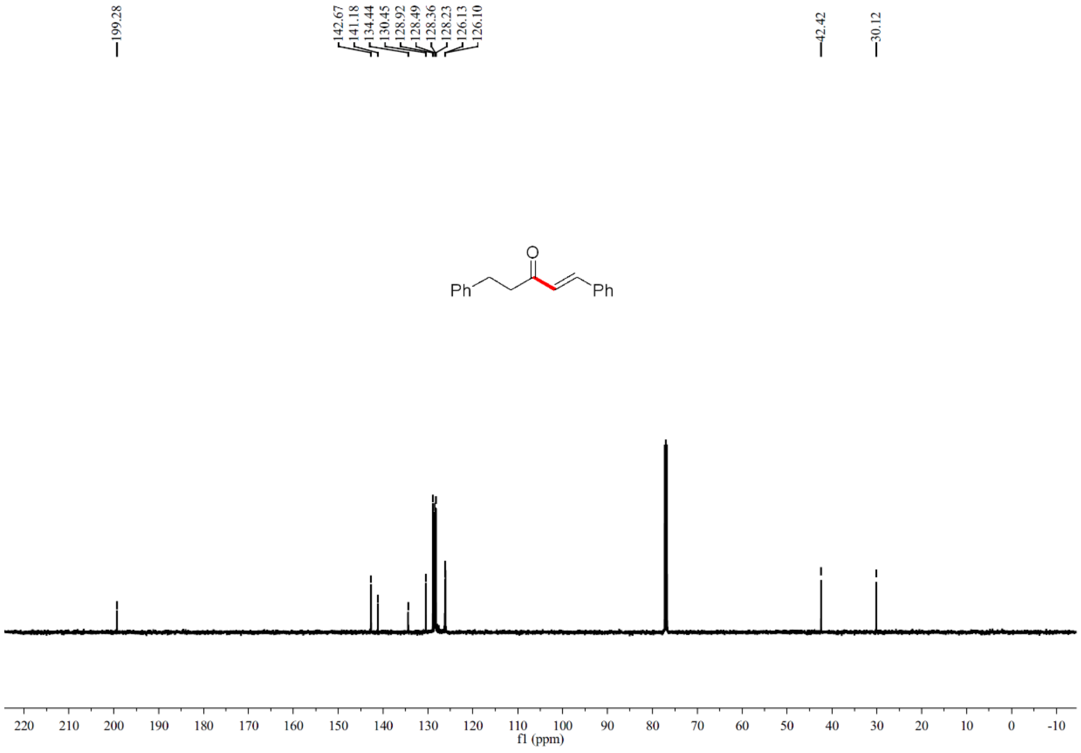
199.28
142.67
141.18
134.44
130.45
128.92
128.49
128.36
128.23
126.13
126.10
42.42
30.12
Ph
O
Ph
f1 (ppm)

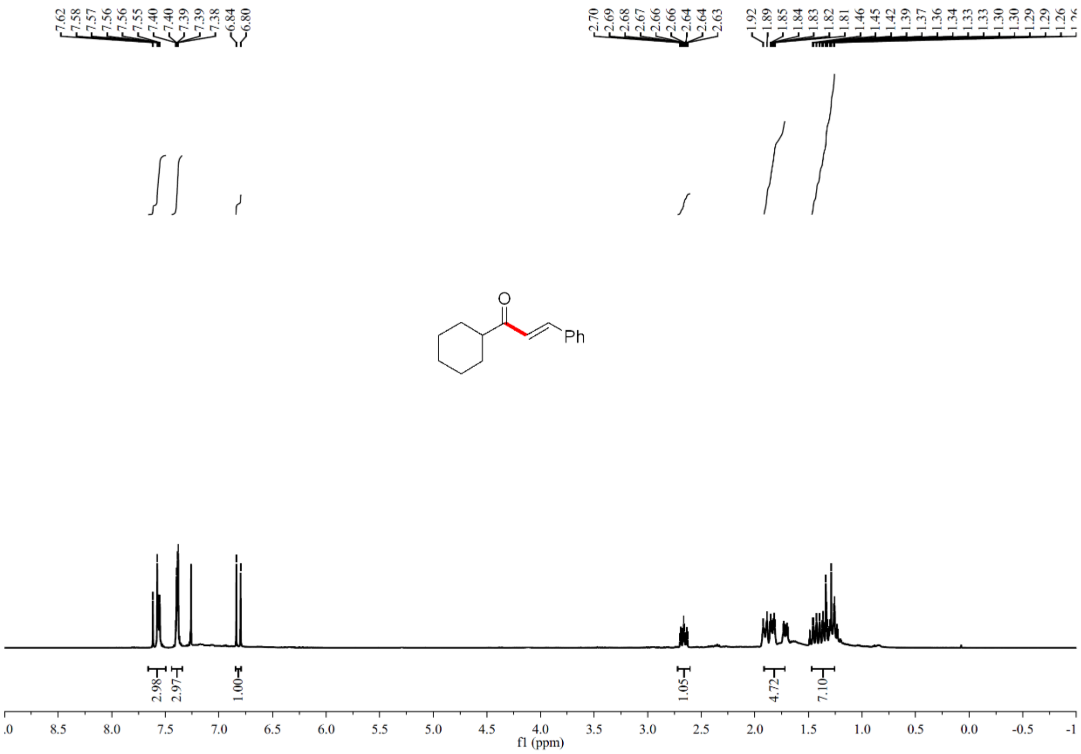
7.62
7.58
7.57
7.56
7.56
7.55
7.40
7.40
7.39
7.39
7.38
6.84
6.80
2.70
2.69
2.68
2.67
2.66
2.66
2.64
2.64
2.63
1.92
1.89
1.85
1.84
1.83
1.82
1.81
1.46
1.45
1.42
1.39
1.37
1.36
1.34
1.33
1.33
1.30
1.30
1.29
1.29
1.26
O
Ph
2.98
2.97
1.00
1.05
4.72
7.10
8.5
8.0
7.5
7.0
6.5
6.0
5.5
5.0
4.5
4.0
3.5
3.0
2.5
2.0
1.5
1.0
0.5
0.0
-0.5
f1 (ppm)

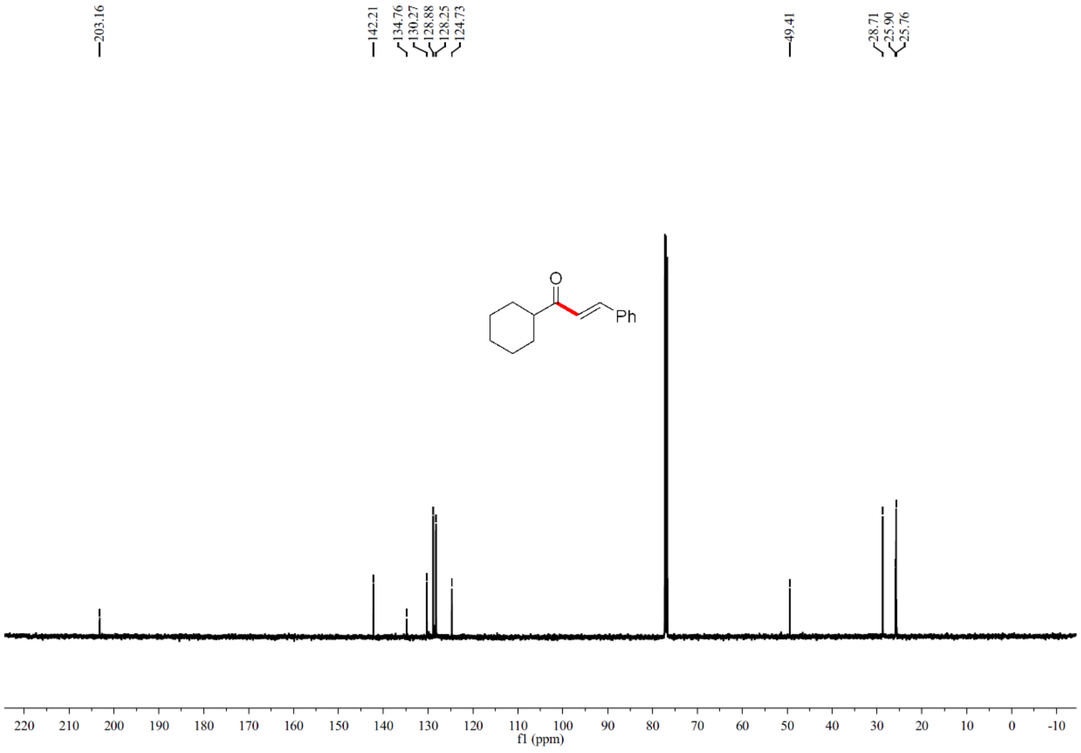
203.16
142.21
134.76
130.27
128.88
128.25
124.73
49.41
28.71
25.90
25.76
O
Ph
220
210
200
190
180
170
160
150
140
130
120
110
100
90
80
70
60
50
40
30
20
10
0
-10
f1 (ppm)

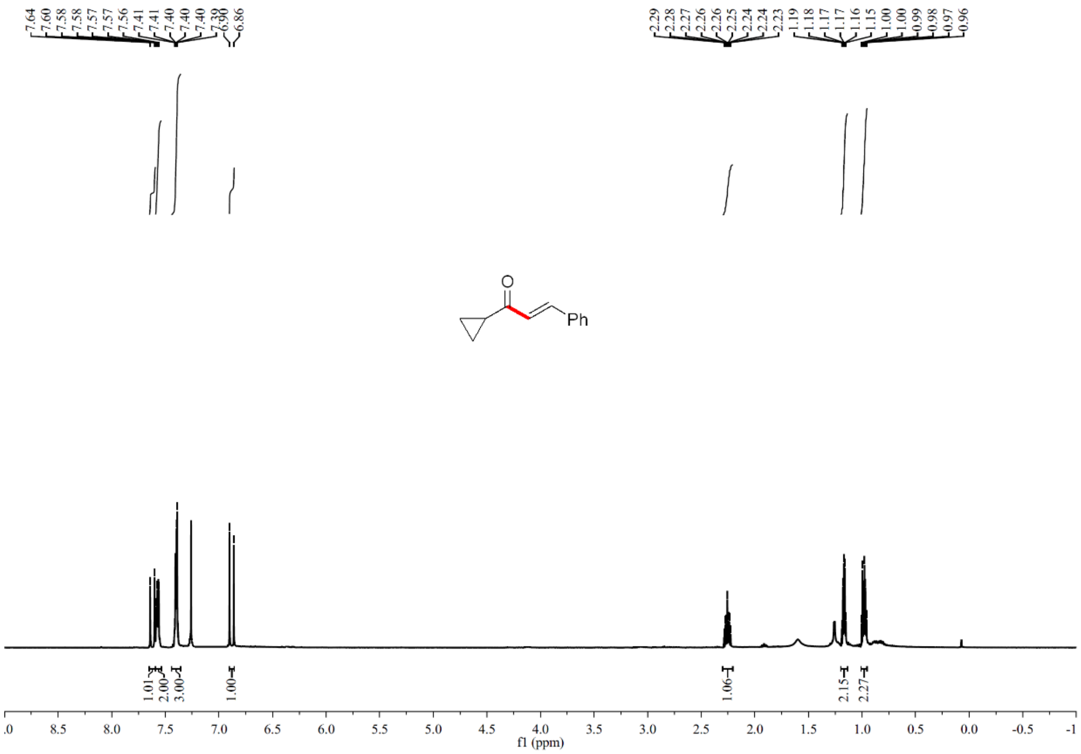

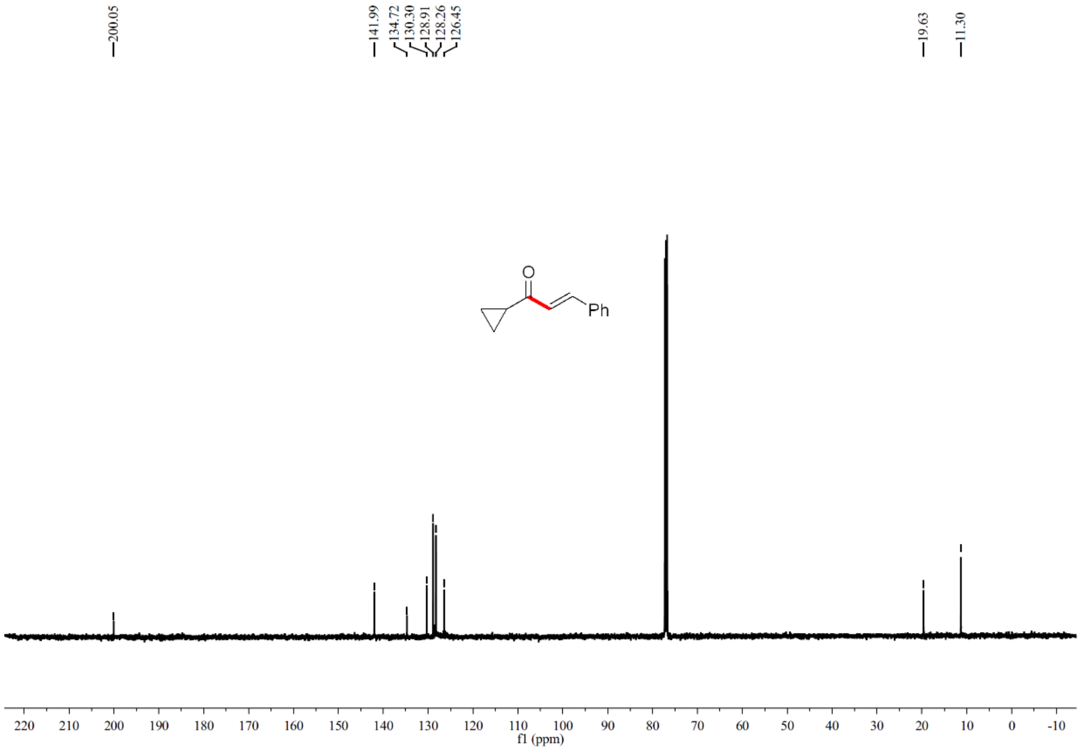

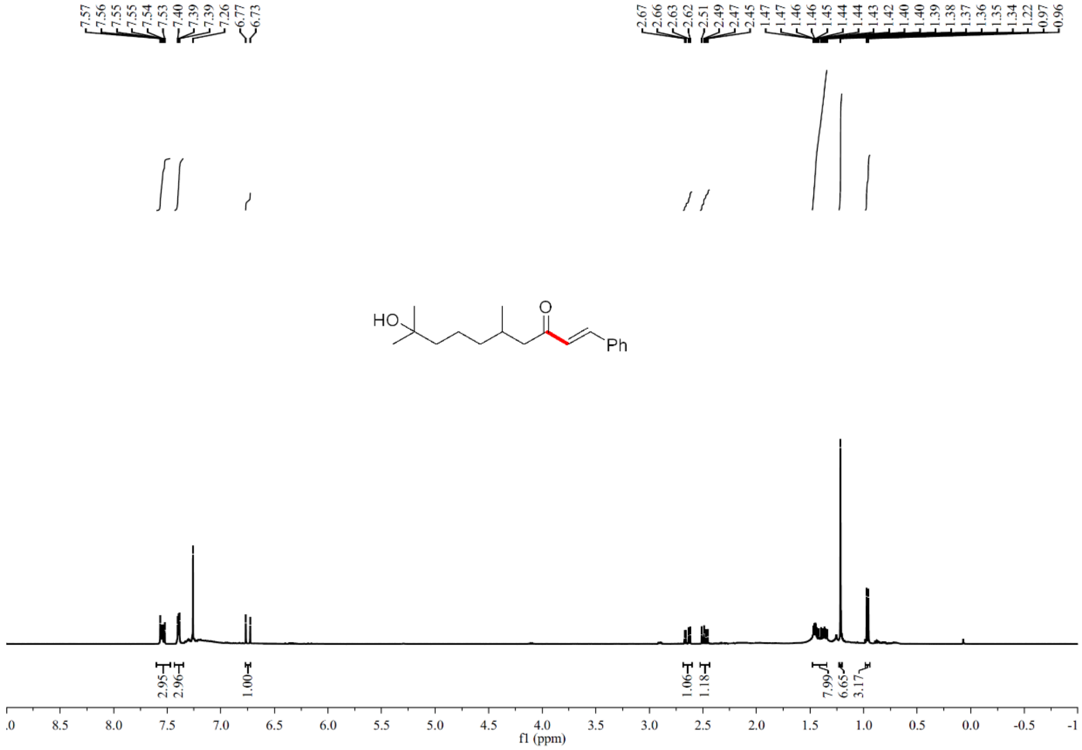

7.57
7.56
7.55
7.55
7.54
7.53
7.40
7.39
7.39
7.26
6.77
6.73
2.67
2.66
2.63
2.62
2.51
2.49
2.47
2.45
1.47
1.47
1.46
1.45
1.44
1.44
1.43
1.42
1.40
1.40
1.39
1.38
1.37
1.36
1.35
1.34
1.22
0.97
0.96
HO
O
Ph
2.95
2.96
1.00
1.06
1.18
7.99
6.65
3.17
f1 (ppm)

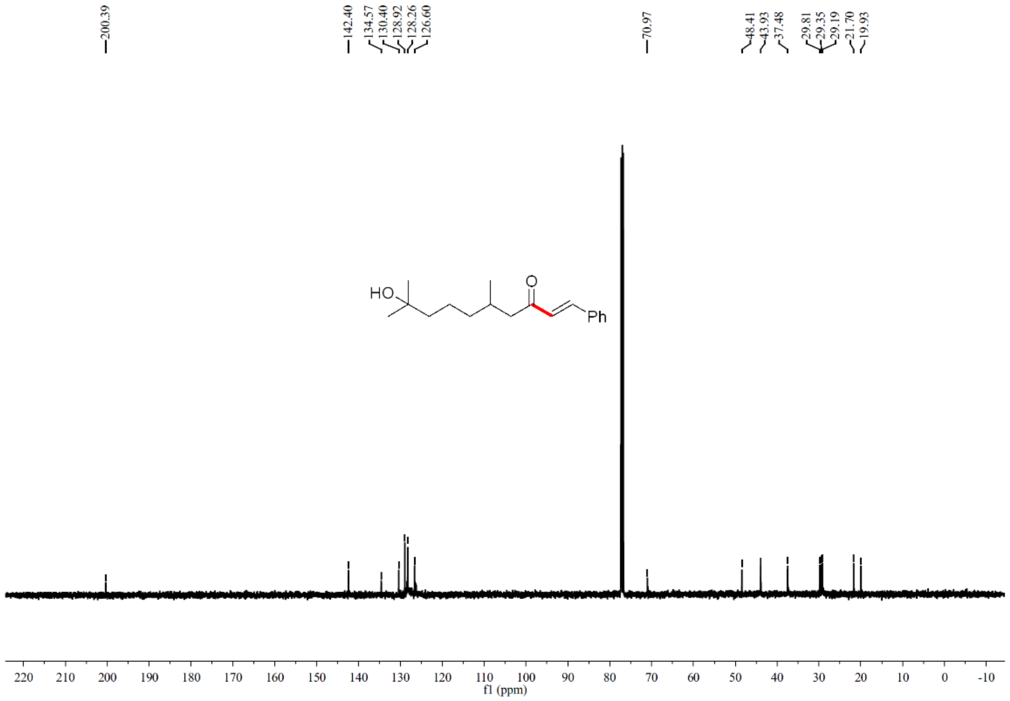

200.39
142.40
134.57
130.40
128.92
128.26
126.60
70.97
48.41
43.93
37.48
29.81
29.35
29.19
21.70
19.93
HO
O
Ph
f1 (ppm)

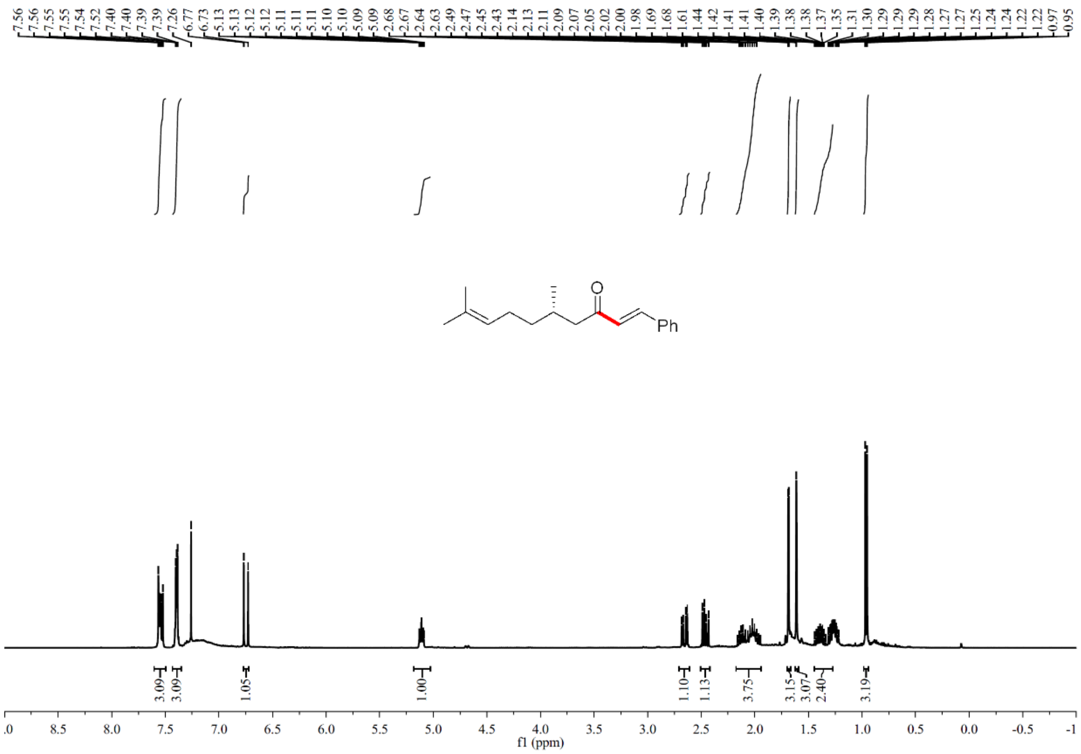

7.56 7.56 7.55 7.55 7.54 7.52 7.40 7.40 7.39 7.39 7.26 6.77 6.73 5.13 5.13 5.12 5.12 5.11 5.11 5.11 5.10 5.10 5.09 5.09 2.68 2.67 2.64 2.63 2.49 2.47 2.45 2.43 2.14 2.13 2.11 2.09 2.07 2.05 2.02 2.00 1.98 1.69 1.68 1.61 1.44 1.42 1.41 1.41 1.40 1.39 1.38 1.38 1.37 1.35 1.31 1.30 1.29 1.29 1.29 1.28 1.27 1.27 1.25 1.24 1.24 1.22 1.22 0.97 0.95
O
Ph
3.09 3.09 1.05 1.00 1.10 1.13 3.75 3.15 3.07 2.40 3.19
f1 (ppm)

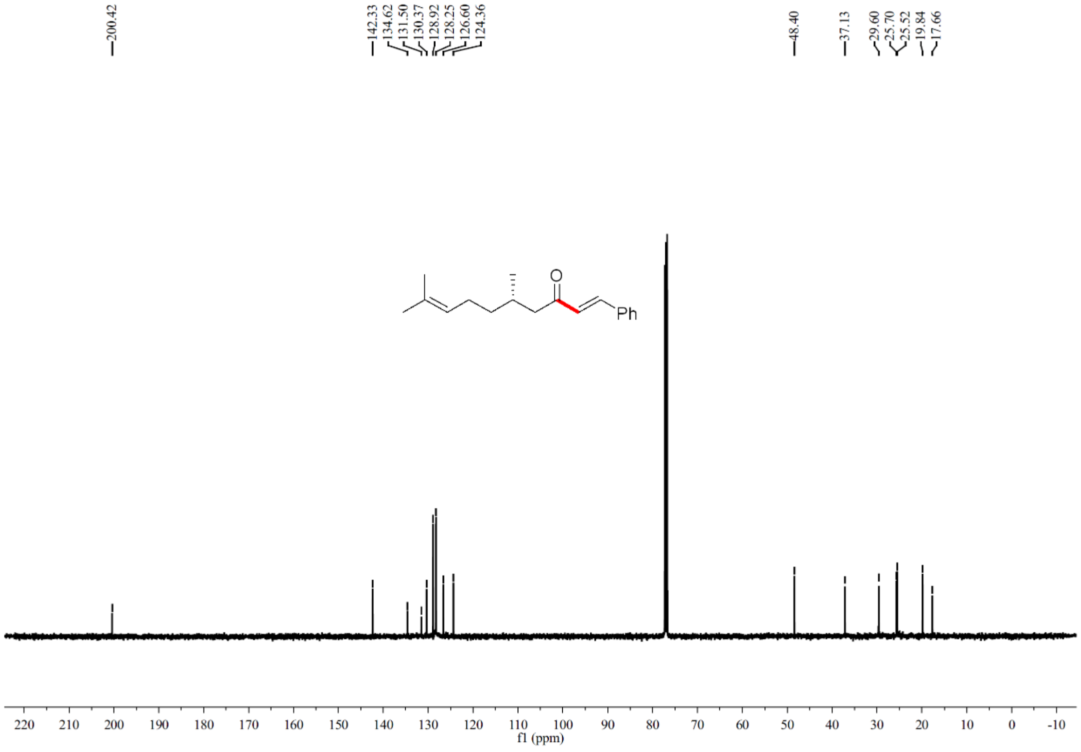

200.42
142.33 134.62 131.50 130.37 128.92 128.25 126.60 124.36
48.40
37.13 29.60 25.70 25.52 19.84 17.66
O
Ph
f1 (ppm)

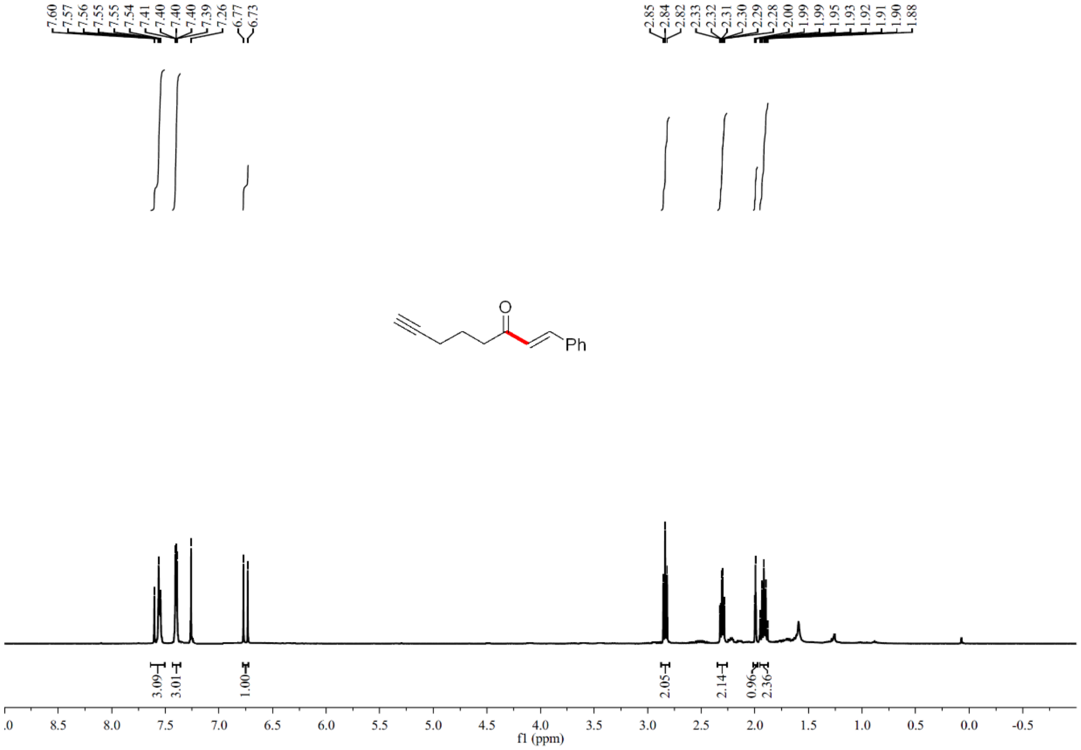
7.60
7.57
7.56
7.55
7.55
7.54
7.41
7.40
7.40
7.40
7.39
7.26
6.77
6.73
2.85
2.84
2.82
2.33
2.32
2.31
2.30
2.29
2.28
2.00
1.99
1.99
1.95
1.93
1.92
1.91
1.90
1.88
O
Ph
3.09
3.01
1.00
2.05
2.14
0.96
2.36
f1 (ppm)

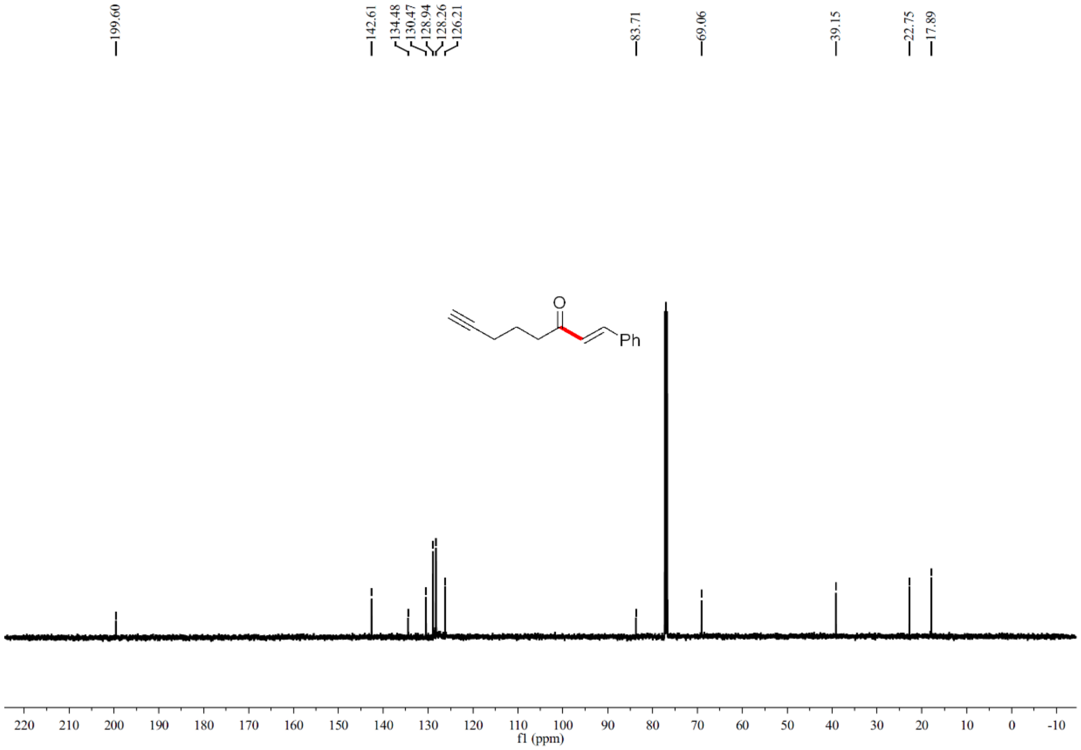
199.60
142.61
134.48
130.47
128.94
128.26
126.21
83.71
69.06
39.15
22.75
17.89
O
Ph
f1 (ppm)

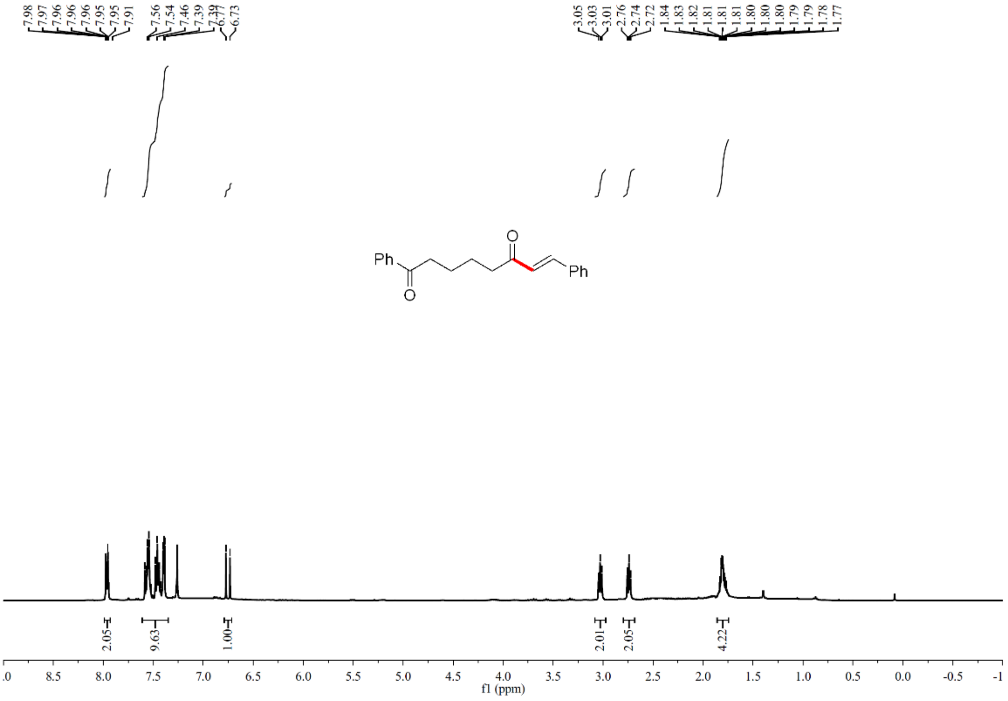

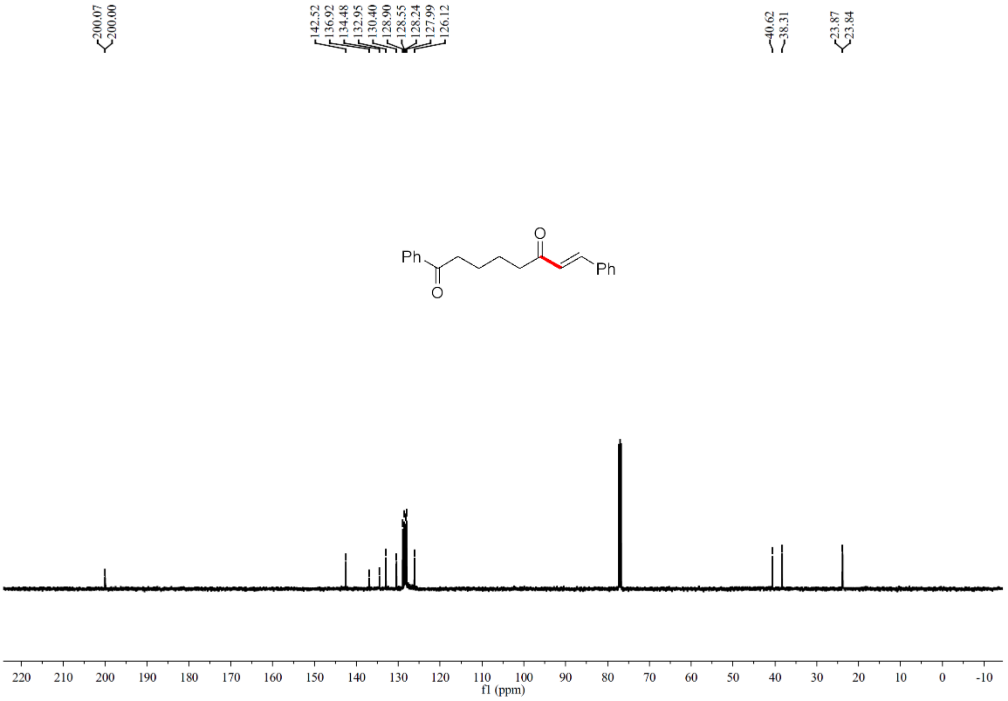

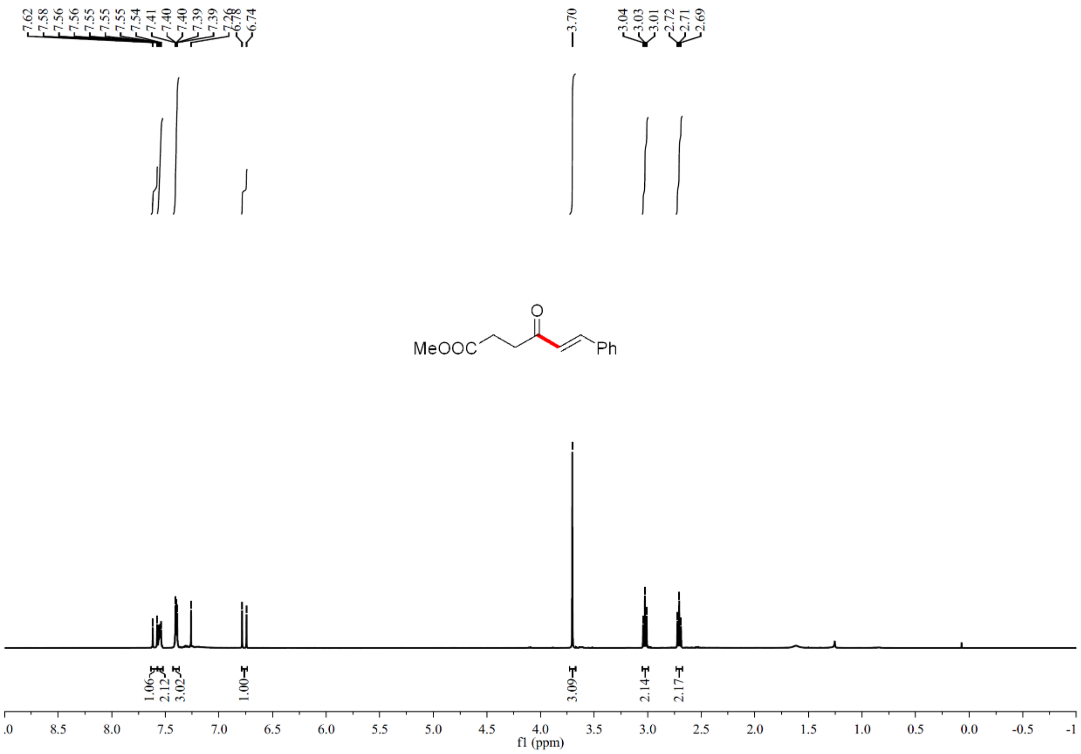

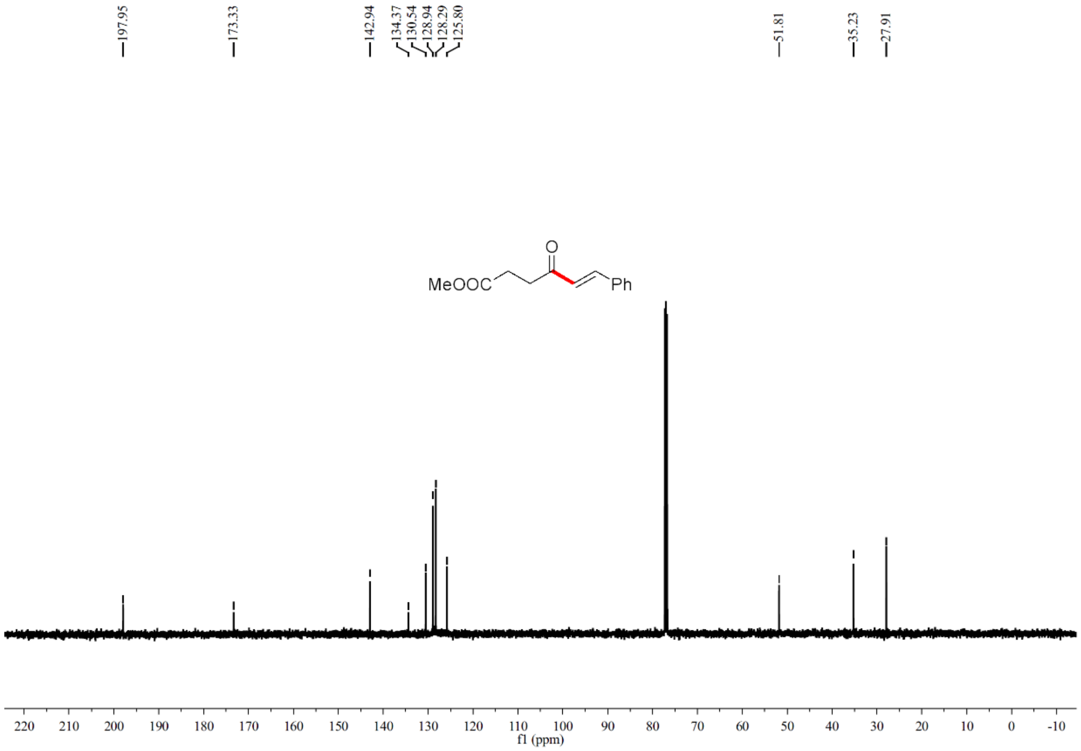

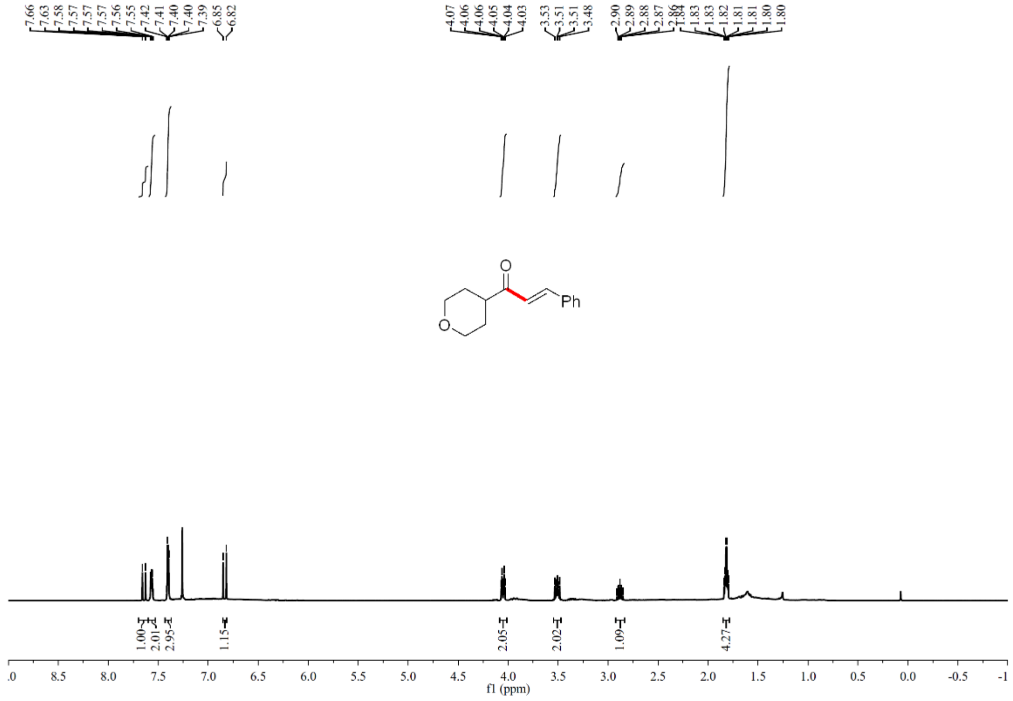
7.66
7.63
7.58
7.57
7.57
7.57
7.56
7.55
7.42
7.41
7.40
7.40
7.39
6.85
6.82
4.07
4.06
4.06
4.05
4.04
4.03
3.53
3.51
3.51
3.48
2.90
2.89
2.88
2.87
1.83
1.83
1.82
1.81
1.81
1.80
1.80
O
Ph
1.00
2.01
2.95
1.15
2.05
2.02
1.09
4.27
f1 (ppm)

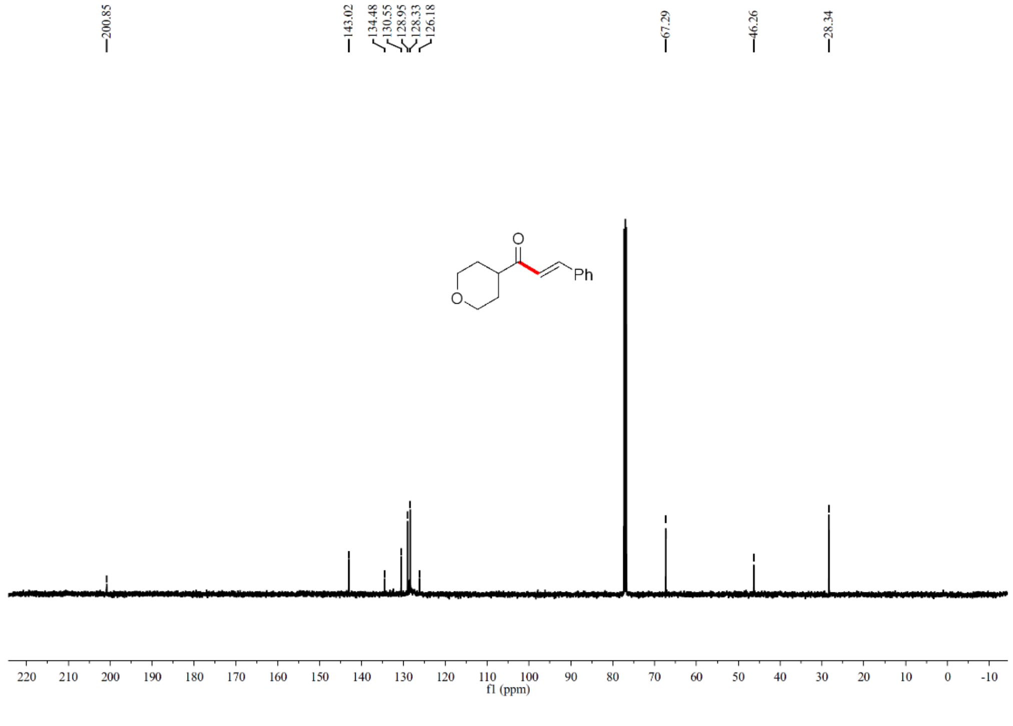
200.85
143.02
134.48
130.55
128.95
128.33
126.18
67.29
46.26
28.34
O
Ph
f1 (ppm)

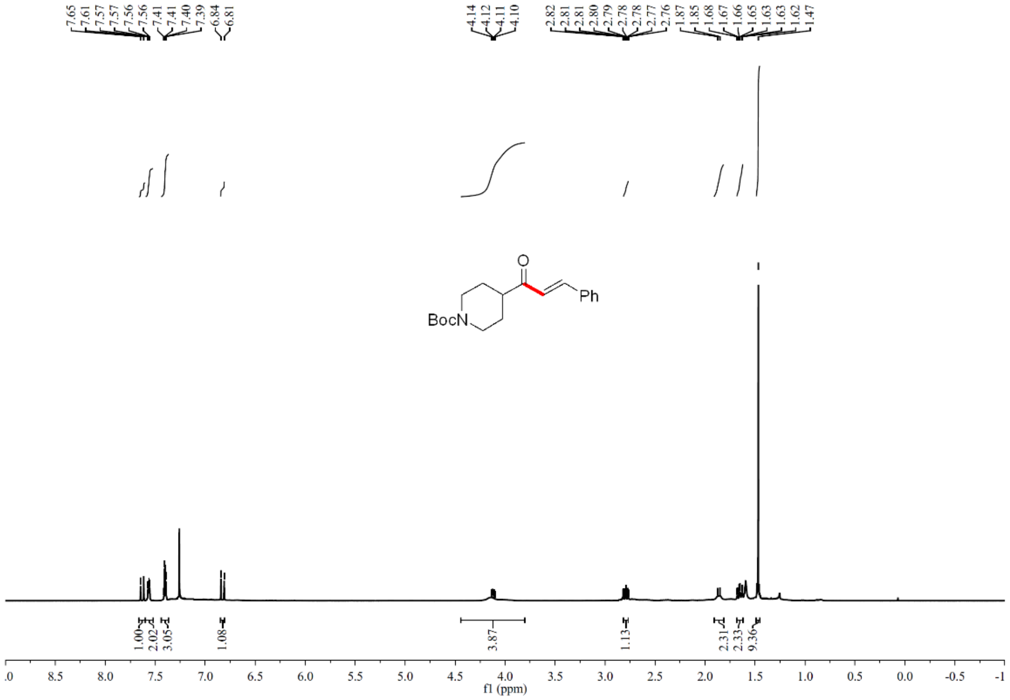
7.65
7.61
7.57
7.57
7.56
7.56
7.41
7.41
7.40
7.39
6.84
6.81
4.14
4.12
4.11
4.10
2.82
2.81
2.81
2.80
2.79
2.78
2.78
2.77
2.76
1.87
1.85
1.68
1.67
1.66
1.65
1.63
1.63
1.62
1.47
O
Ph
BocN
1.00
2.02
3.05
1.08
3.87
1.13
2.31
2.33
9.36
f1 (ppm)

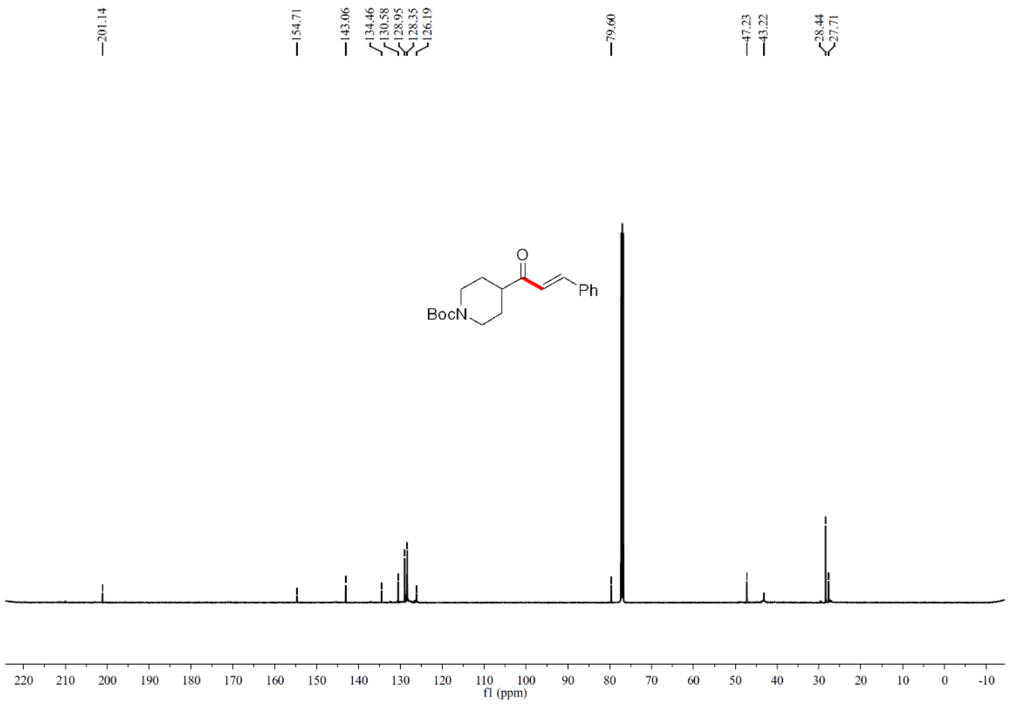
201.14
154.71
143.06
134.46
130.58
128.95
128.35
126.19
79.60
47.23
43.22
28.44
27.71
O
Ph
BocN
f1 (ppm)

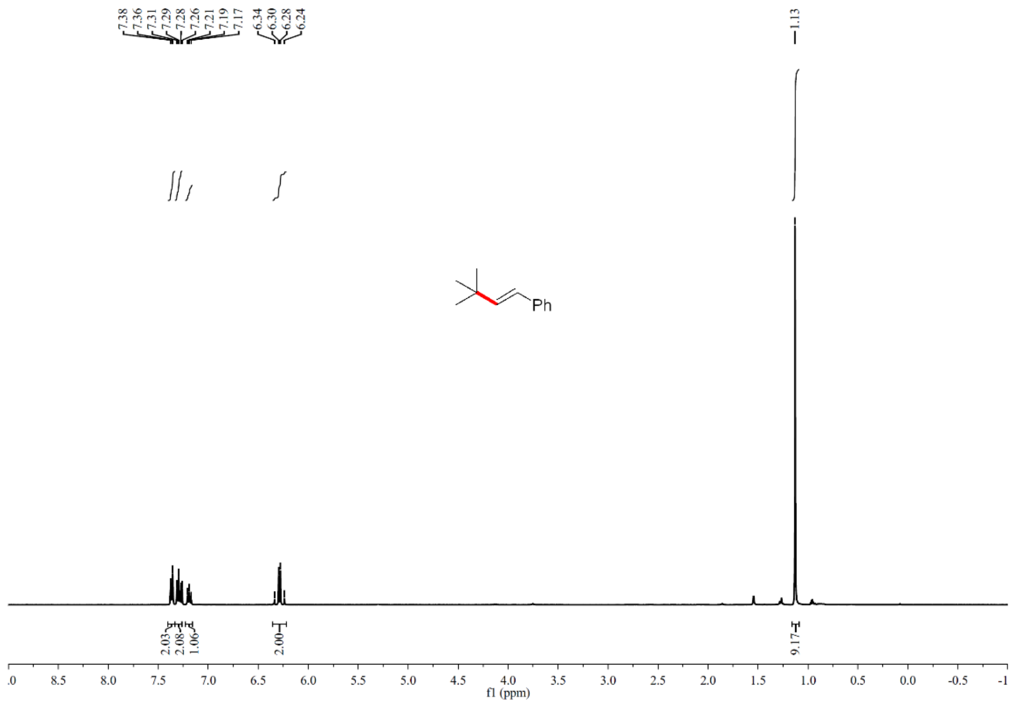
7.38
7.36
7.31
7.29
7.28
7.26
7.21
7.19
7.17
6.34
6.30
6.28
6.24
1.13
Ph
2.03
2.08
1.06
2.00
9.17
.0
8.5
8.0
7.5
7.0
6.5
6.0
5.5
5.0
4.5
4.0
3.5
3.0
2.5
2.0
1.5
1.0
0.5
0.0
-0.5
-1
f1 (ppm)

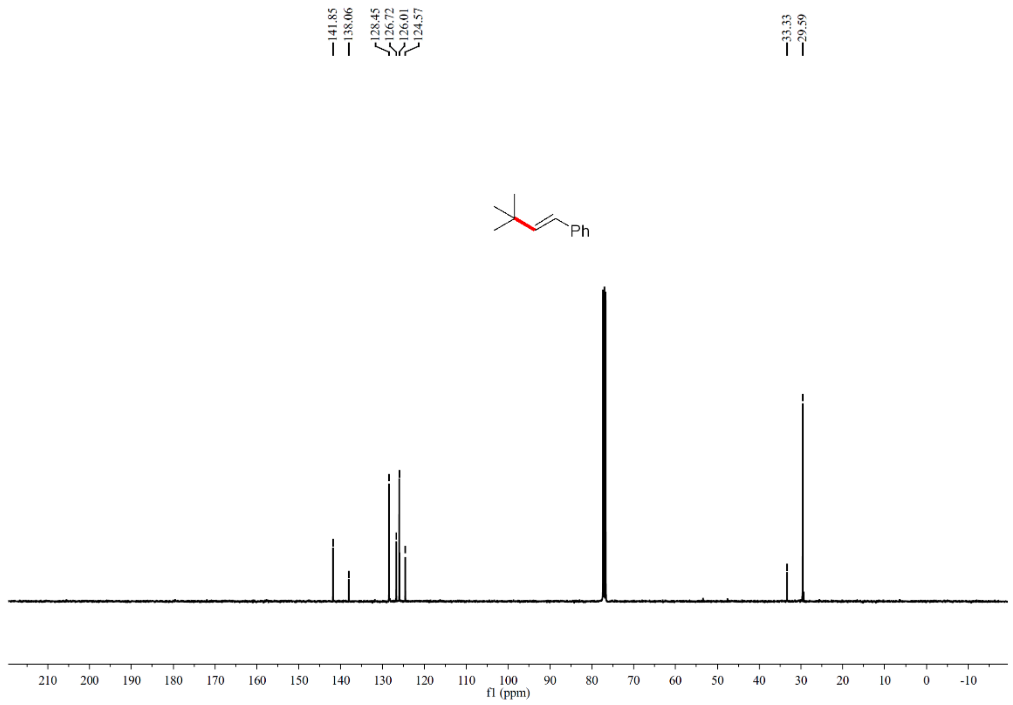
141.85
138.06
128.45
126.72
126.01
124.57
33.33
29.59
Ph
210
200
190
180
170
160
150
140
130
120
110
100
90
80
70
60
50
40
30
20
10
0
-10
f1 (ppm)

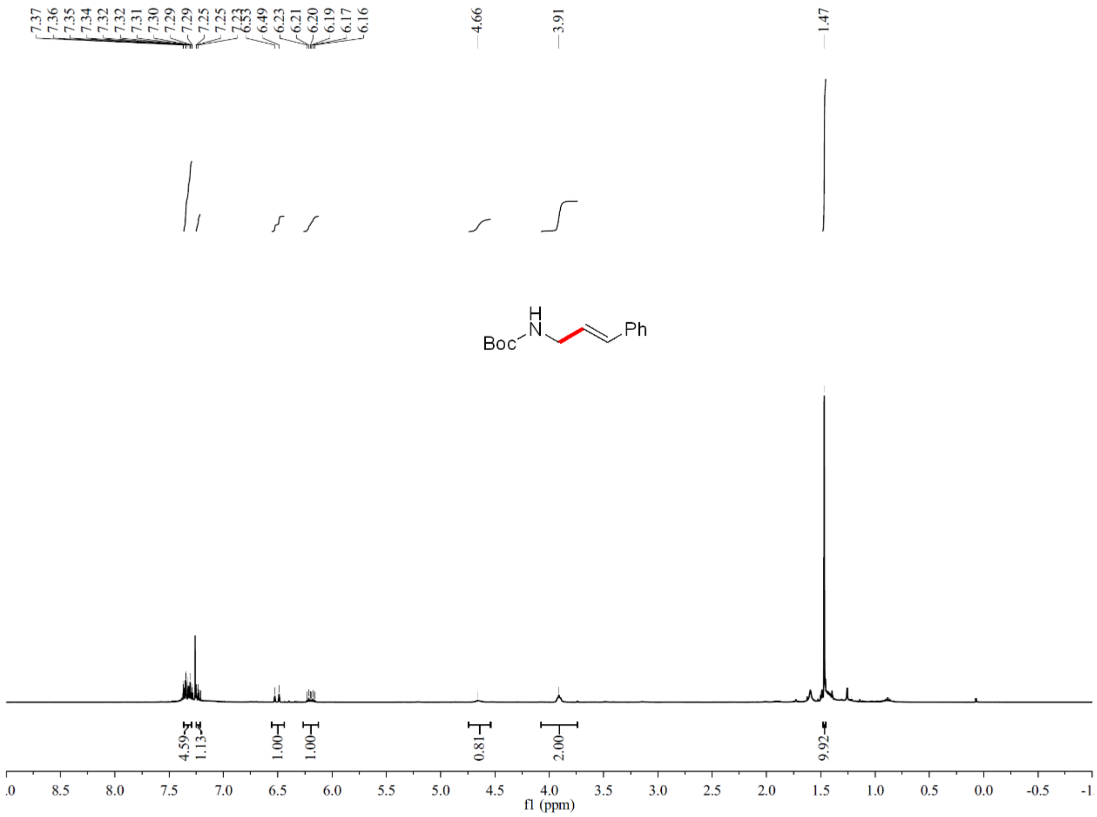
7.37
7.36
7.35
7.34
7.32
7.32
7.31
7.30
7.29
7.29
7.25
7.25
7.23
6.53
6.49
6.23
6.21
6.20
6.19
6.17
6.16
4.66
3.91
1.47
Boc
H
N
Ph
4.59
1.13
1.00
1.00
0.81
2.00
9.92
f1 (ppm)

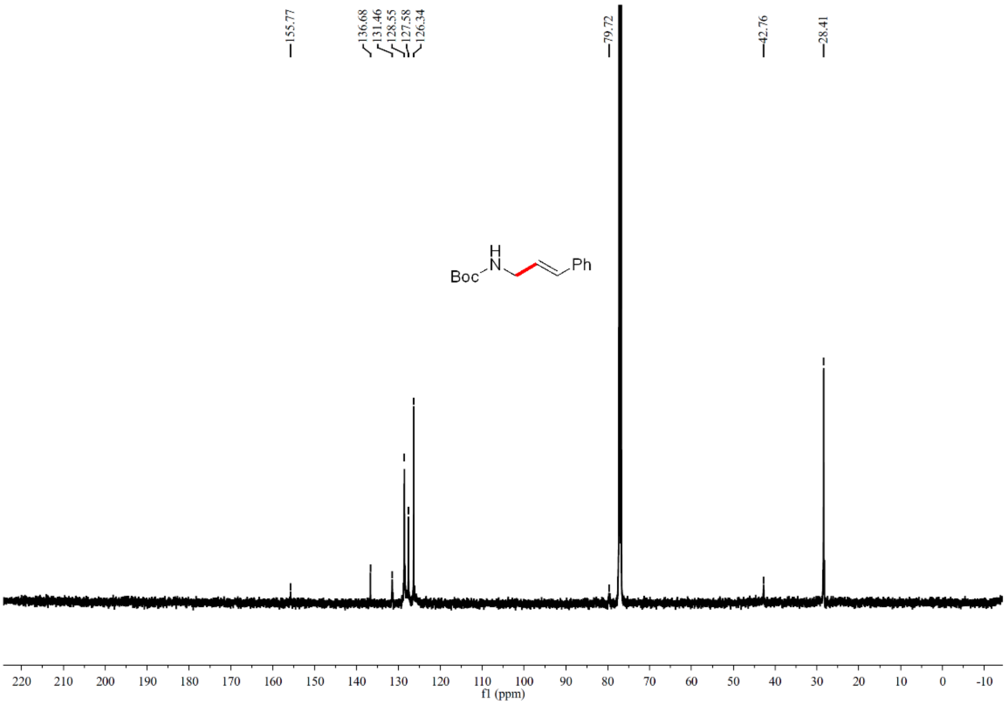
155.77
136.68
131.46
128.55
127.58
126.34
79.72
42.76
28.41
Boc
H
N
Ph
f1 (ppm)

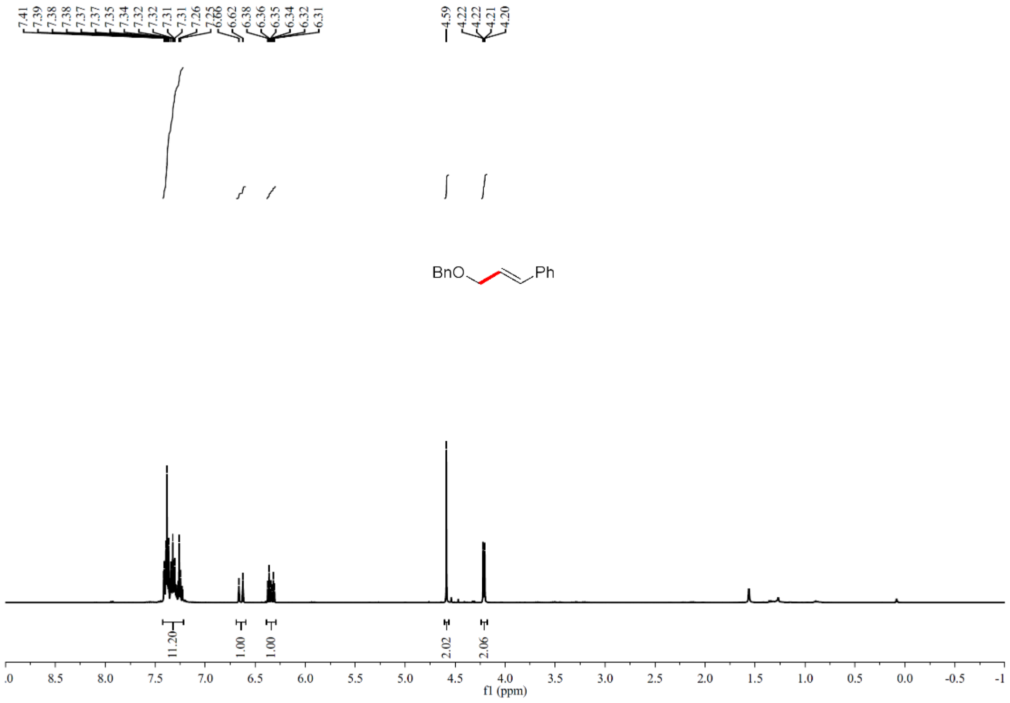
7.41 7.39 7.38 7.38 7.37 7.37 7.35 7.34 7.32 7.32 7.31 7.31 7.26 7.25 6.66 6.62 6.38 6.36 6.35 6.34 6.32 6.31
4.59 4.22 4.22 4.21 4.20
BnO
Ph
11.20 1.00 1.00 2.02 2.06
f1 (ppm)

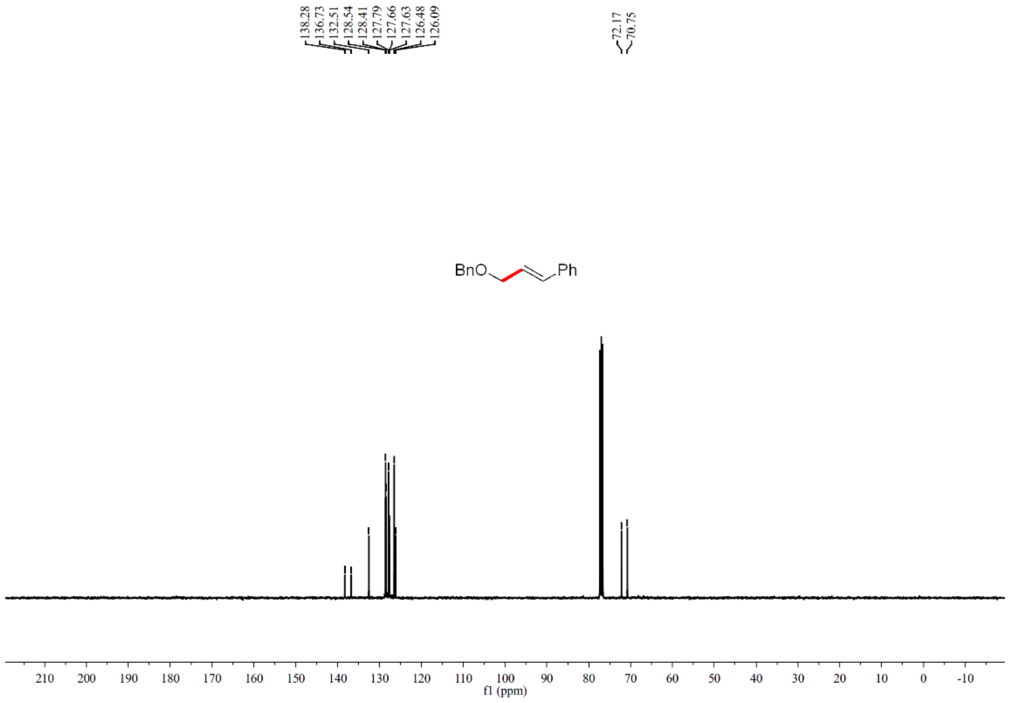
138.28 136.73 132.51 128.54 128.41 127.79 127.66 127.63 126.48 126.09
72.17 70.75
BnO
Ph
f1 (ppm)

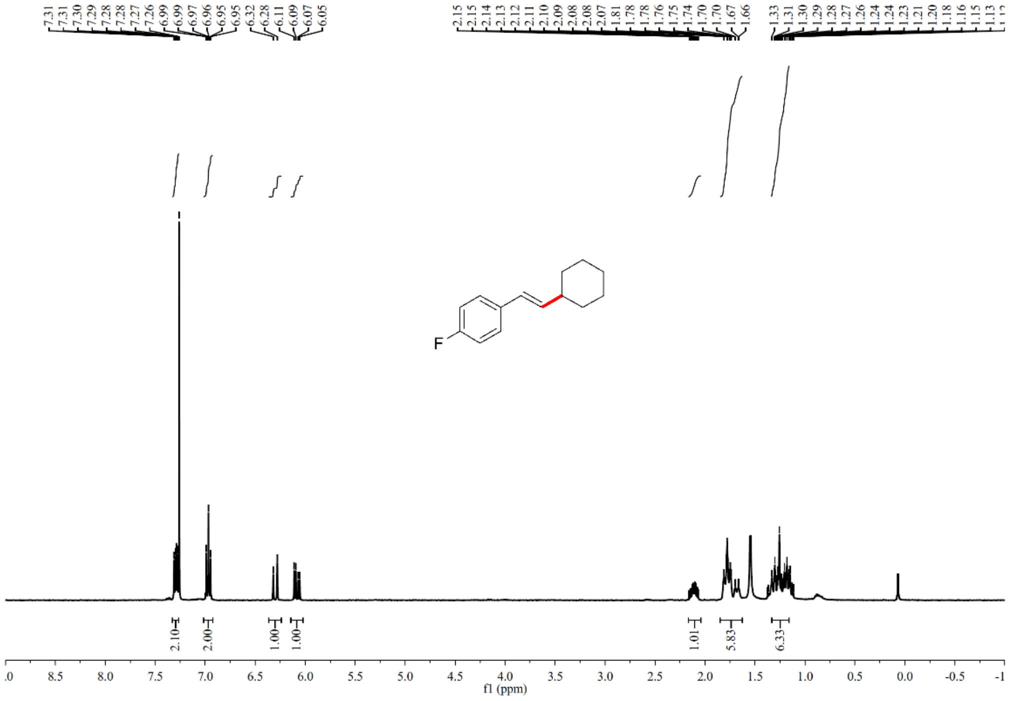

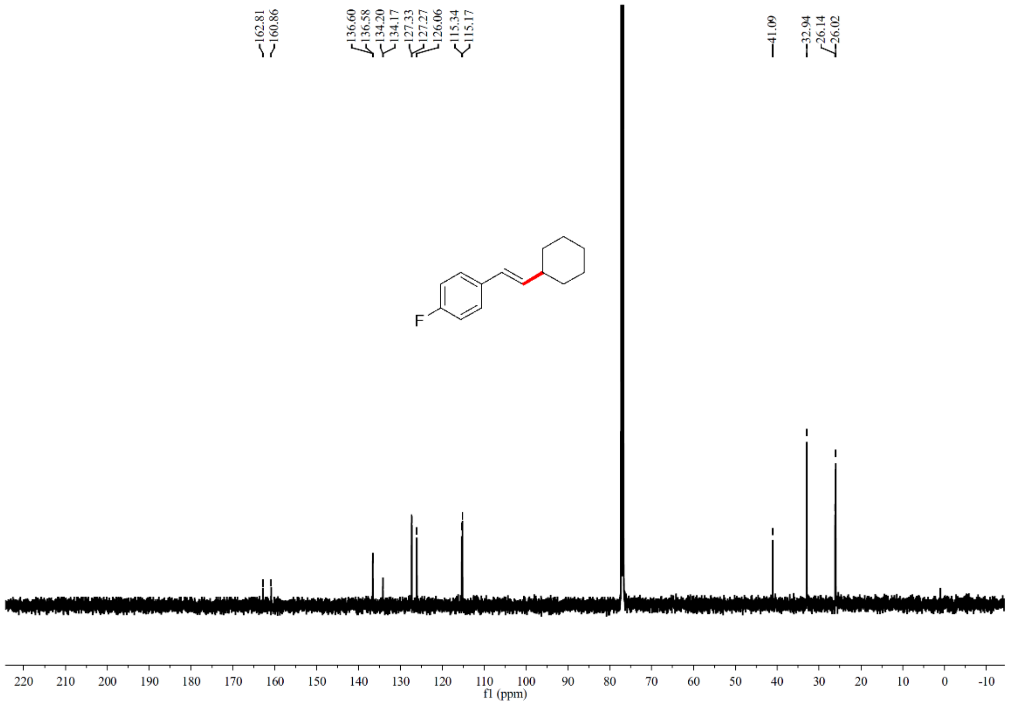

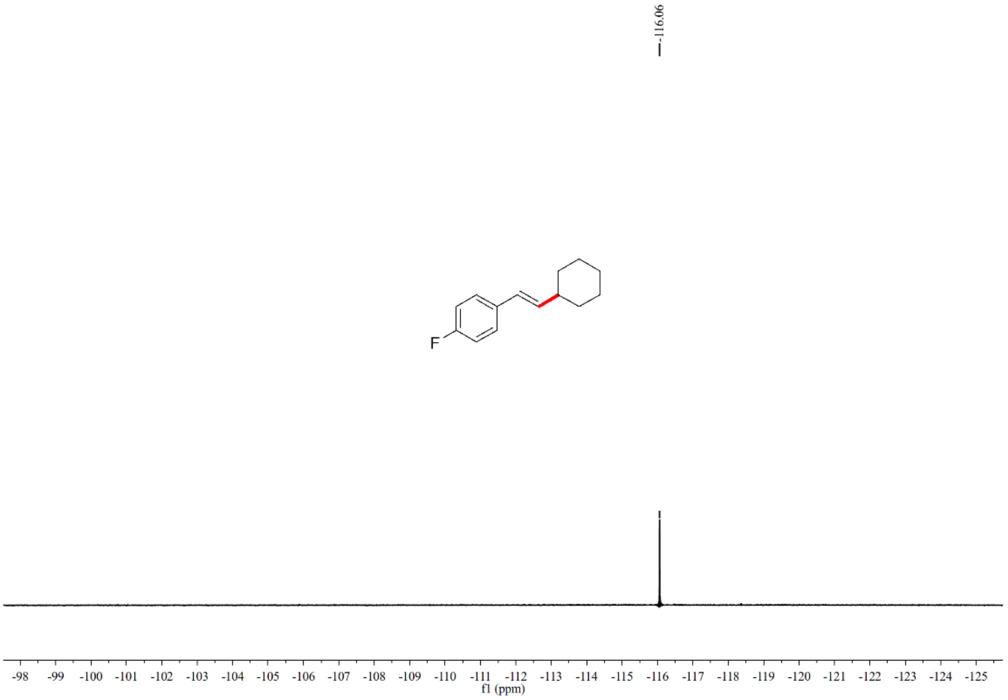

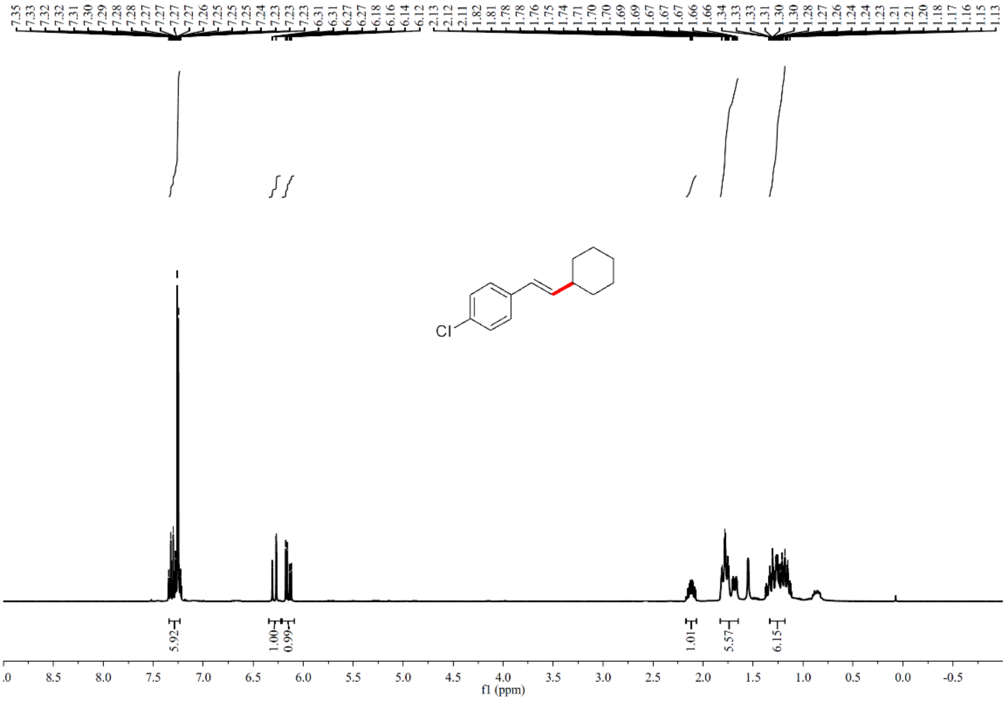

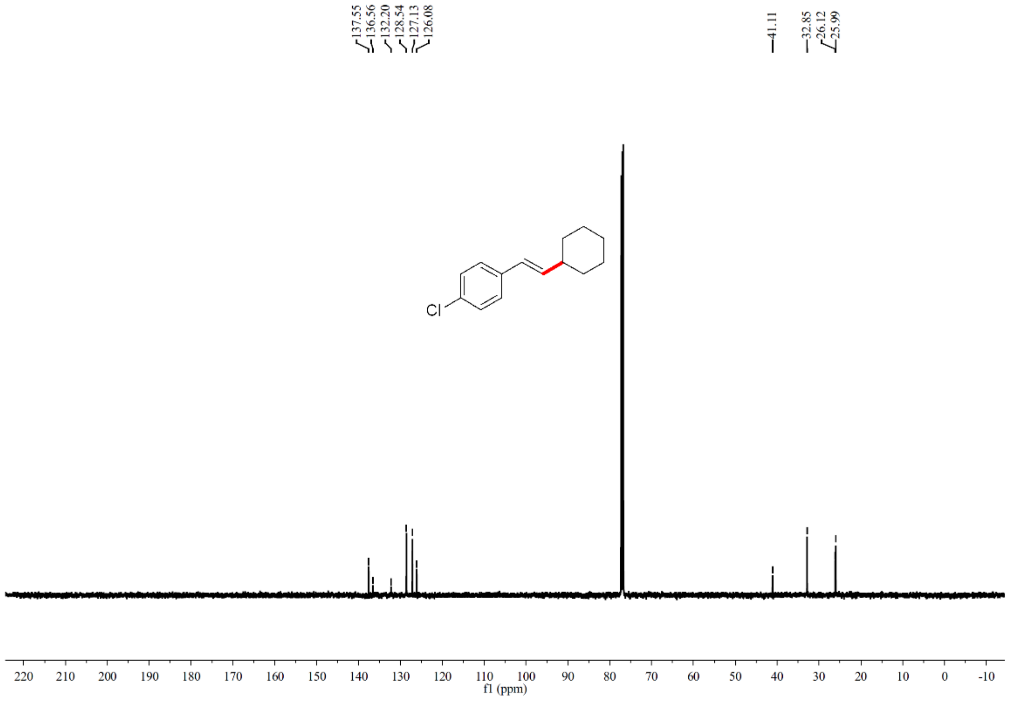

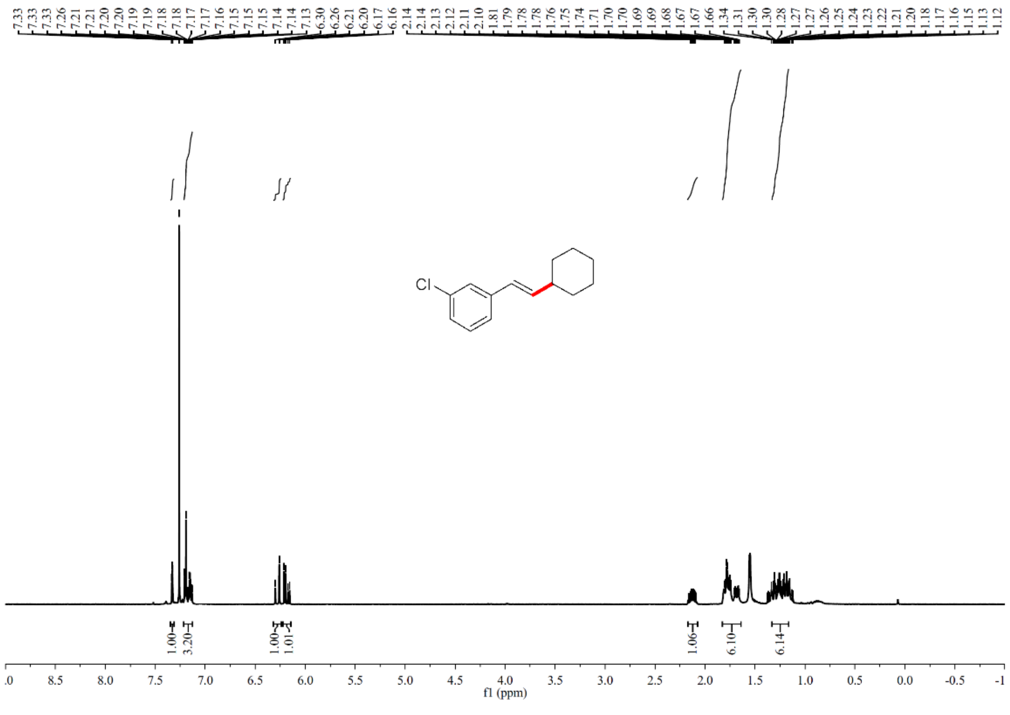

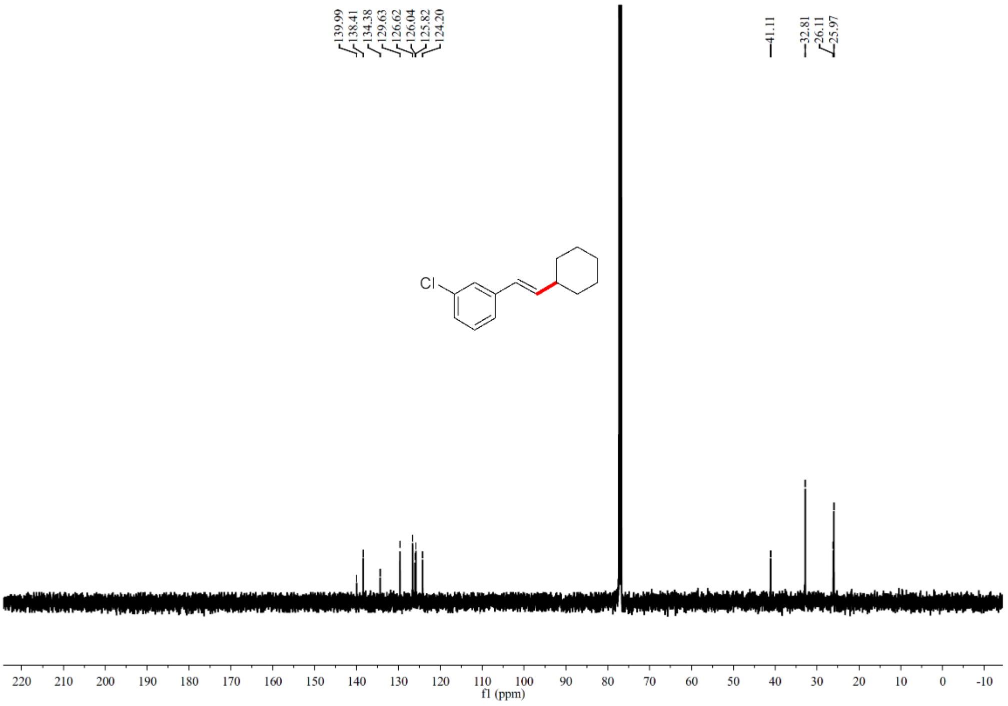
139.99
138.41
134.38
129.63
126.62
126.04
125.82
124.20
41.11
32.81
26.11
25.97
Cl
f1 (ppm)

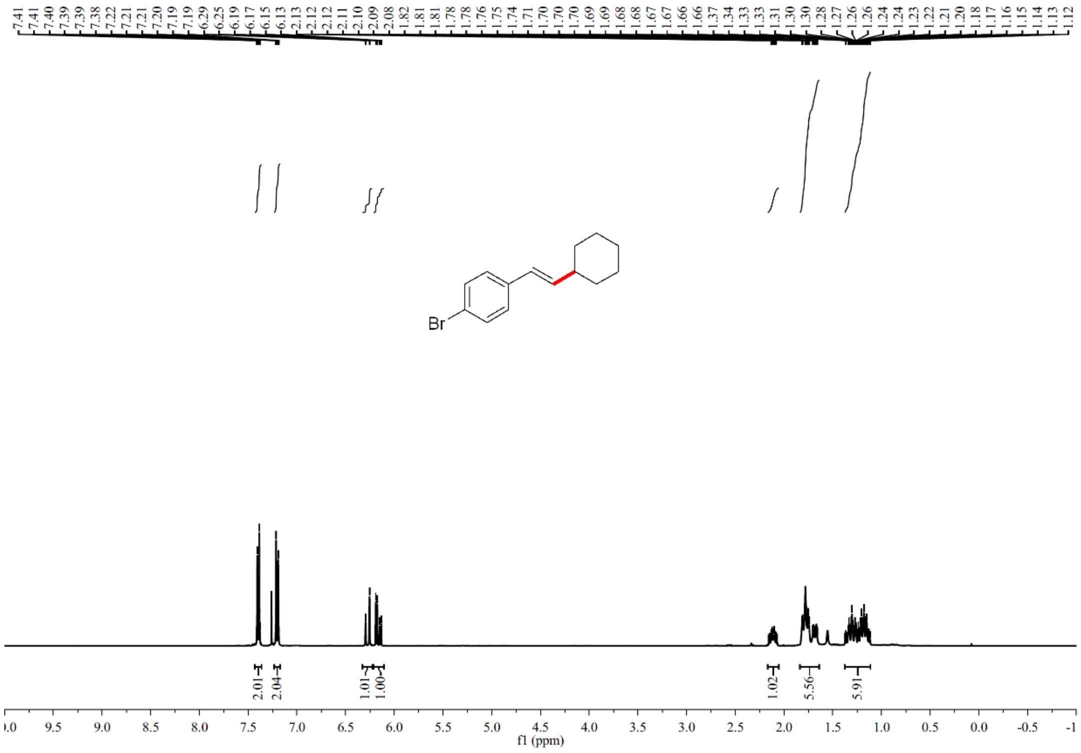
Br
2.01
2.04
1.01
1.00
1.02
5.56
5.91
f1 (ppm)

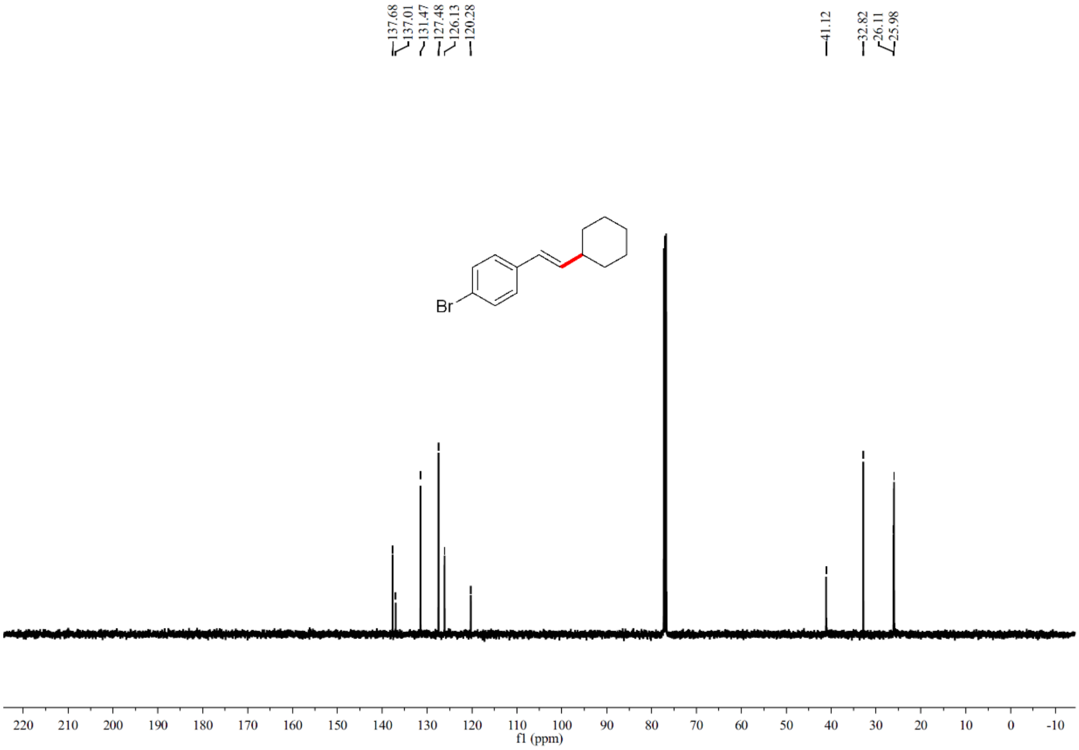

137.68
137.01
131.47
127.48
126.13
120.28
41.12
32.82
26.11
25.98
Br
220 210 200 190 180 170 160 150 140 130 120 110 100 90 80 70 60 50 40 30 20 10 0 -10
f1 (ppm)

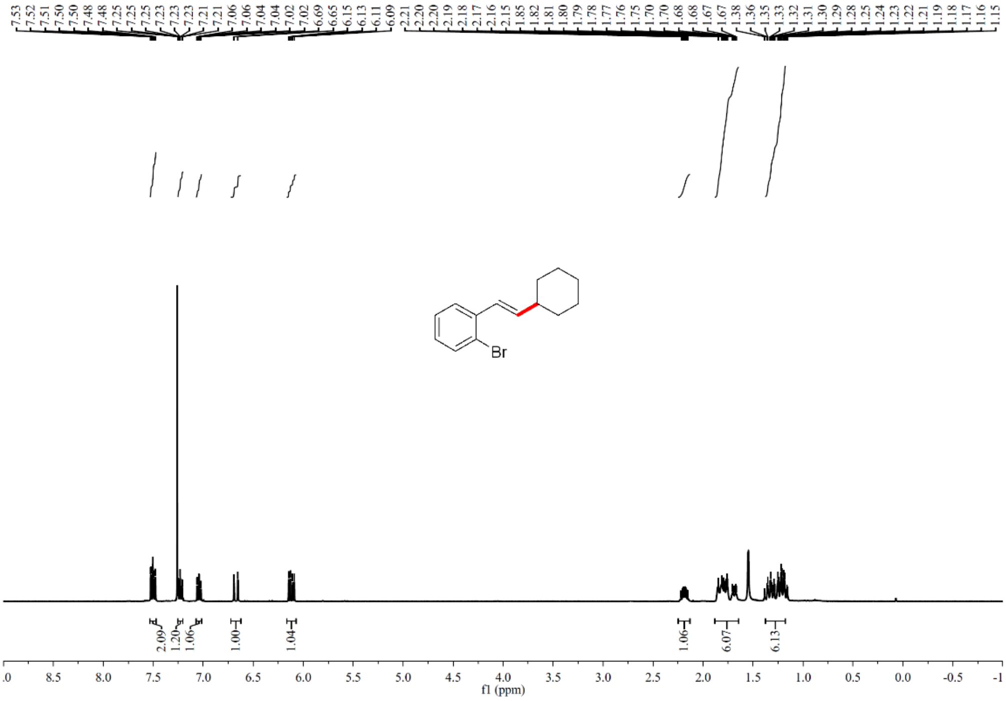

Br
2.09
1.20
1.06
1.00
1.04
1.06
6.07
6.13
f1 (ppm)

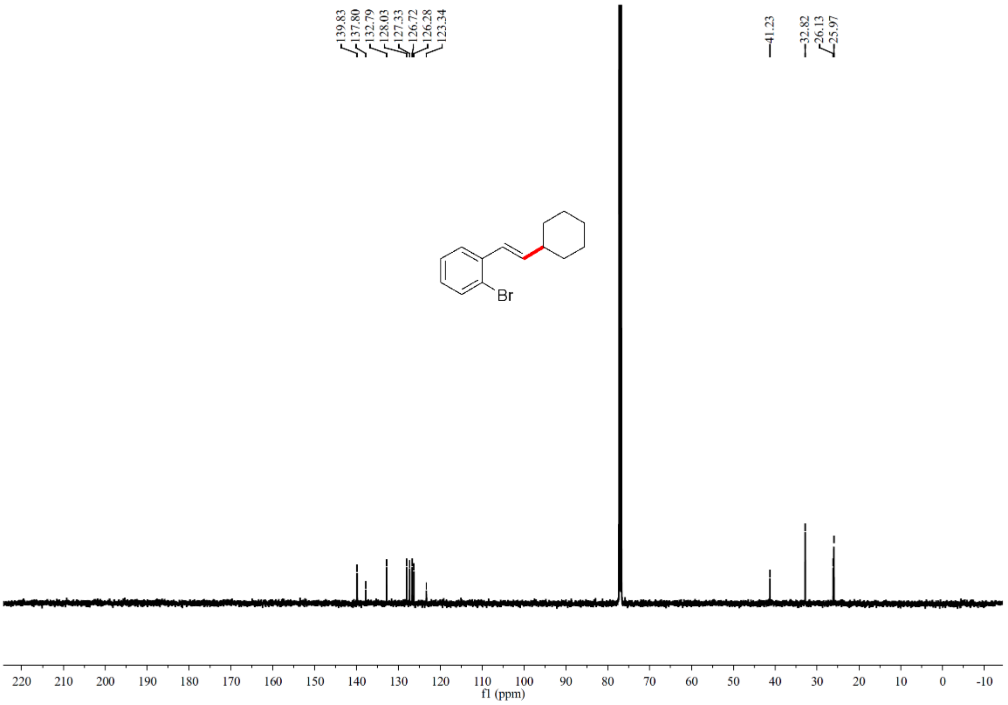
139.83
137.80
132.79
128.03
127.33
126.72
126.28
123.34
41.23
32.82
26.13
25.97
Br
f1 (ppm)

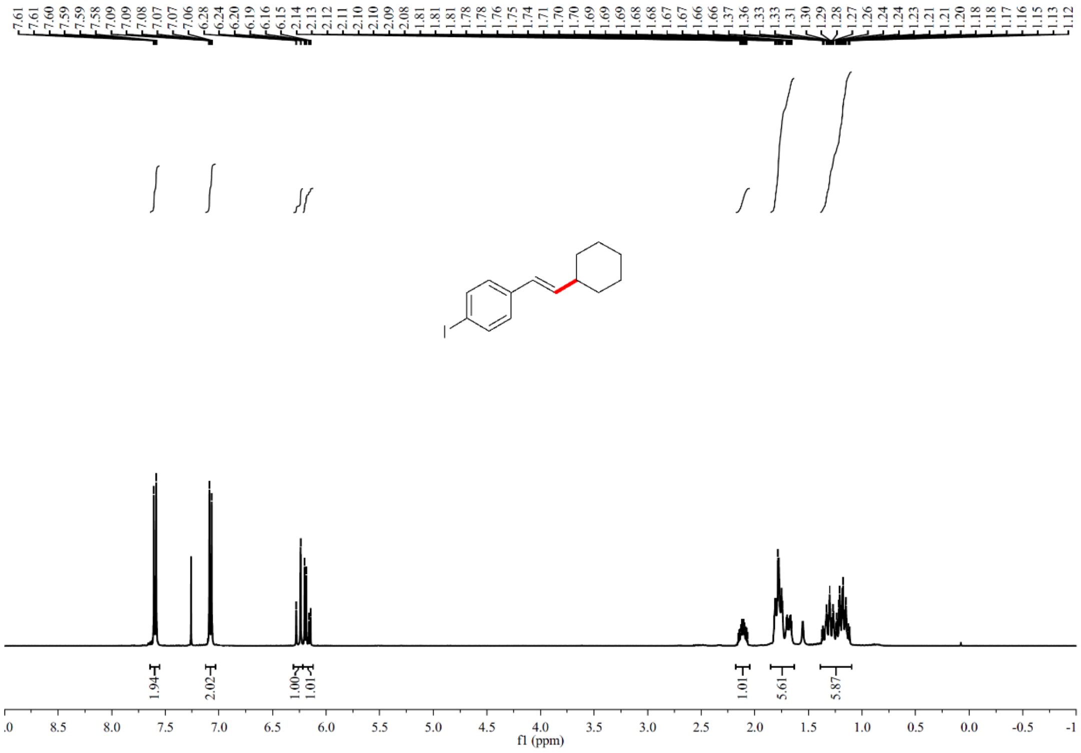
7.61 7.61 7.60 7.59 7.59 7.58 7.09 7.09 7.08 7.07 7.07 7.06 6.28 6.24 6.20 6.19 6.16 6.15 2.14 2.13 2.12 2.11 2.10 2.09 2.08 1.81 1.81 1.78 1.76 1.75 1.74 1.71 1.70 1.70 1.69 1.69 1.68 1.68 1.67 1.67 1.66 1.66 1.37 1.36 1.33 1.31 1.30 1.29 1.28 1.27 1.26 1.24 1.24 1.23 1.21 1.21 1.20 1.18 1.18 1.17 1.16 1.15 1.13 1.12
I
1.94
2.02
1.00
1.01
1.01
5.61
5.87
f1 (ppm)

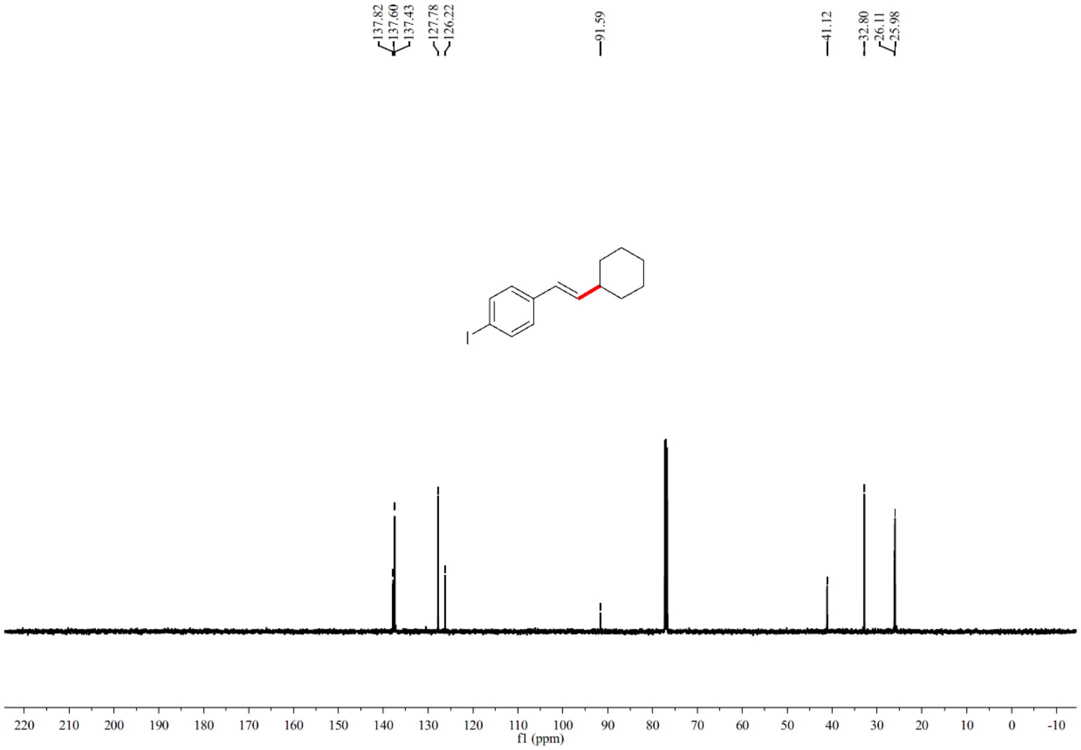
137.82
137.60
137.43
127.78
126.22
91.59
41.12
32.80
26.11
25.98
I
f1 (ppm)

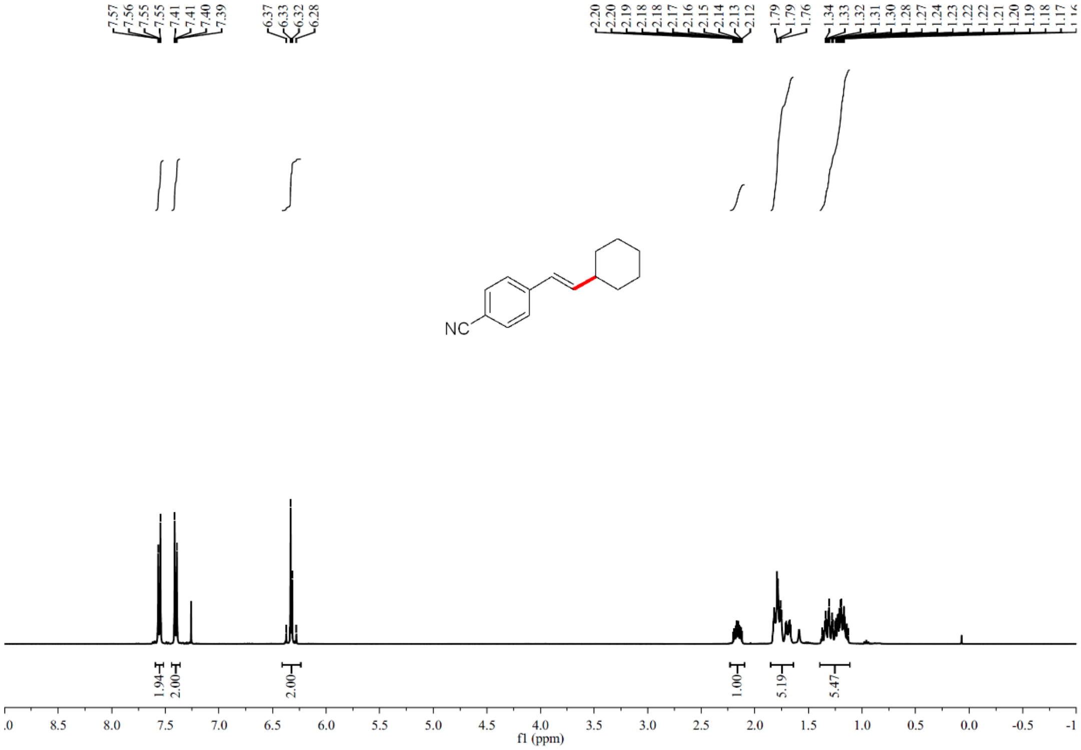
NC
f1 (ppm)

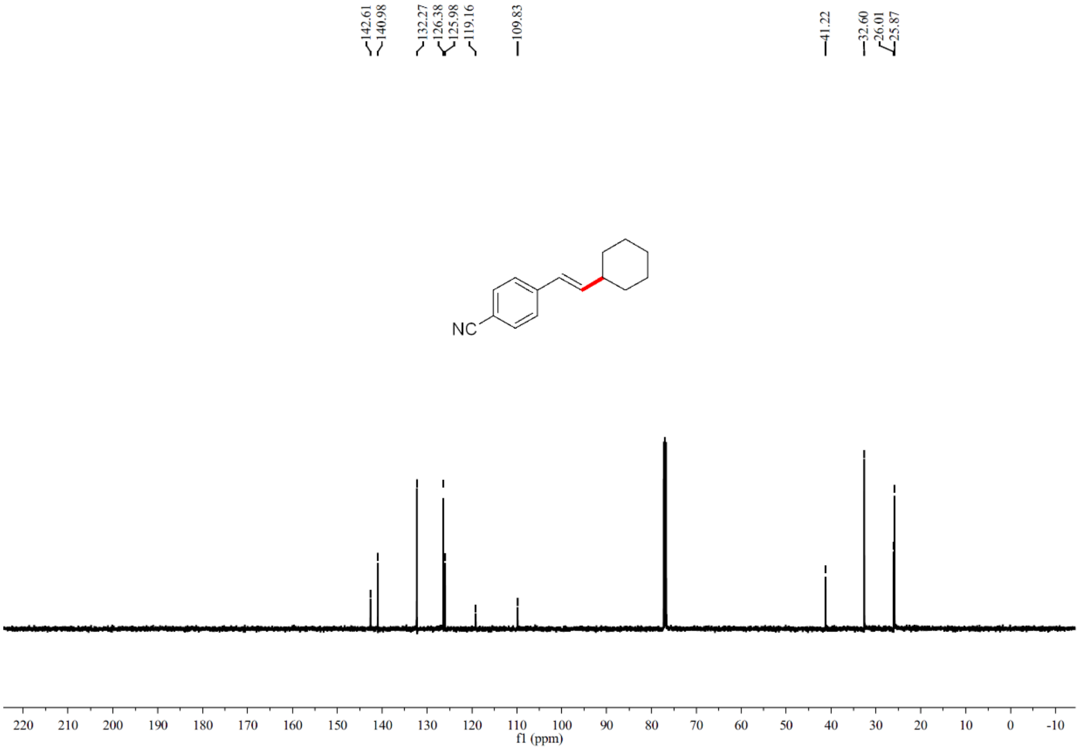

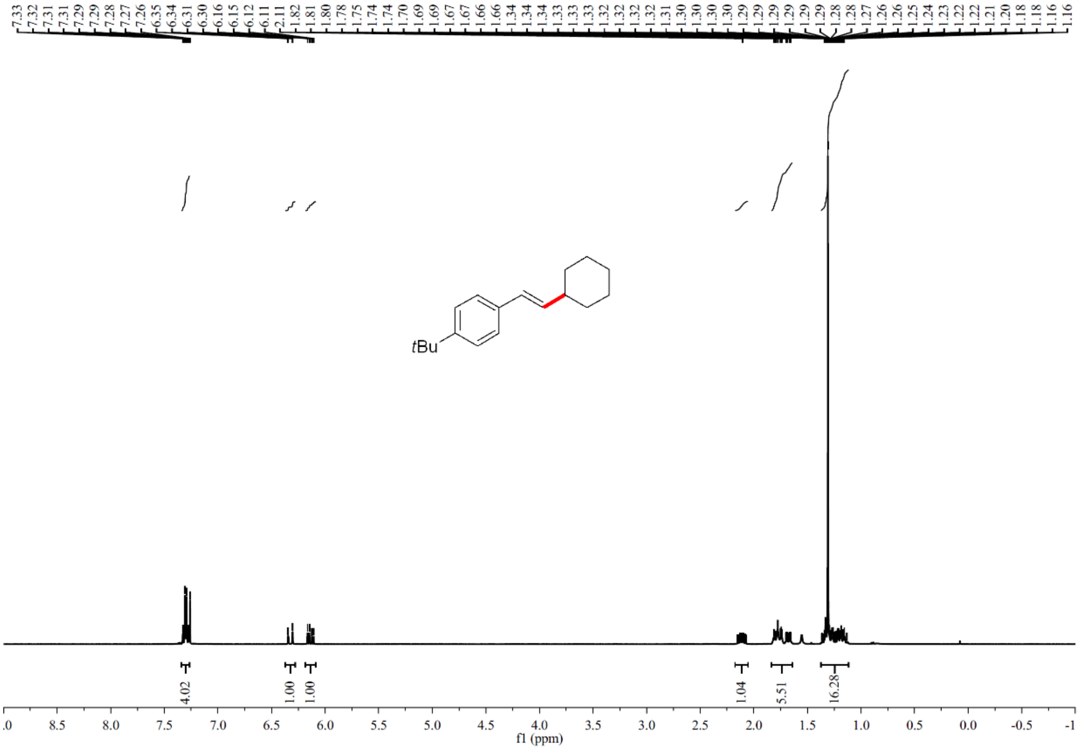

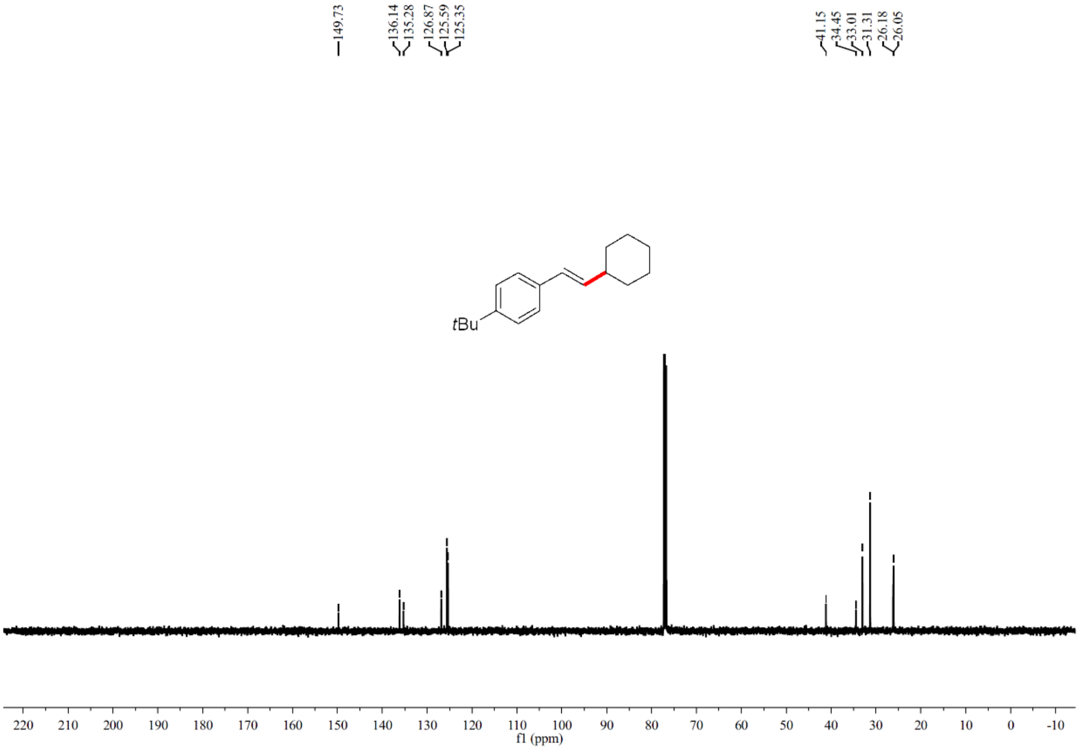
149.73
136.14
135.28
126.87
125.59
125.35
41.15
34.45
33.01
31.31
26.18
26.05
tBu
220 210 200 190 180 170 160 150 140 130 120 110 100 90 80 70 60 50 40 30 20 10 0 -10
f1 (ppm)

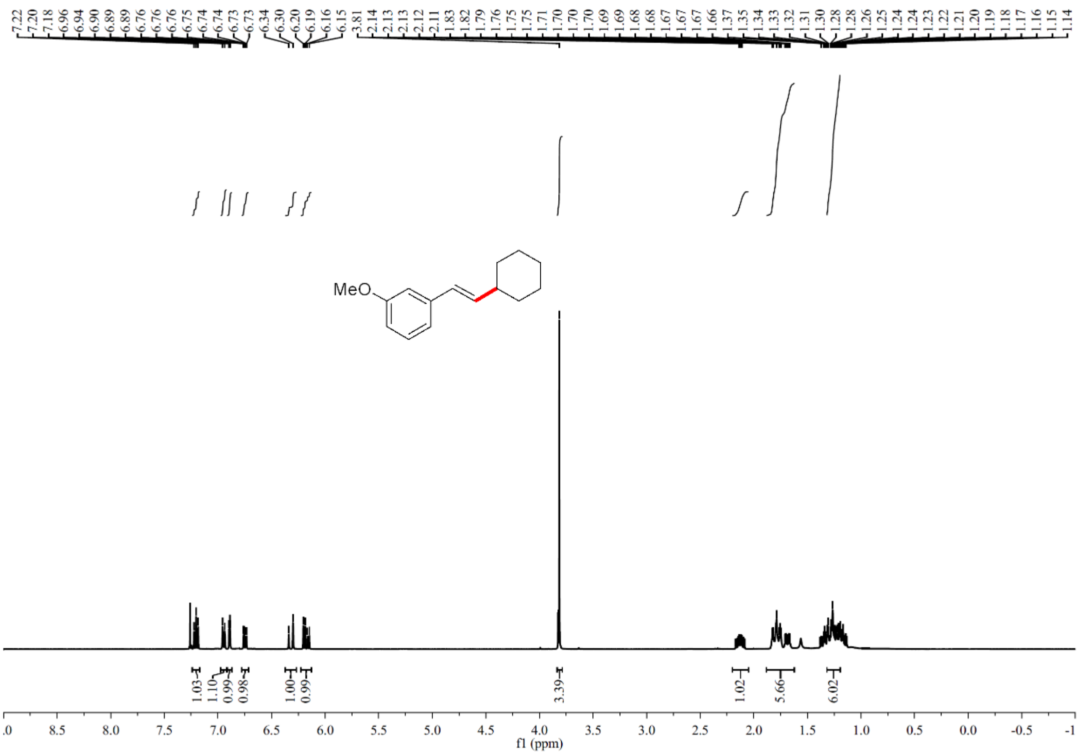
7.22 7.20 7.18 6.96 6.94 6.90 6.89 6.76 6.76 6.76 6.75 6.74 6.74 6.73 6.73 6.34 6.30 6.20 6.19 6.16 6.15 3.81 2.14 2.13 2.13 2.12 2.11 1.83 1.82 1.79 1.76 1.75 1.75 1.71 1.70 1.70 1.70 1.69 1.69 1.68 1.68 1.67 1.67 1.67 1.66 1.37 1.35 1.34 1.33 1.32 1.31 1.30 1.28 1.28 1.26 1.25 1.24 1.24 1.23 1.22 1.21 1.20 1.19 1.18 1.17 1.16 1.15 1.14
MeO
1.03 1.10 0.99 0.98 1.00 0.99 3.39 1.02 5.66 6.02
.0 8.5 8.0 7.5 7.0 6.5 6.0 5.5 5.0 4.5 4.0 3.5 3.0 2.5 2.0 1.5 1.0 0.5 0.0 -0.5 -1
f1 (ppm)

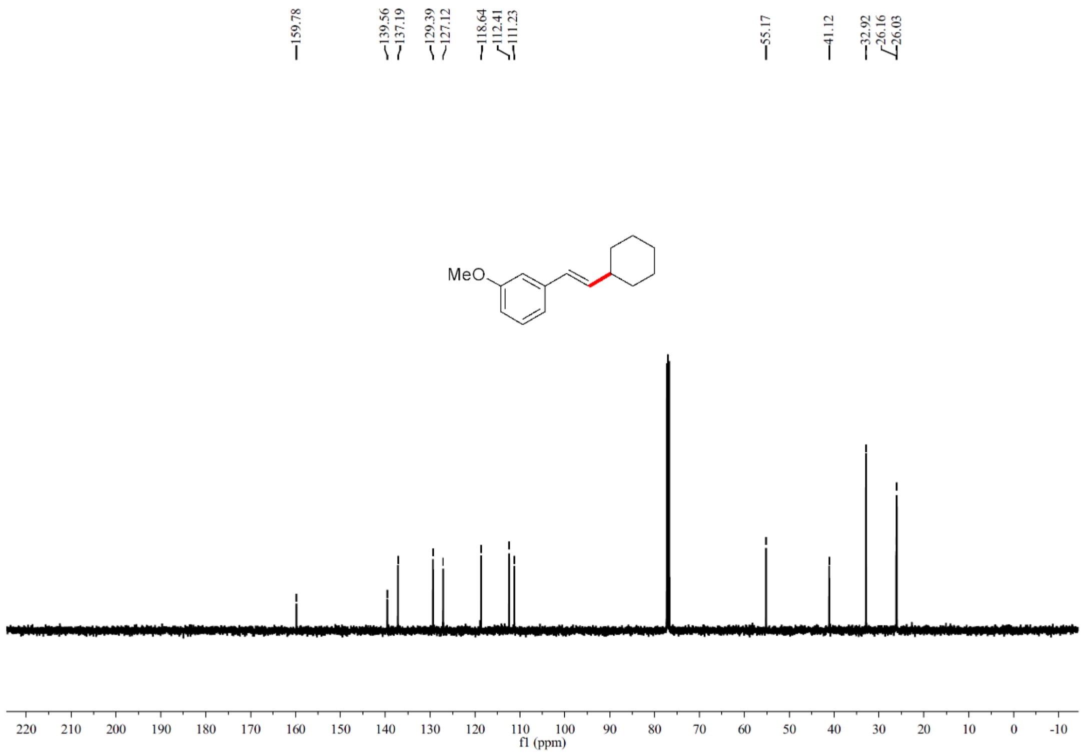

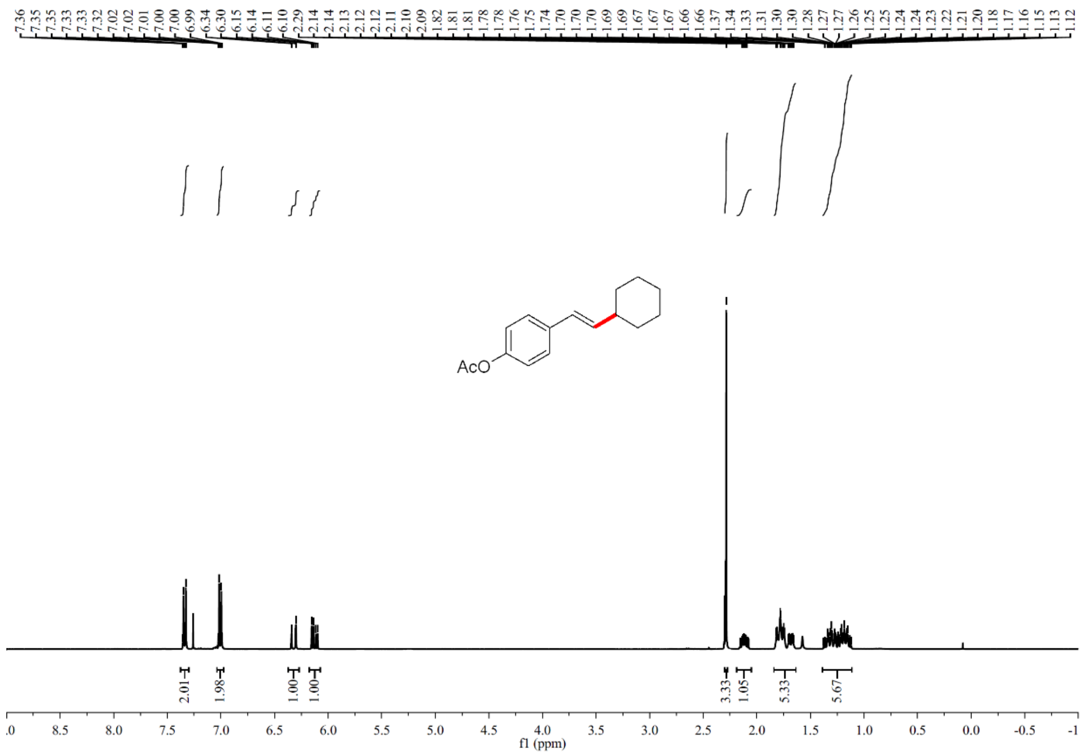

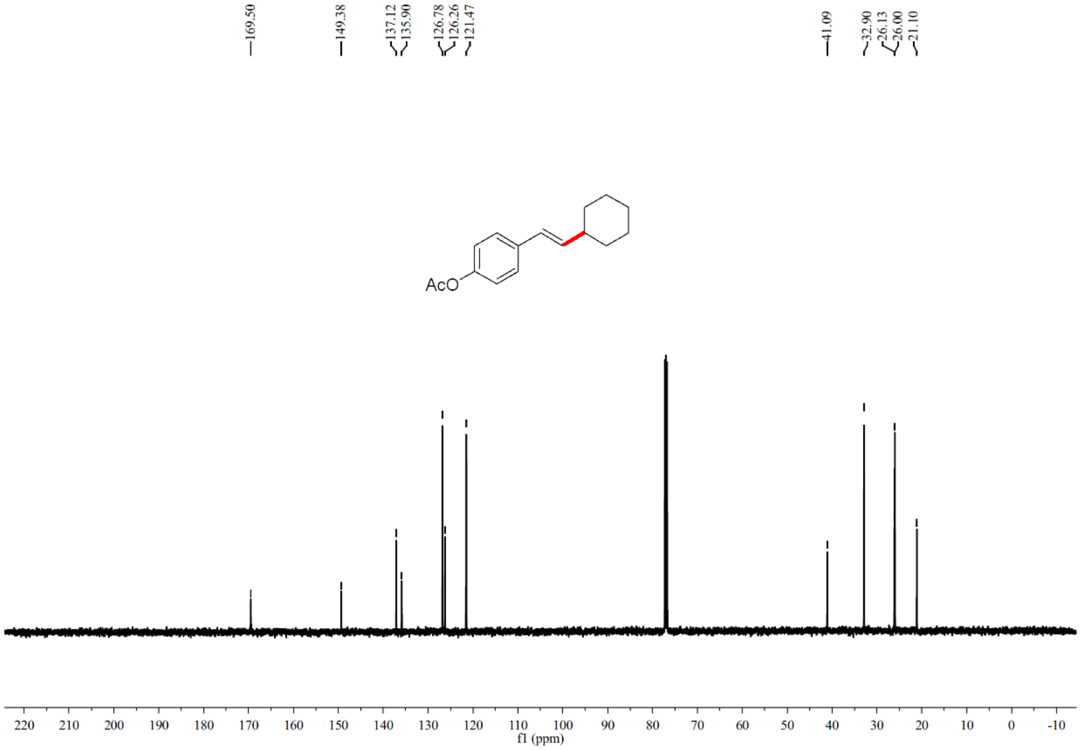

169.50
149.38
137.12
135.90
126.78
126.26
121.47
41.09
32.90
26.13
26.00
21.10
AcO
f1 (ppm)

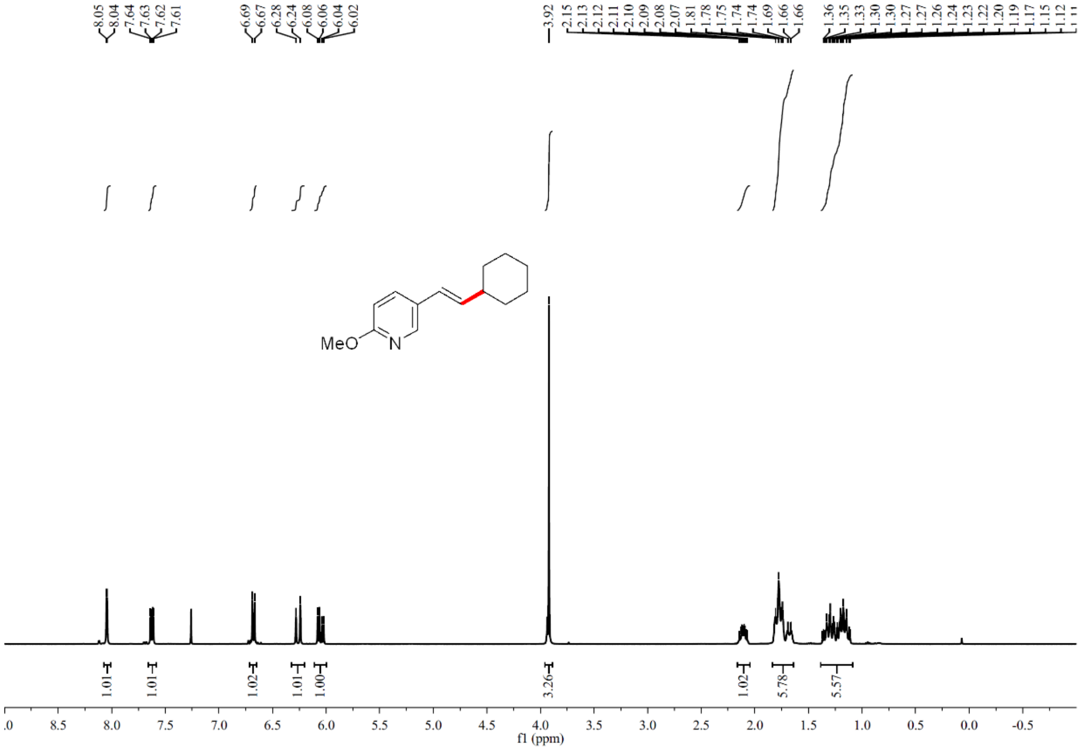

MeO
N
3.92
1.01
1.01
1.02
1.01
1.00
3.26
1.02
5.78
5.57
f1 (ppm)

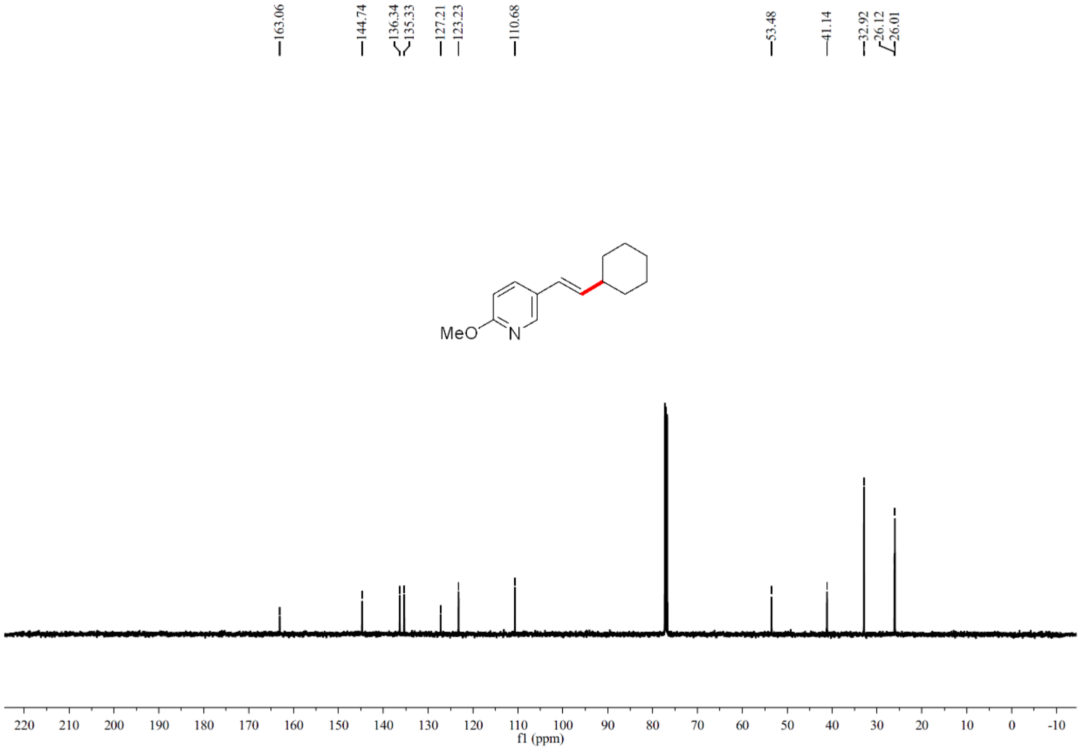
163.06
144.74
136.34
135.33
127.21
123.23
110.68
53.48
41.14
32.92
26.12
26.01
MeO
N
f1 (ppm)

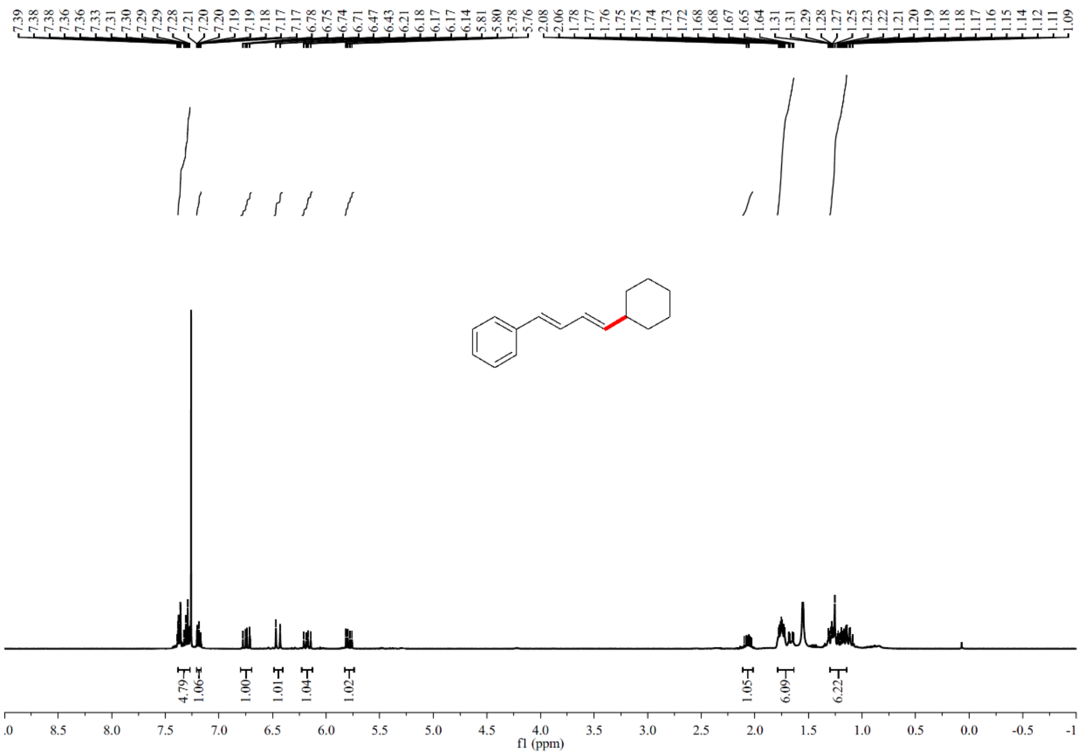
7.39
7.38
7.38
7.36
7.36
7.33
7.31
7.30
7.29
7.29
7.28
7.21
7.20
7.20
7.19
7.19
7.18
7.17
7.17
6.78
6.75
6.74
6.71
6.47
6.43
6.21
6.18
6.17
6.17
6.14
5.81
5.80
5.78
5.76
2.08
2.06
1.78
1.77
1.76
1.75
1.75
1.74
1.73
1.72
1.68
1.67
1.65
1.64
1.31
1.31
1.29
1.28
1.27
1.25
1.23
1.22
1.21
1.20
1.19
1.18
1.18
1.17
1.16
1.15
1.14
1.12
1.11
1.09
4.79
1.06
1.00
1.01
1.04
1.02
1.05
6.09
6.22
f1 (ppm)

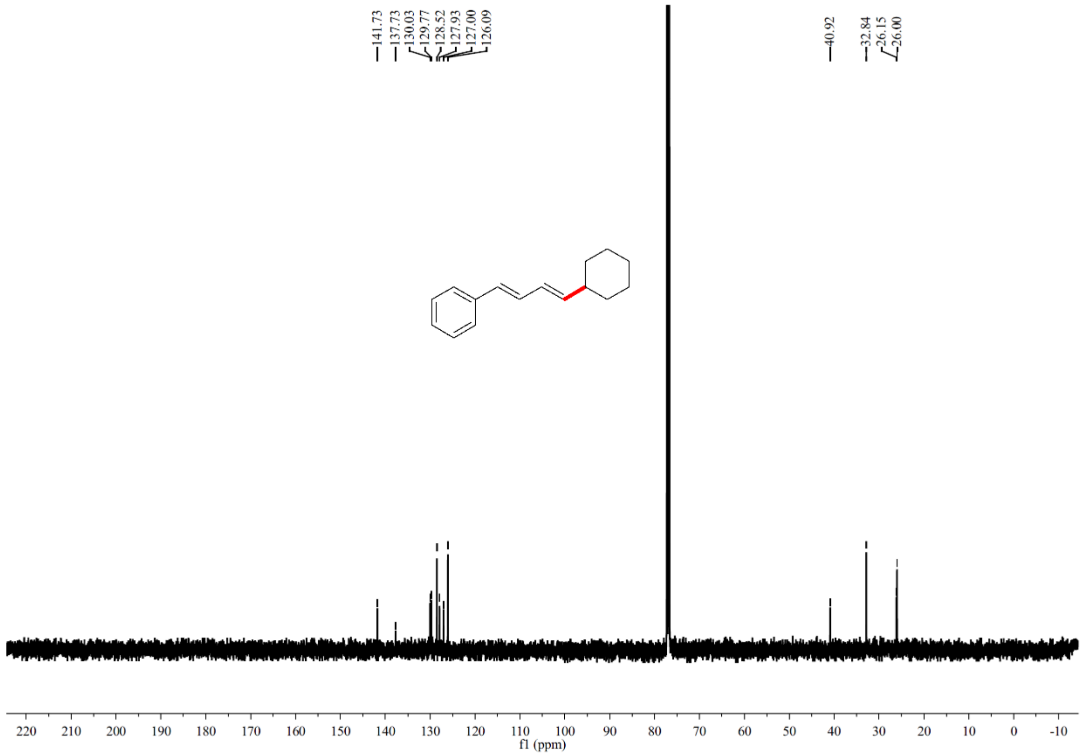

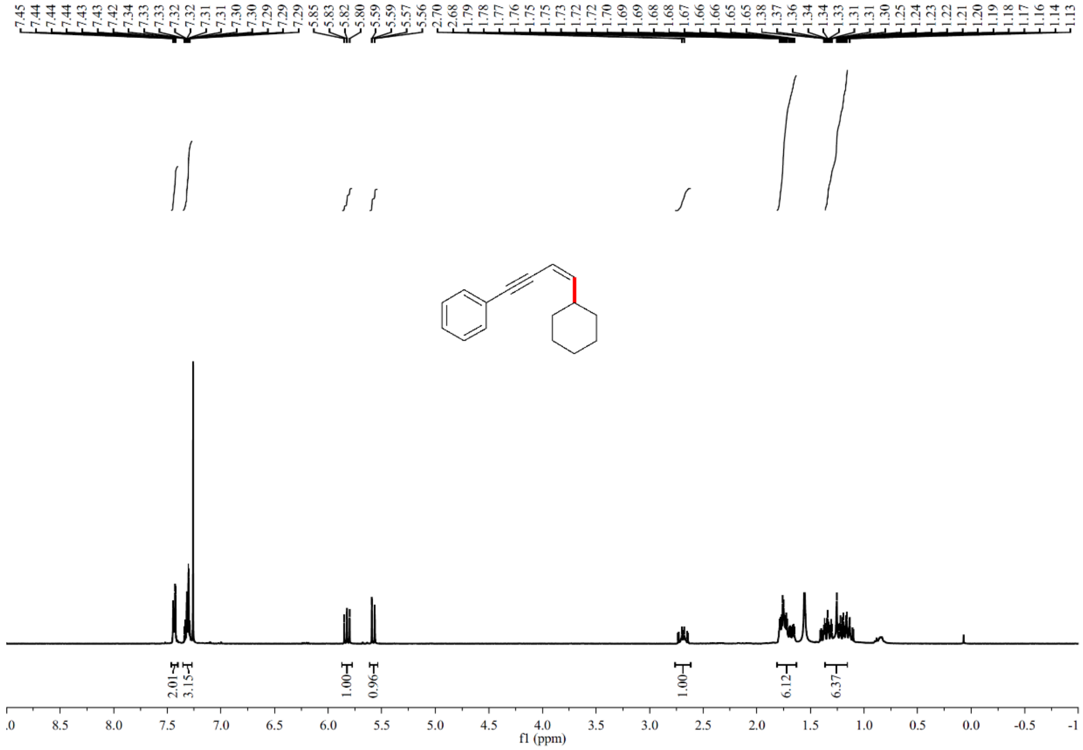

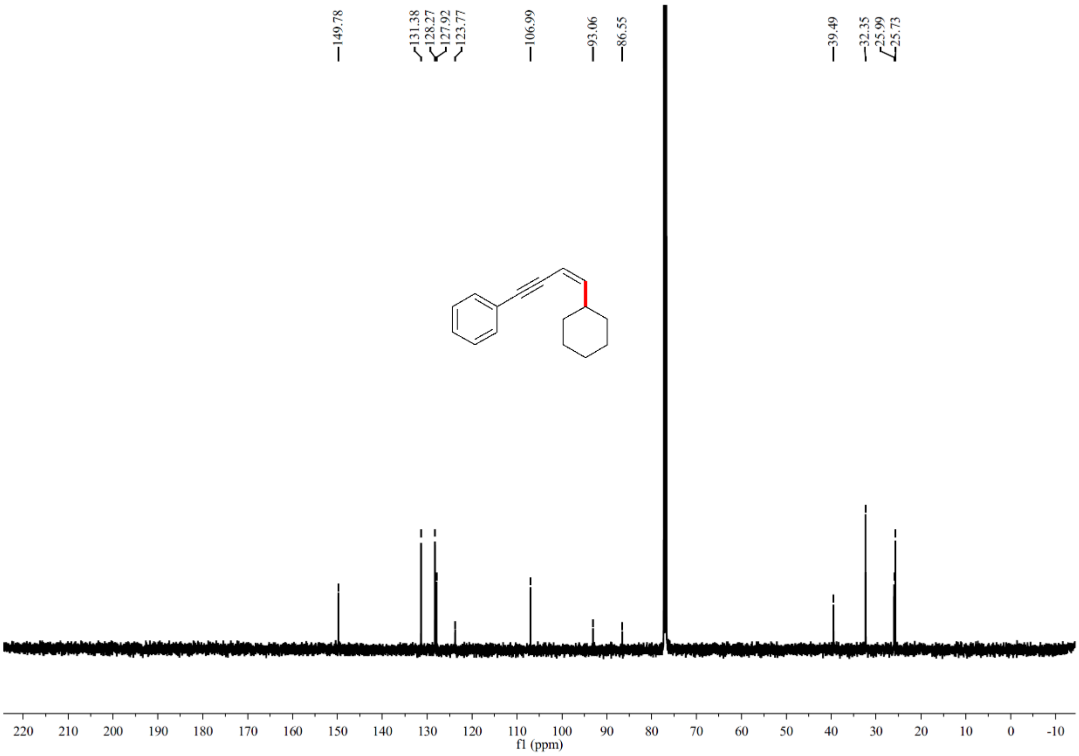

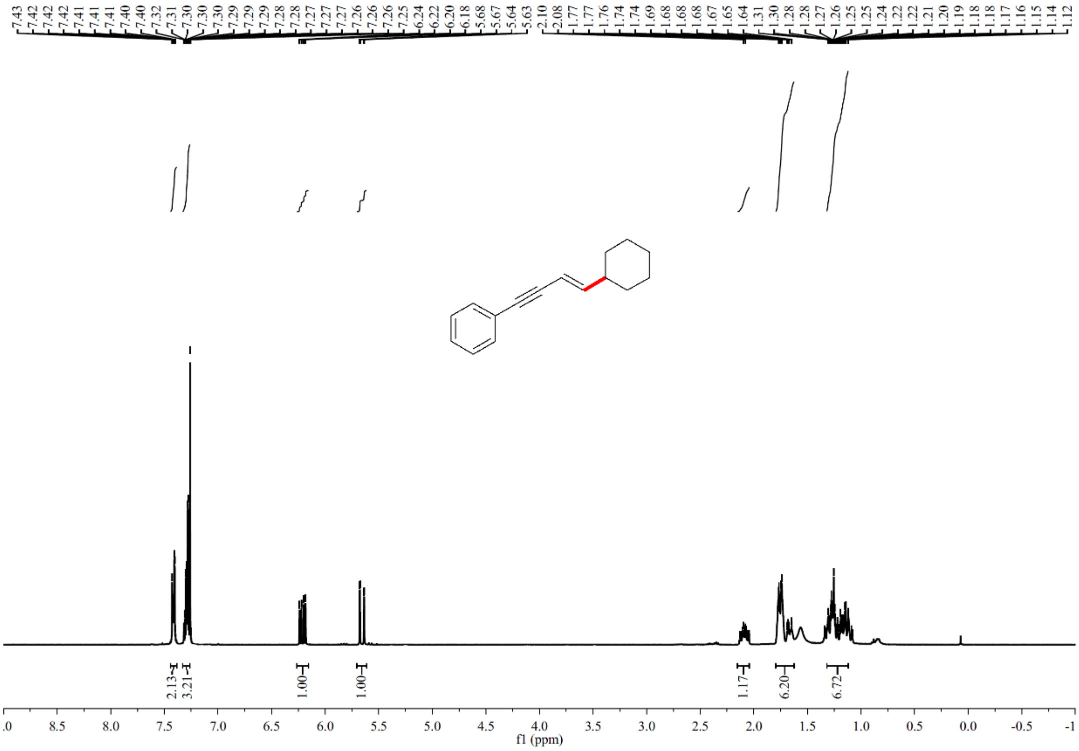

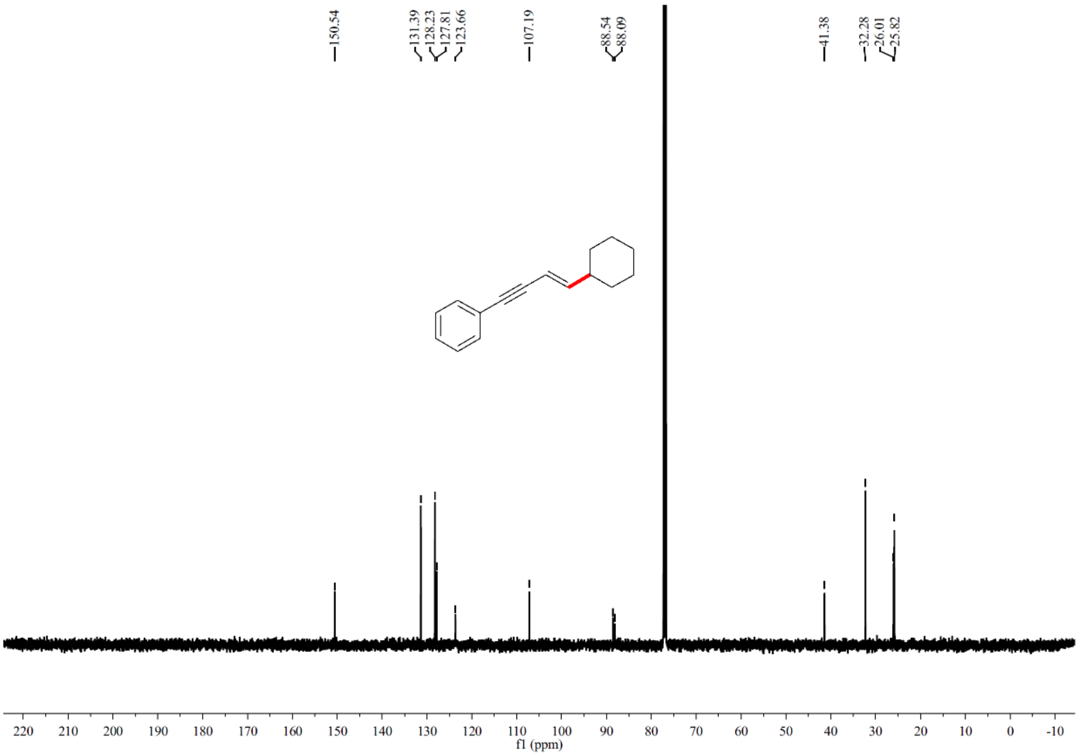
150.54
131.39
128.23
127.81
123.66
107.19
88.54
88.09
41.38
32.28
26.01
25.82
f1 (ppm)

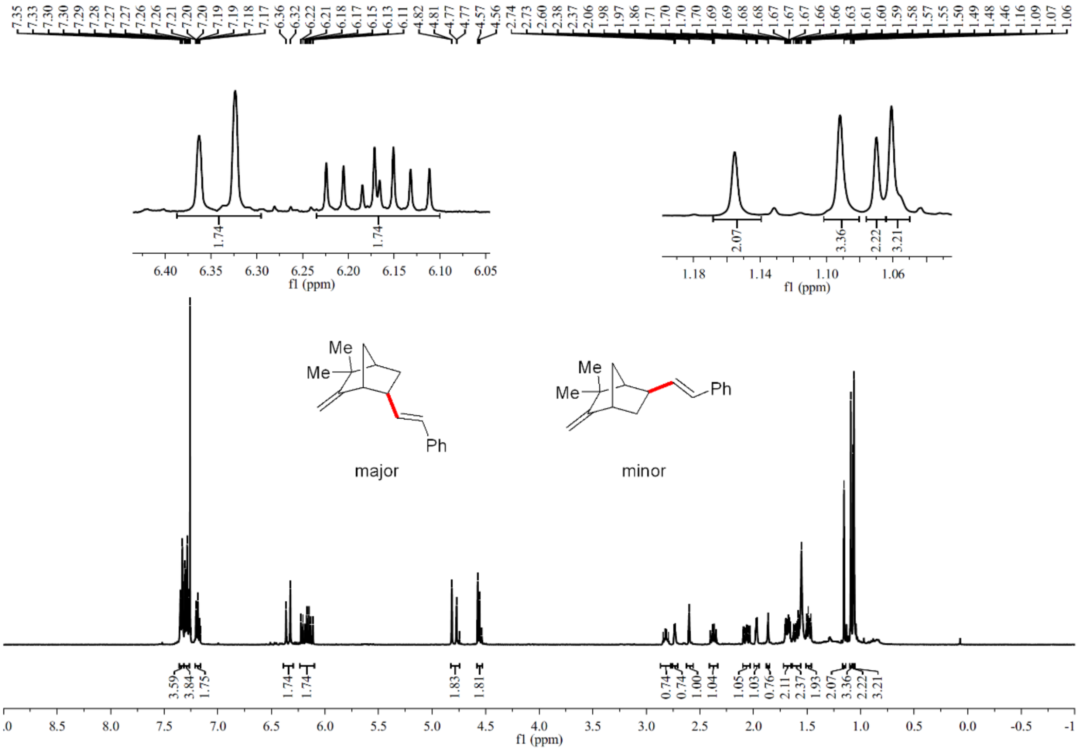
Me
Me
Ph
major
Me
Me
Ph
minor
f1 (ppm)

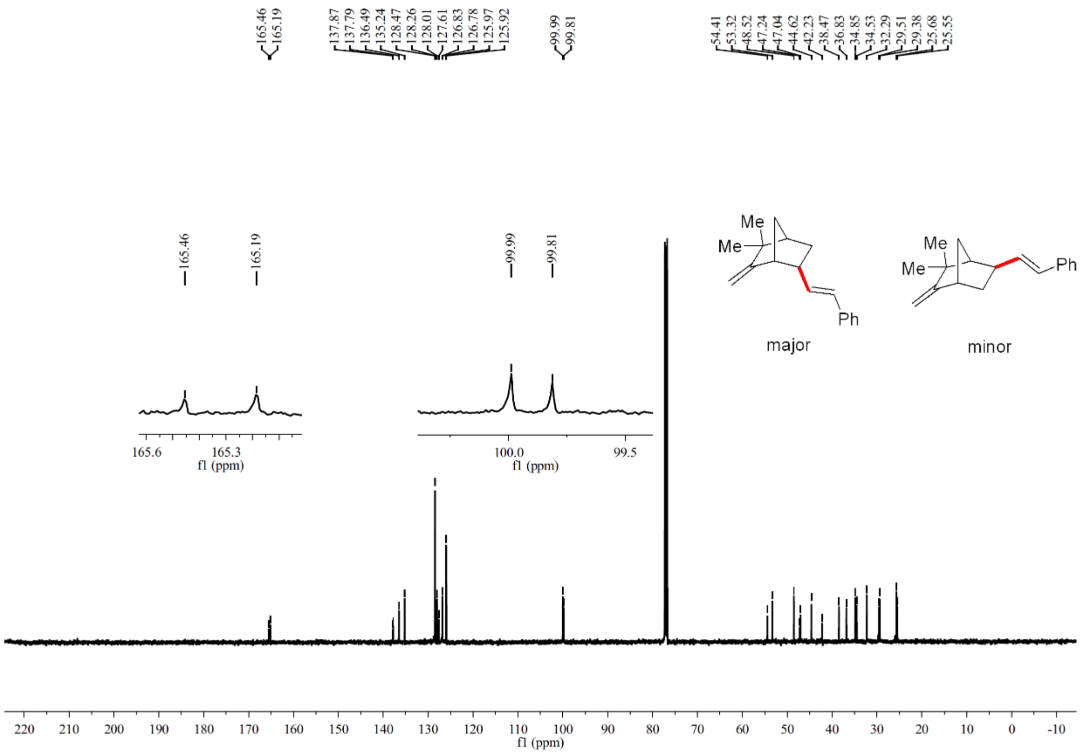

key HMBC correlations to indentify H_a $H_{a'}$ H_b $H_{b'}$

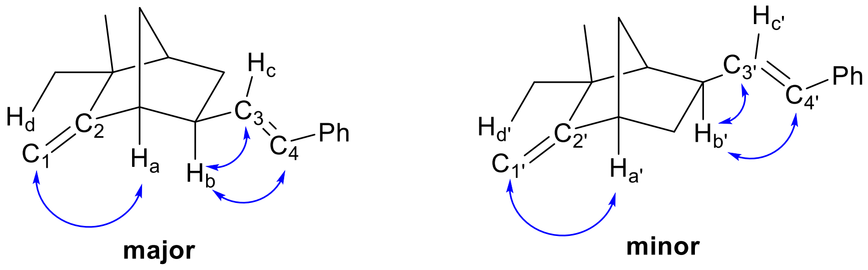

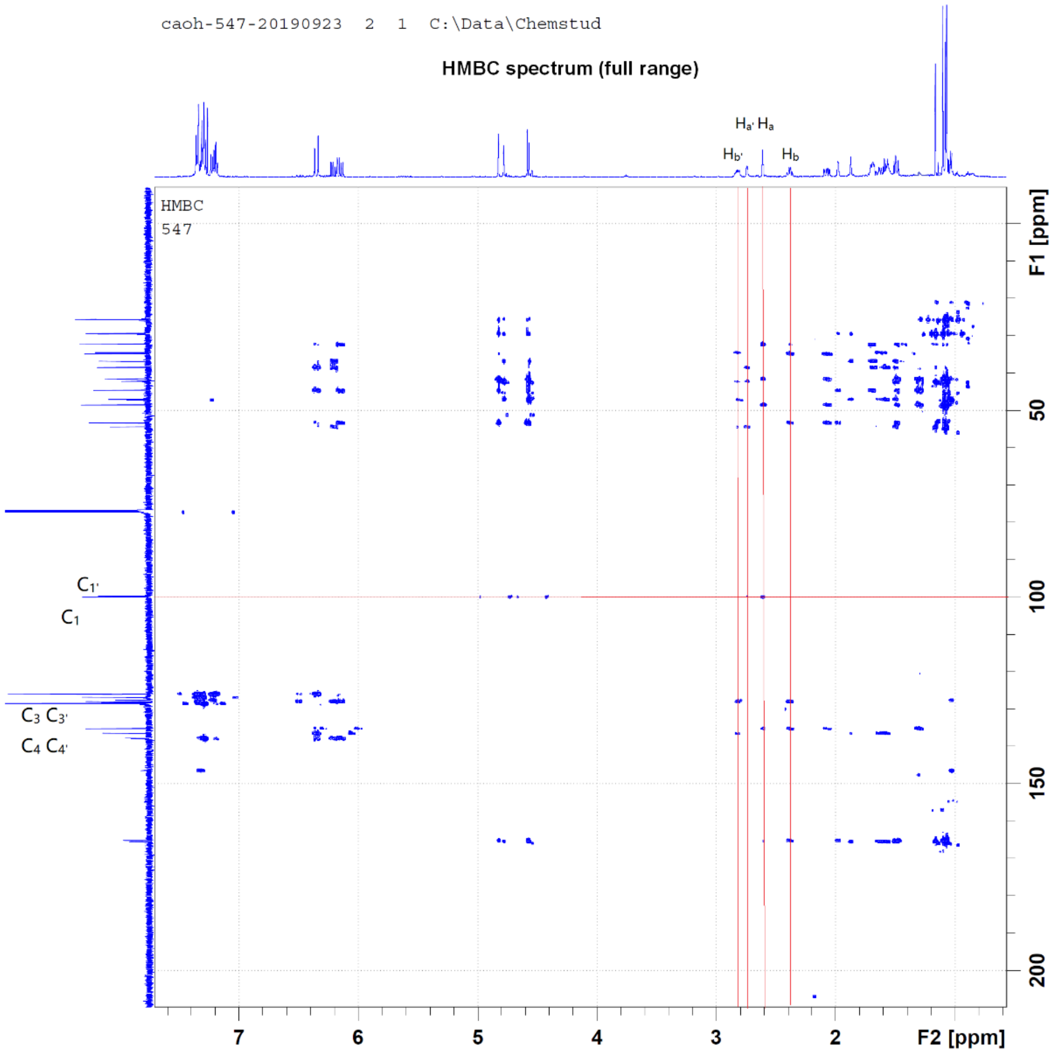

key HMBC correlations of C_2 and $C_{2'}$

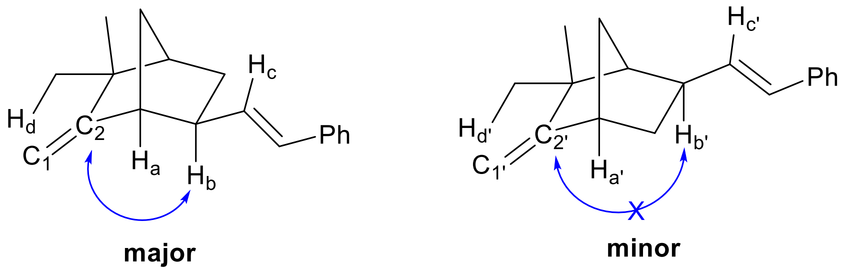

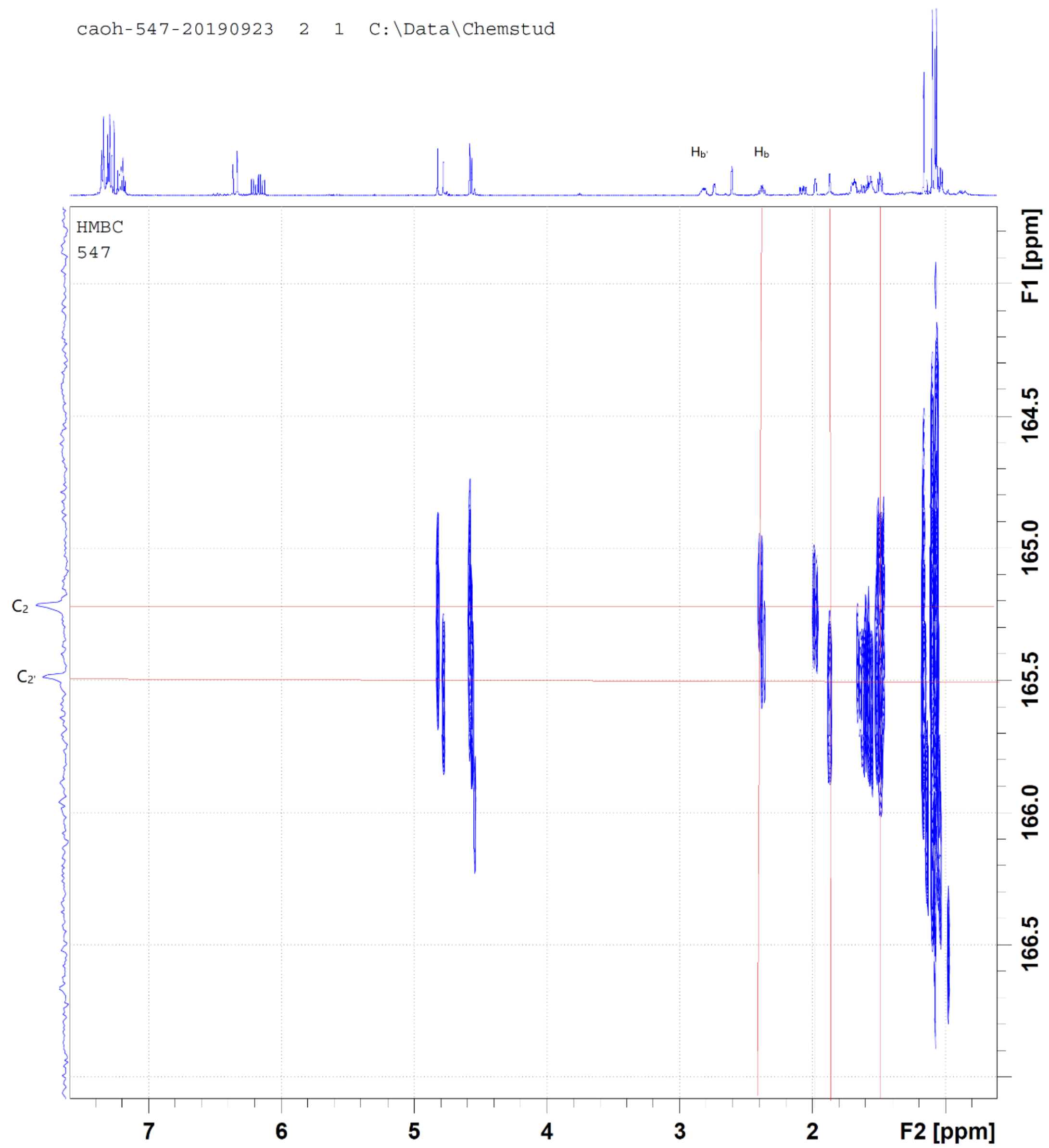

key COSY correlations

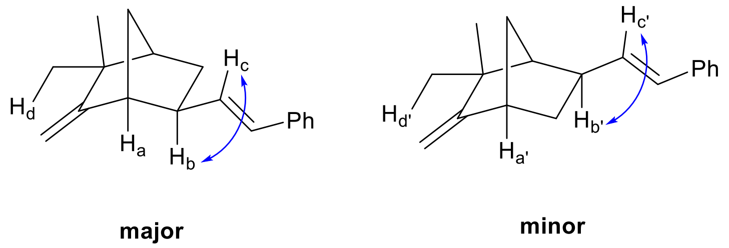

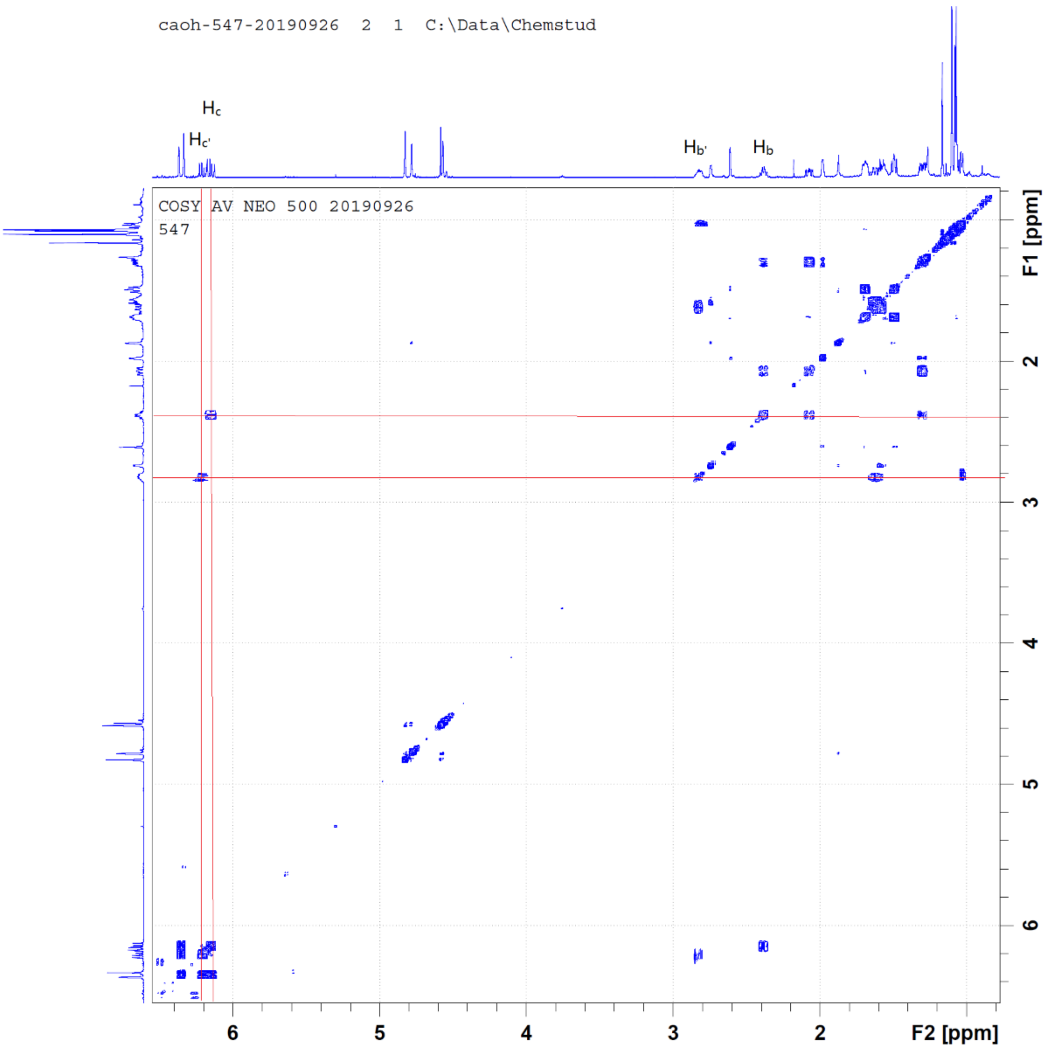

key NOESY correlations

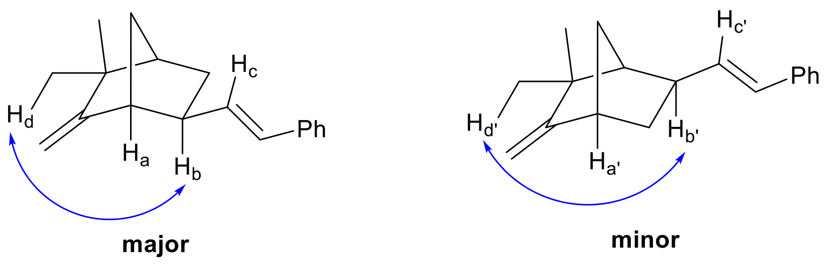

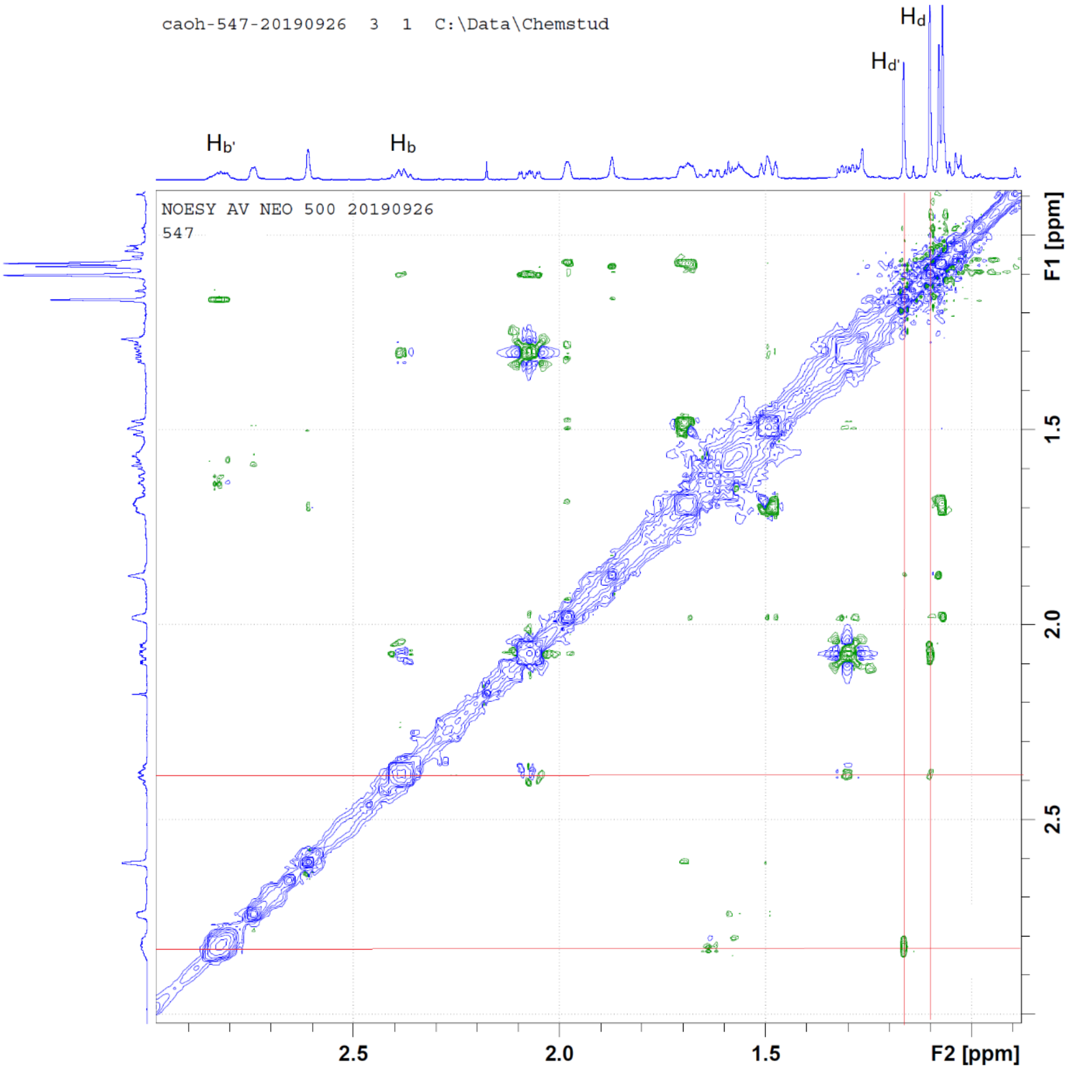

caoh-547-20190926 3 1 C:\Data\Chemstud
Hd
Hd'
Hb'
Hb
NOESY AV NEO 500 20190926
547
F1 [ppm]
1.5
2.0
2.5
2.5
2.0
1.5
F2 [ppm]

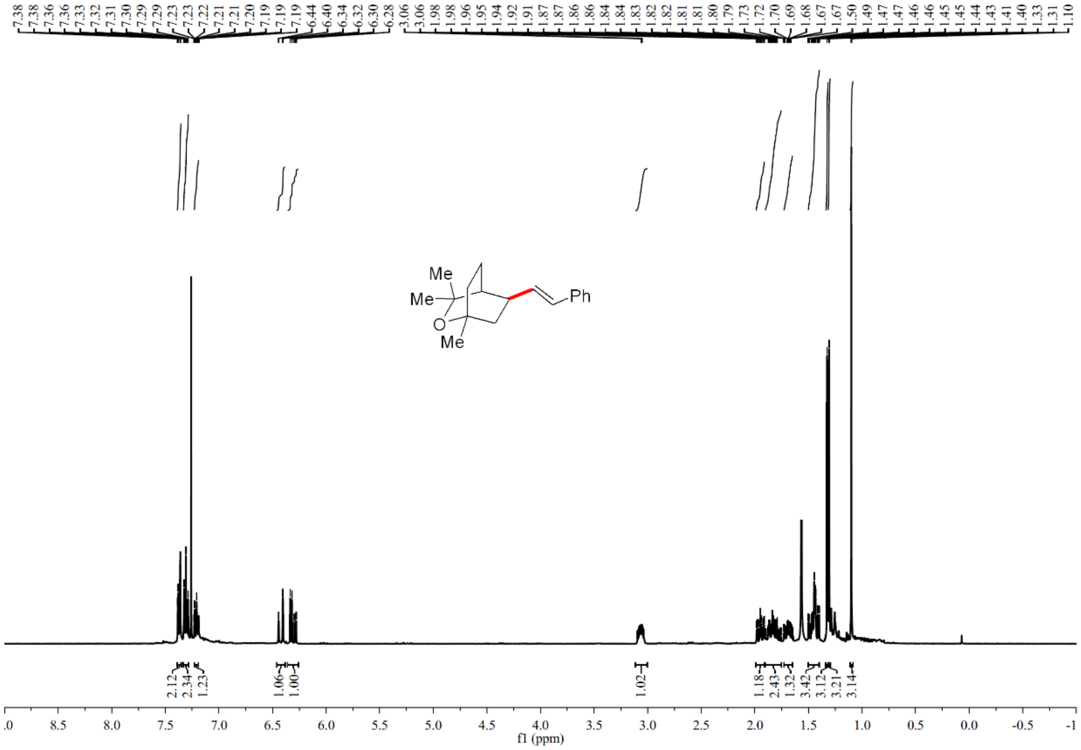
Me
Me
O
Me
Ph
f1 (ppm)

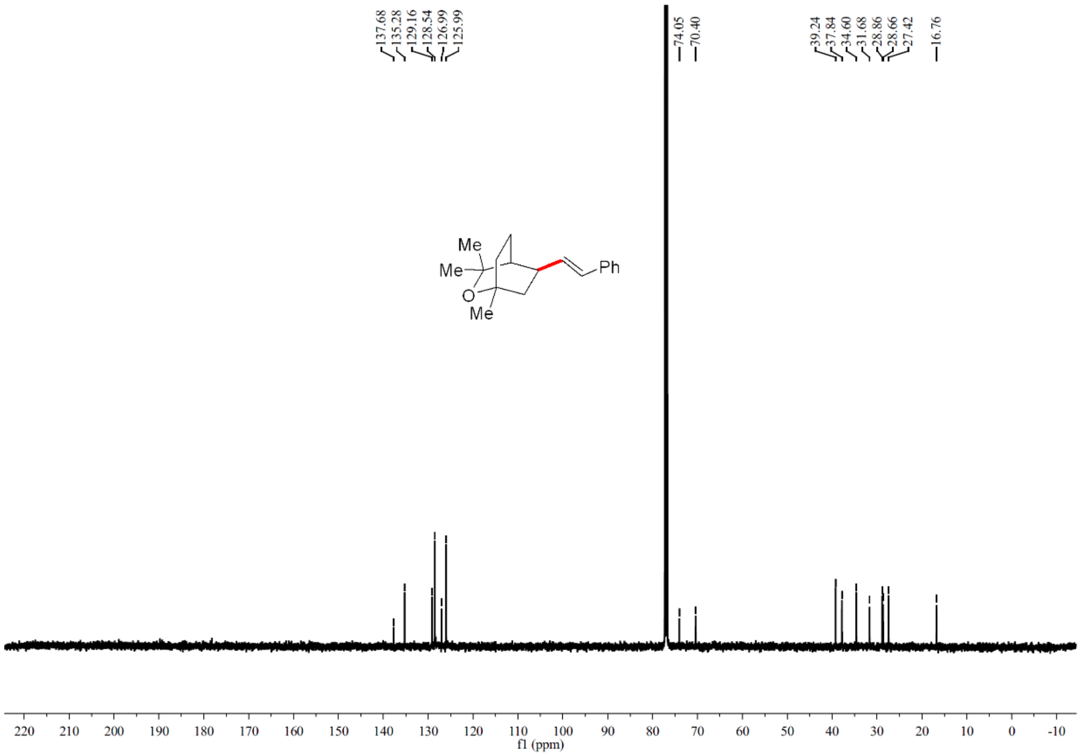
137.68
135.28
129.16
128.54
126.99
125.99
74.05
70.40
39.24
37.84
34.60
31.68
28.86
28.66
27.42
16.76
Me
Me
O
Me
Ph
f1 (ppm)

key COSY correlations

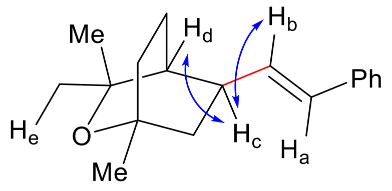

H$_d$ 1.95 ppm (ddd, J = 14.0, 10.8, 3.6 Hz)

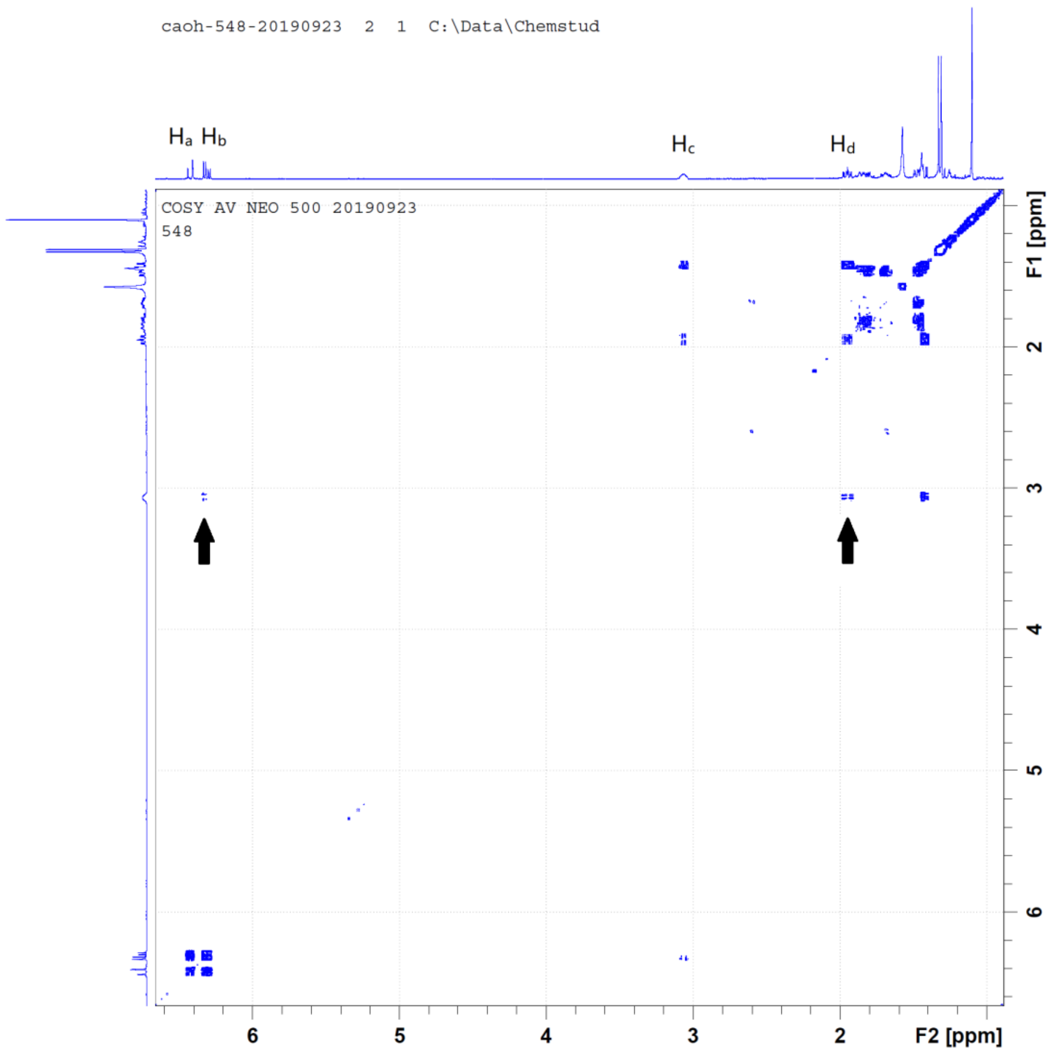

key NOESY correlations

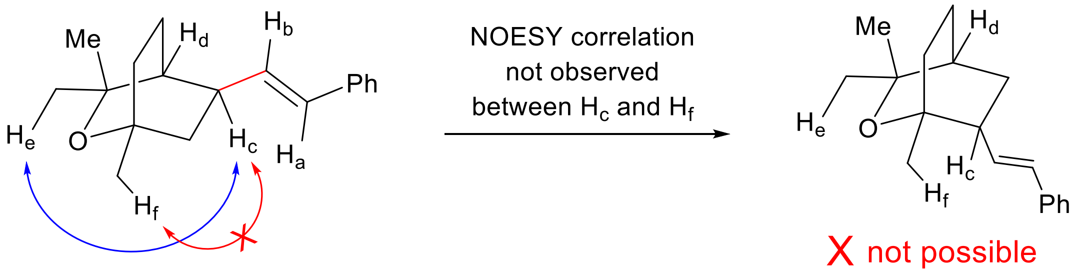

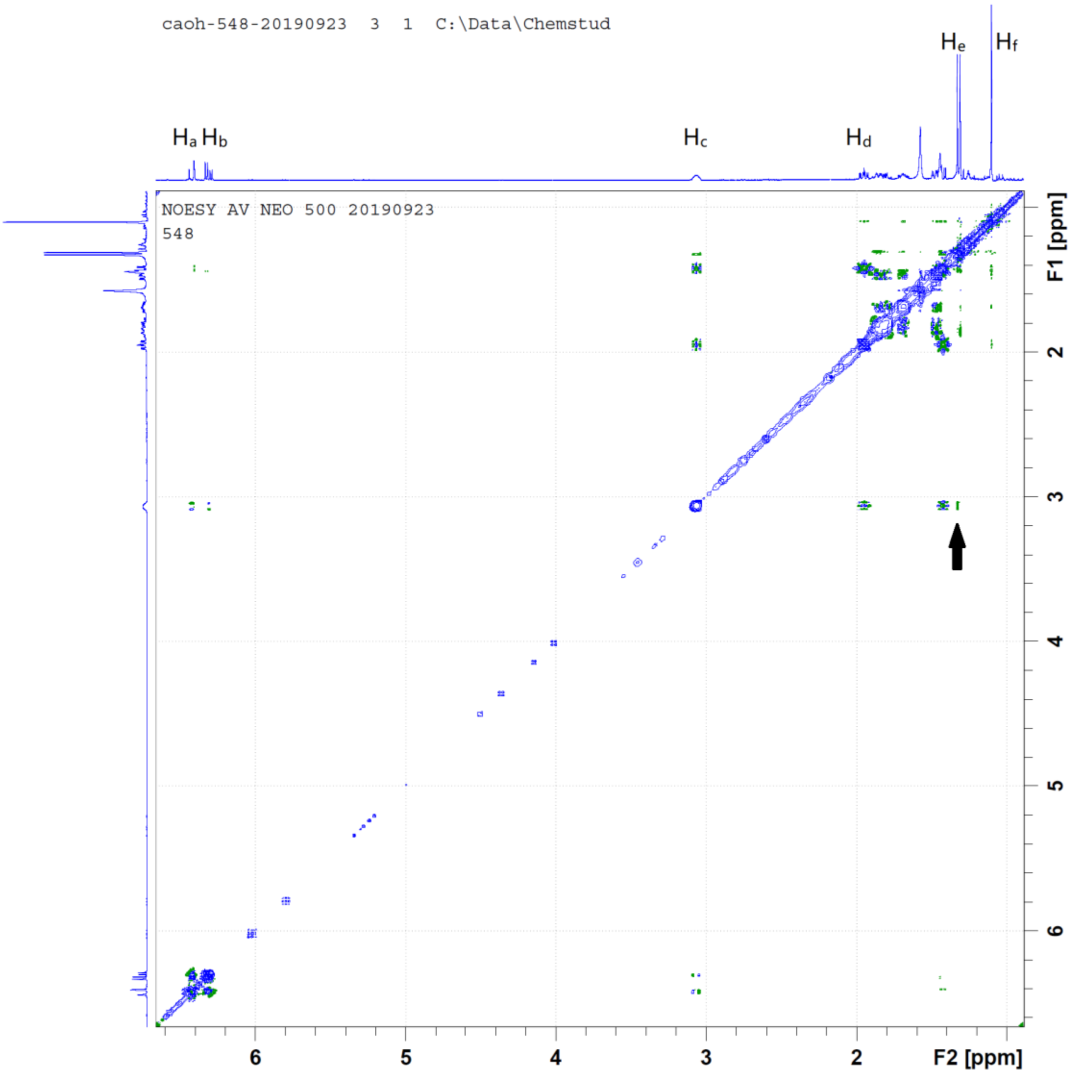

NOESY correlation between H_c and H_e

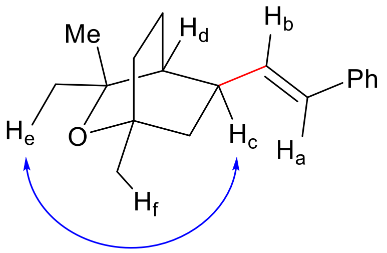

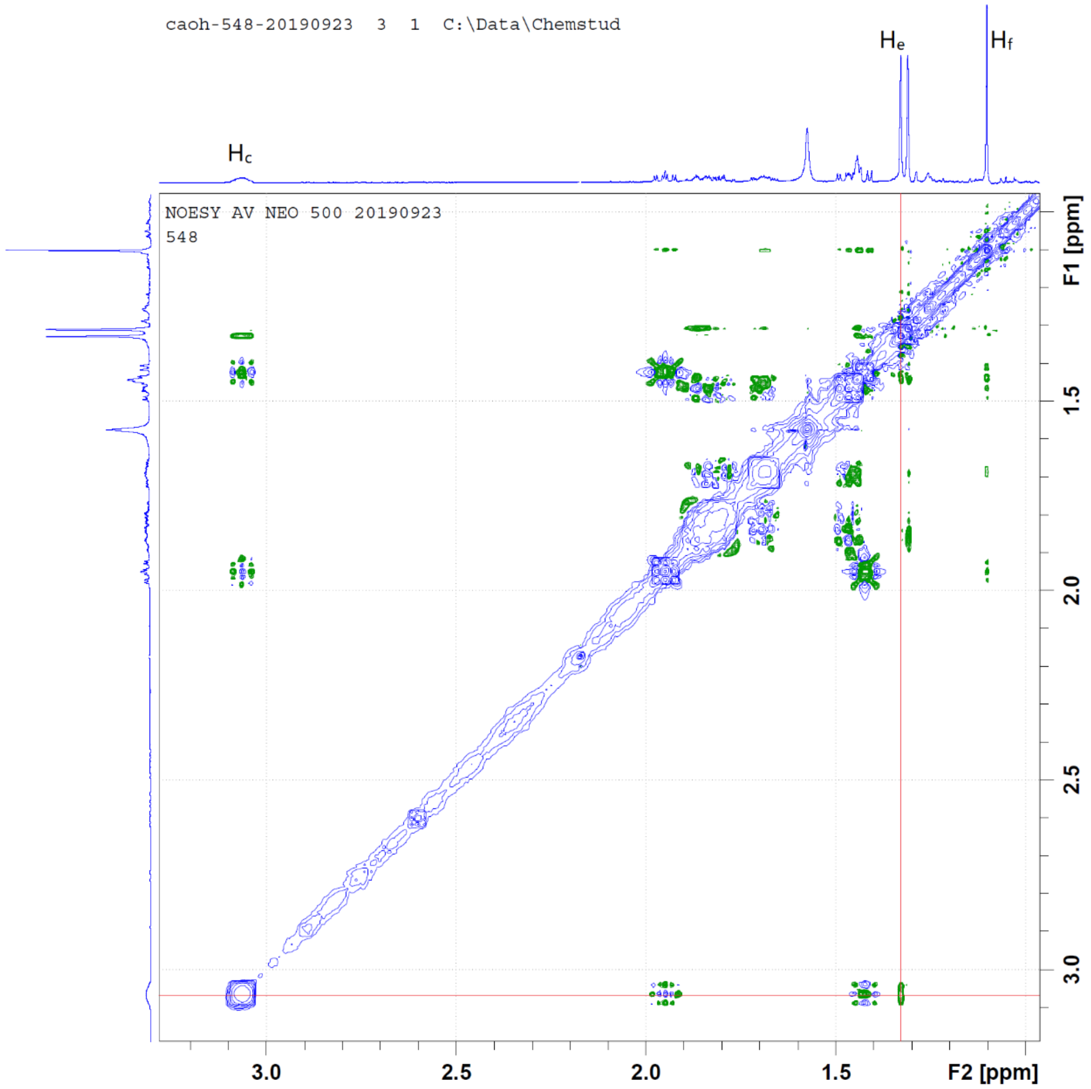

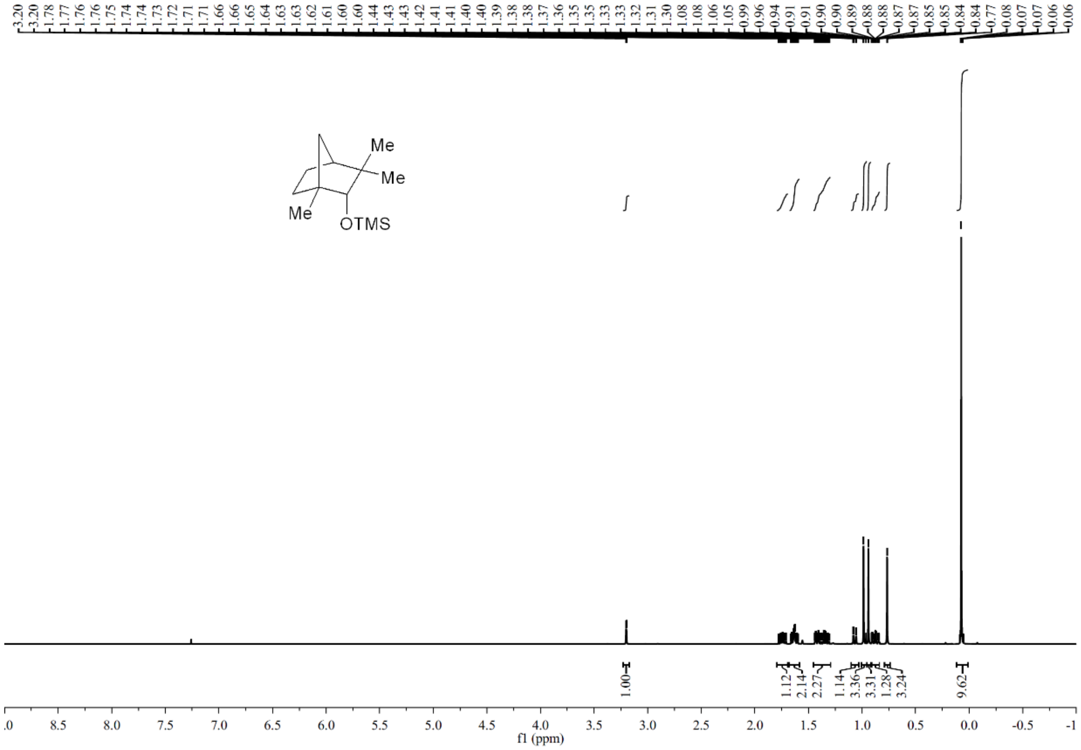
3.20
3.20
1.78
1.77
1.76
1.76
1.75
1.74
1.74
1.73
1.72
1.71
1.71
1.66
1.66
1.65
1.64
1.63
1.63
1.62
1.61
1.60
1.60
1.44
1.43
1.43
1.42
1.41
1.41
1.40
1.40
1.39
1.38
1.38
1.37
1.36
1.35
1.35
1.33
1.33
1.32
1.31
1.30
1.08
1.08
1.06
1.05
0.99
0.96
0.94
0.91
0.91
0.90
0.90
0.89
0.88
0.88
0.87
0.85
0.85
0.84
0.84
0.77
0.08
0.07
0.06
0.06
Me
Me
Me
OTMS
1.00
1.12
2.14
2.27
1.14
3.36
3.31
1.28
3.24
9.62
f1 (ppm)

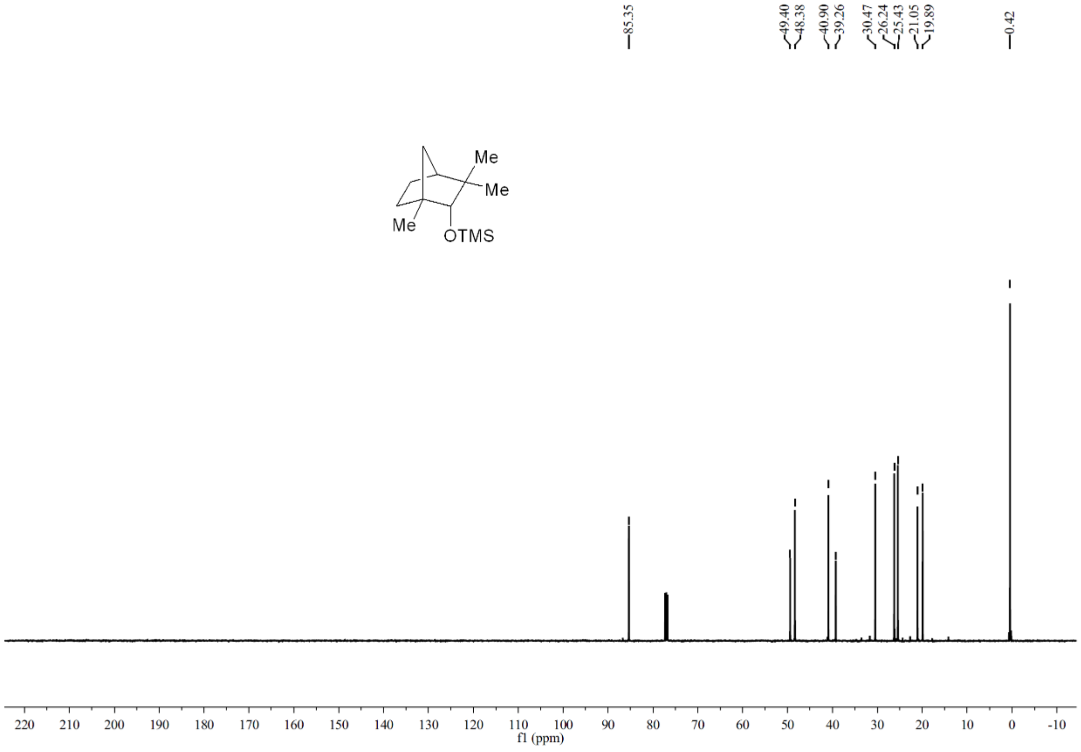
85.35
49.40
48.38
40.90
39.26
30.47
26.24
25.43
21.05
19.89
0.42
Me
Me
Me
OTMS
f1 (ppm)

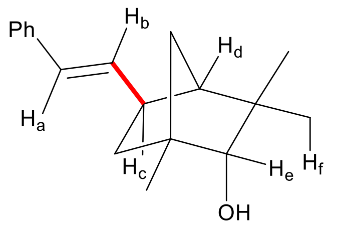

H_d 2.02 ppm (ddd, J = 13.2, 8.8, 1.6 Hz)

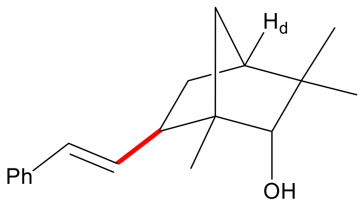

X not the main isomer

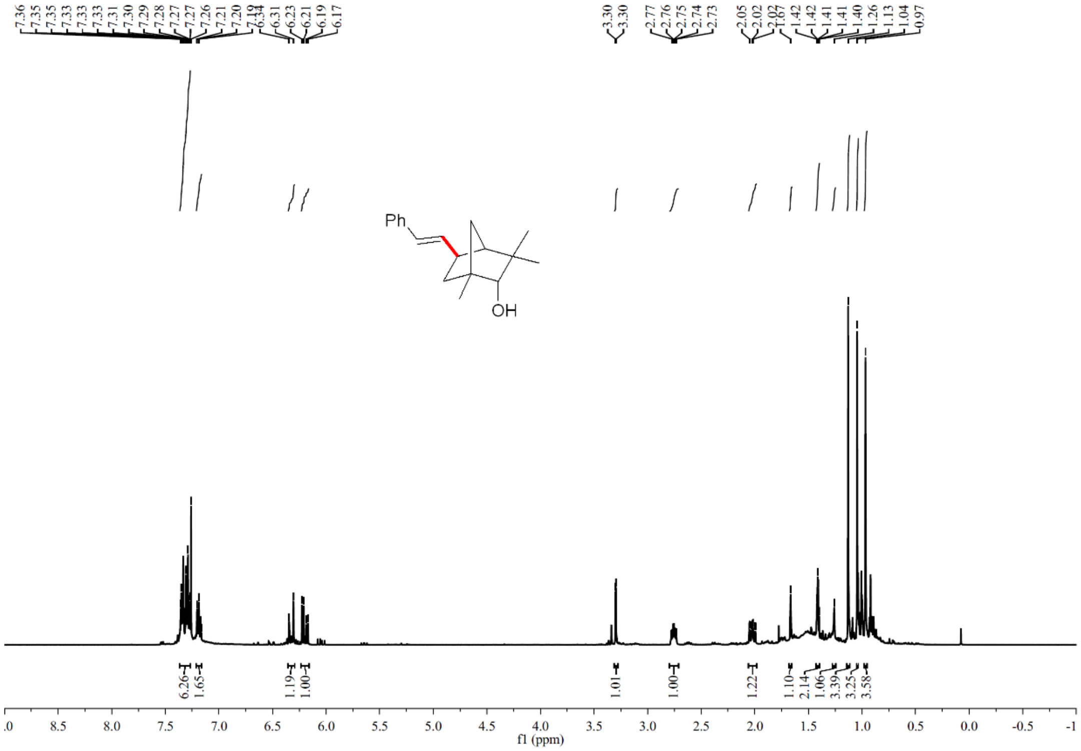

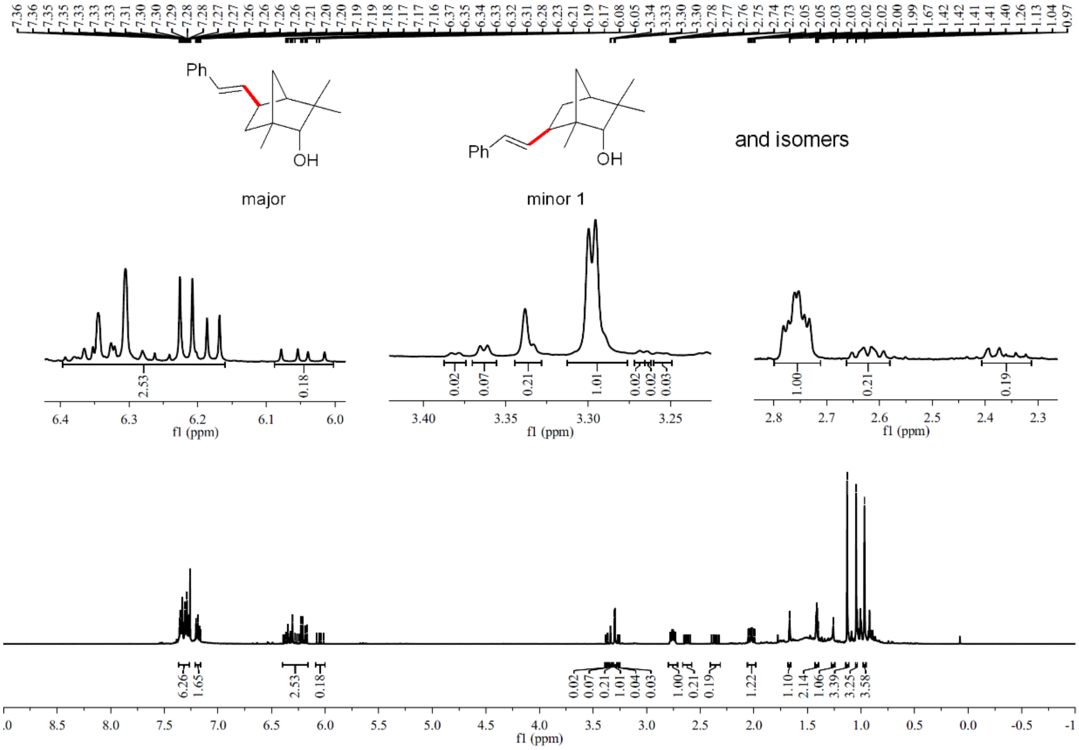

major
minor 1
and isomers
f1 (ppm)

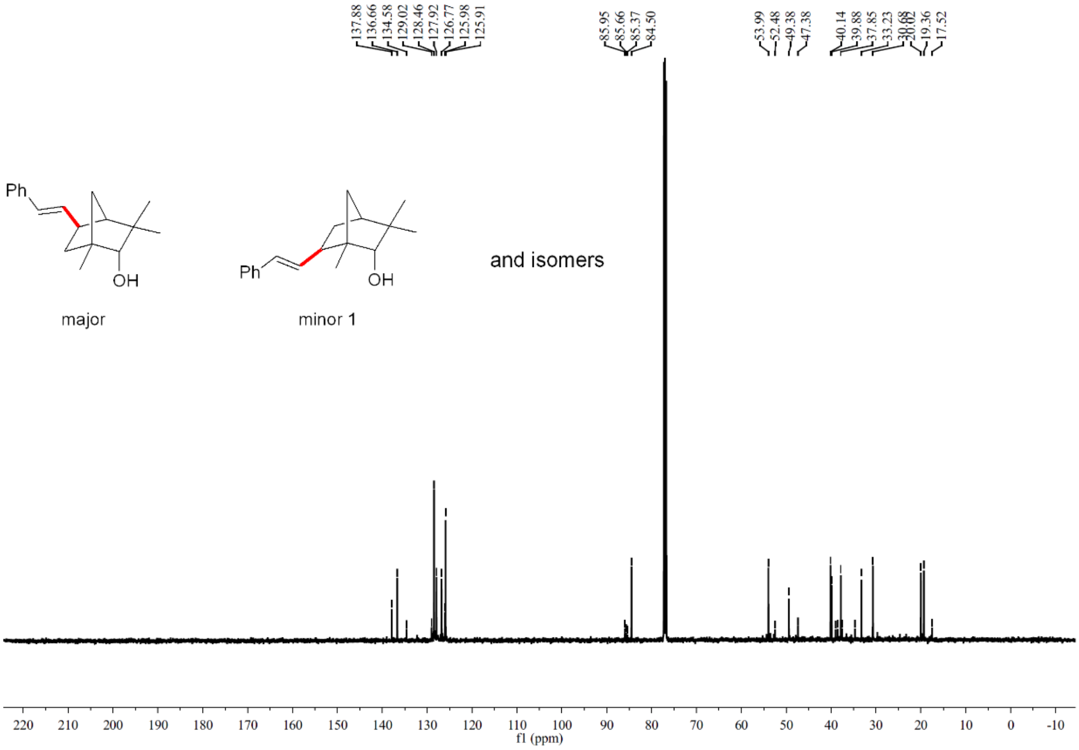

137.88
136.66
134.58
129.02
128.46
127.92
126.77
125.98
125.91
85.95
85.66
85.37
84.50
53.99
52.48
49.38
47.38
40.14
39.88
37.85
33.23
19.36
17.52
major
minor 1
and isomers
f1 (ppm)

key COSY correlations

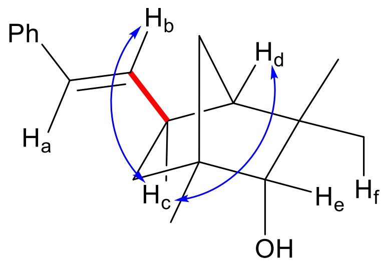

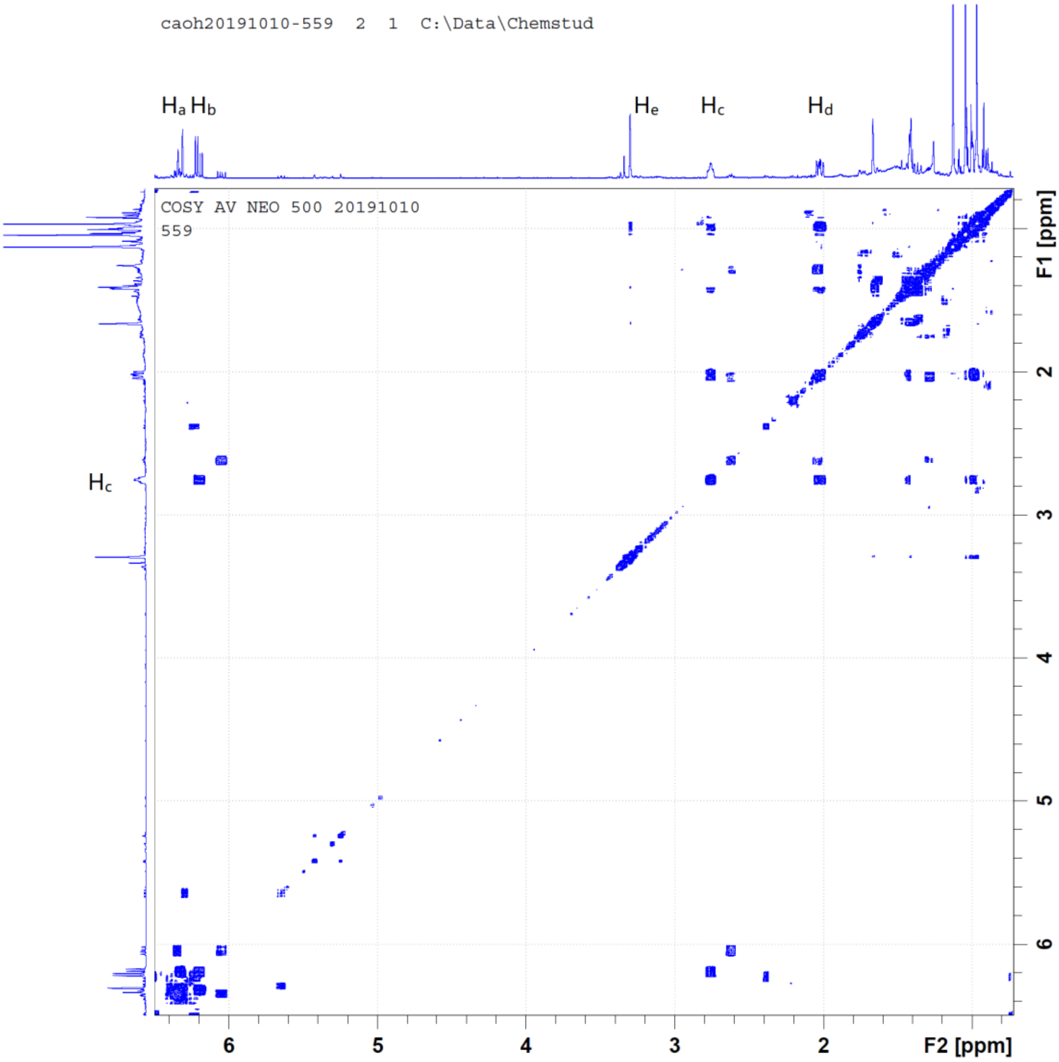

key NOESY correlations

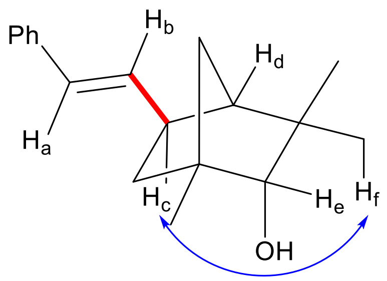

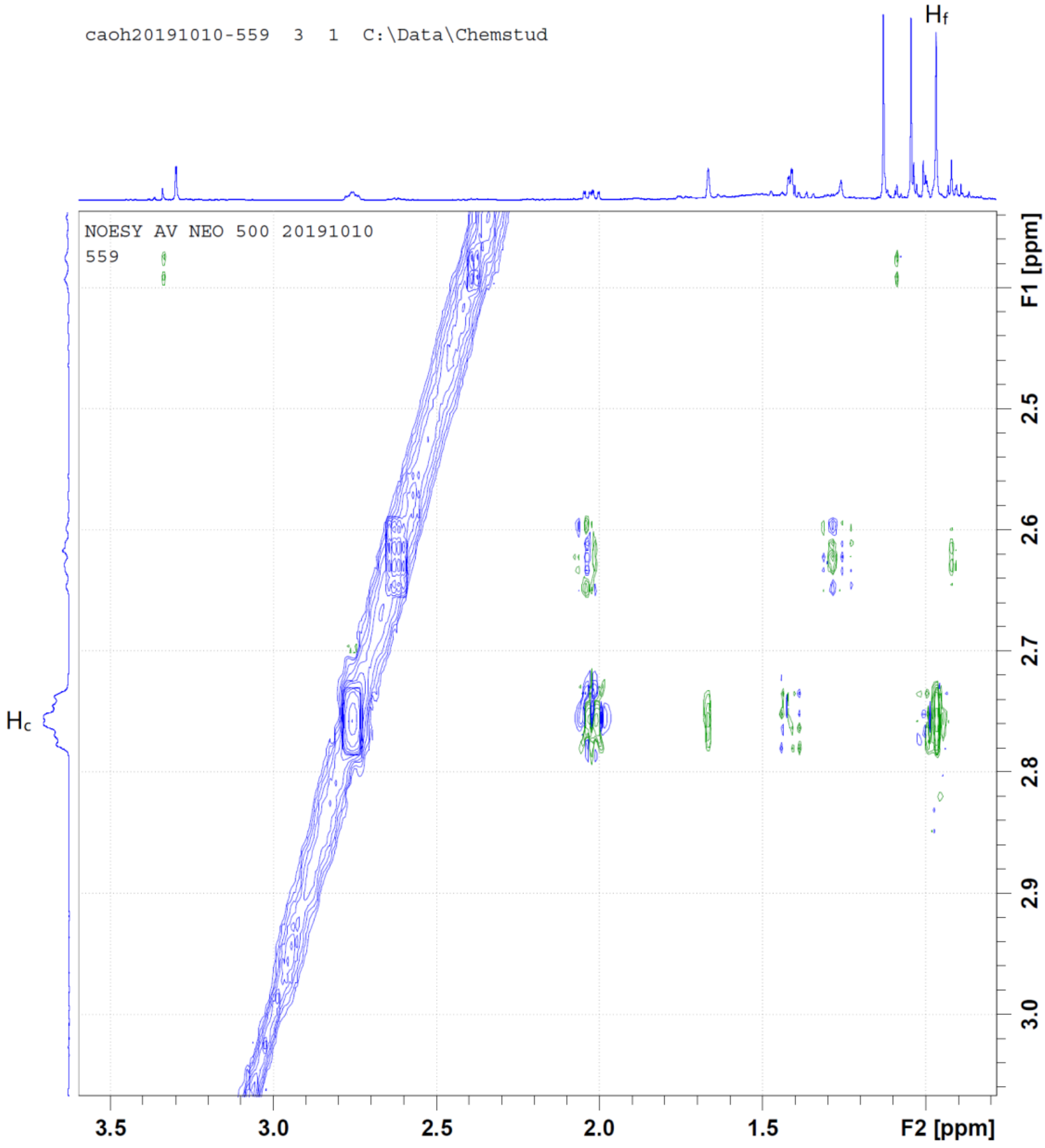

full NOESY spectrum

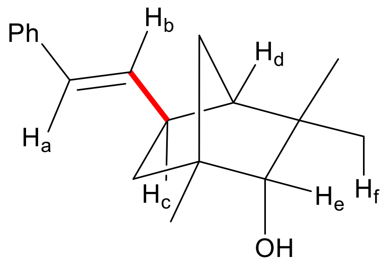

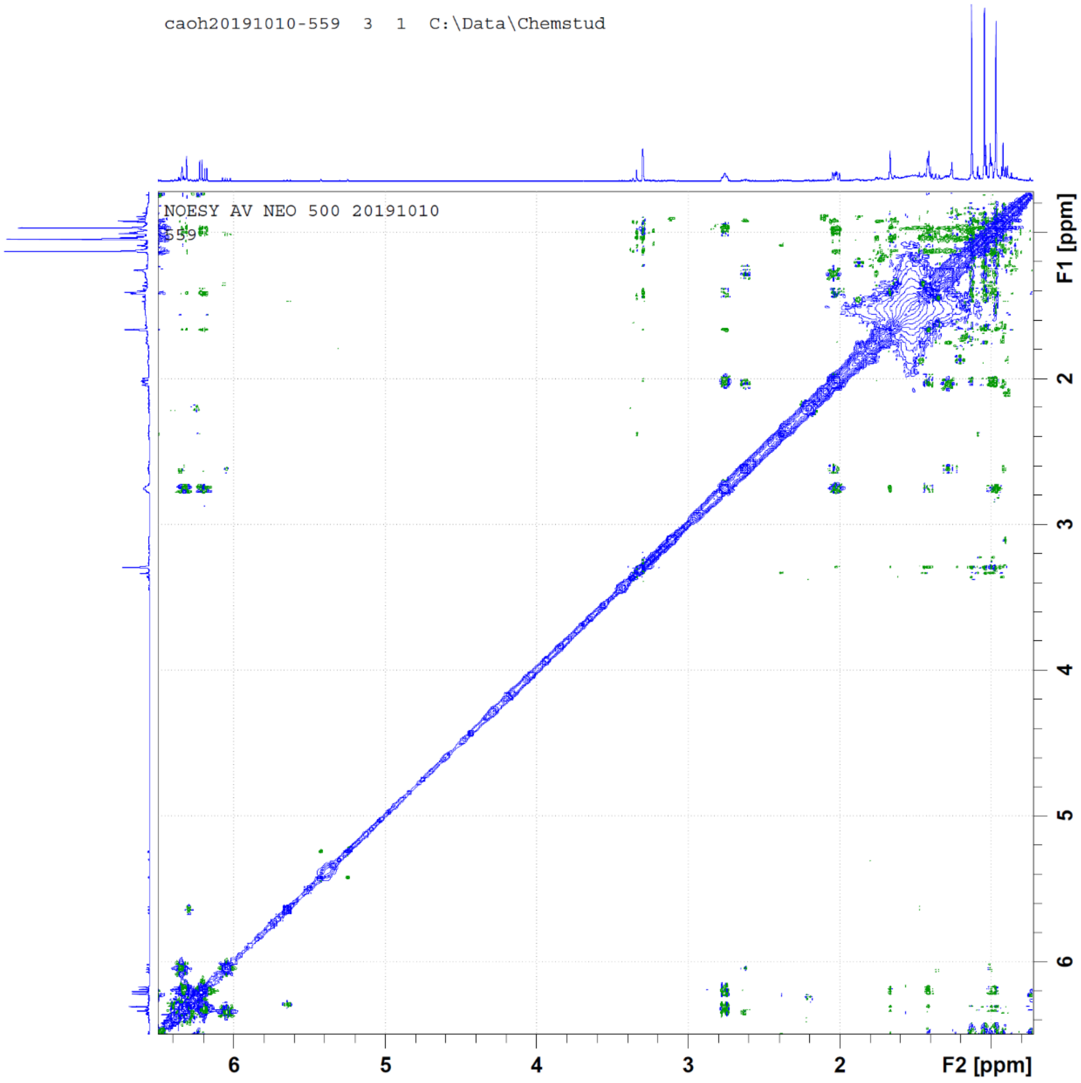

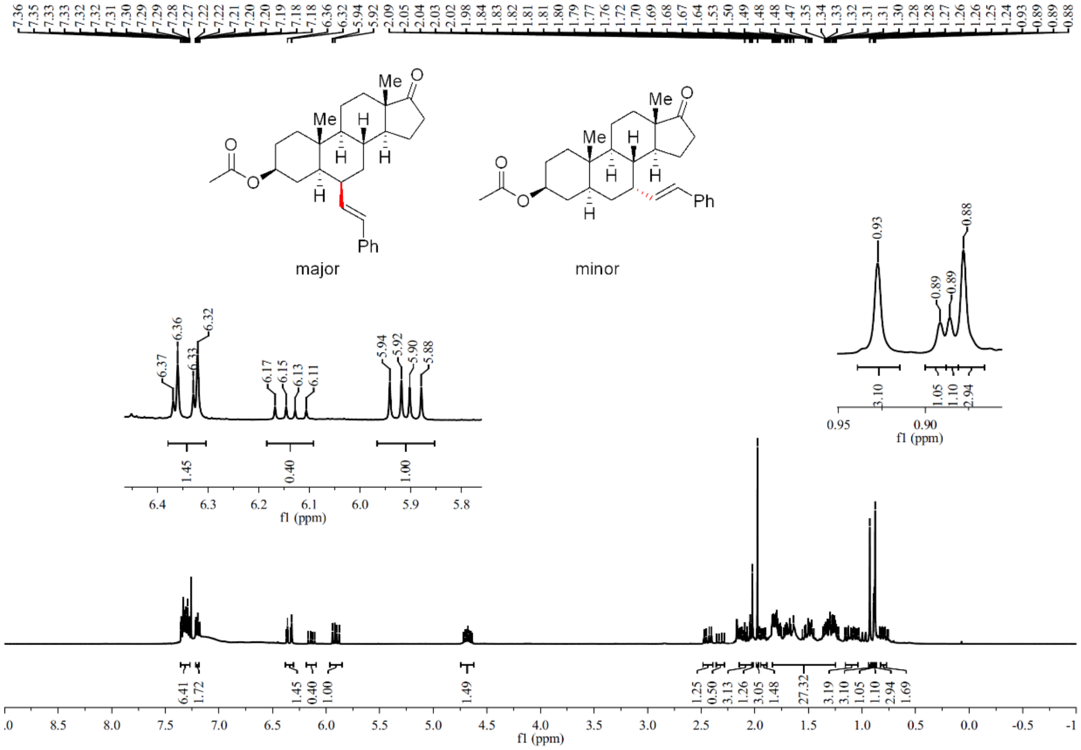

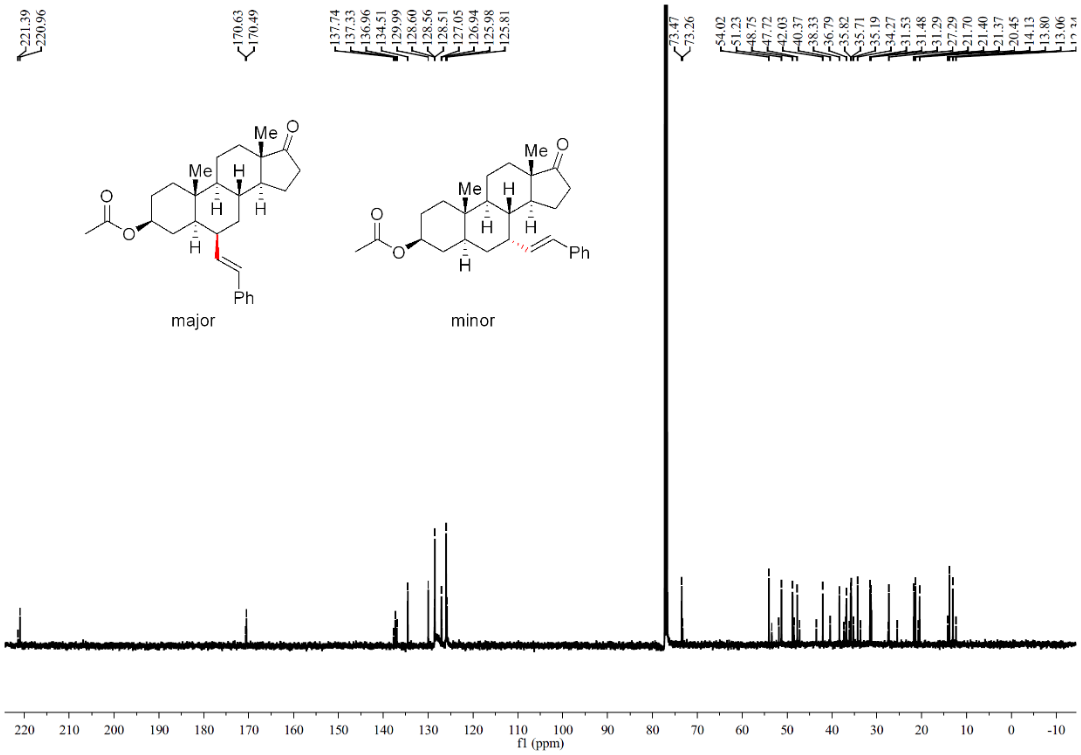

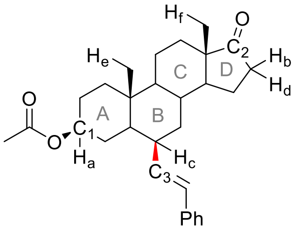

major

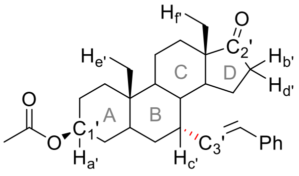

minor

Key ^{1}H NMR and ^{13}C signals:

H_a $H_{a'}$	4.74 - 4.62 ppm (m)
H_b	2.44 ppm (dd, J = 19.6, 8.4 Hz)
$H_{b'}$	2.32 ppm (dd, J = 19.6, 8.0 Hz)
H_c $H_{c'}$	2.15 - 2.03 ppm (m)
H_d $H_{d'}$	2.15 - 2.03 ppm (m)
H_e H_f	0.93 0.88 ppm (s)
$H_{e'}$ $H_{f'}$	0.89 0.89 ppm (s)
C_1	170.49 ppm
C_2	220.96 ppm
C_1'	170.63 ppm
C_2'	221.39 ppm

Key finding from COSY, NOESY, HMQC and HMBC spectra:

1) No COSY or NOESY correlations were found between H_a and H_c. No COSY or NOESY correlations were found between $H_{a'}$ and $H_{c'}$ (Alkenylation on A ring unlikely).
2) No HMBC correlations were found between C_1 and H_c. No HMBC correlations were found between $C_{1'}$ and $H_{c'}$ (Alkenylation on A ring unlikely).
3) No COSY correlations were found between H_c with H_e or H_f. No COSY correlations were found between $H_{c'}$ with $H_{e'}$ or $H_{f'}$ (Alkenylation on C ring unlikely).
4) No HMBC correlations were found between C_2 and H_c. No HMBC correlations were found between $C_{2'}$ and $H_{c'}$ (Alkenylation on D ring unlikely).
5) No HMBC correlations were found between C_3 and H_b. No HMBC correlations were found between $C_{3'}$ and $H_{b'}$ (Alkenylation on D ring unlikely).

full COSY spectrum

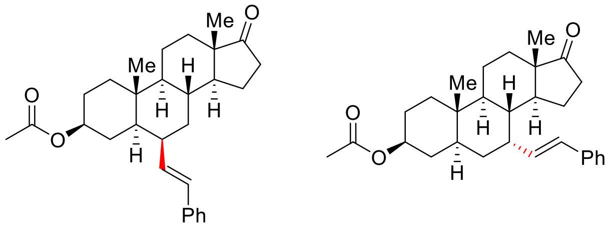

major minor

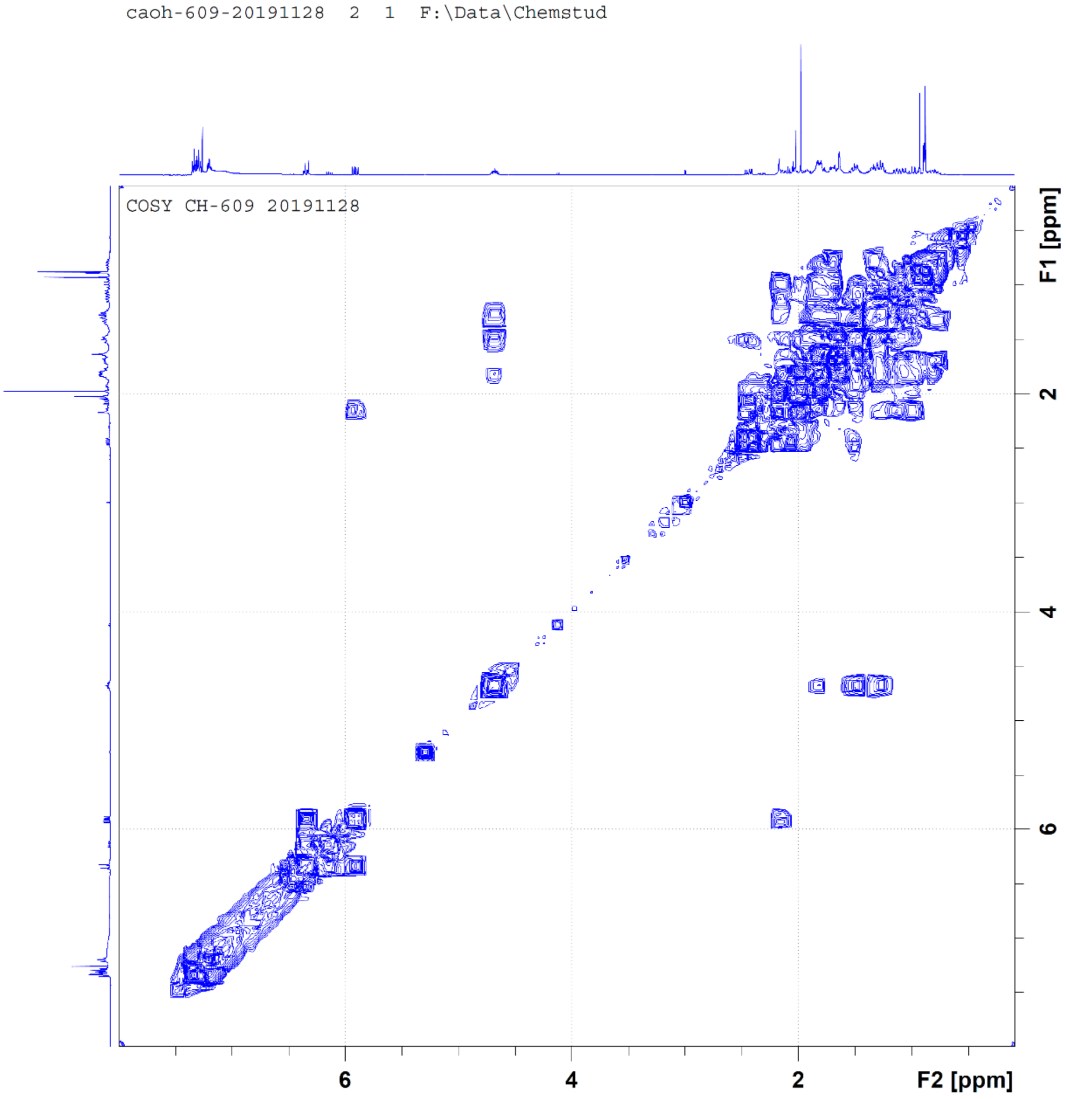

full NOESY spectrum

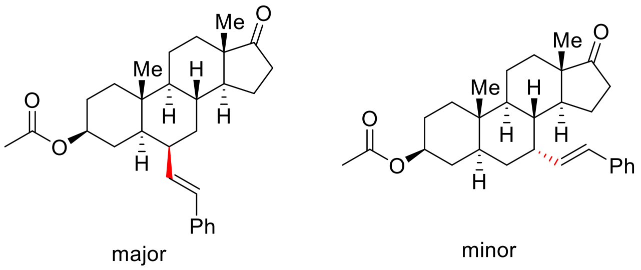

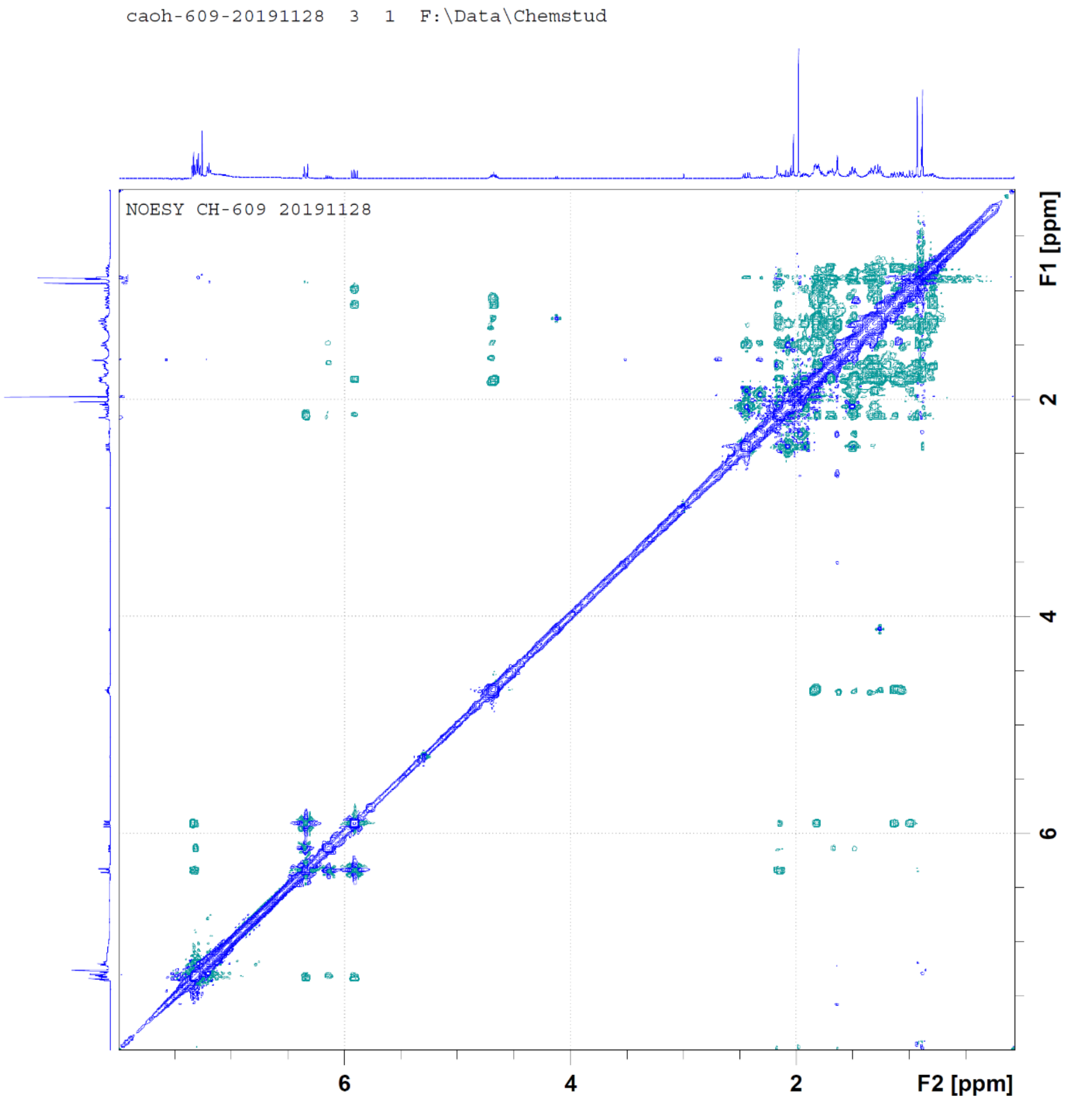

full HMQC spectrum

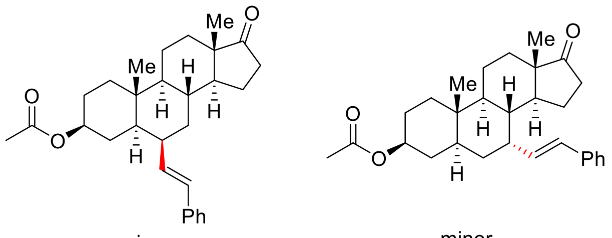

major minor

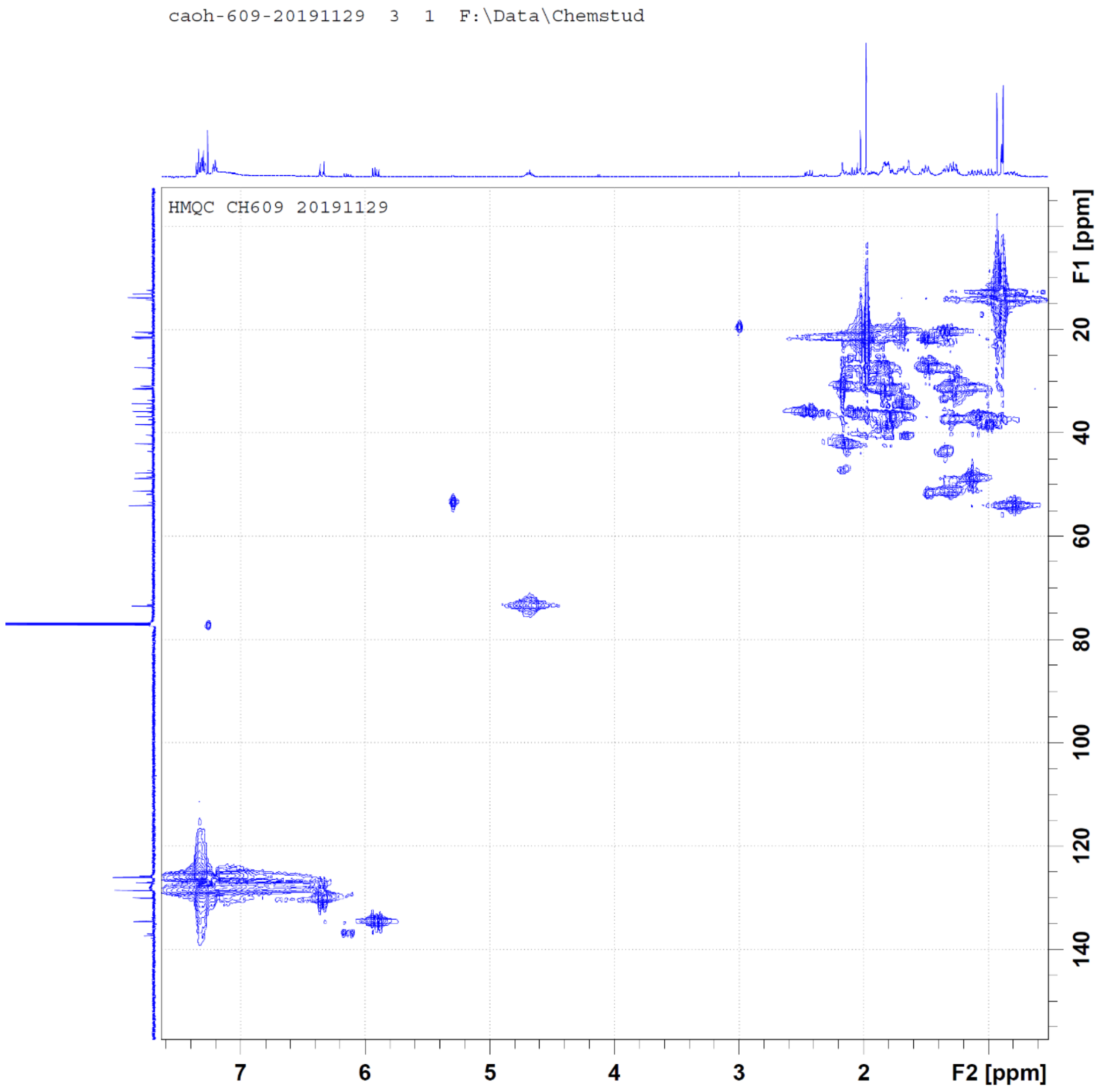

full HMBC spectrum

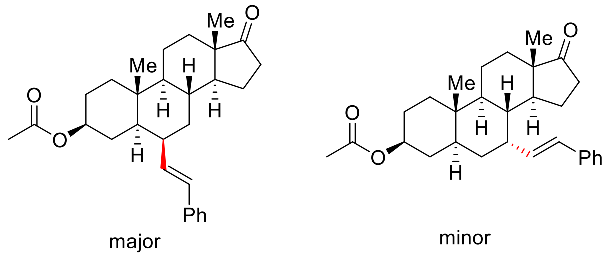

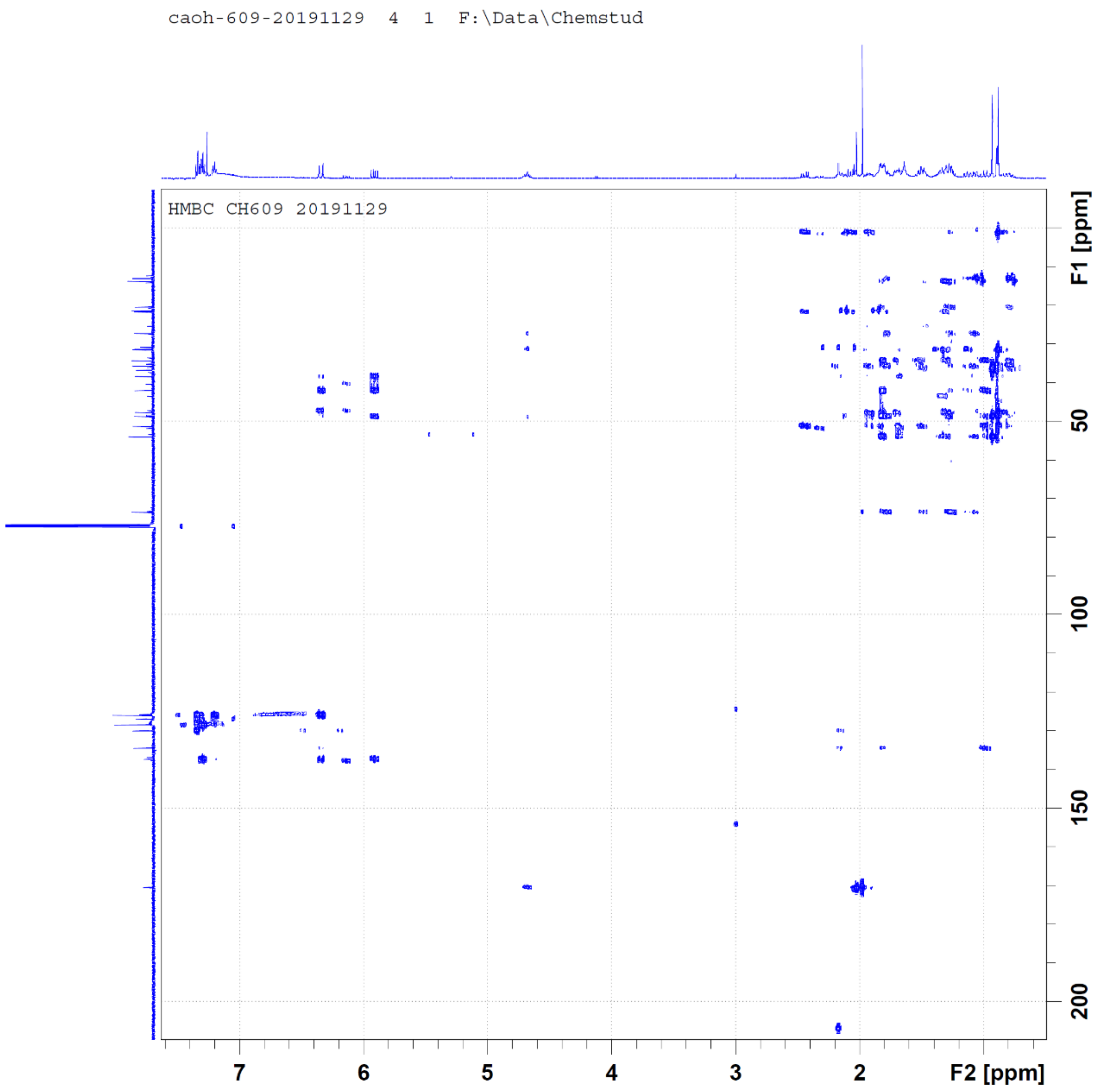

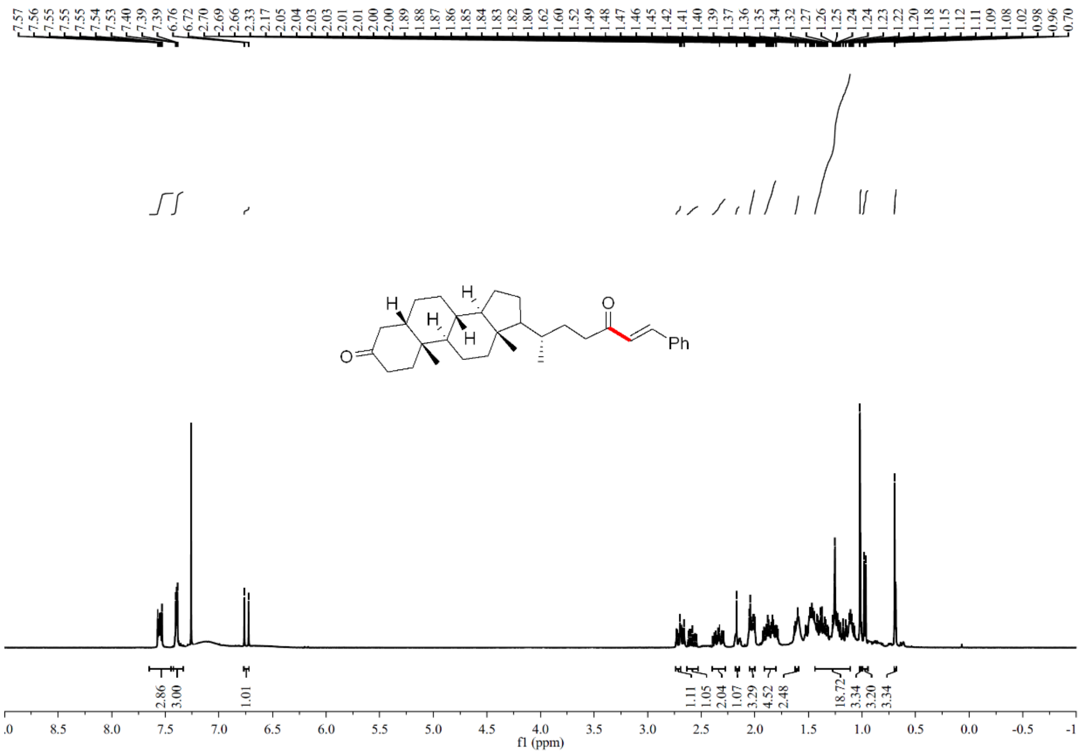

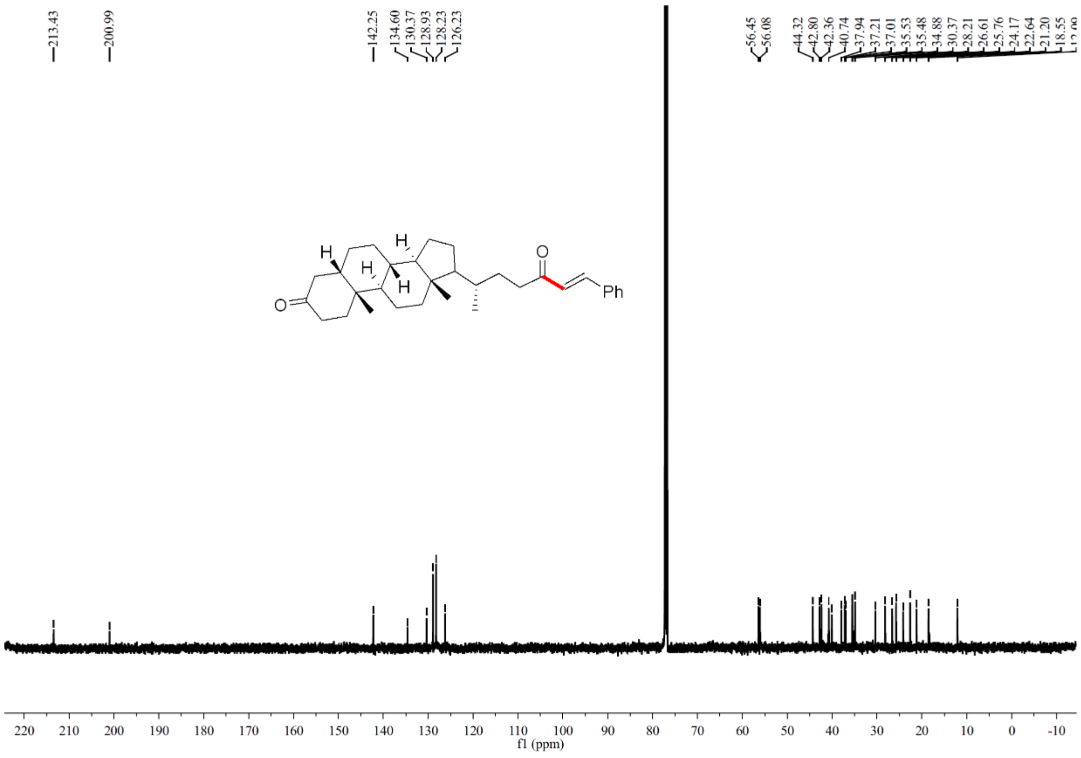

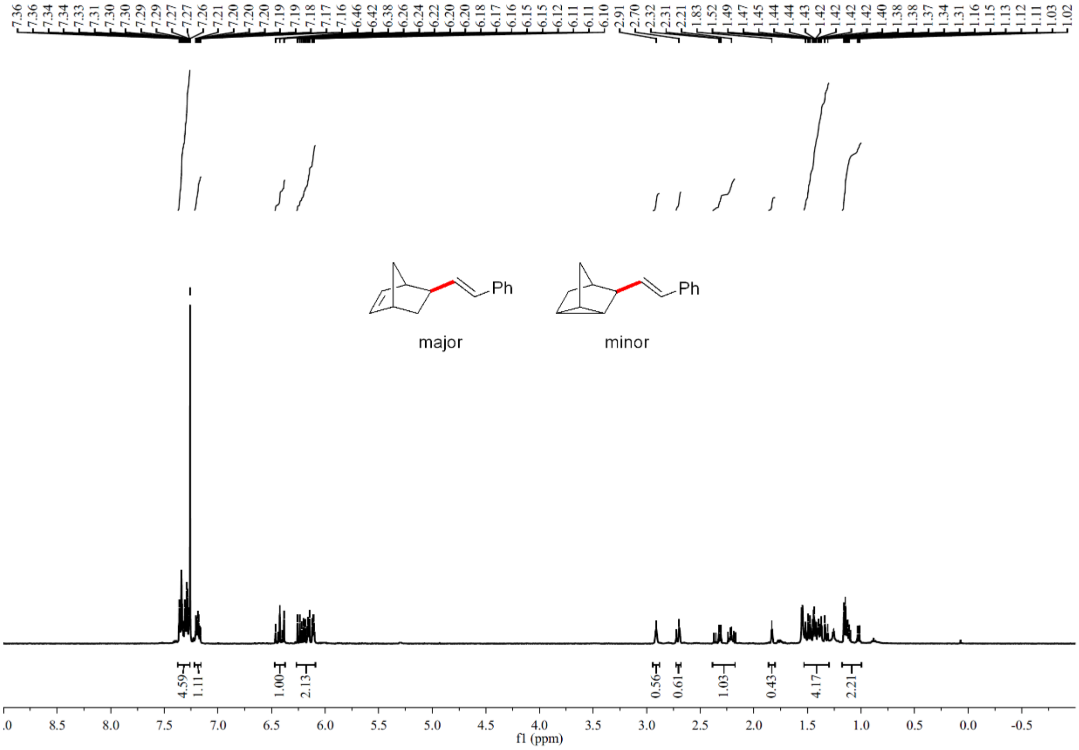
Ph
Ph
major
minor
f1 (ppm)

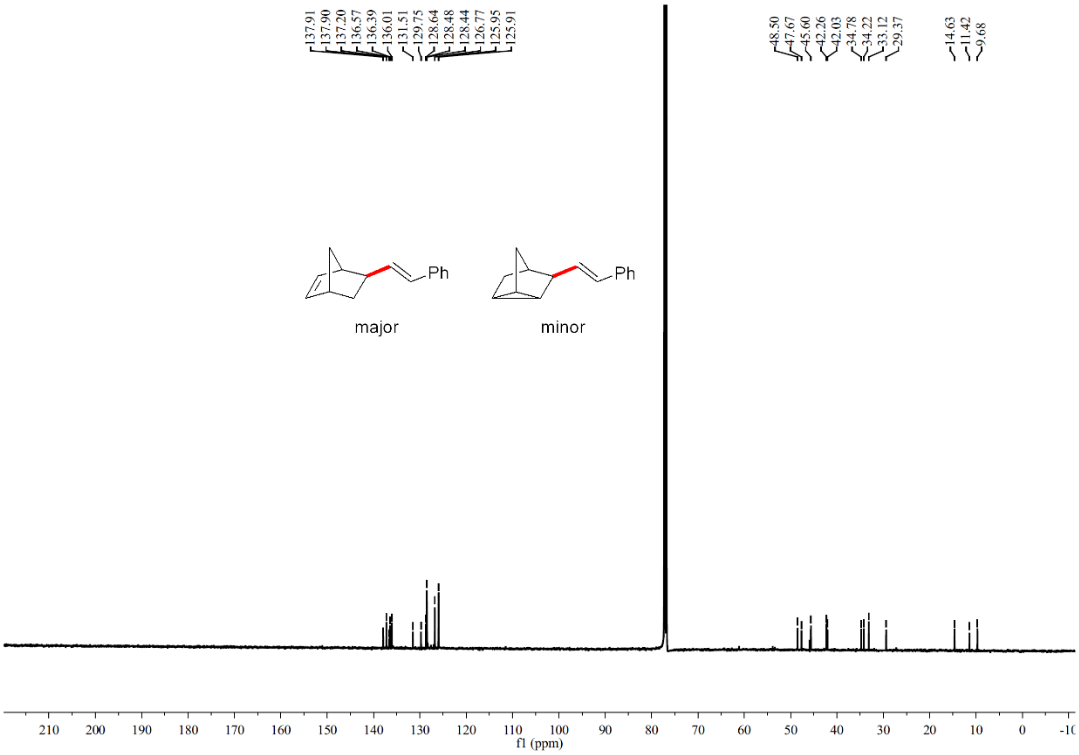
137.91
137.90
137.20
136.57
136.39
136.01
131.51
129.75
128.64
128.48
128.44
126.77
125.95
125.91
48.50
47.67
45.60
42.26
42.03
34.78
34.22
33.12
29.37
14.63
11.42
9.68
Ph
Ph
major
minor
f1 (ppm)

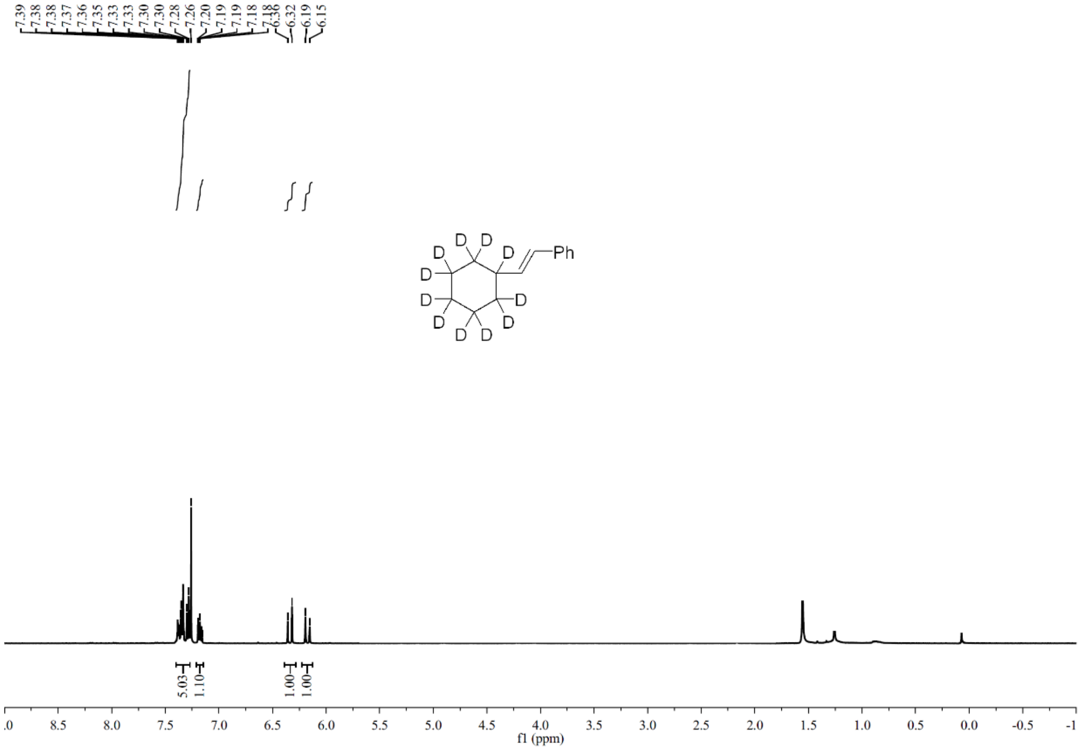

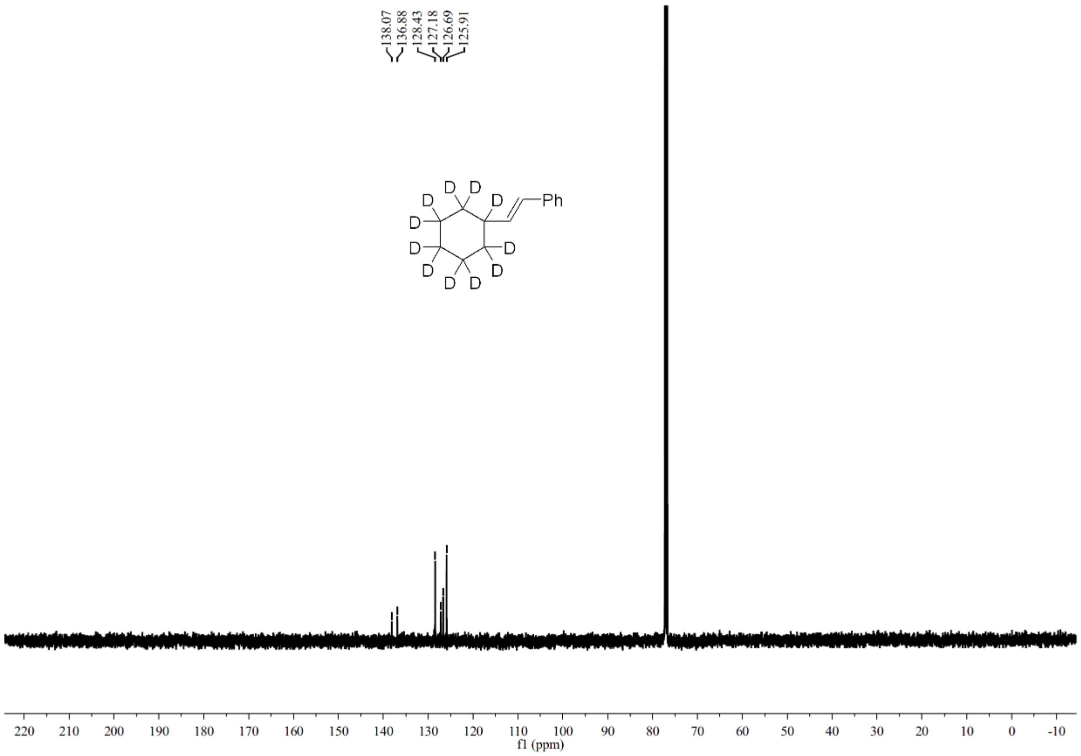

Supplementary References

1. Yamase, T., Takabayashi, N. & Kaji, M. Solution photochemistry of tetrakis(tetrabutylammonium) decatungstate(VI) and catalytic hydrogen evolution from alcohols. *J. Chem. Soc. Dalton Trans.* **5**, 793-799 (1984).
2. Du, P., Schneider, J., Luo, G., Brennessel, W. W. & Eisenberg, R. Visible light-driven hydrogen production from aqueous protons catalyzed by molecular cobaloxime catalysts. *Inorg. Chem.* **48**, 4952-4962 (2009).
3. Thiebes, C., Surya Prakash, G. K., Petasis, N. A. & Olah, G. A. Mild preparation of haloarenes by *ipso*-substitution of arylboronic acids with *N*-halosuccinimides. *Synlett*, 141-142 (1998).
4. Kurandina, D., Parasram, M. & Gevorgyan, V. Visible light- induced room- temperature Heck reaction of functionalized alkyl halides with vinyl arenes/heteroarenes. *Angew. Chem. Int. Ed.* **56**, 14212-14216 (2017).
5. Tortajada, A., Ninokata, R. & Martin, R. Ni-catalyzed site-selective dicarboxylation of 1,3-dienes with CO_2. *J. Am. Chem. Soc.* **140**, 2050-2053 (2018).
6. Lan, Y. *et. al.* Copper-catalyzed silylperoxidation reaction of *α,β*-unsaturated ketones, esters, amides, and conjugated enynes. *ACS Catal.* **7**, 7120-7125 (2017).
7. Purushottamachar, P. & Njar, V. C. O. A new simple and high-yield synthesis of 5α-dihydrotestosterone (DHT), a potent androgen receptor agonist. *Steroids* **77**, 1530-1534 (2012).
8. Nahar, L. & Turner, A. B. Synthesis of dinorcholane and 5*β*-cholane derivatives. *J. Chem. Res.* **11**,747-749 (2004).
9. Zheng, Y.-W. *et. al.* Photocatalytic hydrogen-evolution cross-couplings: benzene C-H amination and hydroxylation. *J. Am. Chem. Soc.* **138**, 10080-10083 (2016).
10. Hu, X., Zhang, G., Bu, F. & Lei, A. Selective oxidative [4+2] imine/alkene annulation with H_2 liberation induced by photo-oxidation. *Angew. Chem. Int. Ed.* **57**, 1286-1290 (2018).
11. Toscano, P. J., Swider, T. F., Marzilli, L. G., Bresciani-Pahor, N. & Randaccio, L. Organocobalt B_{12} models. An assessment of the cis effect using bis(glyoximato) as a sterically minimal equatorial atom set. Structures of *trans*-bis(glyoximato)methyl(L)cobalt(III) with L = triphenylphosphine and trimethyl phosphite. *Inorg. Chem.* **22**, 3416-3421 (1983).
12. López, C., Alvarez, S., Aguiló, M., Solans, X. & Font-Altaba, M. Synthesis and structure of

chloro(ligand)bis(diphenylglyoximato)cobalt(III) complexes. *Inorg. Chim. Acta* **127**, 153-159 (1987).

13. Du, P., Knowles, K. & Eisenberg, R. A homogeneous system for the photogeneration of hydrogen from water based on a platinum(II) terpyridyl acetylide chromophore and a molecular cobalt catalyst. *J. Am. Chem. Soc.* **130**, 12576-12577 (2008).
14. Lazarides, T. *et al.* Making hydrogen from water using a homogeneous system without noble metals. *J. Am. Chem. Soc.* **131**, 9192-9194 (2009).
15. De Waele, V., Poizat, O., Fagnoni, M., Bagno, A. & Ravelli, D. Unraveling the key features of the reactive state of decatungstate anion in hydrogen atom transfer (HAT) photocatalysis. *ACS Catal.* **6**, 7174-7182 (2016).
16. West, J. G., Huang, D. & Sorensen, E. J. Acceptorless dehydrogenation of small molecules through cooperative base metal catalysis. *Nat. Commun.* **6**, 10093 (2015).
17. Frisch, M. J. *et. al.*, Gaussian 16 revision a. 03; gaussian inc. *Wallingford CT*, 2(3), p.4. (2016).
18. Liu, X.. Zhang, J.. Vazquez, A.. Wang, D. & Li, S. *Phys. Chem. Chem. Phys.* **20**, 18782-18789 (2018).
19. Powell, D. A., Maki, T. & Fu, G. C. Stille cross-couplings of unactivated secondary alkyl halides using monoorganotin reagents. *J. Am. Chem. Soc.* **127**, 510-511 (2005).
20. Ho, C.-Y. & He, L. Catalytic intermolecular tail- to- tail hydroalkenylation of styrenes with *α* olefins: regioselective migratory insertion controlled by a nickel/*N*- heterocyclic carbene. *Angew. Chem. Int. Ed.* **49**, 9182-9186 (2010).
21. Delcamp, J. H. & White, M. C. Sequential hydrocarbon functionalization: allylic C-H oxidation/vinylic C-H arylation. *J. Am. Chem. Soc.* **128**, 15076-15077 (2006).
22. Denmark, S. E. & Kallemeyn, J. M. Stereospecific palladium-catalyzed cross-coupling of (*E*)- and (*Z*)-alkenylsilanolates with aryl chlorides. *J. Am. Chem. Soc.* **128**, 15958-15959 (2006).
23. Zou Y. & Zhou, J. Palladium-catalyzed intermolecular Heck reaction of alkyl halides. *Chem. Commun.* **50**, 3725-3728 (2014).
24. Zhang, N., Quan, Z.-J. & Wang, X.-C. Nickel- catalyzed denitrated coupling reaction of nitroalkenes with aliphatic and aromatic alkenes. *Adv. Synth. Catal.* **358**, 3179-3183 (2016).
25. Dong, D.-J., Li, H.-H. & Tian, S.-K. A highly tunable stereoselective olefination of

semistabilized triphenylphosphonium ylides with *N*-sulfonyl imines. *J. Am. Chem. Soc.* **132**, 5018-5020 (2010).

26. Zhu, Y. & Wei, Y. Copper catalyzed direct alkenylation of simple alkanes with styrenes. *Chem. Sci.* **5**, 2379-2382 (2014).
27. Zhang, L. *et. al.* Palladium- catalyzed regioselective and stereoselective oxidative Heck arylation of allylamines with arylboronic acids. *Adv. Synth. Catal.* **355**, 1570-1578 (2013).
28. Czaplyski, W. L., Na, C. G. & Alexanian, E. J. C-H xanthylation: A synthetic platform for alkane functionalization. *J. Am. Chem. Soc.* **138**, 13854-13857 (2016).
29. Jang, Y.-J., Yan, M.-C., Lin, Y.-F. & Yao, C.-F. A simple radical addition-elimination route to geometrically pure (*E*)-alkene and chromanone derivatives via *β*-nitrostyrene. *J. Org. Chem.* **69**, 3961-3963 (2004).
