# Supplementary material for: Photoinduced site-selective alkenylation of alkanes and aldehydes with aryl alkenes
Source: Nat Commun. 2020 Apr 23;11:1956. doi: 10.1038/s41467-020-15878-6 (PMC7181776; doi:10.1038/s41467-020-15878-6)
Supplement: Supplementary file 3 — Description of Additional Supplementary Files [file 41467_2020_15878_MOESM3_ESM.pdf]

Description of Additional Supplementary Files

File Name: Supplementary Data 1
Description: Calculated Cartesian Coordinates Data
