# Supplementary material for: Photoinduced site-selective alkenylation of alkanes and aldehydes with aryl alkenes
Source: Nat Commun. 2020 Apr 23;11:1956. doi: 10.1038/s41467-020-15878-6 (PMC7181776; doi:10.1038/s41467-020-15878-6)
Supplement: Supplementary file 4 — Supplementary Data 1 [file 41467_2020_15878_MOESM4_ESM.zip › Supplementary Data.docx]

**Calculated Cartesian Coordinates**

Structure 1. Hydrogen radical

H 0.00000000 0.00000000 0.00000000


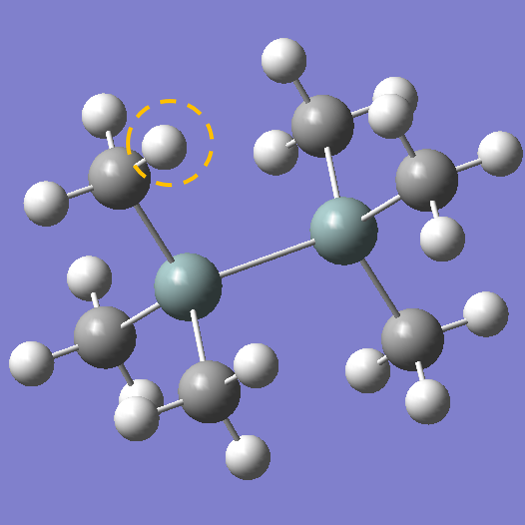


Structure 2. Hexamethyldisilane

Si -1.18657875 -0.00004838 -0.00022759

Si 1.18657907 0.00004614 0.00022430

C -1.85052705 1.51115851 -0.94005779

H -1.51537549 1.51296288 -1.98160700

H -2.94597459 1.51166324 -0.94108691

H -1.51640125 2.44594168 -0.47991747

C -1.85049705 -1.56961390 -0.83889520

H -1.51560580 -2.47228862 -0.31907670

H -2.94594725 -1.57055228 -0.83902981

H -1.51615230 -1.63904322 -1.87839728

C -1.85148455 0.05840937 1.77814202

H -1.51889485 0.96094551 2.29968139

H -2.94693616 0.05648711 1.77835030

H -1.51563041 -0.80601425 2.35878119

C 1.85052763 -1.51072046 0.94076450

H 1.51632148 -2.44572768 0.48113755

H 2.94597525 -1.51127807 0.94170869

H 1.51545751 -1.51196630 1.98234081

C 1.85049262 1.57000158 0.83816491

H 2.94594279 1.57087337 0.83843124

H 1.51571840 2.47242196 0.31783008

H 1.51602652 1.64000053 1.87758978

C 1.85148816 -0.05923322 -1.77811502

H 1.51551333 0.80484259 -2.35920267

H 2.94693955 -0.05716074 -1.77832646

H 1.51902035 -0.96208753 -2.29918121

Structure 3. The radical of hexamethyldisilane generated after H-atom abstraction.

Si -1.15941801 0.00024494 -0.01044991

Si 1.22677776 0.00029849 0.05683852

C -1.83772144 -1.54350281 0.85938017

H -1.53494450 -1.57279111 1.91021872

H -2.93262714 -1.55143643 0.82445181

H -1.48121436 -2.46095893 0.38164365

C -1.83979353 1.54314544 0.85911396

H -1.48567022 2.46101747 0.38040706

H -2.93474121 1.54868595 0.82526953

H -1.53602534 1.57380377 1.90962725

C -1.76369865 -0.00040087 -1.81087213

H -1.41293357 -0.88521239 -2.35030152

H -2.85888423 -0.00007768 -1.84355830

H -1.41243401 0.88361901 -2.35128400

C 1.89580959 1.55275257 -0.80644341

H 1.55998403 2.46259499 -0.30039847

H 2.99046295 1.55184357 -0.80878438

H 1.55644855 1.60180372 -1.84539154

C 1.89469332 -1.55233319 -0.80703044

H 2.98936230 -1.55249780 -0.80834787

H 1.55758856 -2.46219820 -0.30188072

H 1.55627650 -1.60038256 -1.84632172

C 1.76392563 -0.00036297 1.83121086

H 1.87925518 -0.91362277 2.41099692

H 1.87777050 0.91241231 2.41205899


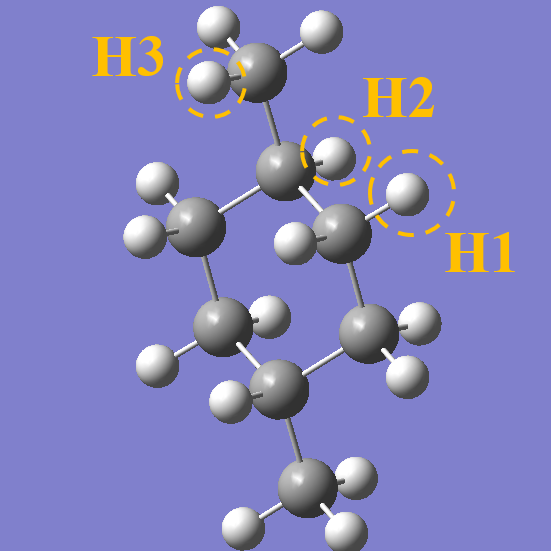


Structure 4. trans-1,4-Dimethylcyclohexane

C 0.74433781 1.25453866 0.17640151

C 1.45534772 -0.00000993 -0.34181644

C 0.74432665 -1.25453361 0.17644253

C -0.74432576 -1.25453556 -0.17643252

C -1.45535014 -0.00001062 0.34181779

C -0.74433709 1.25453973 -0.17639120

H 1.23146300 -2.15202187 -0.22516261

H 1.37683874 -0.00002771 -1.44028625

H 0.85846580 1.29427661 1.27041397

H 1.23144228 2.15199913 -0.22530671

H -0.85838482 -1.29419736 -1.27045657

H -1.23146062 -2.15202155 0.22517946

H -1.37685852 -0.00002847 1.44028854

H -0.85846431 1.29428426 -1.27040345

H -1.23144270 2.15199740 0.22532198

H 0.85838676 -1.29418739 1.27046684

C -2.93212901 -0.00000130 -0.04094252

H -3.44507607 -0.88645056 0.34876336

H -3.44506319 0.88645164 0.34877220

H -3.04608517 0.00000536 -1.13218361

C 2.93213055 -0.00000150 0.04092359

H 3.44507216 -0.88645087 -0.34878915

H 3.44505956 0.88645149 -0.34879763

H 3.04610278 0.00000472 1.13216321

Structure 5. The radical of trans-1,4-dimethylcyclohexane generated after methylene hydrogen abstraction.

C 0.71366755 1.23122309 0.17776559

C 1.44052729 -0.00838202 -0.35477916

C 0.74311745 -1.28259163 0.17319655

C -0.72454763 -1.24871194 -0.10019129

C -1.48314750 -0.03863830 0.34779208

C -0.77450113 1.22875826 -0.17745164

H 1.21250512 -2.17369573 -0.25826299

H 1.34884056 -0.00887434 -1.45095917

H 0.82620594 1.25941595 1.27249819

H 1.19388585 2.13705104 -0.21314255

H -1.16201374 -1.91076711 -0.84280941

H -1.43453116 0.01380449 1.44995760

H -0.88940975 1.26327382 -1.27044262

H -1.26765977 2.12136325 0.22809652

H 0.92320868 -1.31945461 1.26202715

C -2.94816292 -0.07699118 -0.07708895

H -3.45239886 -0.96444803 0.32011576

H -3.48705617 0.80847579 0.27641075

H -3.02725518 -0.10640353 -1.17042227

C 2.91935056 0.00466617 0.01771608

H 3.43645054 -0.87792467 -0.37500144

H 3.42082475 0.89494946 -0.37786429

H 3.04058104 0.00723948 1.10804322

Structure 6. The radical of trans-1,4-dimethylcyclohexane generated after methine hydrogen abstraction.

C -0.71867299 1.25163002 -0.20335445

C -1.43497994 -0.00000145 0.31201219

C -0.71866973 -1.25162834 -0.20336015

C 0.75731348 -1.27645185 0.21232413

C 1.46888195 0.00000017 -0.12253428

C 0.75731415 1.27645439 0.21231879

H -1.22644115 -2.15331321 0.16034448

H -1.36809184 -0.00000415 1.41159020

H -0.78754844 1.26376851 -1.30075306

H -1.22644228 2.15331090 0.16036330

H 0.80704567 -1.44595563 1.30535900

H 1.27108271 -2.13032871 -0.24713992

H 0.80705563 1.44596759 1.30535143

H 1.27107941 2.13032763 -0.24715683

H -0.78753711 -1.26375601 -1.30075985

C 2.96184965 -0.00000154 -0.15293011

H 3.35662608 -0.88915771 -0.65813828

H 3.35662821 0.88916141 -0.65812402

H 3.38674404 -0.00001129 0.86645223

C -2.90712092 -0.00000204 -0.08873073

H -3.42430598 -0.88660059 0.29473333

H -3.42430644 0.88659366 0.29473899

H -3.00708234 0.00000141 -1.18133327

Structure 7. The radical of trans-1,4-dimethylcyclohexane generated after methyl hydrogen abstraction.

C -0.68447419 -1.25752089 0.15928958

C -1.40401253 -0.00157163 -0.34261091

C -0.70066063 1.25067240 0.19092736

C 0.78732985 1.26845069 -0.16191149

C 1.51157038 0.01298506 0.34412633

C 0.80250321 -1.24742680 -0.20050679

H -1.19335754 2.15001360 -0.19941861

H -1.32719468 0.01247834 -1.44100570

H -0.79290889 -1.31030852 1.25325886

H -1.16687590 -2.15369701 -0.25099641

H 0.90352753 1.31872701 -1.25487831

H 1.26817248 2.16386426 0.24974312

H 1.41989138 -0.01681595 1.44126536

H 0.91731173 -1.26386204 -1.29391313

H 1.29861315 -2.14525395 0.18662095

H -0.81465560 1.27623459 1.28534096

C 2.95625955 0.02371417 -0.02381187

H 3.67314688 -0.57569707 0.52789276

H 3.26310482 0.40395820 -0.99422421

C -2.88020576 -0.01601835 0.04268936

H -3.39894716 0.87226961 -0.33471798

H -3.38770431 -0.90055417 -0.35808331

H -2.99198321 -0.03106478 1.13396632


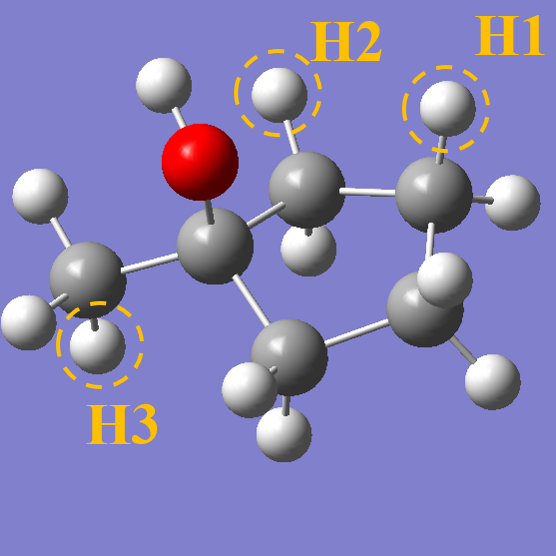


Structure 8. 1-Methylcyclopentanol

C -1.61926106 0.78727220 0.06376668

C -1.65347820 -0.76443643 -0.01789027

C -0.21433118 -1.23083732 0.28509927

C 0.66090176 -0.01187772 -0.00832326

C -0.19772709 1.13732494 0.53290114

H -1.79910114 1.21816548 -0.92495819

H -2.38283736 1.18628182 0.73702656

H -1.96046903 -1.08647148 -1.01658814

H -2.36656865 -1.19387121 0.69137382

H -0.10534938 -1.49339082 1.34327217

H 0.09916299 -2.09312196 -0.31157356

H -0.13830327 1.14132120 1.62802730

H 0.15342455 2.11262438 0.17557043

C 2.06034415 -0.08295164 0.58206827

H 2.59976199 -0.94567953 0.17722248

H 2.62782631 0.82209924 0.33340250

H 2.02469676 -0.17296135 1.67245044

O 0.73196339 0.07321177 -1.43537339

H 1.17335884 0.91234598 -1.64796967

Structure 9. The radical of 1-methylcyclopentanol generated after *γ*-hydroxy methylene hydrogen (remote methylene hydrogen) abstraction.

C -1.61142303 0.82202062 0.07383139

C -1.71020744 -0.67252532 0.00779448

C -0.31364189 -1.13230507 0.46311632

C 0.62367161 -0.01656122 -0.01081420

C -0.19420421 1.26432938 0.26232075

H -2.45237667 1.49492680 -0.04440618

H -1.90697779 -1.01516862 -1.01995235

H -2.51443602 -1.08175919 0.63176853

H -0.27336538 -1.18845455 1.55687231

H -0.01060657 -2.10081968 0.05435960

H -0.00583872 1.61822011 1.28977939

H 0.11451414 2.07972645 -0.40709025

C 1.99485707 -0.02822186 0.64620757

H 2.51309671 -0.96651634 0.42290103

H 2.60754199 0.79966885 0.26863359

H 1.91317527 0.07779391 1.73246411

O 0.75280091 -0.21020917 -1.42153440

H 1.25855305 0.54363638 -1.76779242

Structure 10. The radical of 1-methylcyclopentanol generated after *β*-hydroxy methylene hydrogen abstraction.

C 1.68546016 -0.82115697 0.05464202

C 1.64556076 0.72041738 0.00325479

C 0.26566846 1.06286677 0.57970061

C -0.65913057 -0.01204515 -0.00807395

C 0.24577592 -1.20787410 -0.07937373

H 2.32904800 -1.25828069 -0.71894467

H 2.09235186 -1.16175491 1.02078558

H 1.69581767 1.05475954 -1.03789740

H 2.46873840 1.18678049 0.55094781

H 0.27745029 0.96343319 1.67193978

H -0.08505478 2.06809745 0.32655128

H -0.11534274 -2.22061295 -0.22784046

C -1.94098354 -0.24016167 0.78105696

H -2.52976450 0.68243835 0.82126425

H -2.55071680 -1.01554084 0.30236827

H -1.72039302 -0.56242791 1.80381129

O -0.99187775 0.44548220 -1.33712828

H -1.38121952 -0.31302691 -1.80319972

Structure 11. The radical of 1-methylcyclopentanol generated after methyl hydrogen abstraction.

C 1.58994123 0.77162902 0.00524387

C 1.59586961 -0.78201781 0.00140225

C 0.16728324 -1.20448122 -0.39425435

C -0.70925048 0.00336662 -0.01599513

C 0.18011362 1.17903466 -0.45355417

H 1.77516660 1.14527541 1.01583918

H 2.36325023 1.19153694 -0.64365735

H 1.84403530 -1.16356570 0.99528426

H 2.33521340 -1.18727889 -0.69498382

H 0.09181266 -1.36356650 -1.47523899

H -0.18034805 -2.11105954 0.10970930

H 0.13368558 1.26953368 -1.54523141

H -0.16374127 2.12658036 -0.02401885

C -2.06381085 0.00044661 -0.62903686

H -2.87268044 -0.52150754 -0.12904692

H -2.19718586 0.29210098 -1.66504582

O -0.80075724 -0.03167543 1.41164602

H -1.28402844 0.76748693 1.68038856
